# Choriodecidual Infection Downregulates Angiogenesis and Morphogenesis Pathways in Fetal Lungs from *Macaca Nemestrina*

**DOI:** 10.1371/journal.pone.0046863

**Published:** 2012-10-09

**Authors:** Ryan M. McAdams, Jeroen Vanderhoeven, Richard P. Beyer, Theo K. Bammler, Federico M. Farin, H. Denny Liggitt, Raj P. Kapur, Michael G. Gravett, Craig E. Rubens, Kristina M. Adams Waldorf

**Affiliations:** 1 Department of Pediatrics, University of Washington, Seattle, Washington, United States of America; 2 Department of Obstetrics & Gynecology, University of Washington, Seattle, Washington, United States of America; 3 Department of Environmental and Occupational Health Sciences, University of Washington, Seattle, Washington, United States of America; 4 Department of Comparative Medicine, University of Washington, Seattle, Washington, United States of America; 5 Department of Laboratories, Seattle Children’s, Seattle, Washington, United States of America; 6 Global Alliance to Prevent Prematurity & Stillbirth, Seattle, Washington, United States of America; 7 Division of Infectious Disease, Seattle Children’s, Seattle, Washington, United States of America; Columbia University, United States of America

## Abstract

**Background:**

Intrauterine exposure to amniotic fluid (AF) cytokines is thought to predispose to bronchopulmonary dysplasia (BPD). We evaluated the effects of GBS exposure on RNA expression in fetal lung tissue to determine early molecular pathways associated with fetal lung injury that may progress to BPD.

**Methods:**

Ten chronically catheterized pregnant monkeys (*Macaca nemestrina*) at 118–125 days gestation (term = 172 days) received choriodecidual inoculation of either: 1) Group B Streptococcus (n = 5) or 2) saline (n = 5). Cesarean section and fetal necropsy was performed in the first week after GBS or saline inoculation regardless of labor. RNA was extracted from fetal lungs and profiled by microarray. Results were analyzed using single gene, Gene Set, and Ingenuity Pathway Analysis. Validation was by RT-PCR and immunohistochemistry.

**Results:**

Despite uterine quiescence in most cases, fetal lung injury occurred in four GBS cases (intra-alveolar neutrophils, interstitial thickening) and one control (peri-mortem hemorrhage). Significant elevations of AF cytokines (TNF-α, IL-8, IL-1β, IL-6) were detected in GBS versus controls (p<0.05). Lung injury was not directly caused by GBS, because GBS was undetectable by culture and PCR in the AF and fetal lungs. A total of 335 genes were differentially expressed greater than 1.5 fold (p<0.05) with GBS exposure associated with a striking upregulation of genes in innate and adaptive immunity and downregulation of pathways for angiogenesis, morphogenesis, and cellular growth and development.

**Conclusions:**

A transient choriodecidual infection may induce fetal lung injury with profound alterations in the genetic program of the fetal lung before signs of preterm labor. Our results provide a window for the first time into early molecular pathways disrupting fetal lung angiogenesis and morphogenesis before preterm labor occurs, which may set the stage for BPD. A strategy to prevent BPD should target the fetus *in utero* to attenuate alterations in the fetal lung genetic program.

## Introduction

Intra-amniotic inflammation is thought to play a major role in the pathogenesis of fetal lung injury, aberrant lung development and the resulting neonatal and adult chronic lung disease. [1], [2] Bronchopulmonary dysplasia (BPD) accounts for the vast majority of chronic lung disease in infancy affecting 35% of infants weighing less than 1,500 grams. [3] Studies have linked elevated cytokines in the amniotic fluid with an increase in BPD and neonatal morbidity/mortality. [1], [2], [4], [5] In surfactant-treated patients, BPD is characterized histologically by some degree of alveolar septal fibrosis, arrest in acinar development, and impaired vascular development. [6], [7] Current therapies in the postnatal period are only minimally effective for BPD prevention [8], [9] and the mechanisms initiating and propagating lung injury *in utero* remain ill-defined and difficult to study in humans because of confounding clinical variables in the care of preterm infants.

The underlying pathogenesis of BPD is thought to be due to disruption of normal growth and vasculogenesis in the saccular stage of lung development (24–38 weeks gestation), resulting in alveolar simplification from a lack of secondary alveolar septation. [10] Despite surfactant therapy and newer modes of mechanical ventilation, the prevalence of BPD has increased, particularly in very immature infants who may have little or no evidence of respiratory distress syndrome after birth. [11], [12] Neonatal lung samples to study the early pathologic changes associated with BPD are extremely limited and tend to exist mainly in end-stage BPD leaving a primary role for animal models to explain the mechanisms for alveolar simplification. Possible factors include disrupted signaling between lung mesenchyme derived growth factors and distal airspace epithelium as well as disrupted endothelial–epithelial cross-talk that interferes with normal alveolar and vascular morphogenesis. [7], [10] A critical precursor to BPD may be fetal exposure to cytokines in the amniotic fluid inducing lung injury in utero, which evolves into chronic lung injury following preterm delivery and exposure to mechanical ventilation and hyperoxia. [1], [2], [4], [5], [13], [14].

Prior studies in animal models have used ventilation after preterm delivery (sheep, baboon) or inoculation of lipopolysaccharide during pregnancy to mimic chorioamnionitis and intrauterine infection (sheep), which have produced histologic features consistent with BPD. [15], [16] A comprehensive genomic analysis of the lung injury has not been done in these models or is not yet possible with commercial microarray platforms (e.g. sheep). These models also differ slightly from humans in terms of lung developmental stage at the time of insult, which is a possible limitation in their application to the human neonate. Most neonates who develop BPD are born during the saccular period of lung development, which spans 24 to 38 weeks gestation and reflects a critical period of morphogenesis and angiogenesis. Clusters of thin-walled saccules begin to form giving rise to the alveolar ducts. Epithelial type 2 cells and the number of small vessels increase and capillaries begin to reorganize to form an air-blood interface. [17] In contrast, the preterm ventilated baboon model was created in the late canalicular stage of lung development (67% of term gestation), which precedes the saccular stage and involves formation of the terminal bronchioles. [15] The saccular stage of lung development is not recognized in the sheep with development progressing directly to the alveolar stage much earlier in utero. [18] Although the histopathologic features of BPD have been described in animal models and humans, there is limited understanding of the molecular basis of impaired lung alveolarization and vascular development in the saccular stage of lung development and the relative contribution of intrauterine inflammation to the process.

To investigate early factors involved in the initiation of intrauterine inflammation and fetal lung injury, we used a chronically catheterized pregnant nonhuman primate model (pigtail macaque; *Macaca nemestrina*) that shares many important features with human pregnancy. [19] We infused Group B *Streptococcus*, an organism known to cause preterm birth and neonatal invasive disease, [20], [21] into the choriodecidual space via a catheter placed between the uterine muscle and membranes (external to amniotic fluid) overlying the lower uterine segment. We performed Cesarean section four days after choriodecidual inoculation to capture early biological events associated with intrauterine infection. Bacteria did not translocate into the amniotic fluid, but did cause a cytokine-mediated pro-inflammatory response associated with fetal lung injury and in some cases, preterm labor. In this article, using microarray gene expression profiling, we describe for the first time molecular pathways that are activated and disrupted in the fetal lung during the saccular stage development associated with inflammation induced by a limited GBS choriodecidual infection.

## Results

### Cytokines, Placental and Fetal Lung Pathology and Immunohistochemistry

As we previously reported, significant elevations of AF cytokines (TNF-α, IL-8, IL-1β, IL-6) were detected in GBS versus controls (p<0.05). [22] Lung injury was not directly caused by GBS, because GBS was undetectable by culture and PCR in the AF and fetal lungs (Table 1). Fetal plasma IL-8 was significantly higher in GBS animals versus controls and of the fetal cytokines measured it correlated best with fetal lung injury (p = 0.03).

**Table 1:** Correlation between fetal lung score, amniotic fluid and fetal cytokines, culture results and labor.

Group	Fetal Lung Score	Peak Amniotic Fluid		Peak Fetal Plasma		InoculationSite Culture	Chorio-amnionitis	Labor	Amniotic Fluid	
		IL-6(ng/ml)	IL-8(ng/ml)	IL-6(pg/ml)	IL-8(pg/ml)				Culture*	PCR* for GBS
GBS 1	4	77.9	19.3	11.3	563.5	100 CFU GBS	Yes	Yes	No Growth	Negative
GBS 2	2	102.6	6.3	7.5	558.1	No Growth	Yes	Yes	No Growth	Negative
GBS 3	3	88.1	27.2	3.1	1,975.2	1,000 CFU GBS	No	No	No Growth	Negative
GBS 4	0	13.8	3.6	0	3,142.8	No Growth	No	No	No Growth	Negative
GBS 5	3	80.8	8.5	2.6	1,606.5	No Growth	No	No	No Growth	Negative
Saline 1	0	8.3	1.0	**	**	No Growth	No	No	No Growth	Negative
Saline 2	2	16.0	1.6	2.0	523.4	No Growth	No	No	No Growth	Negative
Saline 3	1	3.0	0.5	**	**	No Growth	No	No	No Growth	Negative
Saline 4	0	10.3	1.6	0.9	182.3	No Growth	No	No	No Growth	Negative
Saline 5	0	11.7	1.9	2.4	223.0	No Growth	No	No	No Growth	Negative

* Serial cultures of amniotic fluid were tested during the course of the experiment with approximately 10 samples tested per animal. Quantitative real-time PCR was performed on serially sampled amniotic fluid at three points in the course of the experiment including the day prior to inoculation, day of Cesarean section and once during the course of the experiment.

Histopathological examination of placenta, cord and fetal membranes revealed chorioamnionitis (neutrophils in amnion and chorion) in 2/5 GBS cases and 0/5 controls (Table 1). In one of the two affected GBS placentas, active inflammation was restricted to the inoculation site. In the other, inflammation was more widely disseminated in the membranes and fetal surface of the placental disc, and was accompanied by funisitis. Neither active nor chronic (lymphohistiocytic) inflammation was observed in any of the other samples, including some GBS cases with elevated cytokine levels.

**Figure 1:**
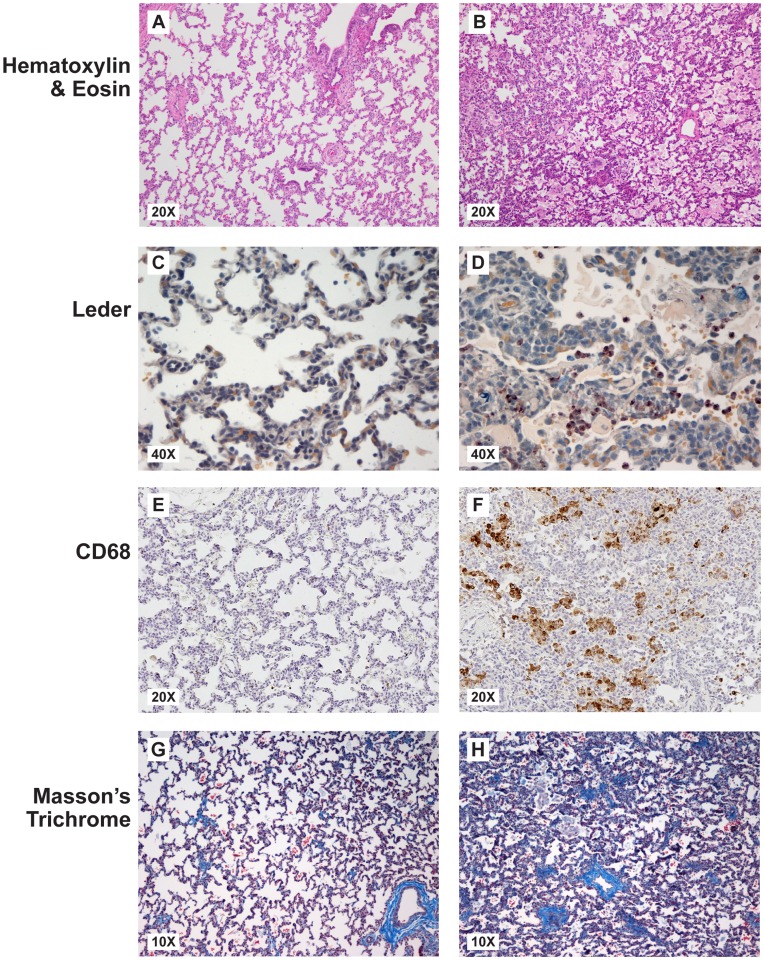
Histopathology of the fetal lungs are shown on the left for a saline control with lung score of 0 (A, C, E, G) and on the right for a GBS animal with severe fetal lung injury and lung score of 4 (B, D, F, H). Panels show hematoxylin and eosin staining (A, B), Leder staining for neutrophils (C, D), CD68 staining for macrophages (E, F), and Masson’s Trichrome staining for collagen (G, H).

While the primary focus of this study was a genomics analysis we performed a limited correlative histologic evaluation of lung to aid in confirming these findings. Representative fetal lung sections from a GBS and saline control animal are shown in Figure 1. Lung injury was defined as an aggregate of histologic changes involving all sections from each animal including accumulation of inflammatory cells, evidence of necrosis, inflammatory related tissue thickening, collapse or other injury such as fibrin exudation or hemorrhage. There was evidence of fetal lung injury in four of the five GBS animals (lung scores = 2, 3, 3, and 4), as well as one control (lung score = 2). Inflammatory cells observed in the fetal lungs from GBS group included high numbers of neutrophils and macrophages, which is consistent with the pattern described in preterm infants at different stages of developing BPD. [23] In addition, there was increased staining density and thickened septa that was absent in saline controls. A lung score of 0 to 1 was considered within normal limits because mild alveolar thickening can be normal in preterm animals and a few scattered neutrophils are also expected following saline inoculation or lavage. [24], [25], [26].

The distribution of injury varied with severity and involved vascular, perivascular, airway and alveolar compartments. The most severe cases of fetal lung injury (fetal lung scores  = 3, 3, and 4) correlated with the highest levels of amniotic fluid and fetal interleukin-8 (IL-8) levels, but not with other cytokines or prostaglandins tested (Table 1). The GBS animal with the greatest degree of fetal lung injury (lung score  = 4) also developed preterm labor and had a fetal interleukin-6 (IL-6) level of 11.3 pg/ml, which is diagnostic of the fetal systemic inflammatory response syndrome (FIRS) in humans. [27] In the other three GBS animals with fetal lung injury, the fetal IL-6 level (2.6, 3.1, 7.5 pg/ml) was below the threshold for FIRS. Fetal plasma IL-1β was undetectable in all but one animal and fetal plasma tumor necrosis factor-alpha (TNF-α) was undetectable in all but two animals. In one control animal with an elevated fetal lung injury score (lung score = 2), there was an infarction of a lung tip that appeared histologically very different from the controls and GBS lungs. In this case, hemorrhage was the predominant finding and thought to have occurred peri-mortem.

### Single Gene Analysis

After choriodecidual GBS exposure, there was differential expression of 707 out of 52,865 probesets (428 up- and 279 downregulated) in the fetal lung at least 1.5 fold (p<0.05). When probesets were matched to genes and duplicates removed, there was differential expression of 335 out of 19,571 genes (232 up- and 103 downregulated). A subset of these genes is shown in Table 2 and the entire set (707 probesets) and a heatmap (335 genes) is available in supplementary material (Table S1, Figure S1). Examples of genes significantly upregulated included indoleamine 2,3-dioxygenase 1 (IDO1), serpin peptidase inhibitor clade A member 3 (SERP3; also called alpha-1 antiproteinase, antitrypsin), chemokine (C-C motif) ligand 3 (CCL3), matrix metalloproteinase 1 (MMP1), IL-1β, and IL-8. Genes significantly downregulated by choriodecidual GBS exposure included similar to aldo-keto reductase family 1 member B10 (AKR1B10), which is implicated in the process of lung septation (see discussion) and adenylosuccinate synthase (ADSS). The magnitude of change ranged from 3.0 log_2_ fold for upregulated and 3.5 log_2_ fold for downregulated genes.

**Table 2:** Examples of most differentially regulated single genes.

ProbeSetID	Rhesus Ensembl ID	log2 fold change	RhesusEntrez gene	Description	Symbol
MmuSTS.4444.1.S1_at	–	−1.95	722683	similar to Kallikrein-7 precursor (hK7) (Stratum corneum chymotryptic enzyme) (hSCCE)	LOC722683
Mmu.8081.1.S1_at	ENSMMUG00000012324///ENSMMUG00000013098	−1.90	706406///713150	similar to aldo-keto reductase family 1, member B10///similarto aldo-keto reductase family 1, member B10	LOC706406///LOC713150
Mmu.8081.1.S1_x_at	ENSMMUG00000012324	−1.40	713150	similar to aldo-keto reductase family 1, member B10	LOC713150
MmugDNA.36075.1.S1_at	ENSMMUG00000018680	−1.15	702126	potassium voltage-gatedchannel, shaker-relatedsubfamily, member 2	KCNA2
Mmu.4846.2.S1_at	ENSMMUG00000000016	−1.09	713580	adenylosuccinate synthase	ADSS
MmugDNA.14045.1.S1_at	ENSMMUG00000005319	−1.05	712581	advillin	AVIL
MmuSTS.2685.1.S1_s_at	–-	2.08	702712	similar to G protein-coupled receptor 109A	LOC702712
Mmu.11363.1.S1_at	ENSMMUG00000020903	2.10	574243	chemokine (C-X-C motif) ligand 10	CXCL10
MmugDNA.5658.1.S1_at	ENSMMUG00000003364	2.27	712571	BCL2-related protein A1	BCL2A1
MmuSTS.652.1.S1_at	ENSMMUG00000019778	2.28	704701	interleukin 1, beta	IL1B
MmuSTS.921.1.S1_at	ENSMMUG00000002037	2.34	703653	matrix metallopeptidase 1 (interstitial collagenase)	MMP1
MmuSTS.2150.1.S1_at	ENSMMUG00000011218	2.38	574106	serpin peptidase inhibitor,clade A (alpha-1 antiproteinase, antitrypsin), member 3	SERPINA3
Mmu.2053.1.S1_s_at	ENSMMUG00000030216	2.39	619514	chemokine (C-C motif) ligand 3	CCL3
MmugDNA.18432.1.S1_at	ENSMMUG00000019304	2.96	574370	indoleamine 2,3-dioxygenase 1	IDO1

There are eight surfactant protein probesets on the Affymetrix Rhesus Macaque Array, but only 5 of the probesets were annotated in the single gene analysis by the Affymetrix software. When the IPA software was used to annotate the genes using their proprietary database, more surfactant proteins (SFTP) were identified. None of the eight probesets demonstrated significant differential regulation in the single gene analysis, but SFTPA1 and SFTPA2 were the most differentially expressed of the group (log_2_ fold 0.71 and 0.73, respectively).

### Gene Set Analysis (GSA)

**Figure 2:**
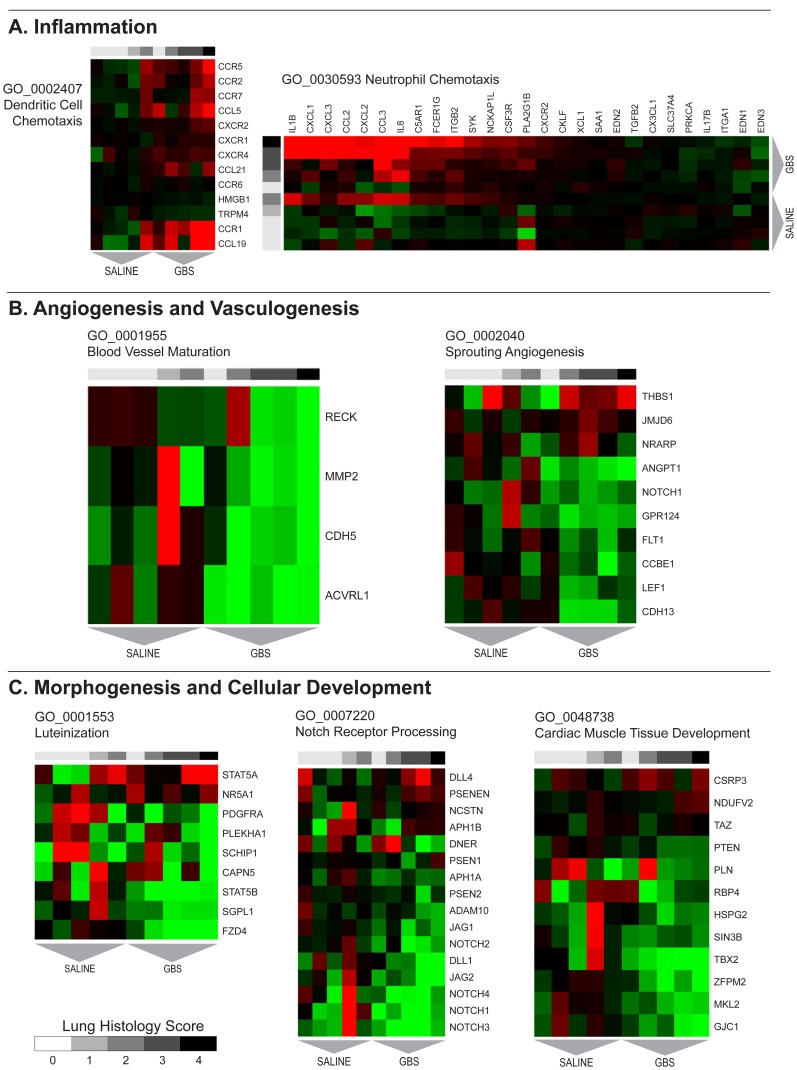
Heatmaps of Gene Ontology gene sets associated with A) inflammation, B) angiogenesis and vasculogenesis, and C) morphogenesis and cellular development pathways.

Gene sets and pathways with concordant changes in expression were identified using GSA. Gene sets enriched after GBS exposure are shown in Table 3 with a complete listing provided in supplementary material (Table S2). Heat maps of select gene sets associated with inflammation, angiogenesis, and cellular growth are shown in Figure 2. Upregulated gene sets in the GBS group were frequently related to activation of an innate and adaptive immune response including positive regulation of immune response, neutrophil chemotaxis, positive regulation of IL-8, regulation of T cell activation, positive regulation of adaptive immune response, dendritic cell chemotaxis, and antigen processing and presentation of exogenous peptide antigen via Major Histocompatibility Complex (MHC) class II. Other pathways upregulated in the GBS group included pyrimidine base metabolic process, leukotriene metabolic process, cell-cell signaling, pyrimidine nucleoside salvage, and negative regulation of nitric oxide synthase activity. Gene sets downregulated following GBS exposure were frequently related to morphogenesis, cellular growth and structure (e.g. cardiac muscle tissue development, luteinization, Notch receptor processing) and angiogenesis (e.g. sprouting angiogenesis, blood vessel maturation, vasculogenesis). These gene sets are shown in Table 4 with a full listing in supplementary material (Table S3).

**Table 3:** Gene sets upregulated after GBS exposure relative to the control group.

Gene set description	Gene set length	Gene Ontology ID	p value
**Biological process**			
pyrimidine base metabolic process	24	GO:0006206	<0.002
leukotriene metabolic process	11	GO:0006691	<0.002
cellular aromatic compound metabolic process	8	GO:0006725	<0.002
cell-cell signaling	252	GO:0007267	<0.002
phospholipid catabolic process	15	GO:0009395	<0.002
pyrimidine nucleoside salvage	9	GO:0043097	<0.002
positive regulation of innate immune response	15	GO:0045089	0.002
pyrimidine base metabolic process	24	GO:0006206	0
**Molecular function**			
neuropeptide hormone activity	27	GO:0005184	<0.002
hyaluronic acid binding	21	GO:0005540	<0.002
oxidoreductase activity	10	GO:0016712	<0.002
aromatase activity	21	GO:0070330	0.003
monooxygenase activity	73	GO:0004497	0.005
pancreatic ribonuclease activity	8	GO:0004522	0.006
organic anion transmembrane transporter activity	15	GO:0008514	0.006
**Cellular component**			
cytoplasmic part	20	GO:0044444	0.008
gap junction	26	GO:0005921	0.013
connexon complex	18	GO:0005922	0.018
vesicle	31	GO:0031982	0.018
dendritic spine membrane	5	GO:0032591	0.018
intrinsic to internal side of plasma membrane	11	GO:0031235	0.019
alpha-amino-3-hydroxy-5-methyl-4-isoxazolepropionic acid selective glutamate receptor complex	13	GO:0032281	0.019

**Table 4:** Gene sets downregulated after GBS exposure relative to the control group.

Gene set description	Gene set length	Gene Ontology ID	p value
**Biological process**			
positive regulation of receptor recycling	5	GO:0001921	0.008
blood vessel maturation	5	GO:0001955	0.008
chromatin assembly or disassembly	38	GO:0006333	0.008
carnitine shuttle	8	GO:0006853	0.008
small GTPase mediated signal transduction	301	GO:0007264	0.008
carnitine metabolic process	7	GO:0009437	0.008
peptidyl-histidine phosphorylation	5	GO:0018106	0.008
microtubule organizing center organization	5	GO:0031023	0.008
substrate adhesion-dependent cell spreading	19	GO:0034446	0.008
protein ubiquitination involved in ubiquitin-dependent protein catabolic process	45	GO:0042787	0.008
stress fiber assembly	8	GO:0043149	0.008
cardiac muscle tissue development	12	GO:0048738	0.008
**Molecular function**			
proline-rich region binding	16	GO:0070064	0.008
positive transcription elongation factor activity	7	GO:0008159	0.010
acid-amino acid ligase activity	79	GO:0016881	0.010
histone acetyltransferase binding	15	GO:0035035	0.011
growth factor binding	39	GO:0019838	0.012
SMAD binding	37	GO:0046332	0.012
phosphoprotein binding	37	GO:0051219	0.012
transmembrane receptor protein tyrosine kinase activity	54	GO:0004714	0.013
transforming growth factor beta receptor activity	18	GO:0005024	0.013
two-component sensor activity	14	GO:0000155	0.014
**Cellular component**			
clathrin adaptor complex	23	GO:0030131	0.009
axon part	15	GO:0033267	0.010
tight junction	83	GO:0005923	0.011
lamellipodium	99	GO:0030027	0.011
CUL4 RING ubiquitin ligase complex	11	GO:0080008	0.013
actin filament	39	GO:0005884	0.016
Axin-APC-beta-catenin-GSK3B complex	7	GO:0034747	0.017
Schmidt-Lanterman incisure	8	GO:0043220	0.017
AP-type membrane coat adaptor complex	6	GO:0030119	0.018
endomembrane system	91	GO:0012505	0.019

### Ingenuity Pathway Analysis (IPA)

**Figure 3:**
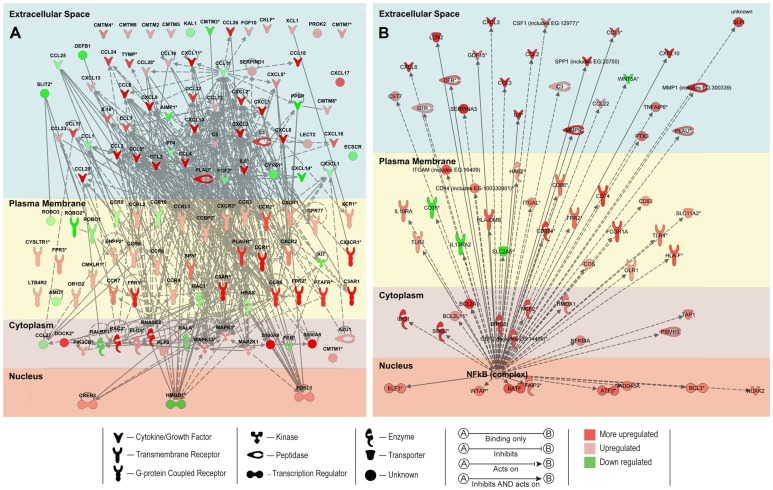
Comparison of the microarray and qRT-PCR analysis. The x-axis represents individual genes and the y-axis fold-change in expression by either microarray (gray bars) or RT-PCR (black bars). All genes shown were significant in the unadjusted microarray analysis. Genes that were significantly up- or downregulated by RT-PCR between GBS and controls are indicated by a star (two-sided t-test, p<0.05).

IPA mapped 36,617 probesets out of the 52,779 probesets on the Affymetrix *Macaca mulatta* microarray. The top five canonical pathways identified by IPA analysis were the antigen presentation pathway, dendritic cell maturation, triggering receptor expressed on myeloid cells 1 (TREM1) signaling, allograft rejection signaling, and communication between innate and adaptive immune cells (Table 5). IPA analysis also has the capability to predict activation states of transcriptional regulators based on the activation or suppression of downstream genes. The top five transcription factors predicted to be associated with the changes in genes expression were NF-kappa B (NF-κB), signal transducer and activator of transcription 3 (STAT3), STAT1, CCAAT/enhancer-binding protein alpha (CEBPA), and spleen focus forming virus (SFFV) proviral integration oncogene (SPI1); all were predicted to be in the activated state. IPA diagrams of the Gene Ontology gene set neutrophil chemotaxis and NF-κB are shown in Figure 3.

**Table 5:** Ingenuity Pathway Analysis Summary.

*Functional Analysis of a Network* *		
*Diseases and Disorders*	Number of Molecules	p value
Inflammatory response	169	1.7×10^−61^–3.77×10^−6^
Connective tissue disorders	144	7.39×10^−32^–1.16×10^−6^
Inflammatory disease	203	7.39×10^−32^–2.15×10^−6^
Skeletal and muscular disorders	144	7.39×10^−32^–1.16×10^−6^
Immunological disease	169	1.23×10^−29^–2.65×10^−6^
***Molecular and Cellular Functions***		
Cellular movement	137	7.27×10^−39^–3.57×10^−6^
Cell-to-cell signaling and interaction	138	1.06×10^−36^–3.77×10^−6^
Antigen presentation	88	3.91×10^−26^–3.77×10^−6^
Cellular growth and proliferation	187	4.31×10^−26^–2.29×10^−6^
Cell death	127	5.13×10^−20^–2.74×10^−6^
***Physiological Systems***		
Immune cell trafficking	126	7.27×10^−39^–3.77×10^−6^
Hematological system development	168	4.91×10^−36^–3.77×10^−6^
Tissue development	142	1.53×10^−26^–2.26×10^−6^
Tissue morphology	84	1.04×10^−24^–1.48×10^−6^
Organismal survival	51	1.33×10^−15^–1.27×10^−7^
***Canonical Pathway Analysis*** **		
**Top Canonical Pathways**	**Ratio**	**p value**
Antigen presentation pathway	13/43	3.93×10^−13^
Dendritic cell maturation	22/188	3.99×10^−12^
TREM1 signaling	14/66	1.45×10^−11^
Allograft rejection signaling	12/96	4.72×10^−11^
Communication between innate and adaptive immune cells	15/109	8.28×10^−11^
***Transcription Factor Analysis*** ***		
**Top Transcription Factors**	**Predicted Activation State**	**p-value of overlap**
NF-κB	Activated	4.80×10^−29^
STAT3	Activated	1.13×10^−22^
STAT1	Activated	2.70×10^−20^
CEBPA	Activated	4.31×10^−19^
SPI1	Activated	9.71×10^−17^

* The Functional Analysis of a Network identified biological functions and/or diseases that were most significant to the molecules in the network using a right-tailed Fisher’s exact test.

** Canonical Pathway Analysis identified pathways from the IPA library that were most significant to the data set. Significance of the association was measured in two ways: (1) as the ratio of the number of molecules from the focus gene set that map to the pathway to the total number of molecules that map to the canonical pathway and (2) using Fisher’s exact test.

*** Transcription factor analysis is based on prior knowledge of expected effects between transcription factors and their target genes stored in the IPA library. The overlap p-value measures whether there is a statistically significant overlap between the dataset genes and the genes regulated by a transcription factor using Fisher’s Exact Test.

### Validation of cDNA Microarray by Quantitative RT-PCR

**Figure 4:**
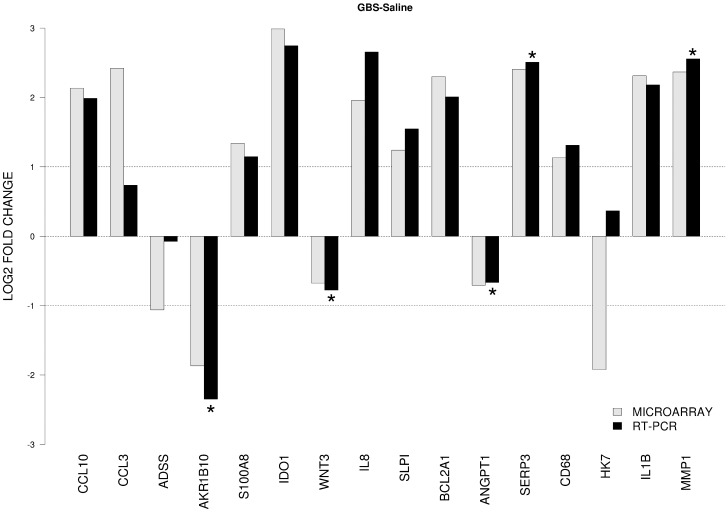
IPA diagrams were generated for A) neutrophil chemotaxis Gene Ontology gene set and B) NF-κB, a transcription factor predicted to be activated by IPA. Genes shown were filtered for interactions reported in humans only and presented by cellular localization. Direct and indirect interactions are shown by solid lines and dashed lines respectively. Green indicates gene downregulation and red depicts upregulation. White symbols indicate functionally associated neighboring genes that were not differentially expressed in the data. Color intensity represents the average of log2 fold change with brighter colors representing a more significant difference between GBS and controls. Symbols for each molecule are presented according to molecular functions and type of interactions.

We identified 16 genes of interest from the microarray dataset, which we analyzed by quantitative RT-PCR. We directly compared levels of gene expression obtained with amplified RNA samples using GAPDH expression as a control for input cDNA. Overall agreement between the mRNA generated microarray data and the quantitative RT-PCR data was approximately 94% with only one discordant gene out of 16 tested [human kallikrein 7 (HK7); Figure 4]. There was a significant difference in RT-PCR results between GBS and controls for AKR1B10, wingless-type MMTV integration site family member 3 (WNT3), angiopoietin 1 (ANGPT1), serpina 3 (SERP3), and MMP1 (all p<0.05).

## Discussion

Studying biological events that occur prior to birth is extraordinarily difficult for ethical reasons and also because lung development in other animal models does not necessarily emulate the human fetus. We have overcome these challenges and present the first comprehensive genomics study of cytokine-induced fetal lung injury in a nonhuman primate model that shares many key features with human pregnancy and lung development. [17] A key feature of this model is the amniotic fluid contained elevated cytokine levels without detectable bacteria suggesting that the fetal lung injury may occur silently through the action of inflammatory mediators many days before the development of preterm labor. [22] The current study expands our understanding of the gene pathways affected by *in utero* inflammation that may be precursor pathways important for the prevention of alveolar growth arrest and microvascular disruption present in infants that develop BPD.

**Figure 5:**
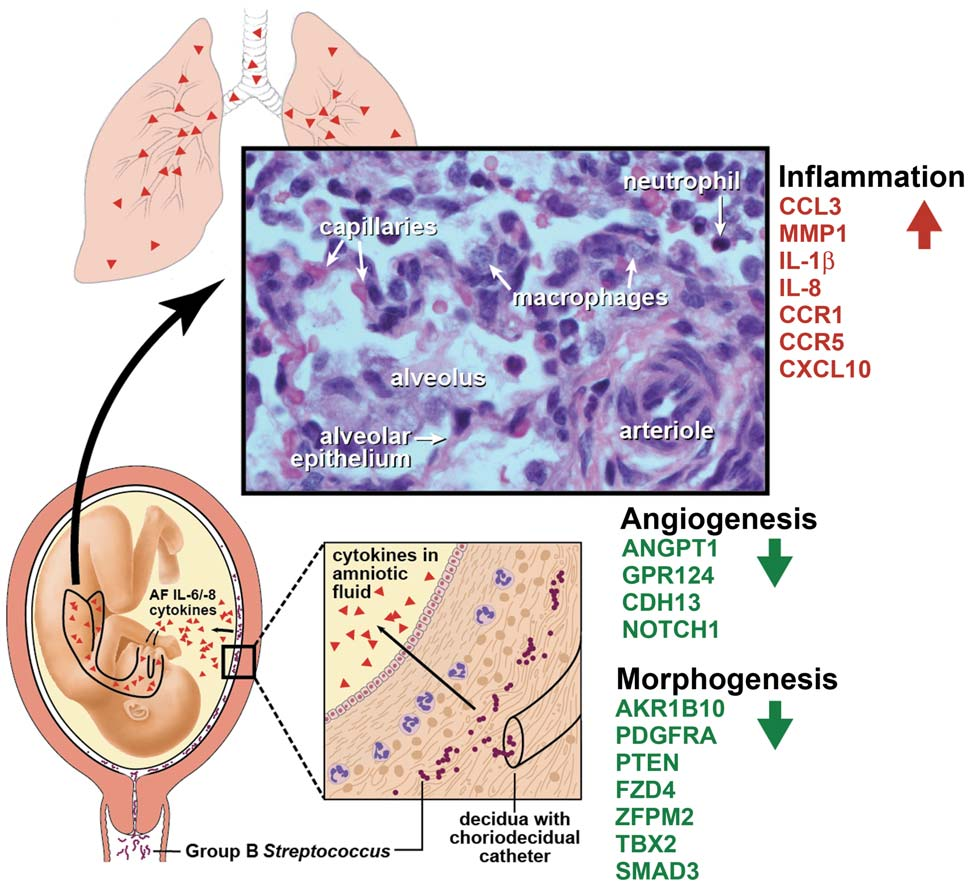
Our conceptual model of events leading to fetal lung injury *in utero*. First, bacteria from the vagina traffics into the choriodecidual space. Inflammation (e.g. IL-8) is produced by the decidua and/or membranes, which diffuses into the amniotic fluid and fetal lung. Fetal lung injury is induced by inflammatory mediators with significant genes shown involved in inflammation, cellular growth, and angiogenesis. IL, Interleukin; AF, amniotic fluid.

Our data suggest a conceptual model shown in Figure 5, which extends the original hypothesis of Yoon et al in 1997 that began to link amniotic fluid inflammation with fetal lung injury. [4] First, we hypothesize that vaginal bacteria traffic upwards through the cervix and into the choriodecidual space, which lies between the fetal membranes and the uterus (external to the amniotic sac). A pro-inflammatory cytokine response ensues, which may or may not trigger preterm labor depending on the severity. Cytokines and other inflammatory mediators produced in the choriodecidual space begin to diffuse into the AF. The fetal lungs are in direct contact with the amniotic fluid due to normal swallowing *in utero*, which has been reported as early as 11 weeks. [28] When pro-inflammatory cytokines from the AF come into contact with the fetal lungs, an innate immune response is initiated in the fetal lungs with subsequent recruitment of neutrophils and macrophages. [29], [30] Inflammation results with the degree likely related to the intensity, duration, and developmental timing of the cytokine exposure. Pulmonary gene expression related to the innate and adaptive immune response increases while expression related to angiogenesis, morphogenesis, and cellular development decreases. This inflammation may set the stage for further lung injury and BPD if preterm delivery occurs with subsequent lung injury by mechanical ventilation and hyperoxia.

Our data is consistent with many prior studies implicating a role for several inflammatory mediators (IL-1β, TNF-α, IL-6, IL-8) in BPD development, which were significantly elevated in the AF of our model. [4], [31], [32], [33], [34] Although not specific for BPD, elevated IL-6 and IL-8 precede neutrophil infiltration in tracheal aspirates from preterm infants who develop BPD. [32] Innate immune responses, neutrophil chemotaxis, regulation of IL-8, and leukotriene metabolism were associated with fetal lung injury and featured prominently in our GSA. Interestingly, elevated leukotriene levels in tracheal lavage fluid are thought to be related to the bronchospasm associated with BPD. [35] Consistent with our model, increased neutrophils and macrophages in the pulmonary effluent of neonates with BPD may occur even in the absence of bacterial colonization. [36], [37] The transcriptional activators, NF-κB and STAT3, were predicted to be activated by IPA and are known to play a role in the innate immune response. Downstream genes activated by NF-κB that are associated with BPD development include IL-8 and MMP-1 and MMP-9. STAT3 has been previously implicated in fetal lung injury in the setting of chorioamnionitis and is identified as a potential target for regulating the pulmonary inflammatory response. [38], [39] In addition to innate immune responses, the adaptive immune response gene sets (e.g. dendritic cell chemotaxis, regulation of T cell activation) and genes involved in dendritic cell activation (e.g. CCR7) were significantly upregulated in the analysis. These results are consistent with findings of pulmonary recruitment of dendritic cells in human exposed to antenatal infection and ventilation who develop BPD. [40] Dendritic cells, which express a wide array of pro- and anti-angiogenic mediators, are closely associated with the pulmonary microvasculature and may contribute to BPD-associated dysangiogenesis. [40], [41].

Many of the downregulated genes or gene sets (i.e. Gene Ontology categories associated with specific biological processes) have putative or known roles in lung development, growth and structural integrity. AKR1B10 catalyzes the essential first step in the retinoic acid synthesis pathway, which increases lung septation (−1.9 log_2_ fold). [42], [43], [44] AKR1B10 is also important for cell survival and when silenced by small-interfering RNA resulted in elevated intracellular lipid peroxides and caspase-3-mediated apoptosis. [45] GSA identified many of the following downregulated genes in gene pathways for luteinization and cardiac muscle tissue development that are known to be involved in lung morphogenesis. Both Wnt5a and its receptor Frizzled 4 (FZD4) play an important role in morphogenesis of the distal lung. [46], [47] Deficiency of SMAD3, a major signal transducer in the transforming growth factor-beta (TGF-β) pathway, impairs neonatal lung alveolarization and peripheral lung cell proliferation based in murine studies. [48] Platelet derived growth factor receptor, alpha polypeptide (PDGFRA) is expressed by bronchiolar smooth muscle progenitors and might impair mesodermal development if downregulated. [49] T-box (TBX) transcription factors, such as TBX2 in the cardiac muscle tissue development pathway, have been implicated in developing lung mesoderm. [50] Finally, phosphatase and tensin homolog (PTEN) has been shown to be essential for normal lung morphogenesis and when deleted in mice resulted in impaired branching morphogenesis and distal alveolar epithelial cell differentiation. [51].

Other downregulated genes and gene sets were associated with angiogenesis and vascular dysfunction. ANGPT1 is the primary agonist of the tyrosine kinase receptor Tie 2 (tyrosine kinase with immunoglobulin and EGF-like domains), which is restricted to endothelial cell expression. [52], [53], [54] Cord blood plasma levels of ANGPT1 in preterm infants who subsequently develop BPD are significantly lower than those without BPD. [55] G protein-coupled receptor 124 (GPR124) is thought to play a role in regulating sprouting, migration, and developmental expression of the blood-brain barrier and is also expressed in embryonic epithelium of lung. [56] Cadherin 13 (CDH13), an atypical glycosylphosphatidylinositol (GPI)-anchored member of the cadherin superfamily widely expressed in the cardiovascular system, is involved in tumor angiogenesis and promotes proliferation in vascular cells and angiogenesis via activation of the PI3K/Akt/mTOR signaling pathway when overexpressed and ligated. [57], [58], [59], [60] Notch signaling was downregulated in the GSA, which is thought to play a primary role in selection of Clara cell fate and arterial vascular smooth muscle cell recruitment [61], [62], [63]; Notch signaling also negatively regulates vascular endothelial growth factor (VEGF)-induced angiogenesis and is thought to suppress aberrant vascular branching morphogenesis. [64] Our data also demonstrated a significant downregulation of genes in the GBS group associated with regulation of nitric oxide synthase (NOS). In the preterm lamb lung, antenatal exposure to intra-amniotic endotoxin is associated with decreased postnatal endothelial NOS (eNOS) expression measured at 2–4 days of age followed by vascular remodeling changes in small pulmonary arteries demonstrated by medial smooth muscle hypertrophy and increased adventitial fibrosis. [65].

The strength of our study lies in the similarities in lung development and immune function between the nonhuman primate and human neonate. Pulmonary morphologic and immune features in our model also emulate that in humans, but differ from many other species. [66] Both humans and nonhuman primates lack pulmonary intravascular macrophages present in the lungs of many species (e.g. sheep, cattle, pigs) which tend to concentrate toxins and bacteria in the lungs. In contrast, humans and nonhuman primates localize bacteria and toxins in the liver and spleen, which makes their lungs less susceptible to injury than other species. [65], [67] There are also many similarities to human pregnancy including a singleton fetus with a long gestational period, hemomonochorial placentation, and sensitivity to pathogens (e.g. lipopolysaccharide). Maternal-fetal inflammatory responses induced by infection and parturition also emulate that in humans, but differ significantly in other animal models. Many mammalian models in which lung development has been studied (e.g. sheep) are also in the alveolar stage of lung development, which is more advanced at the time of a preterm birth than in human preterm neonates (saccular) who go on to develop BPD. The elevated AF cytokine levels despite negative AF culture results seen in our model also appears to replicate the clinical condition in humans seen in up to 25% of preterm labor cases with elevated amniotic fluid IL-6 and a negative culture and/or PCR. [68], [69], [70] Therefore, our model may reflect a common biological event of pro-inflammatory cytokine signaling induced by many different pathogens in the choriodecidual space and is not restricted specifically to GBS.

A limitation of our study is the lack of lung morphometry, but the time course from infection to delivery was fairly short (4 days) and differences may not yet have become apparent. Lung injury in this model was originally unexpected and the fetal lung was not preserved in such a way to accurately measure lung morphometry later. Another limitation, as well as study strength, is that our results reflect an early or limited choriodecidual infection. We may have interrupted pathways leading to further fetal lung injury, maturation, or possibly repair with further time *in utero*. The acute histologic changes seen in the GBS exposed animals lungs, including accumulation of inflammatory cells, interstitial wall tissue thickening, and fibrin exudation or hemorrhage, may be precursor findings that subsequently develop into the histopathologic findings of alveolar simplification and enlargement characteristic of the new BPD after premature infants are exposed to mechanical ventilation, hyperoxia, and/or sepsis. [71] The risk for developing BPD in association with histologic chorioamnionitis increases in infants who are exposed to mechanical ventilation postnatally. [72] Since our model focused on early *in utero* time points, it is not known if the histopathologic findings in the GBS exposed animals would eventually develop BPD-like features if exposed to other postnatal insults associated with ventilation; however, our gene expression findings suggest this possibility. Therefore, future studies in our model are needed to determine if the fetal lung injury associated with elevated AF pro-inflammatory cytokines requires subsequent postnatal injury (e.g., mechanical ventilation or sepsis) for BPD to develop. The timing, degree, and duration of exposures and events producing lung injury *in utero* and *ex utero* may influence the ultimate pulmonary phenotype of preterm infants at risk for BPD. Our sample size is also modest, which is typical of nonhuman primate studies and necessary for ethical reasons and conservation. Finally, not all the probe sets on the Affymetrix chip are annotated and some differentially regulated genes could not be identified.

The microarray analysis reported in this study measured expression of thousands of genes in a limited number of biological replicates. A concern with this experimental design is a Type I (“false positive”) or Type II (“false negative”) error when assessing the statistical significance of expression changes of single genes. We minimized this concern with the following strategies. First, the samples that were used for microarray analysis were carefully phenotyped, i.e. lung injury was defined as an aggregate of histologic changes including accumulation of inflammatory cells, evidence of necrosis, inflammatory related tissue thickening, collapse or other injury such as fibrin exudation or hemorrhage. We also used a 1.5-fold expression change criteria in addition to a p-value cut-off (p<0.05) to define differential gene expression. By using a combination of a fold-change and p-value criteria, Type I and II errors are decreased. In addition to carrying out single gene analysis, we also carried out Gene Set Analysis and Ingenuity Pathway Analysis. Both of these methods assess the statistical significance of pre-defined sets of genes/pathways as a whole, rather than single genes. Therefore, these methods are less prone to Type I or II errors derived from single genes. Finally, we validated a subset of genes that microarray analysis identified as differentially expressed with quantitative RT-PCR analysis and found a 94% agreement between these two independent methods. Given that it is cost-prohibitive to increase the number of animals (biological replicates) in this study, the aforementioned approaches minimize Type I and II errors as much as it is practically possible.

Our work confirms many of the pathways reported in later stages of lung development from ventilated preterm neonates [4], [40], [73], [74] and alveolar stage models of fetal lung injury. [47], [65], [75], [76] Our study extends this work with the first comprehensive genomic analysis of early *in utero* lung injury in the saccular stage of lung development. Molecular pathways reveal a significant disruption in angiogenesis and morphogenesis with upregulation of both innate and adaptive immune pathways. The finding that significant fetal lung injury occurred silently before preterm labor is also novel and quite sobering for the development of preventive strategies. Our data suggest that molecular pathways leading to BPD may originate *in utero* and precede delivery by several days or longer before a clinically recognizable event like preterm labor. Future studies to analyze changes in the fetal lung proteome and microRNAs will be important to complement this work in understanding the pathogenesis of *in utero* fetal lung injury. The nonhuman primate may be uniquely suited for the future study of therapies during pregnancy to limit *in utero* lung injury and prevent the resulting aberrant lung development.

## Materials and Methods

### Ethics Statement

This study was carried out in strict accordance with the recommendations in the Guide for the Care and Use of Laboratory Animals of the National Research Council and the Weatherall report, “The use of non-human primates in research”. The protocol was approved by the Institutional Animal Care Use Committee of the University of Washington (Permit Number: 4165-01). All surgery was performed under general anesthesia and all efforts were made to minimize suffering.

### Animals and Study Groups

Ten chronically catheterized pregnant monkeys (*Macaca nemestrina*) at 118–125 days gestation (term = 172 days) received one of two experimental treatments: 1) choriodecidual and intra-amniotic saline infusions (n = 5), or 2) GBS choriodecidual inoculation (n = 5). In two saline controls, fetal samples were not collected due to either an inability to place the fetal catheter during initial surgery or clotting of the fetal catheter. This resulted in three fetal cytokine analyses in the saline group. In one GBS case, technical problems led to only intermittent data collection and so the remaining uterine activity data was excluded for this animal. The cytokine analyses were previously published and are presented to give context to the genomics analyses. [77].

In our model, pregnant pigtail macaques were time-mated and fetal age determined using early ultrasound. Temperature in the animal quarters was maintained at 72–82 degrees Fahrenheit. Animals were fed a commercial monkey chow, supplemented daily with fruits and vegetables and drinking water was available at all times. The tethered chronic catheter preparation was used for all *in vivo* experiments and is a major breakthrough in studying maternal-fetal immunologic responses. [78], [79] The animal was first conditioned to a nylon jacket/tether system for several weeks before surgery, which allowed free movement within the cage, but protected the catheters. On day 118–125 of pregnancy (term = 172 days) catheters were surgically implanted via laparotomy into the maternal femoral artery and vein, fetal internal jugular vein, amniotic cavity, and choriodecidual interface in the lower uterine segment (between uterine muscle and fetal membranes, external to amniotic fluid). Fetal ECG electrodes and a maternal temperature probe were also implanted. Post-operative analgesia was provided by a 25 microgram fentanyl patch applied the day prior to surgery, in addition to postoperative indomethacin. After 48 hours, the animals appeared to have recovered from surgery based on a return to baseline for activity, appetite, and bowel function.

After surgery, the animal was placed in the jacket and tether with the catheters/electrodes tracked through the tether system. Cefazolin and terbutaline sulfate were administered to reduce postoperative infection risk and uterine activity. Both cefazolin and terbutaline were stopped at least 72 hours before experimental start (∼13 half-lives for terbutaline, 40 half-lives for cefazolin, >97% of both drugs eliminated), which represented approximately a 7–10 day period of postoperative terbutaline administration. Cefazolin 1 gram was administered intravenously each day in saline controls to minimize chances of a catheter-related infection. Experiments began approximately two weeks after catheterization surgery to allow recovery (∼30–31 weeks human gestation). At our center, term gestation in the non-instrumented pigtail macaque population averages 172 days.

### Pathology and Lung Injury

After cesarean section, fetuses were euthanized by barbiturate overdose followed by exsanguination and fetal necropsy with tissue fixation in 10% neutral buffered formalin. Complete gross and histopathologic examination was performed on infants and placentas. For histologic examination two to three randomly selected fixed fetal lung tissues were embedded in paraffin and sections stained with hematoxylin and eosin (H&E), Masson’s trichrome or specific esterase (Leder stain) using standard protocols. Leder staining was performed using the Naphthol AS-D Chloroacetate Specific Esterase Kit (Sigma-Aldrich, St. Louis, MO) per manufacturer’s instructions. Masson’s trichrome staining was performed to differentially highlight the presence of connective tissue by a standard method involving serial incubations in Bouin’s fixative, Weigert’s iron hematoxylin, Biebrich scarlet-acid fuchsin, phosphomolybdic-phosphotungstic acid and aniline blue. The placenta was examined by a board-certified pediatric pathologist (R.P.K.) and fetal lungs examined by a board-certified veterinary pathologist (H.D.L.) with each pathologist blinded to group assignment. H&E-stained full-thickness sections of placental disc, umbilical cord, and a fetal membrane roll were examined from each case to exclude inflammation, necrosis, fetal vascular thrombosis, or other histopathological findings. Chorioamnionitis was diagnosed when neutrophils were identified in the chorion and/or amnion. Funisitis denoted neutrophils in the umbilical vessels and/or surrounding connective tissue. Lung histologic sections were evaluated and scored, as previously described, using a semi-quantitative scale. [80] Components were scored on a scale of 0–4 (0 = normal) for inflammatory cells, necrosis, and inflammation including tissue thickening, collapse or other injury (e.g. fibrin exudation). Lung compartments scored were (1) vascular/perivascular; (2) bronchial/peribronchial; (3) alveolar wall; and (4) trichrome stain intensity positivity. Mononuclear inflammatory cells and neutrophils (Leder stain) within alveolar spaces were counted (5 random 40X fields). An overall severity score was generated. A lung score of 0 to 1 was considered within normal limits as a few neutrophils are expected following saline inoculation and mild alveolar thickening is normal in some preterm animals.

### Immunohistochemistry of Fetal Lung Tissues

Immunohistochemistry staining for CD68 was performed using a mouse monoclonal CD68 primary antibody (1∶1,000 dilution, Clone: KP1, MS-397-P1 (Thermo Fisher Scientific, Waltham, MA) a normal mouse IgG isotype control (1∶200 dilution, Vector Labs, I-2000), and a spleen control from *M. nemestrina*. First, the slides were baked for 30 minutes at 60°C and deparaffinized on the Leica Bond Automated Immunostainer (Leica Microsystems, Buffalo Grove, IL). Antigen retrieval was performed by placing slides in HIER Citrate Buffer for 10 minutes at 100°C. Blocking consisted of Leica Bond Peroxide block for 5 minutes at room temperature (RT) and then 10% Normal Goat Serum in PBS for 10 minutes at RT. Either the primary antibody (mouse anti-CD68, 1∶1,000 dilution, 0.2 µg/mL) or mouse isotype IgG control (1∶200 dilution, 1 µg/mL) in Leica Primary antibody diluent was applied for 30 minutes at RT. Leica Bond post primary was then applied for 8 minutes at RT. Antibody complexes were visualized using Leica Bond Polymer DAB Refine for 8 minutes at RT and then Leica Bond Mixed Refine (DAB) detection 2X for 10 minutes at RT. Tissues were counterstained with hematoxylin counterstain for 10 second followed by two rinses in H_2_0. Unless otherwise specified all reagents were obtained from Leica Microsystems.

### RNA Extraction and Microarray Processing

To study genetic pathways in *M. nemestrina*, we used the Affymetrix Rhesus Macaque Array (GeneChip® Rhesus Macaque Genome Array, Affymetrix, Santa Clara, CA), which allows interrogation of 47,000 *M. mulatta* transcripts and provides comprehensive transcriptome coverage. Genetic differences between *M. mulatta* and *M. nemestrina* are predicted to be <1%, which is consistent with our published data. [81] RNA extraction was performed by the CHDD Genomics Core Laboratory followed by the manufacturer’s protocols using the GeneChip platform by Affymetrix. Briefly, these methods include the synthesis of first- and second-strand cDNAs, the purification of double-stranded cDNA, the synthesis of cRNA by in vitro transcription (IVT), the recovery and quantitation of biotin-labeled cRNA, the fragmentation of this cRNA and subsequent hybridization to the microarray slide, the post-hybridization washings, and the detection of the hybridized cRNAs using a streptavidin-coupled fluorescent dye. Hybridized Affymetrix arrays were scanned with an Affymetrix GeneChip® 3000 fluorescent scanner. Image generation and feature extraction was performed using Affymetrix GeneChip Command Console Software.

### Single Gene Analysis

The data discussed in this publication have been deposited in NCBI’s Gene Expression Omnibus (Edgar *et al*., 2002) and are accessible through GEO Series accession number GSE39029 (http://www.ncbi.nlm.nih.gov/geo/query/acc.cgi?acc=GSE39029). Analysis of the microarray data focused first on differential expression of single genes. Raw microarray data was pre-processed and analyzed with Bioconductor (http://www.bioconductor.org/). [82] Several quality control steps were carried out to insure that the data was of high quality: 1) visual inspection of the GCOS DAT chip images, 2) visual inspection of the chip pseudo-images generated by the Bioconductor affyPLM package, 3) generation of percent present calls and average background signals with the Bioconductor simpleaffy package, 4) generation and inspection histograms of raw signal intensities, and 5) generation and comparison of the Relative Log Expression and Normalized Unscaled Standard Errors using the Bioconductor affyPLM package. The data was normalized with the Bioconductor GeneChip Robust Multiarray Averaging (RMA) package. [83] From the normalized data, genes with significant evidence for differential expression were identified using the Limma package in Bioconductor. [84] P-values were calculated with a modified t-test in conjunction with an empirical Bayes method to moderate the standard errors of the estimated log-fold changes. P-values were adjusted for multiplicity with the Bioconductor package qvalue, which allows for selecting statistically significant genes while controlling the estimated false discovery rate. [85].

### Gene Set Analysis (GSA)

Next, the data was analyzed using GSA in order to investigate categories of genes. [86], [87] GSA assesses the statistical significance of pre-defined gene sets/pathways as a whole rather than of single genes, which allows for the identification of modest but concordant changes in expression of individual genes that may be missed by single gene analysis. GSA software is available as R code (http://www.broad.mit.edu/GSA/). [86], [88] GSA considers all the genes in the experiment and allows for the identification of gene sets with strong cross-correlation by boosting the signal-to-noise ratio, which makes it possible to detect modest changes in gene expression. In GSA, the p-values that are calculated to test the null hypothesis are based on permutations of the sample labels. We used four gene set databases for the GSA: three from Gene Ontology [89] (Biological Process, Molecular Function, and Cellular Component), and the functional C2 gene set from the Molecular Signature Database [88] (http://www.broad.mit.edu/GSA/msigdb/msigdb_index.html).

### IPA Analysis

We used the Ingenuity Pathway Analysis (IPA) software (Ingenuity® Systems, www.ingenuity.com) to discover pathways and transcriptional networks in the gene expression microarray data. Our data set containing gene identifiers and corresponding expression changes between the experimental groups and p-values was uploaded into the IPA application. Each identifier was mapped to its corresponding object in the Ingenuity® Knowledge Base. The Functional Analysis identified the biological functions and/or diseases that were most significant to the data set. Genes from the data set with more than 1.5-fold differential expression (up/down regulation) and p<0.05 that were associated with biological functions and/or diseases in the Ingenuity Knowledge Base were considered for the analysis. The categories “Top Canonical Pathways” and “Top Transcription Factors” were primarily used in this analysis. Right-tailed Fisher’s exact test was used to calculate a p-value determining the probability that each biological function and/or disease assigned to that data set is due to chance alone. The IPA Path Designer Graphical Representation was used to generate figures. Molecules are represented as nodes, and the biological relationship between two nodes is represented as an edge (line). All edges are supported by at least one reference from the literature, from a textbook, or from canonical information stored in the Ingenuity Knowledge Base. Human, mouse, and rat orthologs of a gene are stored as separate objects in the Ingenuity Knowledge Base, but are represented as a single node in the network. The intensity of the node color indicates the degree of up- (red) or down- (green) regulation. Nodes are displayed using various shapes that represent the functional class of the gene product. Edges are displayed with various labels that describe the nature of the relationship between the nodes (see figure legends for details). IPA also allows prediction of the activation or inhibition of transcription factors involved in the gene expression patterns seen in our study.

### Validation of cDNA Microarray by Quantitative RT-PCR

Quantitation of mRNA levels was performed by the CHDD Genomics Core Laboratory using fluorogenic 5′ nuclease-based assays and has been previously described. [90], [91], [92].

## Supporting Information

Figure S1 **Comparison of mRNA expression in the fetal lung by microarray analysis in GBS and saline groups displaying the relative Cy3/Cy5 ratios.** mRNA expression is displayed as either higher (red) or lower (green) in GBS compared to saline controls.
(TIF)

Table S1 **All probe sets in the single gene analysis differentially expressed at the 1.5-fold level, p<0.05.**
(DOCX)

Table S2 **Gene sets upregulated after GBS exposure relative to the control group.**
(DOCX)

Table S3 **Gene sets downregulated after GBS exposure relative to the control group.**
(DOCX)
