# Correction: Choriodecidual Infection Downregulates Angiogenesis and Morphogenesis Pathways in Fetal Lungs from *Macaca Nemestrina*

**DOI:** 10.1371/annotation/2832117a-0601-4416-a256-c7e64c541a0b

**Published:** 2013-02-07

**Authors:** Ryan M. McAdams, Jeroen Vanderhoeven, Richard P. Beyer, Theo K. Bammler, Federico M. Farin, H. Denny Liggitt, Raj P. Kapur, Michael G. Gravett, Craig E. Rubens, Kristina M. Adams Waldorf

The legends for Figures 3 and 4 were incorrectly switched. The legend that appears with Figure 3 should be with Figure 4, and the legend that appears with Figure 4 should be with Figure 3.

