# Supplementary figures and images for: Choriodecidual Infection Downregulates Angiogenesis and Morphogenesis Pathways in Fetal Lungs from Macaca Nemestrina
Source: PLoS One. 2012 Oct 9;7(10):e46863. doi: 10.1371/journal.pone.0046863 (PMC3467273; doi:10.1371/journal.pone.0046863)

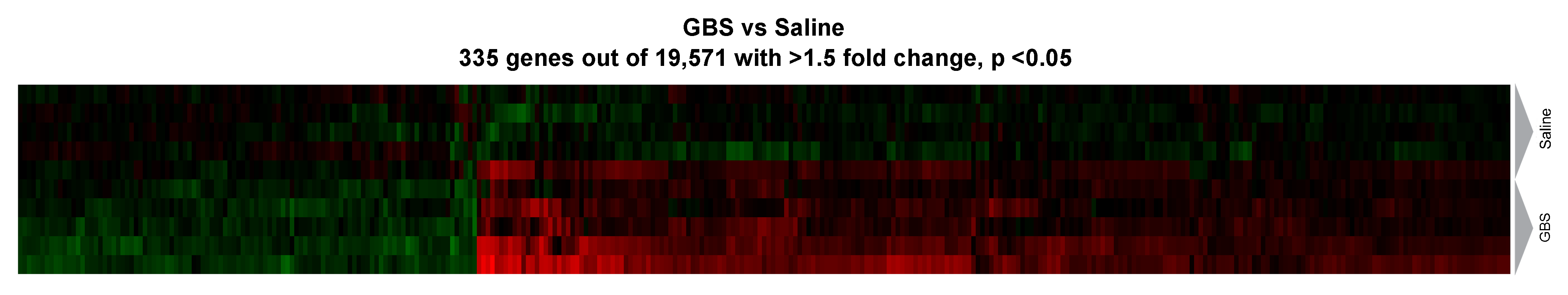

Supplement: Figure S1 — Comparison of mRNA expression in the fetal lung by microarray analysis in GBS and saline groups displaying the relative Cy3/Cy5 ratios. mRNA expression is displayed as either higher (red) or lower (green) in GBS compared to saline controls. (TIF) [file pone.0046863.s001.tif]
