# Supplementary material for: Choriodecidual Infection Downregulates Angiogenesis and Morphogenesis Pathways in Fetal Lungs from Macaca Nemestrina
Source: PLoS One. 2012 Oct 9;7(10):e46863. doi: 10.1371/journal.pone.0046863 (PMC3467273; doi:10.1371/journal.pone.0046863)
Supplement: Table S1 — All probe sets in the single gene analysis differentially expressed at the 1.5-fold level, p<0.05. (DOCX) [file pone.0046863.s002.docx]

Table S1. All probe sets in the single gene analysis differentially expressed at the 1.5-fold level, p < 0.05.

| ProbeID | Ensembl | log2 fold  change | Rhesus Entrez  Gene | Description | Symbol |
| --- | --- | --- | --- | --- | --- |
|  |  |  |  |  |  |
| Mmu.10025.1.S1_at | ENSMMUG00000010093 | 0.693 | 711437 | cathepsin H | CTSH |
| Mmu.10083.1.S1_s_at | ENSMMUG00000011218 | 1.928 | 574106 | serpin peptidase inhibitor, clade A (alpha-1 antiproteinase, antitrypsin), member 3 | SERPINA3 |
| Mmu.10122.1.S1_at | --- | 1.351 | 708080 | similar to Cathepsin S precursor | LOC708080 |
| Mmu.10209.2.S1_at | --- | -3.490 | --- | --- | --- |
| Mmu.10246.2.S1_x_at | --- | 0.737 | --- | --- | --- |
| Mmu.10472.1.S1_at | ENSMMUG00000021905 | 1.049 | 713696 | sphingomyelin phosphodiesterase, acid-like 3A | SMPDL3A |
| Mmu.10556.1.S1_s_at | ENSMMUG00000004492 | 0.896 | 719260 | absent in melanoma 2 | AIM2 |
| Mmu.10941.2.S1_at | ENSMMUG00000000413 | 0.601 | 712466 | hypothetical protein LOC712466 | LOC712466 |
| Mmu.11363.1.S1_at | ENSMMUG00000020903 | 2.105 | 574243 | chemokine (C-X-C motif) ligand 10 | CXCL10 |
| Mmu.11668.1.S1_at | ENSMMUG00000002037 | 1.036 | 703653 | matrix metallopeptidase 1 (interstitial collagenase) | MMP1 |
| Mmu.1183.2.S1_at | ENSMMUG00000008227 /// ENSMMUG00000029799 | 1.609 | 677701 /// 692100 /// 705588 /// 721522 | similar to HLA class II histocompatibility antigen, DRB1-4 beta chain precursor (MHC class I antigen DRB1*4) (DR-4) (DR4) /// similar to HLA class II histocompatibility antigen, DRB1-7 beta chain precursor (MHC class I antigen DRB1*7) (DR-7) (DR7) /// MHC class II antigen /// MHC class II antigen, Mamu-DRB5 | LOC705588 /// LOC721522 /// MAMU-DRB /// MAMU-DRB5 |
| Mmu.11960.1.S1_at | ENSMMUG00000000662 | 1.369 | 694538 | guanylate binding protein 1, interferon-inducible, 67kD | GBP1 |
| Mmu.11960.1.S1_x_at | ENSMMUG00000000662 | 1.509 | 694538 | guanylate binding protein 1, interferon-inducible, 67kD | GBP1 |
| Mmu.13156.4.S1_x_at | --- | 1.018 | 705588 | similar to HLA class II histocompatibility antigen, DRB1-4 beta chain precursor (MHC class I antigen DRB1*4) (DR-4) (DR4) | LOC705588 |
| Mmu.13791.1.S1_at | --- | 1.091 | 722003 | Similar to eukaryotic translation elongation factor 1 alpha 1 | LOC722003 |
| Mmu.14068.1.S1_at | ENSMMUG00000014176 | 0.646 | 697677 | cytohesin 1 interacting protein | CYTIP |
| Mmu.14068.2.A1_at | --- | 0.717 | --- | --- | --- |
| Mmu.14152.1.S1_at | --- | 0.666 | 693936 | IKAROS family zinc finger 1 (Ikaros) | IKZF1 |
| Mmu.14249.1.S1_at | --- | 0.772 | 735311 | chemokine (C-C motif) receptor 5 | CCR5 |
| Mmu.14389.1.S1_at | ENSMMUG00000009755 | 0.645 | 696276 | ubiquitin-conjugating enzyme E2W (putative) | UBE2W |
| Mmu.14436.1.S1_at | ENSMMUG00000011620 | 0.909 | 701010 | mannose receptor, C type 1 | MRC1 |
| Mmu.14728.1.S1_at | ENSMMUG00000020385 | 0.620 | 698947 | similar to zinc finger protein 395 | LOC698947 |
| Mmu.1485.1.S1_at | ENSMMUG00000000811 | 0.829 | 702350 | CD53 molecule | CD53 |
| Mmu.15276.1.S1_s_at | ENSMMUG00000013098 | -0.969 | 706293 | similar to aldo-keto reductase family 1, member B10 | LOC706293 |
| Mmu.15738.1.S1_at | ENSMMUG00000006528 | 1.190 | 705311 | Rho GTPase activating protein 15 | ARHGAP15 |
| Mmu.16215.1.S1_at | ENSMMUG00000012786 | 0.952 | 714589 | complement component 1, s subcomponent | C1S |
| Mmu.2018.1.S1_at | ENSMMUG00000022626 | 1.118 | 717870 | similar to HLA class II histocompatibility antigen, DM beta chain precursor (MHC class II antigen DMB) | LOC717870 |
| Mmu.2053.1.S1_s_at | ENSMMUG00000030216 | 2.392 | 619514 | chemokine (C-C motif) ligand 3 | CCL3 |
| Mmu.2177.1.S1_x_at | ENSMMUG00000003765 /// ENSMMUG00000023461 /// ENSMMUG00000029029 | 0.887 | 1E+08 | major histocompatibility complex, class I, B | MAMU-B18 |
| Mmu.249.1.S1_at | ENSMMUG00000023598 | 0.673 | 700507 | sorting nexin 2 | SNX2 |
| Mmu.2883.1.S1_at | ENSMMUG00000014171 | 0.630 | 697172 | similar to complement component 7 precursor | LOC697172 |
| Mmu.3346.2.S1_at | ENSMMUG00000005529 | 0.978 | 712210 | ceruloplasmin (ferroxidase) | CP |
| Mmu.4013.1.S1_at | ENSMMUG00000004530 | -0.704 | 693975 | transmembrane protein 47 | TMEM47 |
| Mmu.4098.1.S1_at | --- | 0.846 | 699733 | syntaxin 11 | STX11 |
| Mmu.414.1.S1_at | ENSMMUG00000014171 | 0.652 | 696926 | complement component 7 | C7 |
| Mmu.4423.1.S1_at | ENSMMUG00000023648 | 0.670 | 709083 | similar to myeloid/lymphoid or mixed lineage-leukemia translocation to 10 homolog | LOC709083 |
| Mmu.452.1.S1_at | ENSMMUG00000021180 | 0.900 | 719466 | growth differentiation factor 15 | GDF15 |
| Mmu.4846.2.S1_at | ENSMMUG00000000016 | -1.089 | 713580 | adenylosuccinate synthase | ADSS |
| Mmu.5491.1.S1_at | ENSMMUG00000012969 | 0.586 | 722479 | Similar to Nucleoside diphosphate kinase 3 (NDK 3) (NDP kinase 3) (Nucleoside diphosphate kinase C) (NDPKC) (nm23-H3) (DR-nm23) | LOC722479 |
| Mmu.6271.1.S1_at | ENSMMUG00000008603 | 0.714 | 712972 | ecotropic viral integration site 2B | EVI2B |
| Mmu.6271.2.S1_a_at | ENSMMUG00000008603 | 0.726 | 712972 | ecotropic viral integration site 2B | EVI2B |
| Mmu.6272.2.S1_at | ENSMMUG00000008604 | 1.182 | 712972 /// 713110 | ecotropic viral integration site 2B /// similar to ecotropic viral integration site 2A isoform 2 | EVI2B /// LOC713110 |
| Mmu.629.1.S1_s_at | ENSMMUG00000022139 | 0.610 | 701346 | Similar to 60 kDa heat shock protein, mitochondrial precursor (Hsp60) (60 kDa chaperonin) (CPN60) (Heat shock protein 60) (HSP-60) (Mitochondrial matrix protein P1) (P60 lymphocyte protein) (HuCHA60) | LOC701346 |
| Mmu.629.2.S1_a_at | ENSMMUG00000022139 | 1.274 | 701346 | Similar to 60 kDa heat shock protein, mitochondrial precursor (Hsp60) (60 kDa chaperonin) (CPN60) (Heat shock protein 60) (HSP-60) (Mitochondrial matrix protein P1) (P60 lymphocyte protein) (HuCHA60) | LOC701346 |
| Mmu.629.2.S1_at | ENSMMUG00000022139 | 1.084 | 701346 | Similar to 60 kDa heat shock protein, mitochondrial precursor (Hsp60) (60 kDa chaperonin) (CPN60) (Heat shock protein 60) (HSP-60) (Mitochondrial matrix protein P1) (P60 lymphocyte protein) (HuCHA60) | LOC701346 |
| Mmu.6471.1.S1_x_at | ENSMMUG00000030216 | 1.227 | 619514 | chemokine (C-C motif) ligand 3 | CCL3 |
| Mmu.6716.1.S1_at | ENSMMUG00000010670 | 1.297 | 708080 | similar to Cathepsin S precursor | LOC708080 |
| Mmu.6730.1.S1_at | ENSMMUG00000022311 | 1.371 | --- | --- | --- |
| Mmu.7150.1.S1_at | ENSMMUG00000003619 | 0.635 | --- | --- | --- |
| Mmu.8081.1.S1_at | ENSMMUG00000012324 /// ENSMMUG00000013098 | -1.898 | 706406 /// 713150 | similar to aldo-keto reductase family 1, member B10 /// similar to aldo-keto reductase family 1, member B10 | LOC706406 /// LOC713150 |
| Mmu.8081.1.S1_x_at | ENSMMUG00000012324 | -1.405 | 713150 | similar to aldo-keto reductase family 1, member B10 | LOC713150 |
| Mmu.8608.1.S2_at | --- | 1.111 | 574178 | chemokine (C-C motif) ligand 5 | CCL5 |
| Mmu.9241.2.S1_at | ENSMMUG00000009113 | 1.661 | 710820 | CD74 molecule, major histocompatibility complex, class II invariant chain | CD74 |
| Mmu.9805.1.S1_at | ENSMMUG00000029350 | 0.842 | 708329 | ERO1-like | ERO1L |
| Mmu.9964.1.S1_at | --- | -0.764 | --- | --- | --- |
| MmugDNA.10110.1.S1_at | ENSMMUG00000001657 | 0.902 | 704579 | ribonuclease, RNase A family, k6 | RNASE6 |
| MmugDNA.1022.1.S1_s_at | --- | 0.792 | --- | --- | --- |
| MmugDNA.1028.1.S1_at | ENSMMUG00000019258 | 1.566 | 100187587 /// 720539 | similar to HLA class II histocompatibility antigen, DR alpha chain precursor (MHC class II antigen DRA) /// major histocompatibility complex, class II, DR alpha | LOC720539 /// MAMU-DRA |
| MmugDNA.1034.1.S1_at | --- | -0.589 | 697319 | Similar to EMSY protein | LOC697319 |
| MmugDNA.10362.1.S1_at | --- | -0.645 | --- | --- | --- |
| MmugDNA.1042.1.S1_s_at | ENSMMUG00000008799 | 1.150 | 709076 | major histocompatibility complex, class I, F | MAMU-F |
| MmugDNA.1046.1.S1_at | --- | 1.685 | 100187587 /// 723547 | similar to HLA class II histocompatibility antigen, DR alpha chain precursor (MHC class II antigen DRA) /// major histocompatibility complex, class II, DR alpha | LOC723547 /// MAMU-DRA |
| MmugDNA.1046.1.S1_s_at | ENSMMUG00000019258 | 1.476 | 1E+08 | major histocompatibility complex, class II, DR alpha | MAMU-DRA |
| MmugDNA.10481.1.S1_at | --- | 0.977 | 697677 | cytohesin 1 interacting protein | CYTIP |
| MmugDNA.10537.1.S1_at | --- | 0.884 | --- | --- | --- |
| MmugDNA.10659.1.S1_at | --- | 1.110 | --- | --- | --- |
| MmugDNA.1068.1.S1_at | ENSMMUG00000030017 | 0.600 | 718513 | similar to insulin induced gene 1 isoform 2 | LOC718513 |
| MmugDNA.10735.1.S1_at | ENSMMUG00000011772 | -0.631 | 722280 | adenomatosis polyposis coli down-regulated 1 | APCDD1 |
| MmugDNA.1081.1.S1_at | ENSMMUG00000029790 | 0.720 | 717726 | transporter 1, ATP-binding cassette, sub-family B (MDR/TAP) | TAP1 |
| MmugDNA.10834.1.S1_at | ENSMMUG00000005296 | 1.144 | 574115 | allograft inflammatory factor 1 | AIF1 |
| MmugDNA.10839.1.S1_at | --- | -0.889 | 710295 | parathyroid hormone-like hormone | PTHLH |
| MmugDNA.10998.1.S1_at | --- | -0.866 | 695775 | KIAA1009 | KIAA1009 |
| MmugDNA.11207.1.S1_at | --- | -0.648 | 709955 | zinc finger protein 559 | ZNF559 |
| MmugDNA.11299.1.S1_at | --- | -0.702 | --- | --- | --- |
| MmugDNA.11328.1.S1_at | ENSMMUG00000012185 | 0.819 | 710832 | activating transcription factor 3 | ATF3 |
| MmugDNA.11353.1.S1_at | --- | 1.746 | --- | --- | --- |
| MmugDNA.1158.1.S1_at | --- | -0.841 | --- | --- | --- |
| MmugDNA.11615.1.S1_at | --- | -0.845 | --- | --- | --- |
| MmugDNA.11857.1.S1_at | --- | 0.626 | --- | --- | --- |
| MmugDNA.12153.1.S1_at | ENSMMUG00000000731 | 0.979 | 716809 | complement factor B | CFB |
| MmugDNA.12369.1.S1_at | --- | -0.705 | --- | --- | --- |
| MmugDNA.12574.1.S1_s_at | --- | 0.669 | 705838 | carbonic anhydrase XII | CA12 |
| MmugDNA.12584.1.S1_at | --- | -0.608 | 701181 | Similar to DNA-directed RNA polymerase II 7.6 kDa polypeptide (RPB10) (RPB7.6) (RPABC5) | LOC701181 |
| MmugDNA.12725.1.S1_at | --- | -0.688 | --- | --- | --- |
| MmugDNA.12761.1.S1_at | --- | -0.675 | --- | --- | --- |
| MmugDNA.1278.1.S1_at | --- | -0.669 | --- | --- | --- |
| MmugDNA.12814.1.S1_at | --- | -0.603 | --- | --- | --- |
| MmugDNA.12878.1.S1_at | --- | -0.607 | 711598 | similar to Transmembrane anchor protein 1 | LOC711598 |
| MmugDNA.12881.1.S1_at | --- | 1.490 | 694532 | cholesterol 25-hydroxylase | CH25H |
| MmugDNA.12973.1.S1_at | ENSMMUG00000007870 | 0.708 | 699802 | Similar to melanoma antigen family C, 1 | LOC699802 |
| MmugDNA.13052.1.S1_at | --- | 1.223 | 705017 | phosphoinositide-3-kinase adaptor protein 1 | PIK3AP1 |
| MmugDNA.1315.1.S1_at | --- | -0.698 | --- | --- | --- |
| MmugDNA.13316.1.S1_at | --- | -0.777 | --- | --- | --- |
| MmugDNA.13403.1.S1_at | --- | 1.109 | 718056 | complement component 5a receptor 1 | C5AR1 |
| MmugDNA.13610.1.S1_at | --- | -0.603 | --- | --- | --- |
| MmugDNA.13687.1.S1_at | --- | -0.593 | --- | --- | --- |
| MmugDNA.13726.1.S1_at | --- | -0.655 | --- | --- | --- |
| MmugDNA.13757.1.S1_at | ENSMMUG00000022311 | 1.218 | 693310 | placenta-specific 8 | PLAC8 |
| MmugDNA.13865.1.S1_at | --- | -0.610 | --- | --- | --- |
| MmugDNA.13892.1.S1_at | --- | -0.899 | --- | --- | --- |
| MmugDNA.13903.1.S1_at | --- | -0.692 | --- | --- | --- |
| MmugDNA.14045.1.S1_at | ENSMMUG00000005319 | -1.050 | 712581 | advillin | AVIL |
| MmugDNA.14204.1.S1_at | ENSMMUG00000014381 | 0.940 | 716518 | granzyme B (granzyme 2, cytotoxic T-lymphocyte-associated serine esterase 1) | GZMB |
| MmugDNA.14262.1.S1_at | --- | -0.616 | --- | --- | --- |
| MmugDNA.14283.1.S1_at | ENSMMUG00000007506 | 1.241 | --- | --- | --- |
| MmugDNA.14295.1.S1_at | --- | 0.788 | --- | --- | --- |
| MmugDNA.14316.1.S1_at | --- | -0.788 | --- | --- | --- |
| MmugDNA.14336.1.S1_at | --- | 0.826 | --- | --- | --- |
| MmugDNA.14342.1.S1_at | ENSMMUG00000014789 | 0.688 | 718718 | CD37 molecule | CD37 |
| MmugDNA.14367.1.S1_at | --- | -0.608 | --- | --- | --- |
| MmugDNA.14371.1.S1_at | --- | -0.686 | --- | --- | --- |
| MmugDNA.14375.1.S1_at | ENSMMUG00000015812 | 1.399 | 719762 | CD48 molecule | CD48 |
| MmugDNA.14551.1.S1_at | --- | 0.619 | 709295 | similar to cat eye syndrome critical region protein 1 isoform a precursor | LOC709295 |
| MmugDNA.14681.1.S1_at | --- | -0.637 | --- | --- | --- |
| MmugDNA.1472.1.S1_s_at | ENSMMUG00000010460 | -0.637 | 700567 | RAS-like, estrogen-regulated, growth inhibitor | RERG |
| MmugDNA.14767.1.S1_at | --- | -0.697 | 696113 | FRAS1 related extracellular matrix protein 2 | FREM2 |
| MmugDNA.14779.1.S1_at | ENSMMUG00000004961 | -0.623 | 706456 | G elongation factor, mitochondrial 1 | GFM1 |
| MmugDNA.14891.1.S1_at | --- | -0.668 | --- | --- | --- |
| MmugDNA.14892.1.S1_at | --- | -0.624 | 700892 | iduronate 2-sulfatase | IDS |
| MmugDNA.14930.1.S1_at | ENSMMUG00000012307 | -0.659 | --- | --- | --- |
| MmugDNA.15084.1.S1_s_at | ENSMMUG00000010240 | 0.716 | 712017 | Similar to PML-RARA regulated adaptor molecule 1 | LOC712017 |
| MmugDNA.15107.1.S1_at | ENSMMUG00000015865 | 0.622 | 692336 | Leukocyte immunoglobulin-like receptor, subfamily B, member c | LILRBC |
| MmugDNA.15194.1.S1_at | --- | 0.971 | --- | --- | --- |
| MmugDNA.15319.1.S1_at | ENSMMUG00000003476 | -0.677 | 705316 | EH-domain containing 3 | EHD3 |
| MmugDNA.1533.1.S1_at | ENSMMUG00000005534 | 1.404 | 717870 | similar to HLA class II histocompatibility antigen, DM beta chain precursor (MHC class II antigen DMB) | LOC717870 |
| MmugDNA.15406.1.S1_at | --- | -0.810 | --- | --- | --- |
| MmugDNA.15546.1.S1_at | --- | -0.670 | --- | --- | --- |
| MmugDNA.15576.1.S1_at | --- | 0.662 | 692340 | leukocyte immunoglobulin-like receptor, subfamily B, member a | LILRBA |
| MmugDNA.15595.1.S1_at | ENSMMUG00000004362 | 1.254 | 705855 | HtrA serine peptidase 1 | HTRA1 |
| MmugDNA.15824.1.S1_at | --- | -0.636 | --- | --- | --- |
| MmugDNA.15842.1.S1_at | --- | 0.735 | --- | --- | --- |
| MmugDNA.15862.1.S1_at | ENSMMUG00000012534 | 0.612 | 697324 | similar to CDC28 protein kinase 2 | LOC697324 |
| MmugDNA.15909.1.S1_at | --- | -0.606 | --- | --- | --- |
| MmugDNA.15931.1.S1_at | ENSMMUG00000004647 | -0.625 | 701107 | relaxin/insulin-like family peptide receptor 1 | RXFP1 |
| MmugDNA.16026.1.S1_at | --- | 0.624 | --- | --- | --- |
| MmugDNA.16313.1.S1_at | --- | 0.695 | 719241 | pyrin and HIN domain family, member 1 | PYHIN1 |
| MmugDNA.16555.1.S1_at | ENSMMUG00000009593 | 0.634 | 698446 | WAS/WASL interacting protein family, member 1 | WIPF1 |
| MmugDNA.16681.1.S1_at | --- | -0.916 | --- | --- | --- |
| MmugDNA.16779.1.S1_at | ENSMMUG00000016848 | 0.587 | 699062 | solute carrier organic anion transporter family, member 4A1 | SLCO4A1 |
| MmugDNA.16846.1.S1_at | ENSMMUG00000000242 | -0.728 | --- | --- | --- |
| MmugDNA.16876.1.S1_at | ENSMMUG00000014757 | -0.812 | 694130 | synaptotagmin XVII | SYT17 |
| MmugDNA.17015.1.S1_at | --- | -0.736 | --- | --- | --- |
| MmugDNA.17139.1.S1_s_at | ENSMMUG00000007039 | 0.635 | 716455 | Similar to syntaxin 12 | LOC716455 |
| MmugDNA.17191.1.S1_at | --- | 0.890 | --- | --- | --- |
| MmugDNA.17253.1.S1_at | ENSMMUG00000030808 | -0.621 | 721498 | centromere protein J | CENPJ |
| MmugDNA.17276.1.S1_at | --- | -0.716 | --- | --- | --- |
| MmugDNA.17292.1.S1_at | --- | -0.811 | --- | --- | --- |
| MmugDNA.17363.1.S1_at | ENSMMUG00000018854 | 0.838 | 696709 | ras-related C3 botulinum toxin substrate 2 (rho family, small GTP binding protein Rac2) | RAC2 |
| MmugDNA.17373.1.S1_at | ENSMMUG00000004833 | -0.705 | 714943 | S100 calcium binding protein A7 | S100A7 |
| MmugDNA.17376.1.S1_at | ENSMMUG00000004831 | 1.311 | 714740 | S100 calcium binding protein A8 | S100A8 |
| MmugDNA.17449.1.S1_at | ENSMMUG00000016347 | 1.560 | 704124 | transcription factor EC | TFEC |
| MmugDNA.17565.1.S1_at | --- | -0.854 | 709260 | Similar to sno, strawberry notch homolog 1 | LOC709260 |
| MmugDNA.17577.1.S1_at | --- | -0.589 | --- | --- | --- |
| MmugDNA.17588.1.S1_at | ENSMMUG00000000731 | 0.934 | 716809 | complement factor B | CFB |
| MmugDNA.17590.1.S1_at | --- | -0.587 | --- | --- | --- |
| MmugDNA.17604.1.S1_at | --- | -0.688 | 714878 | zinc finger protein 397 | ZNF397 |
| MmugDNA.17656.1.S1_at | --- | -0.799 | --- | --- | --- |
| MmugDNA.17668.1.S1_at | --- | -0.605 | --- | --- | --- |
| MmugDNA.17819.1.S1_at | ENSMMUG00000016549 | 1.864 | 708084 | matrix metallopeptidase 9 (gelatinase B, 92kDa gelatinase, 92kDa type IV collagenase) | MMP9 |
| MmugDNA.17946.1.S1_s_at | ENSMMUG00000007395 | 0.702 | 695170 | similar to bicaudal D homolog 1 isoform 1 | LOC695170 |
| MmugDNA.18001.1.S1_at | --- | -0.790 | 695935 | FAT tumor suppressor homolog 3 (Drosophila) | FAT3 |
| MmugDNA.18015.1.S1_at | ENSMMUG00000022626 | 1.130 | 717870 | similar to HLA class II histocompatibility antigen, DM beta chain precursor (MHC class II antigen DMB) | LOC717870 |
| MmugDNA.18024.1.S1_at | ENSMMUG00000022062 | 0.691 | 712895 | TRAF3 interacting protein 3 | TRAF3IP3 |
| MmugDNA.18037.1.S1_at | --- | 1.870 | --- | --- | --- |
| MmugDNA.18099.1.S1_at | ENSMMUG00000008693 | 0.591 | 701958 | ADAM metallopeptidase domain 18 | ADAM18 |
| MmugDNA.18224.1.S1_at | ENSMMUG00000017260 | 1.221 | 693449 | G protein-coupled receptor 65 | GPR65 |
| MmugDNA.18226.1.S1_at | --- | -0.734 | --- | --- | --- |
| MmugDNA.1831.1.S1_at | --- | -0.631 | 700292 | hypothetical protein LOC700292 | LOC700292 |
| MmugDNA.1832.1.S1_at | --- | 1.197 | 715052 | neutrophil cytosolic factor 2 | NCF2 |
| MmugDNA.18432.1.S1_at | ENSMMUG00000019304 | 2.959 | 574370 | indoleamine 2,3-dioxygenase 1 | IDO1 |
| MmugDNA.18446.1.S1_s_at | --- | -0.634 | 704335 | KDEL (Lys-Asp-Glu-Leu) endoplasmic reticulum protein retention receptor 2 | KDELR2 |
| MmugDNA.18463.1.S1_at | --- | -0.618 | --- | --- | --- |
| MmugDNA.18472.1.S1_at | --- | -0.703 | 716909 | wingless-type MMTV integration site family, member 3 | WNT3 |
| MmugDNA.18498.1.S1_at | ENSMMUG00000003834 | 0.965 | 702646 | basic leucine zipper transcription factor, ATF-like | BATF |
| MmugDNA.18601.1.S1_at | ENSMMUG00000019367 | 1.440 | 717969 | major histocompatibility complex, class II, DP beta | MAMU-DPB |
| MmugDNA.18603.1.S1_at | ENSMMUG00000020158 | 1.104 | 695437 /// 705266 | Similar to C03A3.3 /// Similar to peptidylprolyl isomerase A isoform 1 | LOC695437 /// LOC705266 |
| MmugDNA.18723.1.S1_at | --- | -0.802 | --- | --- | --- |
| MmugDNA.18754.1.S1_at | ENSMMUG00000012696 | -0.684 | 712991 | zinc finger protein 300 | ZNF300 |
| MmugDNA.18781.1.S1_at | --- | 0.611 | --- | --- | --- |
| MmugDNA.18838.1.S1_at | --- | -1.178 | --- | --- | --- |
| MmugDNA.18917.1.S1_at | --- | -0.618 | 711519 | hypothetical protein LOC711519 | LOC711519 |
| MmugDNA.18922.1.S1_at | --- | 0.644 | --- | --- | --- |
| MmugDNA.18927.1.S1_at | --- | -0.989 | --- | --- | --- |
| MmugDNA.1894.1.S1_s_at | ENSMMUG00000011835 | -0.625 | --- | --- | --- |
| MmugDNA.19056.1.S1_at | ENSMMUG00000018563 | 1.031 | 698123 | somatostatin receptor 1 | SSTR1 |
| MmugDNA.19059.1.S1_at | --- | 0.590 | --- | --- | --- |
| MmugDNA.19066.1.S1_at | ENSMMUG00000016206 | 1.039 | 720006 | Fc gamma RIIIa | FCGR3 |
| MmugDNA.19209.1.S1_s_at | ENSMMUG00000023801 | 1.972 | 697208 | lipocalin 2 | LCN2 |
| MmugDNA.19245.1.S1_at | ENSMMUG00000009112 | 0.884 | 574360 | toll-like receptor 4 | TLR4 |
| MmugDNA.19485.1.S1_s_at | ENSMMUG00000004327 | 0.792 | 702435 | Similar to protocadherin 1 isoform 2 precursor | LOC702435 |
| MmugDNA.19508.1.S1_at | ENSMMUG00000003836 | 1.931 | 613028 | interleukin 8 | IL8 |
| MmugDNA.19691.1.S1_at | ENSMMUG00000010906 | 0.768 | 701365 | fatty acid binding protein 4, adipocyte | FABP4 |
| MmugDNA.19732.1.S1_s_at | ENSMMUG00000015764 | 0.966 | 710951 | similar to T-cell receptor alpha chain C region | LOC710951 |
| MmugDNA.19939.1.S1_s_at | ENSMMUG00000015790 | 0.705 | 702941 | lymphatic vessel endothelial hyaluronan receptor 1 | LYVE1 |
| MmugDNA.2014.1.S1_at | --- | 0.763 | --- | --- | --- |
| MmugDNA.20186.1.S1_at | --- | 0.701 | --- | --- | --- |
| MmugDNA.20240.1.S1_s_at | ENSMMUG00000005319 | -0.823 | 712581 | advillin | AVIL |
| MmugDNA.20310.1.S1_at | --- | -0.786 | --- | --- | --- |
| MmugDNA.20313.1.S1_at | --- | 0.828 | 704383 | interleukin 10 receptor, alpha | IL10RA |
| MmugDNA.20383.1.S1_at | --- | -0.686 | --- | --- | --- |
| MmugDNA.20547.1.S1_at | --- | -0.689 | 714758 | similar to Zinc finger protein 267 (Zinc finger protein HZF2) | LOC714758 |
| MmugDNA.20749.1.S1_at | --- | -0.593 | 694130 | synaptotagmin XVII | SYT17 |
| MmugDNA.20755.1.S1_at | --- | -0.950 | --- | --- | --- |
| MmugDNA.20774.1.S1_at | --- | 1.368 | --- | --- | --- |
| MmugDNA.20995.1.S1_at | ENSMMUG00000007506 | 1.591 | --- | --- | --- |
| MmugDNA.210.1.S1_at | ENSMMUG00000022224 | -0.960 | 708339 | similar to interleukin 13 receptor, alpha 2 precursor | IL5RA |
| MmugDNA.21025.1.S1_at | ENSMMUG00000022101 | -0.609 | 706175 | similar to cold inducible RNA binding protein | LOC706175 |
| MmugDNA.21033.1.S1_at | --- | 0.651 | --- | --- | --- |
| MmugDNA.21050.1.S1_at | --- | -0.627 | 700667 | hypothetical protein LOC700667 | LOC700667 |
| MmugDNA.2108.1.S1_s_at | ENSMMUG00000010341 | 1.213 | 711156 | secretory leukocyte peptidase inhibitor | SLPI |
| MmugDNA.21255.1.S1_at | ENSMMUG00000007586 | 1.133 | 694207 | neutrophil cytosolic factor 1 | NCF1 |
| MmugDNA.21330.1.S1_at | ENSMMUG00000002665 | 0.648 | 712272 | similar to fibronectin type 3 and SPRY domain-containing protein | LOC712272 |
| MmugDNA.21475.1.S1_at | --- | 0.671 | --- | --- | --- |
| MmugDNA.21746.1.S1_at | ENSMMUG00000004530 | -0.610 | 693975 | transmembrane protein 47 | TMEM47 |
| MmugDNA.2176.1.S1_at | ENSMMUG00000007669 | 0.852 | 698376 | interferon stimulated exonuclease gene 20kDa | ISG20 |
| MmugDNA.22091.1.S1_at | --- | 1.169 | 716425 | Similar to Transcription initiation factor TFIID subunit 9 (Transcription initiation factor TFIID 31 kDa subunit) (TAFII-31) (TAFII-32) (TAFII32) (STAF31/32) | TAF9 |
| MmugDNA.22160.1.S1_at | --- | 0.660 | 707383 | amyloid beta (A4) precursor protein-binding, family B, member 1 interacting protein | APBB1IP |
| MmugDNA.22297.1.S1_at | ENSMMUG00000008987 | 1.216 | 718361 | lysozyme | LYZ |
| MmugDNA.22360.1.S1_at | ENSMMUG00000011896 | 0.916 | 701088 | similar to proteasome beta 10 subunit proprotein | LOC701088 |
| MmugDNA.22360.1.S1_s_at | ENSMMUG00000011896 | 0.823 | 701088 | similar to proteasome beta 10 subunit proprotein | LOC701088 |
| MmugDNA.22361.1.S1_at | ENSMMUG00000019831 | -0.605 | --- | --- | --- |
| MmugDNA.22369.1.S1_at | ENSMMUG00000005185 | 0.741 | 693383 | regulator of G-protein signaling 4 | RGS4 |
| MmugDNA.22506.1.S1_at | ENSMMUG00000021330 | 1.379 | 705179 | kynureninase (L-kynurenine hydrolase) | KYNU |
| MmugDNA.22506.1.S1_s_at | ENSMMUG00000021330 | 1.315 | 705179 | kynureninase (L-kynurenine hydrolase) | KYNU |
| MmugDNA.22572.1.S1_at | --- | -0.624 | --- | --- | --- |
| MmugDNA.22748.1.S1_at | --- | 0.718 | --- | --- | --- |
| MmugDNA.22788.1.S1_s_at | --- | 0.616 | --- | --- | --- |
| MmugDNA.22814.1.S1_at | --- | 0.670 | --- | --- | --- |
| MmugDNA.22932.1.S1_at | ENSMMUG00000011304 | 0.779 | 703405 | Similar to ribosomal protein S14 | LOC703405 |
| MmugDNA.22991.1.S1_at | ENSMMUG00000012473 | 0.596 | 715288 | proteasome (prosome, macropain) activator subunit 2 (PA28 beta) | PSME2 |
| MmugDNA.23177.1.S1_at | --- | 0.682 | --- | --- | --- |
| MmugDNA.23209.1.S1_at | ENSMMUG00000002192 | 0.610 | 574356 | C-type lectin domain family 7, member A | CLEC7A |
| MmugDNA.23317.1.S1_at | ENSMMUG00000011720 | 0.615 | 708081 | G patch domain containing 2 | GPATCH2 |
| MmugDNA.23338.1.S1_at | --- | -0.687 | --- | --- | --- |
| MmugDNA.23339.1.S1_at | --- | -0.703 | --- | --- | --- |
| MmugDNA.23348.1.S1_at | --- | -0.832 | --- | --- | --- |
| MmugDNA.23367.1.S1_s_at | ENSMMUG00000007410 | 1.988 | 715967 | hypothetical protein LOC715967 | LOC715967 |
| MmugDNA.23418.1.S1_at | --- | -0.720 | --- | --- | --- |
| MmugDNA.23424.1.S1_at | ENSMMUG00000020374 | 0.668 | 705568 | Similar to signal recognition particle 68kDa | LOC705568 |
| MmugDNA.23458.1.S1_at | --- | -0.739 | 694216 | similar to UL16 binding protein 1 | LOC694216 |
| MmugDNA.23460.1.S1_s_at | --- | -0.591 | 706237 | thiamin pyrophosphokinase 1 | TPK1 |
| MmugDNA.23533.1.S1_at | --- | -0.723 | 704838 | hypothetical protein LOC704838 | LOC704838 |
| MmugDNA.23544.1.S1_s_at | ENSMMUG00000003775 /// ENSMMUG00000031519 | 1.447 | 694225 | Similar to DCP2 decapping enzyme | LOC694225 |
| MmugDNA.2360.1.S1_at | ENSMMUG00000001012 | -0.742 | 713062 | 6-phosphofructo-2-kinase/fructose-2,6-biphosphatase 3 | PFKFB3 |
| MmugDNA.23693.1.S1_at | ENSMMUG00000014670 | 0.688 | 706853 | phosphorylase, glycogen, liver | PYGL |
| MmugDNA.24030.1.S1_at | ENSMMUG00000005885 | 1.963 | 574097 | Mn-superoxide dismutase | LOC574097 |
| MmugDNA.24072.1.S1_at | --- | -0.606 | --- | --- | --- |
| MmugDNA.24181.1.S1_s_at | ENSMMUG00000022363 | -0.622 | 722689 | dihydropyrimidinase-like 4 | DPYSL4 |
| MmugDNA.24184.1.S1_at | --- | -0.763 | --- | --- | --- |
| MmugDNA.24363.1.S1_at | --- | 0.665 | 698091 | interleukin-1 receptor-associated kinase 2 | IRAK2 |
| MmugDNA.24470.1.S1_at | --- | -0.593 | --- | --- | --- |
| MmugDNA.2452.1.S1_at | --- | -0.647 | --- | --- | --- |
| MmugDNA.2453.1.S1_at | ENSMMUG00000012145 | 0.621 | --- | --- | --- |
| MmugDNA.24639.1.S1_at | --- | -0.903 | --- | --- | --- |
| MmugDNA.24747.1.S1_at | ENSMMUG00000002191 | 0.612 | 717596 | similar to oxidised low density lipoprotein (lectin-like) receptor 1 | LOC717596 |
| MmugDNA.2477.1.S1_at | ENSMMUG00000010641 /// ENSMMUG00000029029 /// ENSMMUG00000029555 /// ENSMMUG00000029875 /// ENSMMUG00000030209 | 1.041 | 100187576 /// 699243 /// 711832 /// 721022 /// 723284 | similar to HLA class I histocompatibility antigen, B-38 alpha chain precursor (MHC class I antigen B*38) (Bw-4) /// similar to HLA class I histocompatibility antigen, alpha chain H precursor (HLA-AR) (HLA-12.4) /// MHC class I antigen /// major histocompatibility complex, class I, A /// major histocompatibility complex, class I, AG | LOC721022 /// LOC723284 /// MAMU-A /// MAMU-A /// MAMU-AG |
| MmugDNA.2477.1.S1_x_at | ENSMMUG00000010641 /// ENSMMUG00000029029 /// ENSMMUG00000029555 /// ENSMMUG00000029875 /// ENSMMUG00000030209 | 0.915 | 100187576 /// 699243 /// 711832 /// 721022 /// 723284 | similar to HLA class I histocompatibility antigen, B-38 alpha chain precursor (MHC class I antigen B*38) (Bw-4) /// similar to HLA class I histocompatibility antigen, alpha chain H precursor (HLA-AR) (HLA-12.4) /// MHC class I antigen /// major histocompatibility complex, class I, A /// major histocompatibility complex, class I, AG | LOC721022 /// LOC723284 /// MAMU-A /// MAMU-A /// MAMU-AG |
| MmugDNA.2478.1.S1_at | --- | 1.375 | --- | --- | --- |
| MmugDNA.24795.1.S1_at | --- | 0.610 | --- | --- | --- |
| MmugDNA.24863.1.S1_at | --- | 1.351 | 708080 | similar to Cathepsin S precursor | LOC708080 |
| MmugDNA.24887.1.S1_at | --- | -0.670 | 696517 | tripartite motif-containing 2 | TRIM2 |
| MmugDNA.24916.1.S1_at | ENSMMUG00000021676 | -0.598 | 710319 | SVOP-like | SVOPL |
| MmugDNA.24921.1.S1_at | --- | -0.649 | --- | --- | --- |
| MmugDNA.25012.1.S1_at | --- | -0.614 | 696659 | hypothetical protein LOC696659 | LOC696659 |
| MmugDNA.25042.1.S1_at | ENSMMUG00000018717 | -0.594 | 709436 | similar to zinc finger protein 75 | LOC709436 |
| MmugDNA.25072.1.S1_at | --- | -0.628 | 705692 | wingless-type MMTV integration site family, member 5A | WNT5A |
| MmugDNA.25080.1.S1_at | ENSMMUG00000001618 | 0.908 | 693838 | ATPase, H+ transporting, lysosomal 38kDa, V0 subunit d2 | ATP6V0D2 |
| MmugDNA.25081.1.S1_at | --- | 0.597 | 693838 | ATPase, H+ transporting, lysosomal 38kDa, V0 subunit d2 | ATP6V0D2 |
| MmugDNA.25113.1.S1_at | --- | -0.646 | --- | --- | --- |
| MmugDNA.25123.1.S1_s_at | ENSMMUG00000019511 | 1.426 | 706466 | ribonuclease, RNase A family, 2 (liver, eosinophil-derived neurotoxin) | RNASE2 |
| MmugDNA.25145.1.S1_at | --- | -0.588 | --- | --- | --- |
| MmugDNA.2538.1.S1_at | --- | -0.635 | 696006 | similar to transcriptional regulating factor 1 isoform 1 | LOC696006 |
| MmugDNA.25411.1.S1_at | --- | 0.703 | --- | --- | --- |
| MmugDNA.25492.1.S1_s_at | --- | 0.802 | 719412 | similar to Probable DNA dC->dU-editing enzyme APOBEC-3A (Phorbolin-1) | LOC719412 |
| MmugDNA.25557.1.S1_at | --- | -0.623 | --- | --- | --- |
| MmugDNA.25594.1.S1_at | --- | 0.960 | 702929 | phosphatidylinositol-4-phosphate 3-kinase C2 domain-containing subunit gamma-like | PIK3C2G |
| MmugDNA.25657.1.S1_at | ENSMMUG00000005668 | 1.624 | --- | --- | --- |
| MmugDNA.25657.1.S1_s_at | ENSMMUG00000005668 | 1.824 | --- | --- | --- |
| MmugDNA.25729.1.S1_s_at | --- | 0.634 | --- | --- | --- |
| MmugDNA.25927.1.S1_at | ENSMMUG00000015442 | -0.653 | 715357 | similar to amisyn | LOC715357 |
| MmugDNA.26082.1.S1_at | ENSMMUG00000019926 | 0.652 | 704141 | cytoplasmic polyadenylation element binding protein 4 | CPEB4 |
| MmugDNA.26170.1.S1_at | --- | 0.596 | 705788 | inducible T-cell co-stimulator | ICOS |
| MmugDNA.26711.1.S1_at | --- | -0.615 | --- | --- | --- |
| MmugDNA.26746.1.S1_at | ENSMMUG00000012939 | 0.652 | 722120 | similar to UNC-112 related protein 2 short form | LOC722120 |
| MmugDNA.2677.1.S1_at | --- | -0.593 | --- | --- | --- |
| MmugDNA.269.1.S1_at | --- | 0.741 | --- | --- | --- |
| MmugDNA.26925.1.S1_s_at | ENSMMUG00000004512 | 0.956 | 715814 | Similar to 40S ribosomal protein S3a (V-fos transformation effector protein) | LOC715814 |
| MmugDNA.27037.1.S1_at | ENSMMUG00000020177 | 1.101 | 698027 | membrane-spanning 4-domains, subfamily A, member 4 | MS4A4A |
| MmugDNA.27072.1.S1_at | --- | -0.684 | --- | --- | --- |
| MmugDNA.27079.1.S1_at | --- | -0.682 | 709226 | Similar to fucokinase | LOC709226 |
| MmugDNA.27318.1.S1_at | ENSMMUG00000005726 | 0.586 | 716050 | similar to small inducible cytokine A24 precursor | LOC716050 |
| MmugDNA.27361.1.S1_at | --- | 1.682 | --- | --- | --- |
| MmugDNA.27370.1.S1_s_at | ENSMMUG00000010159 | -0.611 | 696199 | AKNA domain containing 1 | AKNAD1 |
| MmugDNA.27398.1.S1_at | --- | -0.617 | --- | --- | --- |
| MmugDNA.27443.1.S1_at | ENSMMUG00000021180 | 0.823 | 719466 | growth differentiation factor 15 | GDF15 |
| MmugDNA.27513.1.S1_at | --- | -0.639 | 719145 | zinc finger protein 82 homolog (mouse) | ZFP82 |
| MmugDNA.27574.1.S1_at | ENSMMUG00000013530 | 1.146 | 697689 | membrane-spanning 4-domains, subfamily A, member 6A | MS4A6A |
| MmugDNA.27636.1.S1_at | ENSMMUG00000004821 | 1.189 | 697200 | membrane-spanning 4-domains, subfamily A, member 7 | MS4A7 |
| MmugDNA.27740.1.S1_at | --- | 0.893 | --- | --- | --- |
| MmugDNA.27825.1.S1_at | --- | -0.654 | 708287 | collagen, type IV, alpha 4 | COL4A4 |
| MmugDNA.27848.1.S1_at | ENSMMUG00000000016 | -0.940 | 713580 | adenylosuccinate synthase | ADSS |
| MmugDNA.2786.1.S1_at | --- | 0.682 | --- | --- | --- |
| MmugDNA.28072.1.S1_at | ENSMMUG00000022139 | 0.773 | 701346 | Similar to 60 kDa heat shock protein, mitochondrial precursor (Hsp60) (60 kDa chaperonin) (CPN60) (Heat shock protein 60) (HSP-60) (Mitochondrial matrix protein P1) (P60 lymphocyte protein) (HuCHA60) | LOC701346 |
| MmugDNA.28074.1.S1_at | ENSMMUG00000004907 | 0.832 | 696345 | Similar to membrane-spanning 4-domains, subfamily A, member 8B | LOC696345 |
| MmugDNA.28111.1.S1_at | --- | 0.981 | 706725 | similar to ADP-ribosylation factor-like 11 | LOC706725 |
| MmugDNA.28179.1.S1_at | --- | -0.756 | --- | --- | --- |
| MmugDNA.28205.1.S1_at | ENSMMUG00000012609 | -0.863 | --- | --- | --- |
| MmugDNA.28232.1.S1_at | --- | -0.642 | 706296 | Similar to Sec5 protein | LOC706296 |
| MmugDNA.28381.1.S1_at | --- | -0.720 | --- | --- | --- |
| MmugDNA.2850.1.S1_at | --- | 0.866 | 721040 | Similar to gene trap ROSA b-geo 22 | LOC721040 |
| MmugDNA.28598.1.S1_at | ENSMMUG00000001780 | -0.635 | --- | --- | --- |
| MmugDNA.28654.1.S1_at | --- | -0.675 | 709661 | immunoglobulin superfamily member 10-like | LOC709661 |
| MmugDNA.28691.1.S1_at | ENSMMUG00000018069 | -0.733 | --- | --- | --- |
| MmugDNA.28711.1.S1_at | ENSMMUG00000021218 | 0.667 | 717889 | Similar to LIM domain containing 2 | LOC717889 |
| MmugDNA.28734.1.S1_at | ENSMMUG00000001975 | -0.616 | --- | --- | --- |
| MmugDNA.28763.1.S1_at | --- | -0.693 | 704853 | Similar to C32A3.3a | LOC704853 |
| MmugDNA.28800.1.S1_at | ENSMMUG00000009344 | -0.784 | 707983 | SRY (sex determining region Y)-box 11 | SOX11 |
| MmugDNA.28808.1.S1_at | ENSMMUG00000000009 | 1.075 | 712657 | similar to protein tyrosine phosphatase, receptor type, C isoform 1 precursor | LOC712657 |
| MmugDNA.28887.1.S1_at | --- | 1.629 | --- | --- | --- |
| MmugDNA.2896.1.S1_at | --- | 0.649 | --- | --- | --- |
| MmugDNA.29165.1.S1_s_at | ENSMMUG00000013530 | 1.240 | 697689 | membrane-spanning 4-domains, subfamily A, member 6A | MS4A6A |
| MmugDNA.29180.1.S1_at | --- | -0.623 | --- | --- | --- |
| MmugDNA.29213.1.S1_s_at | ENSMMUG00000029923 | 0.637 | 696629 | similar to Histone H2A type 1-C | LOC696629 |
| MmugDNA.29342.1.S1_at | --- | -0.812 | --- | --- | --- |
| MmugDNA.29473.1.S1_at | ENSMMUG00000020695 | 0.599 | 702089 | SAM and SH3 domain containing 3 | SASH3 |
| MmugDNA.29522.1.S1_at | --- | 0.605 | --- | --- | --- |
| MmugDNA.29568.1.S1_at | --- | -0.604 | --- | --- | --- |
| MmugDNA.29574.1.S1_at | --- | -0.591 | 707870 | similar to insulin receptor substrate 1 | IRS4 |
| MmugDNA.29585.1.S1_at | ENSMMUG00000033584 | 1.147 | 719127 | sialic acid binding Ig-like lectin 11 | SIGLEC11 |
| MmugDNA.29694.1.S1_at | --- | -0.879 | --- | --- | --- |
| MmugDNA.29694.1.S1_s_at | --- | -0.661 | --- | --- | --- |
| MmugDNA.29742.1.S1_s_at | ENSMMUG00000005150 | 1.199 | 715410 | similar to CD163 antigen isoform a | LOC715410 |
| MmugDNA.29745.1.S1_at | --- | -0.713 | --- | --- | --- |
| MmugDNA.29758.1.S1_at | ENSMMUG00000004710 | -0.949 | 709846 | similar to proline-rich cyclin A1-interacting protein | LOC709846 |
| MmugDNA.29804.1.S1_at | --- | 0.999 | 696291 | hypothetical protein LOC696291 | LOC696291 |
| MmugDNA.29935.1.S1_at | ENSMMUG00000006705 | -0.745 | 705707 | FRY-like | FRYL |
| MmugDNA.29937.1.S1_at | --- | 0.708 | 695994 | ATPase, aminophospholipid transporter, class I, type 8B, member 1 | ATP8B1 |
| MmugDNA.29937.1.S1_x_at | --- | 0.659 | 695994 | ATPase, aminophospholipid transporter, class I, type 8B, member 1 | ATP8B1 |
| MmugDNA.30019.1.S1_at | ENSMMUG00000006269 | 1.681 | 714547 | cystatin A (stefin A) | CSTA |
| MmugDNA.30026.1.S1_at | ENSMMUG00000015790 | 0.635 | 702941 | lymphatic vessel endothelial hyaluronan receptor 1 | LYVE1 |
| MmugDNA.30119.1.S1_at | --- | -0.660 | --- | --- | --- |
| MmugDNA.30133.1.S1_at | --- | -0.755 | --- | --- | --- |
| MmugDNA.30161.1.S1_at | --- | -0.721 | --- | --- | --- |
| MmugDNA.30221.1.S1_at | --- | -0.651 | 707870 | similar to insulin receptor substrate 1 | IRS4 |
| MmugDNA.30223.1.S1_at | --- | -0.686 | --- | --- | --- |
| MmugDNA.30231.1.S1_at | --- | -0.666 | --- | --- | --- |
| MmugDNA.30350.1.S1_at | --- | 1.007 | 719792 | formyl peptide receptor 2 | FPR2 |
| MmugDNA.30583.1.S1_at | --- | 1.288 | --- | --- | --- |
| MmugDNA.30706.1.S1_at | ENSMMUG00000010579 | -0.642 | --- | --- | --- |
| MmugDNA.30721.1.S1_at | ENSMMUG00000013945 | 1.656 | 710385 | similar to cytochrome P450, family 1, subfamily B, polypeptide 1 | LOC710385 |
| MmugDNA.30998.1.S1_at | ENSMMUG00000017683 | -0.739 | 707447 | actin, gamma 2, smooth muscle, enteric | ACTG2 |
| MmugDNA.31020.1.S1_at | --- | -1.012 | 701079 | zinc binding alcohol dehydrogenase domain containing 2 | ZADH2 |
| MmugDNA.31066.1.S1_at | ENSMMUG00000008504 | -0.600 | 715714 | bromodomain and WD repeat domain containing 1 | BRWD1 |
| MmugDNA.31072.1.S1_at | --- | 0.966 | --- | --- | --- |
| MmugDNA.31114.1.S1_at | --- | -0.675 | --- | --- | --- |
| MmugDNA.31189.1.S1_at | --- | -0.588 | --- | --- | --- |
| MmugDNA.31218.1.S1_at | --- | 1.548 | 692336 | leukocyte immunoglobulin-like receptor, subfamily B, member c | LILRBC |
| MmugDNA.3132.1.S1_at | --- | 1.024 | --- | --- | --- |
| MmugDNA.31375.1.S1_s_at | ENSMMUG00000009852 | 0.699 | 706568 | caspase 1, apoptosis-related cysteine peptidase (interleukin 1, beta, convertase) | CASP1 |
| MmugDNA.31377.1.S1_s_at | ENSMMUG00000009852 | 0.642 | 706568 | caspase 1, apoptosis-related cysteine peptidase (interleukin 1, beta, convertase) | CASP1 |
| MmugDNA.31465.1.S1_at | --- | 1.180 | --- | --- | --- |
| MmugDNA.31473.1.S1_at | --- | -0.682 | 710731 | palmdelphin | PALMD |
| MmugDNA.31663.1.S1_at | --- | -0.795 | --- | --- | --- |
| MmugDNA.31741.1.S1_at | ENSMMUG00000018423 | 0.611 | 697082 | BCL2-like 11 (apoptosis facilitator) | BCL2L11 |
| MmugDNA.31751.1.S1_s_at | ENSMMUG00000012797 | -0.837 | 713398 | zinc finger protein 233-like | LOC713398 |
| MmugDNA.31841.1.S1_at | ENSMMUG00000008040 | 1.393 | 693917 | tumor necrosis factor (ligand) superfamily, member 13b | TNFSF13B |
| MmugDNA.31943.1.S1_at | --- | -0.591 | --- | --- | --- |
| MmugDNA.31947.1.S1_at | --- | 0.606 | 695451 | Similar to myopalladin | LOC695451 |
| MmugDNA.3204.1.S1_at | --- | 0.675 | --- | --- | --- |
| MmugDNA.321.1.S1_at | --- | 0.915 | --- | --- | --- |
| MmugDNA.32138.1.S1_at | --- | 0.983 | 712125 | similar to zinc transporter like 2 | LOC712125 |
| MmugDNA.32153.1.S1_at | --- | 0.925 | --- | --- | --- |
| MmugDNA.32465.1.S1_at | --- | -0.645 | --- | --- | --- |
| MmugDNA.32522.1.S1_at | ENSMMUG00000017127 | 0.700 | 700690 | carbohydrate (chondroitin 4) sulfotransferase 11 | CHST11 |
| MmugDNA.32538.1.S1_at | ENSMMUG00000008603 | 0.733 | 712972 | ecotropic viral integration site 2B | EVI2B |
| MmugDNA.32570.1.S1_at | --- | 1.037 | --- | --- | --- |
| MmugDNA.32691.1.S1_at | --- | -0.720 | --- | --- | --- |
| MmugDNA.32699.1.S1_at | ENSMMUG00000000912 | 1.012 | 714390 | CD86 molecule | CD86 |
| MmugDNA.32736.1.S1_at | --- | -0.628 | --- | --- | --- |
| MmugDNA.32865.1.S1_at | ENSMMUG00000002457 | 0.609 | 702568 | DENN/MADD domain containing 2D | DENND2D |
| MmugDNA.32962.1.S1_at | --- | -0.635 | --- | --- | --- |
| MmugDNA.33042.1.S1_at | --- | -0.786 | 699435 | similar to latent transforming growth factor beta binding protein 2 | LTBP1 |
| MmugDNA.33060.1.S1_at | --- | 1.056 | 692340 | leukocyte immunoglobulin-like receptor, subfamily B, member a | LILRBA |
| MmugDNA.33060.1.S1_x_at | ENSMMUG00000013289 | 0.884 | 692339 /// 692340 /// 696677 | leukocyte immunoglobulin-like receptor, subfamily B, member a /// leukocyte immunoglobulin-like receptor, subfamily B, member b /// hypothetical protein LOC696677 | LILRBA /// LILRBB /// LOC696677 |
| MmugDNA.33127.1.S1_at | --- | -0.593 | --- | --- | --- |
| MmugDNA.33174.1.S1_at | --- | -0.599 | 702680 | Similar to T-cell receptor beta chain V region C5 precursor | LOC702680 |
| MmugDNA.3325.1.S1_at | --- | -0.591 | 705975 | cyclin-dependent kinase-like 1 (CDC2-related kinase) | CDKL1 |
| MmugDNA.33453.1.S1_at | --- | -0.926 | --- | --- | --- |
| MmugDNA.3352.1.S1_s_at | ENSMMUG00000001461 | -0.595 | --- | --- | --- |
| MmugDNA.33529.1.S1_at | --- | 0.633 | 719965 | similar to HLA class I histocompatibility antigen, Cw-4 alpha chain precursor (MHC class I antigen Cw*4) | LOC719965 |
| MmugDNA.33563.1.S1_at | ENSMMUG00000031509 | 1.147 | 693768 | similar to histone 2a | LOC693768 |
| MmugDNA.33563.1.S1_s_at | ENSMMUG00000031509 | 1.106 | 693768 | similar to histone 2a | LOC693768 |
| MmugDNA.33605.1.S1_at | --- | 0.770 | --- | --- | --- |
| MmugDNA.33626.1.S1_at | --- | 0.737 | 693936 | IKAROS family zinc finger 1 (Ikaros) | IKZF1 |
| MmugDNA.3370.1.S1_at | ENSMMUG00000017988 | -0.661 | 695655 | Nucleotide-binding oligomerization domain containing 1 | NOD1 |
| MmugDNA.33751.1.S1_at | --- | -0.627 | 698839 | RAS, dexamethasone-induced 1 | RASD1 |
| MmugDNA.34019.1.S1_at | --- | 1.045 | 705927 | similar to lung-inducible neuralized-related C3HC4 RING domain protein | LOC705927 |
| MmugDNA.34026.1.S1_at | ENSMMUG00000014293 | 0.844 | 718510 | phospholipase A2, group IVC (cytosolic, calcium-independent) | PLA2G4C |
| MmugDNA.34086.1.S1_at | --- | -0.610 | --- | --- | --- |
| MmugDNA.34119.1.S1_at | ENSMMUG00000002737 | 0.658 | 694856 | fibrinogen-like 2 | FGL2 |
| MmugDNA.34136.1.S1_at | ENSMMUG00000028568 /// ENSMMUG00000032691 | 1.389 | 1E+08 | microRNA mir-147b | MIR147B |
| MmugDNA.34226.1.S1_at | ENSMMUG00000020177 | 1.295 | 698027 | membrane-spanning 4-domains, subfamily A, member 4 | MS4A4A |
| MmugDNA.34277.1.S1_at | --- | -0.742 | --- | --- | --- |
| MmugDNA.34347.1.S1_s_at | --- | 0.638 | 699911 | solute carrier family 25, member 13 (citrin) | SLC25A13 |
| MmugDNA.34401.1.S1_at | --- | -0.622 | --- | --- | --- |
| MmugDNA.34685.1.S1_at | --- | -1.180 | --- | --- | --- |
| MmugDNA.34787.1.S1_at | ENSMMUG00000004362 | 1.443 | 705855 | HtrA serine peptidase 1 | HTRA1 |
| MmugDNA.34863.1.S1_at | --- | -0.608 | --- | --- | --- |
| MmugDNA.35059.1.S1_at | ENSMMUG00000013098 | -0.672 | 706528 | similar to aldo-keto reductase family 1, member B10 | LOC706528 |
| MmugDNA.35142.1.S1_at | --- | -1.002 | --- | --- | --- |
| MmugDNA.35261.1.S1_at | --- | 0.926 | --- | --- | --- |
| MmugDNA.35326.1.S1_at | --- | -0.593 | --- | --- | --- |
| MmugDNA.35499.1.S1_at | --- | -0.609 | 717484 | similar to MTERF domain containing 2 | LOC717484 |
| MmugDNA.35730.1.S1_at | ENSMMUG00000023771 | 1.471 | 694064 | SAM domain, SH3 domain and nuclear localization signals 1 | SAMSN1 |
| MmugDNA.35764.1.S1_s_at | ENSMMUG00000004512 | 0.980 | 715814 | Similar to 40S ribosomal protein S3a (V-fos transformation effector protein) | LOC715814 |
| MmugDNA.35771.1.S1_at | --- | -0.731 | --- | --- | --- |
| MmugDNA.35788.1.S1_at | --- | -0.612 | --- | --- | --- |
| MmugDNA.35854.1.S1_at | ENSMMUG00000020756 | 0.774 | 708589 | complement component 1, q subcomponent, C chain | C1QC |
| MmugDNA.35997.1.S1_at | --- | -0.774 | --- | --- | --- |
| MmugDNA.36047.1.S1_at | ENSMMUG00000011301 | 0.861 | 717766 | similar to proteasome beta 8 subunit isoform E2 proprotein | LOC717766 |
| MmugDNA.36075.1.S1_at | ENSMMUG00000018680 | -1.150 | 702126 | potassium voltage-gated channel, shaker-related subfamily, member 2 | KCNA2 |
| MmugDNA.36.1.S1_at | ENSMMUG00000023230 | 1.204 | --- | --- | --- |
| MmugDNA.36286.1.S1_at | --- | 1.787 | 710385 | similar to cytochrome P450, family 1, subfamily B, polypeptide 1 | LOC710385 |
| MmugDNA.36308.1.S1_at | --- | -0.780 | --- | --- | --- |
| MmugDNA.36389.1.S1_at | ENSMMUG00000005545 | 0.704 | 717994 | similar to oncostatin M precursor | LOC717994 |
| MmugDNA.36433.1.S1_at | ENSMMUG00000000663 | 1.142 | 694684 | guanylate binding protein 2, interferon-inducible | GBP2 |
| MmugDNA.36459.1.S1_at | ENSMMUG00000013220 | 1.526 | 696542 | cytochrome b-245, beta polypeptide (chronic granulomatous disease) | CYBB |
| MmugDNA.36497.1.S1_at | ENSMMUG00000015749 | 0.602 | 713699 | sulfide quinone reductase-like (yeast) | SQRDL |
| MmugDNA.36535.1.S1_at | --- | -0.658 | 709837 | carnitine palmitoyltransferase 1A | CPT1A |
| MmugDNA.36555.1.S1_at | ENSMMUG00000002426 | -0.754 | 707293 | similar to myosin light chain 2a | LOC707293 |
| MmugDNA.36812.1.S1_at | ENSMMUG00000012389 | -0.611 | --- | --- | --- |
| MmugDNA.36919.1.S1_s_at | ENSMMUG00000023771 | 1.322 | 694064 | SAM domain, SH3 domain and nuclear localization signals 1 | SAMSN1 |
| MmugDNA.37021.1.S1_at | --- | -0.694 | --- | --- | --- |
| MmugDNA.37154.1.S1_at | ENSMMUG00000019371 | 1.377 | 717992 | major histocompatibility complex, class II, DP alpha | MAMU-DPA |
| MmugDNA.37257.1.S1_at | ENSMMUG00000006030 | 0.851 | 705403 | ArfGAP with coiled-coil, ankyrin repeat and PH domains 2 | ACAP2 |
| MmugDNA.37303.1.S1_at | --- | -0.636 | 706403 | Similar to ubiquitin specific protease 16 isoform a | LOC706403 |
| MmugDNA.37382.1.S1_s_at | --- | 0.686 | 706963 | Similar to profilin 1 | LOC706963 |
| MmugDNA.37601.1.S1_at | --- | 0.614 | 699584 | stomatin | STOM |
| MmugDNA.3760.1.S1_at | ENSMMUG00000002192 | 0.621 | 574356 | C-type lectin domain family 7, member A | CLEC7A |
| MmugDNA.37612.1.S1_at | ENSMMUG00000003386 | -0.749 | 704379 | leucine rich repeat (in FLII) interacting protein 1 | LRRFIP1 |
| MmugDNA.37862.1.S1_at | --- | -0.593 | --- | --- | --- |
| MmugDNA.3789.1.S1_at | --- | -0.608 | 698892 | zinc finger protein, X-linked | ZFX |
| MmugDNA.37986.1.S1_at | --- | 0.924 | --- | --- | --- |
| MmugDNA.38074.1.S1_at | --- | -0.590 | --- | --- | --- |
| MmugDNA.38175.1.S1_at | ENSMMUG00000015153 | -0.718 | --- | --- | --- |
| MmugDNA.38210.1.S1_at | --- | -0.640 | --- | --- | --- |
| MmugDNA.38493.1.S1_at | --- | -0.612 | --- | --- | --- |
| MmugDNA.38507.1.S1_at | --- | -0.605 | --- | --- | --- |
| MmugDNA.38653.1.S1_at | --- | -0.812 | --- | --- | --- |
| MmugDNA.38680.1.S1_at | --- | -0.647 | --- | --- | --- |
| MmugDNA.39068.1.S1_at | ENSMMUG00000008407 | 0.895 | 704086 | sodium channel, voltage-gated, type I, alpha | SCN1A |
| MmugDNA.39143.1.S1_at | --- | -0.858 | --- | --- | --- |
| MmugDNA.39349.1.S1_at | --- | -0.809 | --- | --- | --- |
| MmugDNA.39351.1.S1_at | --- | -0.883 | --- | --- | --- |
| MmugDNA.39580.1.S1_at | ENSMMUG00000014468 | -0.680 | 712074 | EPH receptor A5 | EPHA5 |
| MmugDNA.39862.1.S1_at | ENSMMUG00000022101 | -0.642 | 706175 | similar to cold inducible RNA binding protein | LOC706175 |
| MmugDNA.39931.1.S1_at | --- | -0.717 | --- | --- | --- |
| MmugDNA.39989.1.S1_at | --- | -0.934 | --- | --- | --- |
| MmugDNA.40138.1.S1_at | ENSMMUG00000008869 | 0.715 | 704130 | tumor necrosis factor, alpha-induced protein 3 | TNFAIP3 |
| MmugDNA.40170.1.S1_s_at | ENSMMUG00000021483 | 1.927 | 711020 | ubiquitin D | UBD |
| MmugDNA.4028.1.S1_s_at | ENSMMUG00000003364 | 1.983 | 717493 | hypothetical protein LOC717493 | LOC717493 |
| MmugDNA.40463.1.S1_at | --- | -0.591 | --- | --- | --- |
| MmugDNA.40478.1.S1_at | --- | -0.639 | 698581 | transmembrane protein 87B | TMEM87B |
| MmugDNA.40598.1.S1_at | ENSMMUG00000001300 | 0.971 | 719284 | similar to Duffy blood group | LOC719284 |
| MmugDNA.40619.1.S1_at | ENSMMUG00000014338 | 0.809 | --- | --- | --- |
| MmugDNA.40734.1.S1_at | ENSMMUG00000007027 | -0.607 | --- | --- | --- |
| MmugDNA.40764.1.S1_at | ENSMMUG00000017595 | 1.005 | --- | --- | --- |
| MmugDNA.40768.1.S1_at | --- | 0.703 | 706285 | hypothetical protein LOC706285 | LOC706285 |
| MmugDNA.40887.1.S1_at | --- | -0.654 | --- | --- | --- |
| MmugDNA.40903.1.S1_at | --- | -0.643 | --- | --- | --- |
| MmugDNA.40949.1.S1_at | --- | -0.619 | --- | --- | --- |
| MmugDNA.41068.1.S1_at | --- | -0.632 | --- | --- | --- |
| MmugDNA.41148.1.S1_at | --- | 0.617 | 705946 | similar to T-cell receptor interacting molecule | LOC705946 |
| MmugDNA.41315.1.S1_at | ENSMMUG00000000662 | 1.402 | 694538 | guanylate binding protein 1, interferon-inducible, 67kD | GBP1 |
| MmugDNA.41384.1.S1_at | --- | 0.737 | --- | --- | --- |
| MmugDNA.41431.1.S1_at | --- | -0.887 | --- | --- | --- |
| MmugDNA.41520.1.S1_at | --- | -0.660 | --- | --- | --- |
| MmugDNA.41641.1.S1_at | ENSMMUG00000013530 | 1.044 | 697689 | membrane-spanning 4-domains, subfamily A, member 6A | MS4A6A |
| MmugDNA.41937.1.S1_at | --- | -0.672 | --- | --- | --- |
| MmugDNA.42098.1.S1_at | --- | 0.623 | 713244 | similar to AP-1 complex subunit sigma-2 | LOC713244 |
| MmugDNA.42128.1.S1_at | ENSMMUG00000004196 | -0.655 | 697003 | chromosome 4 open reading frame 49 ortholog | C5H4orf49 |
| MmugDNA.42156.1.S1_at | --- | 0.694 | --- | --- | --- |
| MmugDNA.42461.1.S1_at | --- | 0.903 | --- | --- | --- |
| MmugDNA.42478.1.S1_at | --- | -0.709 | --- | --- | --- |
| MmugDNA.42543.1.S1_at | ENSMMUG00000006005 | -0.671 | 698567 | similar to Cerebellin-4 precursor (Cerebellin-like glycoprotein 1) | LOC698567 |
| MmugDNA.42599.1.S1_at | ENSMMUG00000001155 | 0.719 | 696404 | similar to acyl-CoA synthetase long-chain family member 5 isoform a | LOC696404 |
| MmugDNA.42707.1.S1_at | --- | -0.947 | --- | --- | --- |
| MmugDNA.42727.1.S1_at | ENSMMUG00000000637 | -0.748 | 699558 | SH3-domain GRB2-like (endophilin) interacting protein 1 | SGIP1 |
| MmugDNA.42759.1.S1_at | ENSMMUG00000015485 | -0.624 | 705425 | MOCO sulphurase C-terminal domain containing 1 | MOSC1 |
| MmugDNA.42764.1.S1_at | ENSMMUG00000016880 | 1.013 | 710142 | zinc transporter ZIP8-like | LOC710142 |
| MmugDNA.42764.1.S1_s_at | ENSMMUG00000016880 | 0.726 | 710142 | zinc transporter ZIP8-like | LOC710142 |
| MmugDNA.42846.1.S1_at | --- | -0.688 | --- | --- | --- |
| MmugDNA.42905.1.S1_at | ENSMMUG00000017244 | 0.667 | 709960 | similar to G protein-coupled receptor 171 | LOC709960 |
| MmugDNA.42953.1.S1_at | --- | -1.006 | --- | --- | --- |
| MmugDNA.42997.1.S1_at | --- | 0.625 | --- | --- | --- |
| MmugDNA.43111.1.S1_at | --- | 0.831 | --- | --- | --- |
| MmugDNA.43116.1.S1_at | ENSMMUG00000012648 | 1.475 | 704990 | glycoprotein (transmembrane) nmb | GPNMB |
| MmugDNA.43193.1.S1_at | --- | -0.907 | --- | --- | --- |
| MmugDNA.43273.1.S1_at | ENSMMUG00000002455 | 1.053 | 705024 | chitinase 3-like 2 | CHI3L2 |
| MmugDNA.43306.1.S1_at | --- | -0.694 | 694405 | Similar to cyclin I | LOC694405 |
| MmugDNA.43358.1.S1_at | --- | -1.210 | --- | --- | --- |
| MmugDNA.43376.1.S1_s_at | ENSMMUG00000004823 | 0.977 | 574178 | chemokine (C-C motif) ligand 5 | CCL5 |
| MmugDNA.43524.1.S1_at | --- | -0.701 | --- | --- | --- |
| MmugDNA.43538.1.S1_at | --- | 0.706 | 697284 | hypothetical protein LOC697284 | LOC697284 |
| MmugDNA.4404.1.S1_at | ENSMMUG00000007329 | 0.651 | 710813 | coronin, actin binding protein, 1A | CORO1A |
| MmugDNA.4710.1.S1_at | ENSMMUG00000012837 | 0.655 | 702295 | Wilms tumor 1 associated protein | WTAP |
| MmugDNA.4868.1.S1_at | ENSMMUG00000014126 | 1.056 | 700374 | lymphocyte cytosolic protein 2 | LCP2 |
| MmugDNA.4869.1.S1_s_at | ENSMMUG00000008160 | 0.921 | 695612 | similar to neutrophil cytosolic factor 4 (40kD) isoform 1 | LOC695612 |
| MmugDNA.4871.1.S1_at | --- | 1.011 | 695612 | similar to neutrophil cytosolic factor 4 (40kD) isoform 1 | LOC695612 |
| MmugDNA.4874.1.S1_at | ENSMMUG00000004492 | 0.946 | 719260 | absent in melanoma 2 | AIM2 |
| MmugDNA.4991.1.S1_at | --- | -0.705 | 706237 | thiamin pyrophosphokinase 1 | TPK1 |
| MmugDNA.5.1.S1_at | --- | -0.591 | --- | --- | --- |
| MmugDNA.5252.1.S1_at | --- | 1.447 | --- | --- | --- |
| MmugDNA.5420.1.S1_at | --- | -0.695 | --- | --- | --- |
| MmugDNA.5445.1.S1_at | --- | 0.732 | 716656 | similar to family with sequence similarity 19 (chemokine (C-C motif)-like), member A2 | LOC716656 |
| MmugDNA.5541.1.S1_at | ENSMMUG00000015250 | 1.481 | 716122 | C-type lectin domain family 4, member A | CLEC4A |
| MmugDNA.559.1.S1_at | --- | -0.644 | 705416 | similar to KH domain containing, RNA binding, signal transduction associated 3 | LOC705416 |
| MmugDNA.565.1.S1_at | --- | 0.604 | --- | --- | --- |
| MmugDNA.5658.1.S1_at | ENSMMUG00000003364 | 2.272 | 712571 | BCL2-related protein A1 | BCL2A1 |
| MmugDNA.5818.1.S1_at | ENSMMUG00000009231 | -0.759 | 697551 | Na+/K+ transporting ATPase interacting 4 | NKAIN4 |
| MmugDNA.5827.1.S1_at | ENSMMUG00000014126 | 1.382 | 700374 | lymphocyte cytosolic protein 2 | LCP2 |
| MmugDNA.592.1.S1_at | --- | -0.710 | --- | --- | --- |
| MmugDNA.6142.1.S1_at | --- | 0.694 | --- | --- | --- |
| MmugDNA.616.1.S1_at | ENSMMUG00000000940 | 1.010 | 574181 | chemokine (C-C motif) ligand 18 (pulmonary and activation-regulated) | CCL18 |
| MmugDNA.6192.1.S1_at | --- | 0.832 | --- | --- | --- |
| MmugDNA.629.1.S1_at | --- | -1.254 | --- | --- | --- |
| MmugDNA.6347.1.S1_at | --- | 0.783 | --- | --- | --- |
| MmugDNA.635.1.S1_at | --- | 1.112 | 693917 | tumor necrosis factor (ligand) superfamily, member 13b | TNFSF13B |
| MmugDNA.6381.1.S1_at | ENSMMUG00000005757 | 0.684 | 704850 | cystatin F (leukocystatin) | CST7 |
| MmugDNA.6382.1.S1_at | ENSMMUG00000003398 | 1.326 | 702468 | baculoviral IAP repeat-containing 3 | BIRC3 |
| MmugDNA.6394.1.S1_at | ENSMMUG00000004128 | -0.750 | --- | --- | --- |
| MmugDNA.6430.1.S1_at | ENSMMUG00000020289 | 0.676 | 701825 | CD83 molecule | CD83 |
| MmugDNA.6622.1.S1_at | --- | -0.672 | --- | --- | --- |
| MmugDNA.6704.1.S1_at | ENSMMUG00000004327 | 0.894 | 702435 | Similar to protocadherin 1 isoform 2 precursor | LOC702435 |
| MmugDNA.6841.1.S1_at | --- | 0.623 | 700942 | TRAF family member-associated NFKB activator | TANK |
| MmugDNA.6875.1.S1_at | --- | 0.880 | 704667 | Similar to CG7818-PA | LOC704667 |
| MmugDNA.6895.1.S1_s_at | ENSMMUG00000015208 | 0.903 | 707984 /// 712156 | similar to Mob4B protein /// MOB1, Mps One Binder kinase activator-like 1B (yeast) | LOC707984 /// MOBKL1B |
| MmugDNA.6929.1.S1_s_at | ENSMMUG00000007586 | 0.916 | 694207 | neutrophil cytosolic factor 1 | NCF1 |
| MmugDNA.6967.1.S1_at | --- | -0.616 | --- | --- | --- |
| MmugDNA.7038.1.S1_at | --- | 1.271 | --- | --- | --- |
| MmugDNA.7052.1.S1_at | --- | 0.820 | 705561 | similar to ubiquitin-conjugating enzyme E2L 6 isoform 1 | LOC705561 |
| MmugDNA.7203.1.S1_at | ENSMMUG00000003237 | -0.610 | 714008 | Similar to mitochondrial ribosomal protein L2 | LOC714008 |
| MmugDNA.7233.1.S1_at | --- | 1.735 | --- | --- | --- |
| MmugDNA.735.1.S1_at | --- | -0.683 | 694695 | similar to taste receptor, type 2, member 48 | LOC694695 |
| MmugDNA.7359.1.S1_s_at | ENSMMUG00000008004 | 0.875 | 574207 | TYRO protein tyrosine kinase binding protein | TYROBP |
| MmugDNA.7364.1.S1_at | --- | 1.244 | --- | --- | --- |
| MmugDNA.7381.1.S1_s_at | ENSMMUG00000008853 | 0.915 | 100141390 /// 715042 | similar to Leukocyte-specific transcript 1 protein (B144 protein) /// leukocyte specific transcript 1 | LOC715042 /// LST1 |
| MmugDNA.7432.1.S1_at | --- | -0.946 | 709776 | oxoglutarate dehydrogenase-like | OGDHL |
| MmugDNA.76.1.S1_s_at | ENSMMUG00000011058 | -0.951 | 706060 | spermatogenesis associated 22 | SPATA22 |
| MmugDNA.7816.1.S1_at | ENSMMUG00000013220 | 1.645 | 696542 | cytochrome b-245, beta polypeptide (chronic granulomatous disease) | CYBB |
| MmugDNA.7822.1.S1_at | ENSMMUG00000021914 | -0.636 | --- | --- | --- |
| MmugDNA.7832.1.S1_at | --- | -0.586 | 707062 | Similar to ribonucleotide reductase M2 B (TP53 inducible) | LOC707062 |
| MmugDNA.7871.1.S1_at | --- | -0.626 | --- | --- | --- |
| MmugDNA.8062.1.S1_at | ENSMMUG00000019455 | 0.777 | 699497 | synaptotagmin-like 3 | SYTL3 |
| MmugDNA.8105.1.S1_at | --- | -0.702 | --- | --- | --- |
| MmugDNA.8126.1.S1_at | --- | 0.681 | --- | --- | --- |
| MmugDNA.8.1.S1_at | --- | -0.853 | --- | --- | --- |
| MmugDNA.8234.1.S1_at | ENSMMUG00000002387 | 0.784 | 693720 | similar to CG11594-PB, isoform B | LOC693720 |
| MmugDNA.8358.1.S1_at | --- | 0.671 | 700473 | similar to gap junction protein, chi 1, 31.9kDa (connexin 31.9) | GJD3 |
| MmugDNA.8385.1.S1_at | --- | -0.599 | --- | --- | --- |
| MmugDNA.8496.1.S1_at | ENSMMUG00000016531 | 1.544 | 574386 | chemokine (C-C motif) ligand 19 | CCL19 |
| MmugDNA.8510.1.S1_s_at | ENSMMUG00000018312 | 1.342 | 722189 | similar to Retinoic acid receptor responder protein 3 (Tazarotene-induced gene 3 protein) (RAR-responsive protein TIG3) (Retinoid-inducible gene 1 protein) | LOC722189 |
| MmugDNA.8556.1.S1_at | ENSMMUG00000008853 | 0.827 | --- | --- | --- |
| MmugDNA.8804.1.S1_at | ENSMMUG00000010706 | -0.749 | 708003 | transmembrane protein 44 | TMEM44 |
| MmugDNA.8919.1.S1_at | --- | -0.650 | --- | --- | --- |
| MmugDNA.9140.1.S1_at | --- | -0.713 | --- | --- | --- |
| MmugDNA.9153.1.S1_at | ENSMMUG00000017745 | -0.677 | 697017 | angiopoietin 1 | ANGPT1 |
| MmugDNA.9153.1.S1_s_at | ENSMMUG00000017745 | -0.735 | 697017 | angiopoietin 1 | ANGPT1 |
| MmugDNA.9703.1.S1_at | ENSMMUG00000003674 | 1.379 | 693558 | triggering receptor expressed on myeloid cells 1 | TREM1 |
| MmugDNA.9853.1.S1_at | --- | 1.085 | 707926 | arachidonate 5-lipoxygenase | ALOX5 |
| MmugDNA.9854.1.S1_at | ENSMMUG00000005572 | 1.274 | 722251 | Hypothetical protein LOC722251 | LOC722251 |
| MmugDNA.9861.1.S1_at | ENSMMUG00000020471 | -0.794 | 710012 | actinin, alpha 2 | ACTN2 |
| MmugDNA.9866.1.S1_at | --- | -0.694 | 710012 | actinin, alpha 2 | ACTN2 |
| MmugDNA.9925.1.S1_at | ENSMMUG00000015785 | 0.598 | 703186 | adenosine monophosphate deaminase 3 | AMPD3 |
| MmugDNA.9959.1.S1_at | --- | -1.567 | --- | --- | --- |
| MmunewRS.431.1.S1_at | ENSMMUG00000020241 | -0.631 | 574274 | Protein tyrosine phosphatase, receptor type, S | PTPRS |
| MmunewRS.436.1.S1_s_at | ENSMMUG00000008227 /// ENSMMUG00000029799 | 1.507 | 677701 /// 692100 /// 705588 | similar to HLA class II histocompatibility antigen, DRB1-4 beta chain precursor (MHC class I antigen DRB1*4) (DR-4) (DR4) /// MHC class II antigen /// MHC class II antigen, Mamu-DRB5 | LOC705588 /// MAMU-DRB /// MAMU-DRB5 |
| MmunewRS.536.1.S1_at | ENSMMUG00000003163 | 0.908 | 719379 | similar to Gamma-interferon-inducible lysosomal thiol reductase precursor (Gamma-interferon-inducible protein IP-30) | LOC719379 |
| MmunewRS.55.1.S1_at | ENSMMUG00000017033 | 1.185 | 703429 | lymphocyte cytosolic protein 1 (L-plastin) | LCP1 |
| MmunewRS.790.1.S1_at | ENSMMUG00000032227 | 1.166 | 711532 | major histocompatibility complex, class I, E | MAMU-E |
| MmunewRS.790.1.S1_x_at | ENSMMUG00000032227 | 1.138 | 711532 | major histocompatibility complex, class I, E | MAMU-E |
| MmunewRS.936.1.S1_at | --- | 0.807 | 695158 | similar to guanylate binding protein family, member 6 | LOC695158 |
| MmuSTS.1097.1.S1_at | ENSMMUG00000003927 | 0.646 | 714170 | similar to progestin and adipoQ receptor family member IX | LOC714170 |
| MmuSTS.1168.1.S1_at | ENSMMUG00000008711 | 0.891 | 703401 | chitinase 3-like 1 (cartilage glycoprotein-39) | CHI3L1 |
| MmuSTS.1204.1.S1_at | ENSMMUG00000015847 | 0.674 | 574290 | toll-like receptor 2 | TLR2 |
| MmuSTS.1219.1.S1_at | --- | -0.620 | 712115 | Similar to ADP-ribosylation factor guanine nucleotide factor 6 isoform a | LOC712115 |
| MmuSTS.1379.1.S1_at | ENSMMUG00000014588 | 0.719 | 707083 | basic helix-loop-helix family, member e40 | BHLHE40 |
| MmuSTS.1385.1.S1_at | --- | 0.686 | --- | --- | --- |
| MmuSTS.139.1.S1_at | ENSMMUG00000023163 | 0.885 | 695389 | bridging integrator 2 | BIN2 |
| MmuSTS.1397.1.S1_at | ENSMMUG00000020756 | 1.259 | 718307 | similar to Complement C1q subcomponent subunit B precursor | LOC718307 |
| MmuSTS.1398.1.S1_at | ENSMMUG00000012786 | 0.869 | 714589 | complement component 1, s subcomponent | C1S |
| MmuSTS.1399.1.S1_at | ENSMMUG00000000729 | 0.745 | 716809 | complement factor B | CFB |
| MmuSTS.1407.1.S1_at | --- | -0.758 | --- | --- | --- |
| MmuSTS.1437.1.S1_at | ENSMMUG00000017033 | 1.193 | 703429 | lymphocyte cytosolic protein 1 (L-plastin) | LCP1 |
| MmuSTS.1447.1.S1_at | ENSMMUG00000020721 | 0.620 | 699939 | lymphocyte antigen 75 | LY75 |
| MmuSTS.1486.1.S1_at | ENSMMUG00000011845 | 0.640 | 710479 | hexokinase 2 | HK2 |
| MmuSTS.1523.1.S1_at | ENSMMUG00000011620 | 0.785 | 701010 | mannose receptor, C type 1 | MRC1 |
| MmuSTS.1556.1.S1_at | --- | -1.147 | --- | --- | --- |
| MmuSTS.1603.1.S1_at | ENSMMUG00000017048 | -0.779 | --- | --- | --- |
| MmuSTS.1741.1.S1_at | --- | -0.668 | 697306 | myogenic factor 6 (herculin) | MYF6 |
| MmuSTS.1747.1.S1_at | --- | -0.826 | 699411 | myosin VIIA | MYO7A |
| MmuSTS.1778.1.S1_at | --- | 0.745 | 693551 | scinderin | SCIN |
| MmuSTS.1849.1.S1_at | ENSMMUG00000015298 | 1.473 | 714297 | Deltex homolog 1 | DTX1 |
| MmuSTS.1893.1.S1_s_at | ENSMMUG00000020903 | 2.035 | --- | --- | --- |
| MmuSTS.1940.1.S1_at | --- | -0.745 | 705813 | similar to Fibroblast growth factor 12 (FGF-12) (Fibroblast growth factor homologous factor 1) (FHF-1) | LOC705813 |
| MmuSTS.1957.1.S1_at | ENSMMUG00000020755 | 1.226 | 708477 | complement component 1, q subcomponent, A chain | C1QA |
| MmuSTS.1981.1.S1_at | --- | 0.761 | --- | --- | --- |
| MmuSTS.1982.1.S1_at | ENSMMUG00000010007 | 0.796 | 697482 | CD14 molecule | CD14 |
| MmuSTS.20.1.S1_at | ENSMMUG00000003840 | 1.051 | --- | --- | --- |
| MmuSTS.2023.1.S1_at | --- | 0.951 | 711746 | similar to paired immunoglobulin-like type 2 receptor alpha isoform 2 precursor | LOC711746 |
| MmuSTS.2023.1.S1_s_at | --- | 0.695 | 711746 | similar to paired immunoglobulin-like type 2 receptor alpha isoform 2 precursor | LOC711746 |
| MmuSTS.2065.1.S1_at | ENSMMUG00000014670 | 1.053 | 706853 | phosphorylase, glycogen, liver | PYGL |
| MmuSTS.2065.1.S1_x_at | ENSMMUG00000014670 | 1.010 | 706853 | phosphorylase, glycogen, liver | PYGL |
| MmuSTS.2106.1.S1_at | ENSMMUG00000000242 | -0.784 | 713213 | Hypothetical protein LOC713213 | LOC713213 |
| MmuSTS.2150.1.S1_at | ENSMMUG00000011218 | 2.375 | 574106 | serpin peptidase inhibitor, clade A (alpha-1 antiproteinase, antitrypsin), member 3 | SERPINA3 |
| MmuSTS.2164.1.S1_at | ENSMMUG00000017653 | 0.811 | 708293 | Similar to DEAD (Asp-Glu-Ala-Asp) box polypeptide 10 | LOC708293 |
| MmuSTS.2175.1.S1_at | ENSMMUG00000005889 | -0.613 | 706395 | similar to zinc finger protein 181 (HHZ181) | LOC706395 |
| MmuSTS.2193.1.S1_at | ENSMMUG00000021905 | 0.832 | 713696 | sphingomyelin phosphodiesterase, acid-like 3A | SMPDL3A |
| MmuSTS.2246.1.S1_at | ENSMMUG00000005334 | 0.622 | 714173 | phospholipase C, gamma 2 (phosphatidylinositol-specific) | PLCG2 |
| MmuSTS.2360.1.S1_at | ENSMMUG00000008145 | -0.837 | 715403 | S100 calcium binding protein A1 | S100A1 |
| MmuSTS.2397.1.S1_s_at | ENSMMUG00000000663 | 1.150 | 694684 | guanylate binding protein 2, interferon-inducible | GBP2 |
| MmuSTS.2411.1.S1_at | ENSMMUG00000015809 | 0.766 | 719747 | similar to signaling lymphocytic activation molecule family member 1 | LOC719747 |
| MmuSTS.2457.1.S1_at | ENSMMUG00000022583 | 0.741 | 702905 | Calpain 11 | CAPN11 |
| MmuSTS.249.1.S1_at | ENSMMUG00000000951 | 1.052 | 710577 | integrin, beta 2 | ITGB2 |
| MmuSTS.2606.1.S1_at | ENSMMUG00000019996 | -0.755 | --- | --- | --- |
| MmuSTS.2685.1.S1_s_at | --- | 2.079 | 702712 | similar to G protein-coupled receptor 109A | LOC702712 |
| MmuSTS.2737.1.S1_at | ENSMMUG00000005318 | 1.909 | 711034 | ADAM-like, decysin 1 | ADAMDEC1 |
| MmuSTS.280.1.S1_at | --- | -0.670 | 700892 | iduronate 2-sulfatase | IDS |
| MmuSTS.2821.1.S1_at | ENSMMUG00000011713 | 0.930 | 702033 | leupaxin | LPXN |
| MmuSTS.2831.1.S1_at | ENSMMUG00000000413 | 0.703 | 712466 | hypothetical protein LOC712466 | LOC712466 |
| MmuSTS.2856.1.S1_at | --- | 1.856 | 722003 | Similar to eukaryotic translation elongation factor 1 alpha 1 | LOC722003 |
| MmuSTS.2862.1.S1_at | ENSMMUG00000008793 | 1.985 | 704930 | secreted phosphoprotein 1 | SPP1 |
| MmuSTS.2919.1.S1_s_at | --- | 0.982 | --- | --- | --- |
| MmuSTS.3015.1.S1_at | --- | -1.483 | --- | --- | --- |
| MmuSTS.3036.1.S1_at | --- | -0.662 | --- | --- | --- |
| MmuSTS.3131.1.S1_at | ENSMMUG00000003486 | 1.365 | 712754 | similar to Regulator of G-protein signaling 1 (RGS1) (Early response protein 1R20) (B-cell activation protein BL34) | LOC712754 |
| MmuSTS.3164.1.S1_at | ENSMMUG00000022139 | 0.704 | 701346 | Similar to 60 kDa heat shock protein, mitochondrial precursor (Hsp60) (60 kDa chaperonin) (CPN60) (Heat shock protein 60) (HSP-60) (Mitochondrial matrix protein P1) (P60 lymphocyte protein) (HuCHA60) | LOC701346 |
| MmuSTS.3202.1.S1_at | --- | 0.588 | 713049 | B-cell CLL/lymphoma 3 | BCL3 |
| MmuSTS.3251.1.S1_at | ENSMMUG00000032156 | 1.217 | 714826 | Similar to 60S ribosomal protein L7 | LOC714826 |
| MmuSTS.3253.1.S1_s_at | ENSMMUG00000014171 | 0.662 | --- | --- | --- |
| MmuSTS.3317.1.S1_at | ENSMMUG00000000288 | 1.380 | 574138 | chemokine CCL2/MCP-1 | MCP-1 |
| MmuSTS.3360.1.S1_at | --- | 0.815 | --- | --- | --- |
| MmuSTS.3361.1.S1_at | ENSMMUG00000004327 | 0.731 | 702435 | Similar to protocadherin 1 isoform 2 precursor | LOC702435 |
| MmuSTS.3375.1.S1_at | ENSMMUG00000000912 | 0.694 | --- | --- | --- |
| MmuSTS.3402.1.S1_at | ENSMMUG00000006127 | 0.596 | 704322 | CCAAT/enhancer binding protein (C/EBP), gamma | CEBPG |
| MmuSTS.3442.1.S1_at | ENSMMUG00000022275 | 0.729 | 706291 | acyloxyacyl hydrolase | AOAH |
| MmuSTS.3443.1.S1_at | ENSMMUG00000002546 | -0.598 | --- | --- | --- |
| MmuSTS.3453.1.S1_at | ENSMMUG00000021704 | 0.913 | 713563 | similar to PYD and CARD domain containing isoform b | LOC713563 |
| MmuSTS.3488.1.S1_at | --- | 1.562 | 719762 | CD48 molecule | CD48 |
| MmuSTS.3488.1.S1_s_at | --- | 1.353 | 719762 | CD48 molecule | CD48 |
| MmuSTS.3488.1.S1_x_at | --- | 1.462 | 719762 | CD48 molecule | CD48 |
| MmuSTS.3489.1.S1_at | --- | 1.104 | 715923 | similar to CD68 antigen | LOC715923 |
| MmuSTS.3532.1.S1_at | ENSMMUG00000000811 | 1.044 | 702350 | CD53 molecule | CD53 |
| MmuSTS.356.1.S1_s_at | ENSMMUG00000020374 | 0.606 | 705568 | Similar to signal recognition particle 68kDa | LOC705568 |
| MmuSTS.3569.1.S1_at | --- | 0.614 | --- | --- | --- |
| MmuSTS.3571.1.S1_s_at | ENSMMUG00000030216 /// ENSMMUG00000031494 | 1.869 | 619514 | chemokine (C-C motif) ligand 3 | CCL3 |
| MmuSTS.3587.1.S1_at | ENSMMUG00000012235 | 0.978 | 716043 | CD274 molecule | CD274 |
| MmuSTS.3643.1.S1_at | ENSMMUG00000008709 | 1.835 | 703286 | chitinase 1 (chitotriosidase) | CHIT1 |
| MmuSTS.3737.1.S1_at | ENSMMUG00000000009 | 0.874 | 712657 | similar to protein tyrosine phosphatase, receptor type, C isoform 1 precursor | LOC712657 |
| MmuSTS.3742.1.S1_at | ENSMMUG00000022850 | 0.947 | 704985 | pentraxin 3, long | PTX3 |
| MmuSTS.3824.1.S1_at | ENSMMUG00000022961 | 0.711 | --- | --- | --- |
| MmuSTS.3918.1.S1_at | ENSMMUG00000019546 | -0.723 | 705733 | regucalcin (senescence marker protein-30) | RGN |
| MmuSTS.3919.1.S1_s_at | ENSMMUG00000021610 | 0.661 | 703125 | regulator of G-protein signaling 10 | RGS10 |
| MmuSTS.3967.1.S1_at | ENSMMUG00000020434 | 0.617 | 713485 | colony stimulating factor 3 receptor (granulocyte) | CSF3R |
| MmuSTS.3971.1.S1_at | --- | 0.958 | 700672 | Similar to coilin | LOC700672 |
| MmuSTS.3980.1.S1_at | --- | -0.884 | --- | --- | --- |
| MmuSTS.3981.1.S1_at | --- | -0.659 | 713393 | SH3-domain GRB2-like 2 | SH3GL2 |
| MmuSTS.3988.1.S1_at | --- | 1.059 | 708080 | similar to Cathepsin S precursor | LOC708080 |
| MmuSTS.4000.1.S1_at | --- | 1.601 | 693386 /// 693406 | Hypothetical protein LOC693386 /// Similar to high-mobility group box 3 | LOC693386 /// LOC693406 |
| MmuSTS.4020.1.S1_at | --- | 1.619 | 710385 | similar to cytochrome P450, family 1, subfamily B, polypeptide 1 | LOC710385 |
| MmuSTS.4032.1.S1_at | ENSMMUG00000009112 | 0.916 | --- | --- | --- |
| MmuSTS.4036.1.S1_at | ENSMMUG00000006876 | -0.643 | 698011 | tetraspanin 7 | TSPAN7 |
| MmuSTS.4068.1.S1_at | ENSMMUG00000008387 | 0.832 | --- | --- | --- |
| MmuSTS.4091.1.S1_at | ENSMMUG00000012338 | 1.278 | 698844 | G protein-coupled receptor 183 | GPR183 |
| MmuSTS.4222.1.S1_at | ENSMMUG00000009906 | 0.948 | 705853 | plasminogen activator, urokinase | PLAU |
| MmuSTS.4227.1.S1_at | --- | 0.804 | 714613 | similar to peroxisomal short-chain alcohol dehydrogenase | LOC714613 |
| MmuSTS.4308.1.S1_s_at | --- | 0.781 | 709962 | Similar to tripartite motif protein TRIM4 isoform alpha | LOC709962 |
| MmuSTS.4339.1.S1_at | ENSMMUG00000011338 | 0.847 | 719494 | SLAM family member 8 | SLAMF8 |
| MmuSTS.4382.1.S1_at | ENSMMUG00000010456 | 0.611 | 718378 | similar to Folate receptor gamma precursor (FR-gamma) (Folate receptor 3) | LOC718378 |
| MmuSTS.4443.1.S1_s_at | --- | 0.836 | --- | --- | --- |
| MmuSTS.4444.1.S1_at | --- | -1.950 | 722683 | similar to Kallikrein-7 precursor (hK7) (Stratum corneum chymotryptic enzyme) (hSCCE) | LOC722683 |
| MmuSTS.4458.1.S1_at | ENSMMUG00000014434 | 0.638 | 716296 | Gardner-Rasheed feline sarcoma viral (v-fgr) oncogene homolog | FGR |
| MmuSTS.4526.1.S1_at | ENSMMUG00000001748 | 0.648 | 704102 | spleen tyrosine kinase | SYK |
| MmuSTS.4551.1.S1_at | --- | 0.635 | 703353 | hyaluronan synthase 2 | HAS2 |
| MmuSTS.4558.1.S1_at | ENSMMUG00000009859 | -0.646 | 716277 | similar to T-box 2 | LOC716277 |
| MmuSTS.4567.1.S1_at | ENSMMUG00000007055 | 0.973 | 711507 | lymphocyte antigen 86 | LY86 |
| MmuSTS.4659.1.S1_at | ENSMMUG00000008869 | 0.701 | 704130 | tumor necrosis factor, alpha-induced protein 3 | TNFAIP3 |
| MmuSTS.4660.1.S1_at | ENSMMUG00000010927 | 0.871 | 694699 | tumor necrosis factor, alpha-induced protein 6 | TNFAIP6 |
| MmuSTS.4669.1.S1_at | --- | 0.801 | 703189 | similar to tumor necrosis factor (ligand) superfamily, member 15 | LOC703189 |
| MmuSTS.4740.1.S1_at | ENSMMUG00000001248 | 1.198 | 711264 | coagulation factor XIII, A1 polypeptide | F13A1 |
| MmuSTS.4778.1.S1_at | ENSMMUG00000009912 | -0.658 | 721324 | similar to fibroblast growth factor 9 | LOC721324 |
| MmuSTS.4816.1.S1_at | ENSMMUG00000014808 | 0.675 | 701054 | growth arrest and DNA-damage-inducible, alpha | GADD45A |
| MmuSTS.4842.1.S1_at | ENSMMUG00000016671 | 1.567 | 705309 | G protein-coupled receptor 84 | GPR84 |
| MmuSTS.535.1.S1_at | ENSMMUG00000009736 | -0.726 | --- | --- | --- |
| MmuSTS.56.1.S1_at | ENSMMUG00000000117 | 1.019 | 712282 | Similar to Hermansky-Pudlak syndrome 3 protein | LOC712282 |
| MmuSTS.652.1.S1_at | ENSMMUG00000019778 | 2.284 | 704701 | interleukin 1, beta | IL1B |
| MmuSTS.690.1.S1_at | ENSMMUG00000003836 | 2.356 | --- | --- | --- |
| MmuSTS.718.1.S1_at | ENSMMUG00000015250 | 1.588 | 716122 | C-type lectin domain family 4, member A | CLEC4A |
| MmuSTS.721.1.S1_at | ENSMMUG00000008992 | 0.703 | 715503 | similar to N-acetylneuraminate pyruvate lyase | LOC715503 |
| MmuSTS.793.1.S1_at | ENSMMUG00000006470 | 0.627 | --- | --- | --- |
| MmuSTS.830.1.S1_at | ENSMMUG00000008604 | 1.066 | 713110 | similar to ecotropic viral integration site 2A isoform 2 | LOC713110 |
| MmuSTS.85.1.S1_s_at | --- | 1.030 | --- | --- | --- |
| MmuSTS.87.1.S1_at | --- | 1.385 | --- | --- | --- |
| MmuSTS.921.1.S1_at | ENSMMUG00000002037 | 2.340 | 703653 | matrix metallopeptidase 1 (interstitial collagenase) | MMP1 |
| MmuSTS.92.1.S1_at | ENSMMUG00000007221 | 0.766 | --- | --- | --- |
| MmuSTS.947.1.S1_at | ENSMMUG00000012596 | -0.633 | 677716 | Mitochondrial ribosomal protein L44 | MRPL44 |
| MmuSTS.991.1.S1_at | ENSMMUG00000018854 | 1.099 | 696709 | ras-related C3 botulinum toxin substrate 2 (rho family, small GTP binding protein Rac2) | RAC2 |
