# Supplementary material for: Choriodecidual Infection Downregulates Angiogenesis and Morphogenesis Pathways in Fetal Lungs from Macaca Nemestrina
Source: PLoS One. 2012 Oct 9;7(10):e46863. doi: 10.1371/journal.pone.0046863 (PMC3467273; doi:10.1371/journal.pone.0046863)
Supplement: Table S2 — Gene sets upregulated after GBS exposure relative to the control group. (DOCX) [file pone.0046863.s003.docx]

Table S2. Gene sets upregulated after GBS exposure relative to the control group

|  | Gene set length | Gene Ontology ID | p value |
| --- | --- | --- | --- |
|  |  |  |  |
| **Biological process** |  |  |  |
| pyrimidine base metabolic process | 24 | GO:0006206 | <0.002 |
| leukotriene metabolic process | 11 | GO:0006691 | <0.002 |
| cellular aromatic compound metabolic process | 8 | GO:0006725 | <0.002 |
| cell-cell signaling | 252 | GO:0007267 | <0.002 |
| phospholipid catabolic process | 15 | GO:0009395 | <0.002 |
| pyrimidine nucleoside salvage | 9 | GO:0043097 | <0.002 |
| positive regulation of innate immune response | 15 | GO:0045089 | 0.002 |
| prostaglandin biosynthetic process | 13 | GO:0001516 | 0.003 |
| temperature homeostasis | 18 | GO:0001659 | 0.003 |
| regulation of striated muscle contraction | 9 | GO:0006942 | 0.003 |
| dibenzo-p-dioxin metabolic process | 11 | GO:0018894 | 0.003 |
| intestinal cholesterol absorption | 6 | GO:0030299 | 0.003 |
| negative regulation of granulocyte differentiation | 6 | GO:0030853 | 0.003 |
| positive regulation of interleukin-8 production | 17 | GO:0032757 | 0.003 |
| negative regulation of peptidyl-tyrosine phosphorylation | 10 | GO:0050732 | 0.004 |
| positive regulation of type 2 immune response | 5 | GO:0002830 | 0.005 |
| response to nutrient | 127 | GO:0007584 | 0.005 |
| elevation of cytosolic calcium ion concentration | 114 | GO:0007204 | 0.006 |
| rhodopsin mediated phototransduction | 6 | GO:0009586 | 0.006 |
| cellular response to acid | 6 | GO:0071229 | 0.006 |
| activation of phospholipase C activity by G-protein coupled receptor protein signaling pathway coupled to IP3 second messenger | 34 | GO:0007200 | 0.007 |
| response to salt stress | 15 | GO:0009651 | 0.007 |
| response to axon injury | 30 | GO:0048678 | 0.007 |
| regulation of T cell activation | 7 | GO:0050863 | 0.007 |
| cellular response to interleukin-4 | 5 | GO:0071353 | 0.007 |
| dendritic cell chemotaxis | 13 | GO:0002407 | 0.008 |
| antigen processing and presentation of peptide or polysaccharide antigen via MHC class II | 11 | GO:0002504 | 0.008 |
| positive regulation of endopeptidase activity | 7 | GO:0010950 | 0.008 |
| immunoglobulin mediated immune response | 16 | GO:0016064 | 0.008 |
| antigen processing and presentation of exogenous peptide antigen via MHC class II | 8 | GO:0019886 | 0.008 |
| cellular nitrogen compound metabolic process | 189 | GO:0034641 | 0.008 |
| hydrogen peroxide biosynthetic process | 8 | GO:0050665 | 0.008 |
| negative regulation of nitric-oxide synthase activity | 7 | GO:0051001 | 0.008 |
| chaperone mediated protein folding requiring cofactor | 12 | GO:0051085 | 0.008 |
| positive regulation of transmission of nerve impulse | 5 | GO:0051971 | 0.008 |
| cellular response to interferon-gamma | 18 | GO:0071346 | 0.008 |
| gluconeogenesis | 43 | GO:0006094 | 0.009 |
| response to amine stimulus | 17 | GO:0014075 | 0.009 |
| microglial cell activation involved in immune response | 9 | GO:0002282 | 0.01 |
| feeding behavior | 38 | GO:0007631 | 0.01 |
| positive regulation of adaptive immune response | 5 | GO:0002821 | 0.011 |
| regulation of dopamine secretion | 10 | GO:0014059 | 0.011 |
| positive regulation of organ growth | 11 | GO:0046622 | 0.011 |
| positive regulation of macrophage cytokine production | 6 | GO:0060907 | 0.011 |
| response to fungus | 6 | GO:0009620 | 0.012 |
| pathogen-associated molecular pattern dependent induction by symbiont of host innate immunity | 8 | GO:0052033 | 0.012 |
| negative regulation of mature B cell apoptosis | 5 | GO:0002906 | 0.013 |
| interferon-gamma production | 5 | GO:0032609 | 0.013 |
| positive regulation of binding | 7 | GO:0051099 | 0.013 |
| positive regulation of interleukin-4 production | 7 | GO:0032753 | 0.014 |
| cellular defense response | 59 | GO:0006968 | 0.015 |
| cell communication | 104 | GO:0007154 | 0.015 |
| macrophage activation | 9 | GO:0042116 | 0.015 |
| positive thymic T cell selection | 8 | GO:0045059 | 0.015 |
| negative regulation of T cell apoptosis | 5 | GO:0070233 | 0.015 |
| positive regulation of neutrophil chemotaxis | 15 | GO:0090023 | 0.015 |
| leukotriene biosynthetic process | 22 | GO:0019370 | 0.016 |
| positive regulation of T cell differentiation | 10 | GO:0045582 | 0.016 |
| potassium ion homeostasis | 5 | GO:0055075 | 0.016 |
| regulation of alpha-amino-3-hydroxy-5-methyl-4-isoxazole propionate selective glutamate receptor activity | 9 | GO:2000311 | 0.016 |
| neuromuscular process controlling posture | 8 | GO:0050884 | 0.018 |
| release of sequestered calcium ion into cytosol | 24 | GO:0051209 | 0.018 |
| pattern recognition receptor signaling pathway | 9 | GO:0002221 | 0.019 |
| complement activation | 25 | GO:0006956 | 0.019 |
| gastric acid secretion | 7 | GO:0001696 | 0.02 |
| negative regulation of platelet-derived growth factor receptor signaling pathway | 5 | GO:0010642 | 0.02 |
| cellular response to drug | 11 | GO:0035690 | 0.02 |
| sodium ion transmembrane transport | 9 | GO:0035725 | 0.02 |
| mast cell activation | 9 | GO:0045576 | 0.02 |
| response to cadmium ion | 30 | GO:0046686 | 0.02 |
| arginine metabolic process | 5 | GO:0006525 | 0.021 |
| response to hydrogen peroxide | 59 | GO:0042542 | 0.021 |
| negative regulation of cAMP-mediated signaling | 5 | GO:0043951 | 0.021 |
| response to ATP | 17 | GO:0033198 | 0.022 |
| response to interferon-gamma | 19 | GO:0034341 | 0.022 |
| positive regulation of interleukin-8 biosynthetic process | 7 | GO:0045416 | 0.022 |
| nucleobase, nucleoside and nucleotide metabolic process | 76 | GO:0055086 | 0.022 |
| righting reflex | 7 | GO:0060013 | 0.022 |
| chemotaxis | 123 | GO:0006935 | 0.023 |
| sensory perception | 11 | GO:0007600 | 0.023 |
| protein refolding | 6 | GO:0042026 | 0.023 |
| positive regulation of macrophage differentiation | 7 | GO:0045651 | 0.023 |
| regulation of action potential | 16 | GO:0001508 | 0.024 |
| anion transport | 27 | GO:0006820 | 0.024 |
| response to sucrose stimulus | 10 | GO:0009744 | 0.024 |
| canalicular bile acid transport | 5 | GO:0015722 | 0.024 |
| cellular carbohydrate metabolic process | 10 | GO:0044262 | 0.024 |
| negative regulation of neural precursor cell proliferation | 5 | GO:2000178 | 0.024 |
| positive regulation of dendritic cell antigen processing and presentation | 7 | GO:0002606 | 0.025 |
| penetration of zona pellucida | 5 | GO:0007341 | 0.026 |
| positive regulation of interleukin-10 production | 16 | GO:0032733 | 0.026 |
| drug catabolic process | 5 | GO:0042737 | 0.026 |
| steroid catabolic process | 6 | GO:0006706 | 0.027 |
| epithelial cell differentiation | 47 | GO:0030855 | 0.027 |
| pyruvate biosynthetic process | 5 | GO:0042866 | 0.027 |
| maintenance of gastrointestinal epithelium | 6 | GO:0030277 | 0.028 |
| positive regulation of actin filament polymerization | 24 | GO:0030838 | 0.028 |
| purinergic nucleotide receptor signaling pathway | 7 | GO:0035590 | 0.028 |
| positive regulation of interferon-beta biosynthetic process | 7 | GO:0045359 | 0.028 |
| sodium-dependent phosphate transport | 7 | GO:0044341 | 0.029 |
| positive regulation of angiogenesis | 63 | GO:0045766 | 0.029 |
| adenosine metabolic process | 9 | GO:0046085 | 0.029 |
| sperm motility | 28 | GO:0030317 | 0.03 |
| male gonad development | 83 | GO:0008584 | 0.031 |
| neutrophil chemotaxis | 29 | GO:0030593 | 0.031 |
| respiratory burst | 12 | GO:0045730 | 0.031 |
| positive regulation of inflammatory response | 39 | GO:0050729 | 0.031 |
| zinc ion transmembrane transport | 17 | GO:0071577 | 0.031 |
| tryptophan catabolic process | 11 | GO:0006569 | 0.033 |
| inflammatory response | 268 | GO:0006954 | 0.033 |
| peptidoglycan catabolic process | 7 | GO:0009253 | 0.033 |
| positive regulation of calcium-mediated signaling | 21 | GO:0050850 | 0.033 |
| response to amphetamine | 30 | GO:0001975 | 0.034 |
| negative regulation of osteoclast differentiation | 17 | GO:0045671 | 0.034 |
| negative regulation of blood pressure | 25 | GO:0045776 | 0.034 |
| macrophage chemotaxis | 11 | GO:0048246 | 0.034 |
| positive regulation of peptidyl-tyrosine phosphorylation | 61 | GO:0050731 | 0.034 |
| oxidative demethylation | 9 | GO:0070989 | 0.034 |
| ion transport | 611 | GO:0006811 | 0.035 |
| cation transport | 97 | GO:0006812 | 0.035 |
| negative regulation of glutamate secretion | 8 | GO:0014050 | 0.035 |
| platelet aggregation | 6 | GO:0070527 | 0.035 |
| regulation of glycolysis | 7 | GO:0006110 | 0.036 |
| complement activation, alternative pathway | 12 | GO:0006957 | 0.036 |
| regulation of S phase | 7 | GO:0033261 | 0.036 |
| positive regulation of blood pressure | 25 | GO:0045777 | 0.036 |
| behavioral response to pain | 13 | GO:0048266 | 0.036 |
| cellular response to glucocorticoid stimulus | 19 | GO:0071385 | 0.036 |
| glycolysis | 43 | GO:0006096 | 0.037 |
| positive regulation of MHC class I biosynthetic process | 5 | GO:0045345 | 0.037 |
| negative regulation of lymphocyte proliferation | 6 | GO:0050672 | 0.037 |
| adaptive immune response | 5 | GO:0002250 | 0.038 |
| response to organic substance | 124 | GO:0010033 | 0.039 |
| organic anion transport | 24 | GO:0015711 | 0.039 |
| neuron projection regeneration | 5 | GO:0031102 | 0.039 |
| T cell proliferation | 18 | GO:0042098 | 0.039 |
| G-protein signaling, coupled to cyclic nucleotide second messenger | 41 | GO:0007187 | 0.04 |
| purine-containing compound salvage | 12 | GO:0043101 | 0.04 |
| positive regulation of ERK1 and ERK2 cascade | 54 | GO:0070374 | 0.04 |
| cellular response to lipoteichoic acid | 7 | GO:0071223 | 0.04 |
| transformed cell apoptosis | 5 | GO:0006927 | 0.041 |
| positive regulation of B cell proliferation | 35 | GO:0030890 | 0.041 |
| activation of Rho GTPase activity | 7 | GO:0032862 | 0.041 |
| negative regulation of thymocyte apoptosis | 5 | GO:0070244 | 0.041 |
| response to carbohydrate stimulus | 17 | GO:0009743 | 0.042 |
| monoterpenoid metabolic process | 5 | GO:0016098 | 0.042 |
| positive regulation of interleukin-6 production | 32 | GO:0032755 | 0.042 |
| negative regulation of I-kappaB kinase/NF-kappaB cascade | 14 | GO:0043124 | 0.042 |
| positive regulation of circadian sleep/wake cycle, non-REM sleep | 5 | GO:0046010 | 0.042 |
| positive regulation of alpha-beta T cell proliferation | 12 | GO:0046641 | 0.043 |
| lymphocyte chemotaxis | 8 | GO:0048247 | 0.043 |
| immunoglobulin secretion | 6 | GO:0048305 | 0.043 |
| response to cAMP | 70 | GO:0051591 | 0.043 |
| inflammatory response to antigenic stimulus | 7 | GO:0002437 | 0.044 |
| negative regulation of tumor necrosis factor production | 22 | GO:0032720 | 0.044 |
| negative regulation of hormone secretion | 13 | GO:0046888 | 0.044 |
| positive regulation of immune response | 11 | GO:0050778 | 0.044 |
| cytokine production | 20 | GO:0001816 | 0.045 |
| striated muscle contraction | 18 | GO:0006941 | 0.045 |
| immune response | 329 | GO:0006955 | 0.045 |
| tryptophan catabolic process to kynurenine | 7 | GO:0019441 | 0.045 |
| cellular response to nutrient levels | 5 | GO:0031669 | 0.045 |
| cellular response to lipopolysaccharide | 55 | GO:0071222 | 0.045 |
| immunological synapse formation | 5 | GO:0001771 | 0.046 |
| sodium ion transport | 123 | GO:0006814 | 0.046 |
| digestion | 46 | GO:0007586 | 0.046 |
| toxin metabolic process | 7 | GO:0009404 | 0.046 |
| regulation of proteolysis | 37 | GO:0030162 | 0.046 |
| T-helper 1 type immune response | 10 | GO:0042088 | 0.046 |
| negative regulation of phagocytosis | 7 | GO:0050765 | 0.046 |
| defense response to Gram-positive bacterium | 43 | GO:0050830 | 0.046 |
| interferon-gamma-mediated signaling pathway | 63 | GO:0060333 | 0.046 |
| response to molecule of bacterial origin | 11 | GO:0002237 | 0.047 |
| peptide cross-linking | 22 | GO:0018149 | 0.047 |
| negative regulation of interleukin-2 production | 10 | GO:0032703 | 0.047 |
| oligopeptide transmembrane transport | 6 | GO:0035672 | 0.047 |
| release of cytochrome c from mitochondria | 23 | GO:0001836 | 0.048 |
| purine ribonucleoside monophosphate biosynthetic process | 16 | GO:0009168 | 0.048 |
| arachidonic acid metabolic process | 8 | GO:0019369 | 0.048 |
| response to prostaglandin E stimulus | 9 | GO:0034695 | 0.048 |
| hormone biosynthetic process | 61 | GO:0042446 | 0.048 |
| membrane depolarization | 24 | GO:0051899 | 0.048 |
| positive regulation of leukocyte chemotaxis | 9 | GO:0002690 | 0.049 |
| oligopeptide transport | 7 | GO:0006857 | 0.049 |
| adult feeding behavior | 8 | GO:0008343 | 0.049 |
| negative regulation of smooth muscle cell migration | 10 | GO:0014912 | 0.049 |
| T cell costimulation | 67 | GO:0031295 | 0.05 |
| cellular response to interferon-beta | 10 | GO:0035458 | 0.05 |
| exogenous drug catabolic process | 9 | GO:0042738 | 0.05 |
| positive regulation of synaptic transmission, glutamatergic | 11 | GO:0051968 | 0.05 |
| desensitization of G-protein coupled receptor protein signaling pathway | 5 | GO:0002029 | 0.051 |
| negative regulation of peptidase activity | 68 | GO:0010466 | 0.051 |
| response to tumor necrosis factor | 24 | GO:0034612 | 0.051 |
| eating behavior | 30 | GO:0042755 | 0.051 |
| negative regulation of G-protein coupled receptor protein signaling pathway | 11 | GO:0045744 | 0.051 |
| positive regulation of protein kinase activity | 33 | GO:0045860 | 0.051 |
| T cell receptor signaling pathway | 77 | GO:0050852 | 0.051 |
| sterol biosynthetic process | 25 | GO:0016126 | 0.052 |
| antigen processing and presentation | 33 | GO:0019882 | 0.052 |
| negative regulation of calcium ion transport | 5 | GO:0051926 | 0.052 |
| mineralocorticoid biosynthetic process | 5 | GO:0006705 | 0.053 |
| positive regulation of cell proliferation | 386 | GO:0008284 | 0.053 |
| oligosaccharide metabolic process | 10 | GO:0009311 | 0.053 |
| embryo development ending in birth or egg hatching | 8 | GO:0009792 | 0.053 |
| regulation of immune response | 84 | GO:0050776 | 0.053 |
| activated T cell proliferation | 9 | GO:0050798 | 0.053 |
| leukocyte migration | 109 | GO:0050900 | 0.053 |
| maternal process involved in female pregnancy | 19 | GO:0060135 | 0.053 |
| regulation of feeding behavior | 6 | GO:0060259 | 0.053 |
| muscarinic acetylcholine receptor signaling pathway | 11 | GO:0007213 | 0.054 |
| binding of sperm to zona pellucida | 17 | GO:0007339 | 0.054 |
| positive regulation of cellular protein metabolic process | 11 | GO:0032270 | 0.054 |
| ribosomal small subunit assembly | 8 | GO:0000028 | 0.055 |
| mesenchymal to epithelial transition involved in metanephros morphogenesis | 6 | GO:0003337 | 0.055 |
| activation of pro-apoptotic gene products | 33 | GO:0008633 | 0.055 |
| cell proliferation in forebrain | 9 | GO:0021846 | 0.055 |
| organ regeneration | 77 | GO:0031100 | 0.055 |
| negative regulation of interferon-gamma production | 14 | GO:0032689 | 0.055 |
| cellular response to mechanical stimulus | 53 | GO:0071260 | 0.055 |
| porphyrin metabolic process | 16 | GO:0006778 | 0.056 |
| heterocycle metabolic process | 5 | GO:0046483 | 0.056 |
| negative regulation of fibroblast proliferation | 18 | GO:0048147 | 0.056 |
| cell maturation | 35 | GO:0048469 | 0.056 |
| positive regulation of nitric-oxide synthase biosynthetic process | 6 | GO:0051770 | 0.056 |
| humoral immune response | 40 | GO:0006959 | 0.057 |
| cytokine-mediated signaling pathway | 165 | GO:0019221 | 0.057 |
| negative thymic T cell selection | 12 | GO:0045060 | 0.057 |
| negative regulation of macrophage differentiation | 5 | GO:0045650 | 0.057 |
| negative regulation of bone resorption | 9 | GO:0045779 | 0.057 |
| cellular response to organic substance | 13 | GO:0071310 | 0.057 |
| antigen processing and presentation of peptide antigen via MHC class I | 10 | GO:0002474 | 0.058 |
| cellular zinc ion homeostasis | 7 | GO:0006882 | 0.058 |
| programmed cell death | 12 | GO:0012501 | 0.058 |
| gap junction assembly | 6 | GO:0016264 | 0.058 |
| superoxide anion generation | 14 | GO:0042554 | 0.058 |
| sphingomyelin catabolic process | 5 | GO:0006685 | 0.059 |
| cell surface receptor linked signaling pathway | 239 | GO:0007166 | 0.059 |
| hemostasis | 12 | GO:0007599 | 0.059 |
| positive regulation of cell adhesion mediated by integrin | 8 | GO:0033630 | 0.059 |
| positive regulation of immunoglobulin secretion | 7 | GO:0051024 | 0.059 |
| positive regulation of chemokine secretion | 5 | GO:0090197 | 0.059 |
| behavioral fear response | 22 | GO:0001662 | 0.06 |
| receptor guanylyl cyclase signaling pathway | 11 | GO:0007168 | 0.06 |
| positive regulation of T-helper 2 cell differentiation | 5 | GO:0045630 | 0.061 |
| natural killer cell activation | 14 | GO:0030101 | 0.062 |
| positive regulation of insulin secretion | 34 | GO:0032024 | 0.062 |
| negative regulation of interleukin-17 production | 8 | GO:0032700 | 0.062 |
| inositol phosphate-mediated signaling | 8 | GO:0048016 | 0.062 |
| endothelial cell proliferation | 10 | GO:0001935 | 0.064 |
| epithelial cell development | 7 | GO:0002064 | 0.064 |
| superoxide metabolic process | 16 | GO:0006801 | 0.064 |
| cerebral cortex GABAergic interneuron migration | 5 | GO:0021853 | 0.064 |
| positive regulation of T-helper 1 cell differentiation | 7 | GO:0045627 | 0.064 |
| lymphocyte proliferation | 5 | GO:0046651 | 0.064 |
| erythrocyte development | 12 | GO:0048821 | 0.064 |
| RNA catabolic process | 15 | GO:0006401 | 0.065 |
| sensory perception of chemical stimulus | 5 | GO:0007606 | 0.065 |
| ion transmembrane transport | 165 | GO:0034220 | 0.065 |
| positive regulation of bone resorption | 9 | GO:0045780 | 0.065 |
| positive regulation of leukocyte migration | 16 | GO:0002687 | 0.066 |
| leukocyte cell-cell adhesion | 28 | GO:0007159 | 0.066 |
| proteolysis | 424 | GO:0006508 | 0.067 |
| nitric oxide biosynthetic process | 16 | GO:0006809 | 0.067 |
| phosphate transport | 11 | GO:0006817 | 0.067 |
| acute-phase response | 36 | GO:0006953 | 0.067 |
| visual perception | 211 | GO:0007601 | 0.067 |
| innate immune response | 228 | GO:0045087 | 0.067 |
| bone resorption | 22 | GO:0045453 | 0.067 |
| negative regulation of leukocyte migration | 5 | GO:0002686 | 0.068 |
| proline biosynthetic process | 5 | GO:0006561 | 0.068 |
| retinal cone cell development | 6 | GO:0046549 | 0.068 |
| cytokine secretion | 6 | GO:0050663 | 0.068 |
| cellular response to tumor necrosis factor | 22 | GO:0071356 | 0.068 |
| glycerol-3-phosphate metabolic process | 7 | GO:0006072 | 0.069 |
| G-protein signaling, coupled to cAMP nucleotide second messenger | 46 | GO:0007188 | 0.069 |
| induction of apoptosis by hormones | 5 | GO:0008628 | 0.069 |
| cerebellar granule cell differentiation | 6 | GO:0021707 | 0.069 |
| keratinization | 25 | GO:0031424 | 0.069 |
| chondroblast differentiation | 5 | GO:0060591 | 0.069 |
| malate metabolic process | 8 | GO:0006108 | 0.07 |
| I-kappaB phosphorylation | 11 | GO:0007252 | 0.07 |
| antigen processing and presentation of endogenous peptide antigen via MHC class I | 6 | GO:0019885 | 0.07 |
| lipopolysaccharide-mediated signaling pathway | 21 | GO:0031663 | 0.07 |
| positive regulation of interferon-gamma production | 29 | GO:0032729 | 0.07 |
| negative regulation of renal sodium excretion | 5 | GO:0035814 | 0.07 |
| B cell proliferation | 15 | GO:0042100 | 0.07 |
| positive regulation of survival gene product expression | 11 | GO:0045885 | 0.07 |
| monocyte chemotaxis | 7 | GO:0002548 | 0.071 |
| DNA damage response, signal transduction by p53 class mediator resulting in transcription of p21 class mediator | 11 | GO:0006978 | 0.071 |
| intracellular transport of viral proteins in host cell | 5 | GO:0019060 | 0.071 |
| vasodilation | 21 | GO:0042311 | 0.071 |
| drinking behavior | 10 | GO:0042756 | 0.071 |
| negative regulation of DNA damage response, signal transduction by p53 class mediator | 7 | GO:0043518 | 0.071 |
| positive regulation of phagocytosis | 26 | GO:0050766 | 0.071 |
| positive regulation of calcium ion transport | 17 | GO:0051928 | 0.071 |
| purine base metabolic process | 32 | GO:0006144 | 0.072 |
| complement activation, classical pathway | 33 | GO:0006958 | 0.072 |
| multicellular organismal aging | 13 | GO:0010259 | 0.072 |
| negative regulation of protein autophosphorylation | 8 | GO:0031953 | 0.073 |
| response to lipopolysaccharide | 179 | GO:0032496 | 0.073 |
| intermediate filament cytoskeleton organization | 10 | GO:0045104 | 0.073 |
| regulation of neuronal synaptic plasticity | 26 | GO:0048168 | 0.073 |
| establishment of T cell polarity | 5 | GO:0001768 | 0.074 |
| protein methylation | 22 | GO:0006479 | 0.074 |
| water transport | 36 | GO:0006833 | 0.075 |
| inhibition of adenylate cyclase activity by G-protein signaling pathway | 35 | GO:0007193 | 0.075 |
| forebrain neuron development | 5 | GO:0021884 | 0.075 |
| leukocyte chemotaxis | 11 | GO:0030595 | 0.075 |
| positive regulation of fever generation | 8 | GO:0031622 | 0.075 |
| negative regulation of inflammatory response to antigenic stimulus | 7 | GO:0002862 | 0.076 |
| blood circulation | 47 | GO:0008015 | 0.076 |
| negative regulation of endopeptidase activity | 66 | GO:0010951 | 0.076 |
| ATP hydrolysis coupled proton transport | 33 | GO:0015991 | 0.076 |
| lipid catabolic process | 88 | GO:0016042 | 0.076 |
| response to follicle-stimulating hormone stimulus | 5 | GO:0032354 | 0.076 |
| positive regulation of T cell proliferation | 43 | GO:0042102 | 0.076 |
| response to drug | 435 | GO:0042493 | 0.076 |
| positive regulation of osteoclast differentiation | 17 | GO:0045672 | 0.076 |
| B cell homeostasis | 14 | GO:0001782 | 0.077 |
| response to herbicide | 14 | GO:0009635 | 0.077 |
| regulation of innate immune response | 8 | GO:0045088 | 0.077 |
| detection of light stimulus involved in visual perception | 7 | GO:0050908 | 0.077 |
| phagocytosis, engulfment | 13 | GO:0006911 | 0.078 |
| acrosome reaction | 15 | GO:0007340 | 0.078 |
| forebrain dorsal/ventral pattern formation | 5 | GO:0021798 | 0.078 |
| negative regulation of interleukin-10 production | 7 | GO:0032693 | 0.079 |
| positive regulation of tumor necrosis factor production | 30 | GO:0032760 | 0.079 |
| response to ethanol | 124 | GO:0045471 | 0.079 |
| cellular calcium ion homeostasis | 84 | GO:0006874 | 0.08 |
| proton transport | 63 | GO:0015992 | 0.08 |
| response to chemical stimulus | 38 | GO:0042221 | 0.08 |
| positive regulation of tumor necrosis factor biosynthetic process | 10 | GO:0042535 | 0.08 |
| negative regulation of fatty acid biosynthetic process | 9 | GO:0045717 | 0.08 |
| defense response | 83 | GO:0006952 | 0.081 |
| fibrinolysis | 16 | GO:0042730 | 0.081 |
| positive regulation of macrophage activation | 11 | GO:0043032 | 0.081 |
| long-term synaptic potentiation | 17 | GO:0060291 | 0.081 |
| neuromuscular synaptic transmission | 16 | GO:0007274 | 0.082 |
| response to wounding | 76 | GO:0009611 | 0.082 |
| positive regulation of chemokine production | 16 | GO:0032722 | 0.082 |
| positive regulation of interferon-beta production | 15 | GO:0032728 | 0.082 |
| positive regulation of stress-activated MAPK cascade | 14 | GO:0032874 | 0.083 |
| response to interleukin-4 | 6 | GO:0070670 | 0.083 |
| cellular response to heat | 18 | GO:0034605 | 0.084 |
| neutrophil activation | 6 | GO:0042119 | 0.084 |
| negative regulation of T cell proliferation | 24 | GO:0042130 | 0.084 |
| membrane hyperpolarization | 5 | GO:0060081 | 0.084 |
| microglial cell activation | 5 | GO:0001774 | 0.085 |
| regulation of pH | 19 | GO:0006885 | 0.085 |
| G-protein coupled purinergic nucleotide receptor signaling pathway | 21 | GO:0035589 | 0.085 |
| regulation of membrane potential | 46 | GO:0042391 | 0.085 |
| defense response to virus | 41 | GO:0051607 | 0.085 |
| cellular response to cytokine stimulus | 7 | GO:0071345 | 0.085 |
| cellular response to ethanol | 5 | GO:0071361 | 0.085 |
| cellular response to gonadotropin stimulus | 12 | GO:0071371 | 0.085 |
| response to interleukin-1 | 33 | GO:0070555 | 0.086 |
| detection of chemical stimulus involved in sensory perception of bitter taste | 8 | GO:0001580 | 0.087 |
| positive regulation of T cell mediated cytotoxicity | 13 | GO:0001916 | 0.087 |
| neuropeptide signaling pathway | 103 | GO:0007218 | 0.087 |
| detection of visible light | 8 | GO:0009584 | 0.087 |
| positive regulation of macrophage chemotaxis | 8 | GO:0010759 | 0.087 |
| collagen catabolic process | 22 | GO:0030574 | 0.087 |
| defense response to bacterium | 75 | GO:0042742 | 0.087 |
| activation of protein kinase C activity by G-protein coupled receptor protein signaling pathway | 38 | GO:0007205 | 0.088 |
| response to bacterium | 35 | GO:0009617 | 0.088 |
| cellular extravasation | 5 | GO:0045123 | 0.088 |
| decidualization | 17 | GO:0046697 | 0.088 |
| grooming behavior | 13 | GO:0007625 | 0.089 |
| monovalent inorganic cation transport | 12 | GO:0015672 | 0.089 |
| cytolysis | 21 | GO:0019835 | 0.089 |
| positive regulation of interferon-alpha production | 7 | GO:0032727 | 0.09 |
| response to starvation | 28 | GO:0042594 | 0.09 |
| negative regulation of interleukin-6 biosynthetic process | 5 | GO:0045409 | 0.09 |
| G1/S transition of mitotic cell cycle | 139 | GO:0000082 | 0.091 |
| response to organic cyclic compound | 206 | GO:0014070 | 0.091 |
| retrograde protein transport, ER to cytosol | 5 | GO:0030970 | 0.091 |
| positive regulation of interleukin-1 beta secretion | 17 | GO:0050718 | 0.091 |
| cellular response to glucose stimulus | 23 | GO:0071333 | 0.091 |
| apoptosis | 673 | GO:0006915 | 0.092 |
| defense response to Gram-negative bacterium | 17 | GO:0050829 | 0.092 |
| sensory perception of light stimulus | 12 | GO:0050953 | 0.092 |
| nucleoside metabolic process | 17 | GO:0009116 | 0.093 |
| anaphase-promoting complex-dependent proteasomal ubiquitin-dependent protein catabolic process | 76 | GO:0031145 | 0.093 |
| regulation of synaptic transmission, GABAergic | 7 | GO:0032228 | 0.093 |
| positive regulation of interferon-gamma biosynthetic process | 12 | GO:0045078 | 0.093 |
| negative regulation of B cell activation | 6 | GO:0050869 | 0.093 |
| opioid receptor signaling pathway | 5 | GO:0038003 | 0.094 |
| positive regulation of gamma-delta T cell differentiation | 7 | GO:0045588 | 0.094 |
| glycerol transport | 6 | GO:0015793 | 0.095 |
| response to exogenous dsRNA | 14 | GO:0043330 | 0.095 |
| negative regulation of lipid storage | 7 | GO:0010888 | 0.096 |
| sodium-independent organic anion transport | 12 | GO:0043252 | 0.097 |
| proteolysis involved in cellular protein catabolic process | 27 | GO:0051603 | 0.097 |
| regulation of cellular amino acid metabolic process | 50 | GO:0006521 | 0.098 |
| response to hormone stimulus | 91 | GO:0009725 | 0.098 |
| tumor necrosis factor-mediated signaling pathway | 26 | GO:0033209 | 0.098 |
| positive regulation of nitric oxide biosynthetic process | 32 | GO:0045429 | 0.098 |
| negative regulation of ubiquitin-protein ligase activity involved in mitotic cell cycle | 64 | GO:0051436 | 0.098 |
| response to protein stimulus | 73 | GO:0051789 | 0.098 |
| response to glucose stimulus | 97 | GO:0009749 | 0.099 |
| response to organic nitrogen | 56 | GO:0010243 | 0.099 |
| ionotropic glutamate receptor signaling pathway | 12 | GO:0035235 | 0.099 |
| B cell receptor signaling pathway | 24 | GO:0050853 | 0.099 |
| positive regulation of epithelial cell proliferation involved in wound healing | 5 | GO:0060054 | 0.099 |
| galactose metabolic process | 7 | GO:0006012 | 0.1 |
| chitin catabolic process | 7 | GO:0006032 | 0.1 |
| activation of caspase activity | 78 | GO:0006919 | 0.1 |
| aging | 160 | GO:0007568 | 0.1 |
| low-density lipoprotein particle clearance | 7 | GO:0034383 | 0.1 |
| response to heat | 66 | GO:0009408 | 0.101 |
| positive regulation vascular endothelial growth factor production | 13 | GO:0010575 | 0.101 |
| positive regulation of interleukin-12 biosynthetic process | 7 | GO:0045084 | 0.102 |
| astrocyte differentiation | 7 | GO:0048708 | 0.102 |
| protein import into nucleus, translocation | 20 | GO:0000060 | 0.103 |
| protein targeting to membrane | 18 | GO:0006612 | 0.103 |
| positive regulation of cholesterol efflux | 9 | GO:0010875 | 0.103 |
| pyrimidine nucleoside catabolic process | 12 | GO:0046135 | 0.103 |
| positive regulation of alpha-beta T cell differentiation | 9 | GO:0046638 | 0.103 |
| regulation of blood pressure | 64 | GO:0008217 | 0.104 |
| regulation of cell adhesion mediated by integrin | 6 | GO:0033628 | 0.104 |
| regulation of neurotransmitter secretion | 22 | GO:0046928 | 0.104 |
| cellular response to interleukin-1 | 18 | GO:0071347 | 0.104 |
| glucocorticoid biosynthetic process | 9 | GO:0006704 | 0.105 |
| chloride transport | 82 | GO:0006821 | 0.105 |
| positive regulation of macrophage derived foam cell differentiation | 13 | GO:0010744 | 0.105 |
| positive regulation of isotype switching to IgG isotypes | 7 | GO:0048304 | 0.105 |
| mesonephros development | 12 | GO:0001823 | 0.106 |
| synaptic transmission, cholinergic | 19 | GO:0007271 | 0.106 |
| peripheral nervous system development | 36 | GO:0007422 | 0.106 |
| lymph node development | 19 | GO:0048535 | 0.106 |
| response to hyperoxia | 28 | GO:0055093 | 0.106 |
| DNA damage response, signal transduction by p53 class mediator resulting in cell cycle arrest | 60 | GO:0006977 | 0.107 |
| ovulation | 9 | GO:0030728 | 0.107 |
| positive regulation of interleukin-12 production | 21 | GO:0032735 | 0.107 |
| hyperosmotic salinity response | 9 | GO:0042538 | 0.107 |
| skeletal muscle tissue regeneration | 12 | GO:0043403 | 0.107 |
| positive regulation of adenylate cyclase activity | 5 | GO:0045762 | 0.107 |
| positive regulation of ubiquitin-protein ligase activity involved in mitotic cell cycle | 68 | GO:0051437 | 0.107 |
| parathyroid gland development | 6 | GO:0060017 | 0.107 |
| positive regulation of growth hormone secretion | 9 | GO:0060124 | 0.107 |
| steroid biosynthetic process | 56 | GO:0006694 | 0.108 |
| response to ozone | 5 | GO:0010193 | 0.108 |
| pyridine nucleotide biosynthetic process | 12 | GO:0019363 | 0.108 |
| regulation of macrophage activation | 6 | GO:0043030 | 0.108 |
| response to stimulus | 261 | GO:0050896 | 0.108 |
| equilibrioception | 6 | GO:0050957 | 0.108 |
| regulation of ubiquitin-protein ligase activity involved in mitotic cell cycle | 72 | GO:0051439 | 0.108 |
| acute inflammatory response | 15 | GO:0002526 | 0.109 |
| cytoplasmic sequestering of NF-kappaB | 6 | GO:0007253 | 0.11 |
| cellular response to protein stimulus | 58 | GO:0071445 | 0.11 |
| manganese ion transport | 6 | GO:0006828 | 0.111 |
| positive regulation of interleukin-6 biosynthetic process | 7 | GO:0045410 | 0.111 |
| brown fat cell differentiation | 29 | GO:0050873 | 0.111 |
| negative regulation of lipid catabolic process | 11 | GO:0050995 | 0.111 |
| induction of apoptosis via death domain receptors | 17 | GO:0008625 | 0.112 |
| T cell activation | 39 | GO:0042110 | 0.112 |
| cellular iron ion homeostasis | 63 | GO:0006879 | 0.113 |
| chemosensory behavior | 6 | GO:0007635 | 0.113 |
| heme catabolic process | 5 | GO:0042167 | 0.113 |
| positive regulation of interleukin-2 biosynthetic process | 13 | GO:0045086 | 0.113 |
| phthalate metabolic process | 7 | GO:0018963 | 0.114 |
| response to hydroperoxide | 5 | GO:0033194 | 0.114 |
| adiponectin-mediated signaling pathway | 5 | GO:0033211 | 0.114 |
| middle ear morphogenesis | 20 | GO:0042474 | 0.114 |
| positive regulation of MHC class II biosynthetic process | 7 | GO:0045348 | 0.114 |
| lung epithelial cell differentiation | 6 | GO:0060487 | 0.114 |
| carbohydrate biosynthetic process | 16 | GO:0016051 | 0.115 |
| regulation of apoptosis | 228 | GO:0042981 | 0.115 |
| metanephric collecting duct development | 6 | GO:0072205 | 0.115 |
| positive regulation of branching involved in ureteric bud morphogenesis | 14 | GO:0090190 | 0.115 |
| positive regulation of humoral immune response mediated by circulating immunoglobulin | 7 | GO:0002925 | 0.116 |
| glycosaminoglycan catabolic process | 6 | GO:0006027 | 0.116 |
| dopamine receptor signaling pathway | 13 | GO:0007212 | 0.116 |
| regulation of heart rate | 13 | GO:0002027 | 0.117 |
| response to cytokine stimulus | 85 | GO:0034097 | 0.117 |
| negative regulation of viral genome replication | 10 | GO:0045071 | 0.117 |
| response to mercury ion | 11 | GO:0046689 | 0.117 |
| apoptosis involved in morphogenesis | 6 | GO:0060561 | 0.117 |
| opsonization | 8 | GO:0008228 | 0.118 |
| response to gonadotropin stimulus | 14 | GO:0034698 | 0.118 |
| negative regulation of potassium ion transport | 9 | GO:0043267 | 0.118 |
| response to glucocorticoid stimulus | 123 | GO:0051384 | 0.118 |
| T cell differentiation | 31 | GO:0030217 | 0.119 |
| neurological system process | 14 | GO:0050877 | 0.119 |
| leukotriene production involved in inflammatory response | 5 | GO:0002540 | 0.12 |
| urate metabolic process | 10 | GO:0046415 | 0.12 |
| detection of mechanical stimulus involved in sensory perception of sound | 11 | GO:0050910 | 0.12 |
| gamma-aminobutyric acid secretion | 5 | GO:0014051 | 0.121 |
| positive regulation of lipid catabolic process | 5 | GO:0050996 | 0.121 |
| cellular response to follicle-stimulating hormone stimulus | 9 | GO:0071372 | 0.121 |
| phosphate transmembrane transport | 5 | GO:0035435 | 0.122 |
| clathrin coat assembly | 9 | GO:0048268 | 0.122 |
| positive regulation of neurogenesis | 18 | GO:0050769 | 0.122 |
| type I interferon-mediated signaling pathway | 54 | GO:0060337 | 0.122 |
| response to dietary excess | 7 | GO:0002021 | 0.123 |
| bicarbonate transport | 16 | GO:0015701 | 0.123 |
| positive regulation of synaptic transmission | 18 | GO:0050806 | 0.123 |
| cholesterol biosynthetic process | 32 | GO:0006695 | 0.124 |
| hair cycle | 5 | GO:0042633 | 0.125 |
| penile erection | 5 | GO:0043084 | 0.125 |
| response to UV-C | 9 | GO:0010225 | 0.126 |
| negative regulation of interleukin-6 production | 14 | GO:0032715 | 0.126 |
| positive regulation of B cell differentiation | 12 | GO:0045579 | 0.126 |
| M/G1 transition of mitotic cell cycle | 72 | GO:0000216 | 0.127 |
| embryo implantation | 38 | GO:0007566 | 0.127 |
| lipid storage | 14 | GO:0019915 | 0.127 |
| water homeostasis | 9 | GO:0030104 | 0.127 |
| positive regulation of activated T cell proliferation | 20 | GO:0042104 | 0.127 |
| transmembrane transport | 677 | GO:0055085 | 0.127 |
| phospholipid metabolic process | 34 | GO:0006644 | 0.128 |
| spinal cord association neuron differentiation | 7 | GO:0021527 | 0.128 |
| endoplasmic reticulum unfolded protein response | 27 | GO:0030968 | 0.128 |
| cellular ketone body metabolic process | 5 | GO:0046950 | 0.128 |
| ketone body catabolic process | 5 | GO:0046952 | 0.128 |
| regulation of defense response to virus by virus | 28 | GO:0050690 | 0.128 |
| cellular response to interleukin-6 | 5 | GO:0071354 | 0.128 |
| response to oxidative stress | 134 | GO:0006979 | 0.129 |
| positive regulation of NF-kappaB import into nucleus | 17 | GO:0042346 | 0.129 |
| establishment of epithelial cell apical/basal polarity | 5 | GO:0045198 | 0.129 |
| pyrimidine nucleoside biosynthetic process | 6 | GO:0046134 | 0.129 |
| response to yeast | 8 | GO:0001878 | 0.13 |
| hyperosmotic response | 11 | GO:0006972 | 0.13 |
| skeletal muscle tissue growth | 5 | GO:0048630 | 0.13 |
| muscle organ morphogenesis | 8 | GO:0048644 | 0.13 |
| positive regulation of T cell activation | 19 | GO:0050870 | 0.13 |
| cellular homeostasis | 10 | GO:0019725 | 0.131 |
| positive regulation of interleukin-2 production | 9 | GO:0032743 | 0.131 |
| regulation of neuron apoptosis | 11 | GO:0043523 | 0.131 |
| placenta blood vessel development | 5 | GO:0060674 | 0.131 |
| biphenyl metabolic process | 8 | GO:0018879 | 0.133 |
| second-messenger-mediated signaling | 6 | GO:0019932 | 0.133 |
| regulation of B cell differentiation | 5 | GO:0045577 | 0.133 |
| behavioral response to ethanol | 5 | GO:0048149 | 0.133 |
| amino acid transport | 44 | GO:0006865 | 0.134 |
| positive regulation of programmed cell death | 6 | GO:0043068 | 0.134 |
| positive regulation of chemokine biosynthetic process | 9 | GO:0045080 | 0.134 |
| angiogenesis involved in wound healing | 6 | GO:0060055 | 0.134 |
| cellular response to exogenous dsRNA | 6 | GO:0071360 | 0.134 |
| S phase of mitotic cell cycle | 104 | GO:0000084 | 0.135 |
| adhesion to symbiont | 8 | GO:0051856 | 0.135 |
| oxaloacetate metabolic process | 11 | GO:0006107 | 0.136 |
| cellular response to oxidative stress | 21 | GO:0034599 | 0.136 |
| amino acid transmembrane transport | 42 | GO:0003333 | 0.137 |
| positive regulation of corticotropin secretion | 5 | GO:0051461 | 0.137 |
| carbohydrate phosphorylation | 13 | GO:0046835 | 0.138 |
| phototransduction, visible light | 8 | GO:0007603 | 0.139 |
| cellular chloride ion homeostasis | 8 | GO:0030644 | 0.139 |
| gene-specific transcription from RNA polymerase II promoter | 5 | GO:0032569 | 0.139 |
| phototransduction | 29 | GO:0007602 | 0.14 |
| regulation of calcium ion transport via voltage-gated calcium channel activity | 32 | GO:0051925 | 0.14 |
| regulation of protein catabolic process | 14 | GO:0042176 | 0.141 |
| ribosome biogenesis | 56 | GO:0042254 | 0.141 |
| cell differentiation involved in embryonic placenta development | 6 | GO:0060706 | 0.141 |
| neuronal action potential propagation | 6 | GO:0019227 | 0.143 |
| startle response | 12 | GO:0001964 | 0.144 |
| cellular component movement | 113 | GO:0006928 | 0.144 |
| oxygen homeostasis | 6 | GO:0032364 | 0.144 |
| B cell activation | 27 | GO:0042113 | 0.144 |
| endosome transport | 52 | GO:0016197 | 0.145 |
| negative regulation of growth of symbiont in host | 17 | GO:0044130 | 0.145 |
| positive regulation of monocyte chemotaxis | 6 | GO:0090026 | 0.145 |
| nucleotide metabolic process | 29 | GO:0009117 | 0.146 |
| response to gamma radiation | 31 | GO:0010332 | 0.146 |
| metanephric epithelium development | 5 | GO:0072207 | 0.146 |
| eye development | 27 | GO:0001654 | 0.147 |
| defense response to protozoan | 14 | GO:0042832 | 0.147 |
| peptide catabolic process | 6 | GO:0043171 | 0.147 |
| homeostasis of number of cells within a tissue | 19 | GO:0048873 | 0.147 |
| cranial suture morphogenesis | 5 | GO:0060363 | 0.147 |
| zinc ion transport | 26 | GO:0006829 | 0.148 |
| positive regulation of proteasomal ubiquitin-dependent protein catabolic process | 29 | GO:0032436 | 0.148 |
| response to muramyl dipeptide | 9 | GO:0032495 | 0.148 |
| chylomicron remnant clearance | 6 | GO:0034382 | 0.148 |
| histone H4-R3 methylation | 5 | GO:0043985 | 0.148 |
| epithelial cell differentiation involved in prostate gland development | 5 | GO:0060742 | 0.148 |
| prostanoid metabolic process | 12 | GO:0006692 | 0.15 |
| positive regulation of glycoprotein biosynthetic process | 5 | GO:0010560 | 0.15 |
| cell wall macromolecule catabolic process | 13 | GO:0016998 | 0.15 |
| regulation of meiosis | 5 | GO:0040020 | 0.15 |
| Peyer's patch development | 8 | GO:0048541 | 0.15 |
| response to reactive oxygen species | 20 | GO:0000302 | 0.151 |
| response to toxin | 98 | GO:0009636 | 0.151 |
| estrogen biosynthetic process | 9 | GO:0006703 | 0.152 |
| positive regulation of tissue remodeling | 6 | GO:0034105 | 0.153 |
| glucose homeostasis | 59 | GO:0042593 | 0.153 |
| response to cold | 37 | GO:0009409 | 0.154 |
| response to selenium ion | 13 | GO:0010269 | 0.154 |
| cellular response to nutrient | 7 | GO:0031670 | 0.154 |
| positive regulation of cell differentiation | 34 | GO:0045597 | 0.155 |
| positive regulation of JNK cascade | 41 | GO:0046330 | 0.155 |
| cellular senescence | 9 | GO:0090398 | 0.155 |
| regulation of respiratory gaseous exchange | 6 | GO:0043576 | 0.156 |
| negative regulation of dendrite morphogenesis | 5 | GO:0050774 | 0.156 |
| positive regulation of cytokine production | 13 | GO:0001819 | 0.157 |
| response to activity | 41 | GO:0014823 | 0.157 |
| cellular phosphate ion homeostasis | 6 | GO:0030643 | 0.157 |
| regulation of ion transmembrane transport | 114 | GO:0034765 | 0.157 |
| regulation of caspase activity | 10 | GO:0043281 | 0.157 |
| cellular response to ionizing radiation | 8 | GO:0071479 | 0.157 |
| response to superoxide | 6 | GO:0000303 | 0.158 |
| actin polymerization or depolymerization | 12 | GO:0008154 | 0.158 |
| cell morphogenesis involved in neuron differentiation | 8 | GO:0048667 | 0.158 |
| cellular response to metal ion | 6 | GO:0071248 | 0.158 |
| cellular response to cAMP | 19 | GO:0071320 | 0.158 |
| glucose 6-phosphate metabolic process | 8 | GO:0051156 | 0.159 |
| calcium ion transport | 127 | GO:0006816 | 0.16 |
| I-kappaB kinase/NF-kappaB cascade | 43 | GO:0007249 | 0.16 |
| oligosaccharide biosynthetic process | 10 | GO:0009312 | 0.16 |
| regulation of blood vessel size | 19 | GO:0050880 | 0.16 |
| glutamate biosynthetic process | 5 | GO:0006537 | 0.161 |
| positive regulation of receptor-mediated endocytosis | 17 | GO:0048260 | 0.161 |
| positive regulation of T-helper 1 type immune response | 8 | GO:0002827 | 0.162 |
| detection of bacterium | 9 | GO:0016045 | 0.163 |
| response to caffeine | 14 | GO:0031000 | 0.163 |
| follicle-stimulating hormone signaling pathway | 6 | GO:0042699 | 0.163 |
| chronic inflammatory response | 11 | GO:0002544 | 0.164 |
| positive regulation of immunoglobulin production | 7 | GO:0002639 | 0.164 |
| L-glutamate transport | 14 | GO:0015813 | 0.164 |
| T cell homeostasis | 20 | GO:0043029 | 0.164 |
| response to peptide hormone stimulus | 118 | GO:0043434 | 0.164 |
| L-glutamate import | 6 | GO:0051938 | 0.164 |
| response to hypoxia | 225 | GO:0001666 | 0.165 |
| astrocyte development | 7 | GO:0014002 | 0.165 |
| response to peptidoglycan | 8 | GO:0032494 | 0.165 |
| response to other organism | 5 | GO:0051707 | 0.165 |
| branching involved in embryonic placenta morphogenesis | 8 | GO:0060670 | 0.165 |
| androgen metabolic process | 14 | GO:0008209 | 0.166 |
| fever generation | 6 | GO:0001660 | 0.167 |
| response to magnesium ion | 17 | GO:0032026 | 0.167 |
| positive regulation of chemotaxis | 7 | GO:0050921 | 0.168 |
| regulation of immunoglobulin secretion | 5 | GO:0051023 | 0.168 |
| positive regulation of T cell cytokine production | 7 | GO:0002726 | 0.17 |
| phagocytosis, recognition | 8 | GO:0006910 | 0.17 |
| plasma membrane organization | 5 | GO:0007009 | 0.17 |
| activation of NF-kappaB-inducing kinase activity | 15 | GO:0007250 | 0.17 |
| regulation of calcium ion-dependent exocytosis | 11 | GO:0017158 | 0.17 |
| urea cycle | 11 | GO:0000050 | 0.171 |
| dicarboxylic acid transport | 9 | GO:0006835 | 0.171 |
| thyroid hormone generation | 10 | GO:0006590 | 0.172 |
| response to vitamin E | 17 | GO:0033197 | 0.172 |
| protein hexamerization | 5 | GO:0034214 | 0.172 |
| lateral line nerve glial cell development | 6 | GO:0048937 | 0.172 |
| positive regulation of lymphocyte proliferation | 7 | GO:0050671 | 0.172 |
| positive regulation of protein tyrosine kinase activity | 13 | GO:0061098 | 0.172 |
| cell killing | 5 | GO:0001906 | 0.173 |
| negative regulation of activated T cell proliferation | 6 | GO:0046007 | 0.173 |
| regulation of inflammatory response | 31 | GO:0050727 | 0.173 |
| regulation of transcription involved in G1/S phase of mitotic cell cycle | 17 | GO:0000083 | 0.174 |
| regulation of cholesterol biosynthetic process | 6 | GO:0045540 | 0.174 |
| positive regulation of histone phosphorylation | 6 | GO:0033129 | 0.175 |
| ATP metabolic process | 26 | GO:0046034 | 0.175 |
| succinyl-CoA metabolic process | 5 | GO:0006104 | 0.176 |
| activation of adenylate cyclase activity by G-protein signaling pathway | 46 | GO:0007189 | 0.176 |
| response to lithium ion | 17 | GO:0010226 | 0.176 |
| cellular response to glucose starvation | 9 | GO:0042149 | 0.176 |
| ribosome assembly | 6 | GO:0042255 | 0.177 |
| myeloid dendritic cell differentiation | 11 | GO:0043011 | 0.177 |
| response to cholesterol | 9 | GO:0070723 | 0.177 |
| positive regulation of granulocyte macrophage colony-stimulating factor production | 5 | GO:0032725 | 0.178 |
| negative regulation of ATPase activity | 5 | GO:0032780 | 0.178 |
| positive regulation of natural killer cell differentiation | 5 | GO:0032825 | 0.178 |
| iron ion transmembrane transport | 8 | GO:0034755 | 0.178 |
| regulation of mitochondrial membrane potential | 11 | GO:0051881 | 0.178 |
| glutathione biosynthetic process | 11 | GO:0006750 | 0.179 |
| response to biotic stimulus | 9 | GO:0009607 | 0.179 |
| protein localization to kinetochore | 5 | GO:0034501 | 0.179 |
| response to virus | 123 | GO:0009615 | 0.18 |
| negative regulation of interleukin-1 beta production | 6 | GO:0032691 | 0.18 |
| negative regulation of immune response | 10 | GO:0050777 | 0.18 |
| methionine biosynthetic process | 11 | GO:0009086 | 0.181 |
| regulation of proton transport | 6 | GO:0010155 | 0.181 |
| negative regulation of inflammatory response | 37 | GO:0050728 | 0.181 |
| neuromuscular process | 16 | GO:0050905 | 0.181 |
| attachment of spindle microtubules to kinetochore | 5 | GO:0008608 | 0.182 |
| macrophage differentiation | 11 | GO:0030225 | 0.182 |
| regulation of proteasomal protein catabolic process | 8 | GO:0061136 | 0.182 |
| cell cycle checkpoint | 123 | GO:0000075 | 0.184 |
| vesicle fusion with Golgi apparatus | 5 | GO:0048280 | 0.184 |
| negative regulation of cytokine secretion | 10 | GO:0050710 | 0.184 |
| progesterone biosynthetic process | 5 | GO:0006701 | 0.185 |
| mesoderm development | 35 | GO:0007498 | 0.185 |
| epidermis development | 86 | GO:0008544 | 0.185 |
| glycine transport | 7 | GO:0015816 | 0.185 |
| transmission of nerve impulse | 17 | GO:0019226 | 0.185 |
| keratinocyte differentiation | 46 | GO:0030216 | 0.185 |
| transepithelial chloride transport | 6 | GO:0030321 | 0.185 |
| postreplication repair | 7 | GO:0006301 | 0.186 |
| iron ion transport | 28 | GO:0006826 | 0.186 |
| positive regulation of activation of JAK2 kinase activity | 6 | GO:0010535 | 0.186 |
| positive regulation of cytokine secretion | 19 | GO:0050715 | 0.186 |
| defense response to fungus | 12 | GO:0050832 | 0.186 |
| respiratory chain complex IV assembly | 7 | GO:0008535 | 0.188 |
| positive regulation of cAMP biosynthetic process | 30 | GO:0030819 | 0.188 |
| Golgi to plasma membrane protein transport | 7 | GO:0043001 | 0.188 |
| retina morphogenesis in camera-type eye | 8 | GO:0060042 | 0.188 |
| death | 9 | GO:0016265 | 0.189 |
| response to vitamin D | 21 | GO:0033280 | 0.19 |
| response to lipid | 21 | GO:0033993 | 0.19 |
| response to nicotine | 38 | GO:0035094 | 0.19 |
| regulation of defense response to virus by host | 5 | GO:0050691 | 0.19 |
| aspartate transport | 5 | GO:0015810 | 0.191 |
| iron ion homeostasis | 22 | GO:0055072 | 0.191 |
| positive regulation of interleukin-23 production | 5 | GO:0032747 | 0.192 |
| sympathetic nervous system development | 16 | GO:0048485 | 0.192 |
| chondrocyte differentiation | 23 | GO:0002062 | 0.193 |
| response to X-ray | 23 | GO:0010165 | 0.193 |
| neuron fate commitment | 27 | GO:0048663 | 0.193 |
| response to temperature stimulus | 11 | GO:0009266 | 0.194 |
| negative regulation of stress-activated MAPK cascade | 5 | GO:0032873 | 0.194 |
| adult heart development | 13 | GO:0007512 | 0.195 |
| oxygen transport | 9 | GO:0015671 | 0.195 |
| vitamin D metabolic process | 12 | GO:0042359 | 0.195 |
| S-adenosylmethionine metabolic process | 9 | GO:0046500 | 0.195 |
| regulation of calcium ion transport | 14 | GO:0051924 | 0.195 |
| forebrain anterior/posterior pattern formation | 5 | GO:0021797 | 0.196 |
| serotonin metabolic process | 7 | GO:0042428 | 0.196 |
| transcytosis | 7 | GO:0045056 | 0.198 |
| positive regulation of NF-kappaB transcription factor activity | 82 | GO:0051092 | 0.198 |
| estrous cycle phase | 9 | GO:0060206 | 0.198 |
| cellular response to chemical stimulus | 5 | GO:0070887 | 0.198 |
| embryonic camera-type eye development | 9 | GO:0031076 | 0.199 |
| response to food | 21 | GO:0032094 | 0.199 |
| regulation of mitochondrial membrane permeability | 14 | GO:0046902 | 0.199 |
| forebrain morphogenesis | 7 | GO:0048853 | 0.199 |
| regulation of I-kappaB kinase/NF-kappaB cascade | 9 | GO:0043122 | 0.2 |
| microvillus assembly | 6 | GO:0030033 | 0.201 |
| B cell differentiation | 43 | GO:0030183 | 0.201 |
| dopamine metabolic process | 16 | GO:0042417 | 0.202 |
| arachidonic acid secretion | 13 | GO:0050482 | 0.202 |
| neutral amino acid transport | 14 | GO:0015804 | 0.203 |
| sterol metabolic process | 6 | GO:0016125 | 0.203 |
| positive regulation of Ras GTPase activity | 17 | GO:0032320 | 0.203 |
| nucleosome assembly | 66 | GO:0006334 | 0.204 |
| response to nutrient levels | 63 | GO:0031667 | 0.204 |
| tissue regeneration | 33 | GO:0042246 | 0.204 |
| neural retina development | 5 | GO:0003407 | 0.205 |
| protein geranylgeranylation | 5 | GO:0018344 | 0.205 |
| NAD metabolic process | 12 | GO:0019674 | 0.205 |
| cGMP-mediated signaling | 6 | GO:0019934 | 0.205 |
| positive regulation of natural killer cell activation | 9 | GO:0032816 | 0.205 |
| transferrin transport | 30 | GO:0033572 | 0.205 |
| tail morphogenesis | 16 | GO:0035121 | 0.205 |
| centrosome duplication | 7 | GO:0051298 | 0.205 |
| positive regulation of T cell chemotaxis | 9 | GO:0010820 | 0.206 |
| neuron fate specification | 8 | GO:0048665 | 0.206 |
| response to glucagon stimulus | 12 | GO:0033762 | 0.207 |
| regulation of T cell differentiation | 5 | GO:0045580 | 0.207 |
| histamine secretion | 5 | GO:0001821 | 0.208 |
| polyamine biosynthetic process | 7 | GO:0006596 | 0.208 |
| negative regulation of astrocyte differentiation | 10 | GO:0048712 | 0.209 |
| regulation of type I interferon-mediated signaling pathway | 18 | GO:0060338 | 0.209 |
| viral transcription | 76 | GO:0019083 | 0.21 |
| regulation of actin filament polymerization | 15 | GO:0030833 | 0.21 |
| response to insulin stimulus | 75 | GO:0032868 | 0.21 |
| UDP-N-acetylglucosamine biosynthetic process | 5 | GO:0006048 | 0.211 |
| isotype switching | 13 | GO:0045190 | 0.211 |
| diet induced thermogenesis | 5 | GO:0002024 | 0.212 |
| response to manganese ion | 11 | GO:0010042 | 0.212 |
| cellular response to extracellular stimulus | 19 | GO:0031668 | 0.212 |
| cellular response to stress | 10 | GO:0033554 | 0.212 |
| astrocyte cell migration | 5 | GO:0043615 | 0.212 |
| protein-chromophore linkage | 15 | GO:0018298 | 0.213 |
| positive regulation of glucokinase activity | 5 | GO:0033133 | 0.213 |
| response to L-ascorbic acid | 12 | GO:0033591 | 0.213 |
| hemoglobin biosynthetic process | 6 | GO:0042541 | 0.213 |
| lactate metabolic process | 5 | GO:0006089 | 0.214 |
| NADH metabolic process | 9 | GO:0006734 | 0.215 |
| fusion of sperm to egg plasma membrane | 8 | GO:0007342 | 0.215 |
| copper ion transmembrane transport | 5 | GO:0035434 | 0.215 |
| negative regulation of urine volume | 6 | GO:0035811 | 0.215 |
| sphingosine metabolic process | 5 | GO:0006670 | 0.217 |
| midbrain-hindbrain boundary development | 7 | GO:0030917 | 0.217 |
| response to morphine | 27 | GO:0043278 | 0.217 |
| adult locomotory behavior | 40 | GO:0008344 | 0.218 |
| positive regulation of calcium ion transport into cytosol | 15 | GO:0010524 | 0.218 |
| response to progesterone stimulus | 41 | GO:0032570 | 0.218 |
| Sertoli cell development | 9 | GO:0060009 | 0.218 |
| positive regulation of vasoconstriction | 25 | GO:0045907 | 0.219 |
| regulation of axonogenesis | 22 | GO:0050770 | 0.219 |
| cytosolic calcium ion homeostasis | 16 | GO:0051480 | 0.219 |
| ventricular septum morphogenesis | 8 | GO:0060412 | 0.219 |
| activation of MAPKKK activity | 7 | GO:0000185 | 0.22 |
| UMP biosynthetic process | 5 | GO:0006222 | 0.22 |
| glial cell migration | 7 | GO:0008347 | 0.22 |
| positive regulation of heart rate | 11 | GO:0010460 | 0.22 |
| L-serine transport | 7 | GO:0015825 | 0.22 |
| osteoclast differentiation | 16 | GO:0030316 | 0.22 |
| neurotransmitter metabolic process | 6 | GO:0042133 | 0.22 |
| positive regulation of erythrocyte differentiation | 14 | GO:0045648 | 0.22 |
| embryonic cranial skeleton morphogenesis | 23 | GO:0048701 | 0.22 |
| skeletal muscle fiber development | 14 | GO:0048741 | 0.22 |
| sensory perception of taste | 36 | GO:0050909 | 0.22 |
| protein monoubiquitination | 9 | GO:0006513 | 0.221 |
| ER-associated protein catabolic process | 32 | GO:0030433 | 0.221 |
| positive regulation of prostaglandin secretion | 8 | GO:0032308 | 0.221 |
| catecholamine biosynthetic process | 7 | GO:0042423 | 0.221 |
| spindle organization | 16 | GO:0007051 | 0.222 |
| cell recognition | 11 | GO:0008037 | 0.222 |
| isoprenoid biosynthetic process | 15 | GO:0008299 | 0.222 |
| peptide transport | 7 | GO:0015833 | 0.222 |
| negative regulation of B cell proliferation | 13 | GO:0030889 | 0.223 |
| regulation of inhibitory postsynaptic membrane potential | 11 | GO:0060080 | 0.224 |
| positive regulation of endothelial cell proliferation | 45 | GO:0001938 | 0.226 |
| neuron-neuron synaptic transmission | 8 | GO:0007270 | 0.227 |
| ISG15-protein conjugation | 7 | GO:0032020 | 0.227 |
| neutrophil mediated immunity | 8 | GO:0002446 | 0.228 |
| organic cation transport | 11 | GO:0015695 | 0.228 |
| cerebellum development | 26 | GO:0021549 | 0.228 |
| maternal behavior | 7 | GO:0042711 | 0.228 |
| soft palate development | 5 | GO:0060023 | 0.228 |
| autophagy | 49 | GO:0006914 | 0.229 |
| positive regulation of cholesterol esterification | 7 | GO:0010873 | 0.229 |
| metal ion transport | 23 | GO:0030001 | 0.229 |
| 3'-UTR-mediated mRNA stabilization | 5 | GO:0070935 | 0.229 |
| positive regulation of release of cytochrome c from mitochondria | 11 | GO:0090200 | 0.229 |
| retinoid metabolic process | 11 | GO:0001523 | 0.23 |
| glutamate signaling pathway | 14 | GO:0007215 | 0.23 |
| L-amino acid transport | 17 | GO:0015807 | 0.23 |
| negative regulation of caspase activity | 48 | GO:0043154 | 0.23 |
| regulation of anion transport | 8 | GO:0044070 | 0.23 |
| social behavior | 29 | GO:0035176 | 0.231 |
| mitochondrial fusion | 7 | GO:0008053 | 0.232 |
| negative regulation of macrophage derived foam cell differentiation | 13 | GO:0010745 | 0.232 |
| musculoskeletal movement | 6 | GO:0050881 | 0.232 |
| germinal center formation | 7 | GO:0002467 | 0.233 |
| triglyceride metabolic process | 37 | GO:0006641 | 0.233 |
| post-chaperonin tubulin folding pathway | 5 | GO:0007023 | 0.233 |
| intermediate filament organization | 14 | GO:0045109 | 0.233 |
| response to electrical stimulus | 28 | GO:0051602 | 0.233 |
| response to fungicide | 14 | GO:0060992 | 0.233 |
| hemopoietic progenitor cell differentiation | 9 | GO:0002244 | 0.235 |
| organic acid metabolic process | 9 | GO:0006082 | 0.235 |
| neurogenesis | 30 | GO:0022008 | 0.236 |
| regulation of excitatory postsynaptic membrane potential | 30 | GO:0060079 | 0.236 |
| regulation of DNA binding | 6 | GO:0051101 | 0.237 |
| positive regulation of release of sequestered calcium ion into cytosol | 15 | GO:0051281 | 0.237 |
| glucose metabolic process | 108 | GO:0006006 | 0.238 |
| translational termination | 82 | GO:0006415 | 0.238 |
| positive regulation of protein homooligomerization | 7 | GO:0032464 | 0.238 |
| behavioral response to cocaine | 8 | GO:0048148 | 0.239 |
| cellular copper ion homeostasis | 12 | GO:0006878 | 0.24 |
| positive regulation of autophagy | 5 | GO:0010508 | 0.24 |
| midbrain development | 30 | GO:0030901 | 0.24 |
| nucleotide-sugar transport | 6 | GO:0015780 | 0.241 |
| cellular response to hydrogen peroxide | 28 | GO:0070301 | 0.241 |
| viral infectious cycle | 85 | GO:0019058 | 0.242 |
| tetrapyrrole biosynthetic process | 5 | GO:0033014 | 0.242 |
| cellular protein metabolic process | 275 | GO:0044267 | 0.242 |
| replicative cell aging | 5 | GO:0001302 | 0.243 |
| protein K63-linked deubiquitination | 9 | GO:0070536 | 0.243 |
| calcium-mediated signaling | 39 | GO:0019722 | 0.244 |
| negative regulation of telomere maintenance via telomerase | 5 | GO:0032211 | 0.244 |
| glycerol metabolic process | 17 | GO:0006071 | 0.245 |
| response to endoplasmic reticulum stress | 13 | GO:0034976 | 0.245 |
| gland development | 5 | GO:0048732 | 0.245 |
| activation of anaphase-promoting complex activity | 5 | GO:0051488 | 0.245 |
| acetyl-CoA biosynthetic process | 5 | GO:0006085 | 0.246 |
| one-carbon metabolic process | 30 | GO:0006730 | 0.246 |
| insulin secretion | 32 | GO:0030073 | 0.246 |
| beta-amyloid metabolic process | 6 | GO:0050435 | 0.246 |
| mitotic recombination | 6 | GO:0006312 | 0.247 |
| positive regulation of mast cell degranulation | 9 | GO:0043306 | 0.247 |
| pathogenesis | 7 | GO:0009405 | 0.248 |
| forebrain neuron differentiation | 10 | GO:0021879 | 0.248 |
| C21-steroid hormone biosynthetic process | 7 | GO:0006700 | 0.249 |
| response to iron(II) ion | 5 | GO:0010040 | 0.249 |
| negative regulation of myeloid cell differentiation | 5 | GO:0045638 | 0.249 |
| autophagic cell death | 7 | GO:0048102 | 0.249 |
| regulation of cytokine production | 6 | GO:0001817 | 0.25 |
| regulation of receptor recycling | 6 | GO:0001919 | 0.25 |
| central nervous system neuron differentiation | 10 | GO:0021953 | 0.25 |
| type I interferon biosynthetic process | 5 | GO:0045351 | 0.25 |
| nerve development | 11 | GO:0021675 | 0.251 |
| positive regulation of acute inflammatory response | 11 | GO:0002675 | 0.252 |
| androgen biosynthetic process | 13 | GO:0006702 | 0.253 |
| neutrophil degranulation | 5 | GO:0043312 | 0.253 |
| positive regulation of sequestering of triglyceride | 5 | GO:0010890 | 0.254 |
| hyaluronan metabolic process | 7 | GO:0030212 | 0.254 |
| positive regulation of smooth muscle contraction | 17 | GO:0045987 | 0.254 |
| mitochondrial genome maintenance | 7 | GO:0000002 | 0.255 |
| DNA recombinase assembly | 5 | GO:0000730 | 0.255 |
| purine base biosynthetic process | 5 | GO:0009113 | 0.255 |
| synaptic transmission, glutamatergic | 28 | GO:0035249 | 0.255 |
| choline transport | 9 | GO:0015871 | 0.256 |
| cellular sodium ion homeostasis | 9 | GO:0006883 | 0.257 |
| positive regulation of insulin receptor signaling pathway | 5 | GO:0046628 | 0.257 |
| endoderm formation | 10 | GO:0001706 | 0.258 |
| regulation of cell size | 11 | GO:0008361 | 0.258 |
| positive regulation of natural killer cell proliferation | 6 | GO:0032819 | 0.258 |
| monocyte activation | 6 | GO:0042117 | 0.258 |
| mRNA stabilization | 13 | GO:0048255 | 0.258 |
| cell activation | 8 | GO:0001775 | 0.259 |
| positive regulation of receptor internalization | 7 | GO:0002092 | 0.259 |
| leukocyte migration involved in inflammatory response | 7 | GO:0002523 | 0.259 |
| axonal fasciculation | 13 | GO:0007413 | 0.259 |
| response to methotrexate | 5 | GO:0031427 | 0.259 |
| negative regulation of type I interferon production | 30 | GO:0032480 | 0.259 |
| regulation of dopamine metabolic process | 7 | GO:0042053 | 0.259 |
| glyoxylate metabolic process | 5 | GO:0046487 | 0.259 |
| chondrocyte development | 10 | GO:0002063 | 0.26 |
| xenobiotic metabolic process | 121 | GO:0006805 | 0.26 |
| excretion | 40 | GO:0007588 | 0.26 |
| regulation of heart contraction | 41 | GO:0008016 | 0.26 |
| chaperone-mediated protein complex assembly | 5 | GO:0051131 | 0.26 |
| regulation of gluconeogenesis | 5 | GO:0006111 | 0.261 |
| regulation of ossification | 13 | GO:0030278 | 0.262 |
| very-low-density lipoprotein particle assembly | 7 | GO:0034379 | 0.262 |
| ER overload response | 9 | GO:0006983 | 0.264 |
| positive regulation of protein kinase A signaling cascade | 5 | GO:0010739 | 0.264 |
| positive regulation of Cdc42 GTPase activity | 5 | GO:0043089 | 0.264 |
| prostaglandin metabolic process | 9 | GO:0006693 | 0.265 |
| anatomical structure morphogenesis | 101 | GO:0009653 | 0.266 |
| long-chain fatty-acyl-CoA biosynthetic process | 17 | GO:0035338 | 0.266 |
| folic acid metabolic process | 9 | GO:0046655 | 0.266 |
| cellular response to organic cyclic compound | 35 | GO:0071407 | 0.266 |
| lens development in camera-type eye | 24 | GO:0002088 | 0.267 |
| hydrogen peroxide catabolic process | 19 | GO:0042744 | 0.267 |
| intestinal absorption | 8 | GO:0050892 | 0.267 |
| nucleobase, nucleoside, nucleotide and nucleic acid metabolic process | 67 | GO:0006139 | 0.268 |
| blood coagulation | 440 | GO:0007596 | 0.268 |
| aromatic amino acid family metabolic process | 10 | GO:0009072 | 0.268 |
| glucose 1-phosphate metabolic process | 8 | GO:0019255 | 0.269 |
| response to light stimulus | 24 | GO:0009416 | 0.27 |
| ubiquinone biosynthetic process | 9 | GO:0006744 | 0.271 |
| regulation of synaptogenesis | 6 | GO:0051963 | 0.271 |
| synaptic transmission | 349 | GO:0007268 | 0.272 |
| telomere maintenance via semi-conservative replication | 21 | GO:0032201 | 0.272 |
| negative regulation of ERK1 and ERK2 cascade | 15 | GO:0070373 | 0.272 |
| pro-B cell differentiation | 5 | GO:0002328 | 0.273 |
| provirus integration | 8 | GO:0019047 | 0.273 |
| peristalsis | 9 | GO:0030432 | 0.273 |
| negative regulation of microtubule polymerization | 8 | GO:0031115 | 0.273 |
| regulation of norepinephrine secretion | 5 | GO:0014061 | 0.274 |
| otic vesicle formation | 8 | GO:0030916 | 0.274 |
| negative regulation of DNA binding | 17 | GO:0043392 | 0.274 |
| actin filament severing | 6 | GO:0051014 | 0.274 |
| protein kinase C signaling cascade | 6 | GO:0070528 | 0.274 |
| erythrocyte maturation | 9 | GO:0043249 | 0.275 |
| cellular response to calcium ion | 11 | GO:0071277 | 0.275 |
| thyroid hormone metabolic process | 12 | GO:0042403 | 0.276 |
| anagen | 10 | GO:0042640 | 0.276 |
| creatine metabolic process | 9 | GO:0006600 | 0.277 |
| rRNA modification | 5 | GO:0000154 | 0.278 |
| acyl-CoA metabolic process | 22 | GO:0006637 | 0.279 |
| central nervous system neuron development | 12 | GO:0021954 | 0.279 |
| negative regulation of collagen biosynthetic process | 5 | GO:0032966 | 0.279 |
| regulation of S phase of mitotic cell cycle | 8 | GO:0007090 | 0.281 |
| leukocyte tethering or rolling | 8 | GO:0050901 | 0.281 |
| regulation of sensory perception of pain | 26 | GO:0051930 | 0.281 |
| purine nucleotide biosynthetic process | 15 | GO:0006164 | 0.282 |
| DNA unwinding involved in replication | 12 | GO:0006268 | 0.282 |
| positive regulation of insulin-like growth factor receptor signaling pathway | 12 | GO:0043568 | 0.282 |
| single strand break repair | 8 | GO:0000012 | 0.283 |
| post-translational protein modification | 160 | GO:0043687 | 0.283 |
| cellular response to estrogen stimulus | 6 | GO:0071391 | 0.283 |
| response to external stimulus | 9 | GO:0009605 | 0.284 |
| mitotic cell cycle G1/S transition DNA damage checkpoint | 9 | GO:0031571 | 0.284 |
| translational elongation | 103 | GO:0006414 | 0.285 |
| entrainment of circadian clock | 6 | GO:0009649 | 0.285 |
| negative regulation of cardiac muscle hypertrophy | 6 | GO:0010614 | 0.285 |
| heart trabecula formation | 9 | GO:0060347 | 0.285 |
| response to stilbenoid | 7 | GO:0035634 | 0.286 |
| positive regulation of G-protein coupled receptor protein signaling pathway | 9 | GO:0045745 | 0.286 |
| regulation of short-term neuronal synaptic plasticity | 9 | GO:0048172 | 0.286 |
| nucleotide-excision repair, DNA gap filling | 19 | GO:0006297 | 0.287 |
| sex determination | 9 | GO:0007530 | 0.287 |
| urea transport | 6 | GO:0015840 | 0.287 |
| ameboidal cell migration | 5 | GO:0001667 | 0.288 |
| tRNA splicing, via endonucleolytic cleavage and ligation | 6 | GO:0006388 | 0.288 |
| mitotic cell cycle G2/M transition DNA damage checkpoint | 6 | GO:0007095 | 0.288 |
| motor axon guidance | 18 | GO:0008045 | 0.288 |
| spliceosomal snRNP assembly | 24 | GO:0000387 | 0.29 |
| phospholipid transport | 24 | GO:0015914 | 0.29 |
| positive regulation of interleukin-17 production | 10 | GO:0032740 | 0.29 |
| prostate epithelial cord arborization involved in prostate glandular acinus morphogenesis | 7 | GO:0060527 | 0.29 |
| tissue development | 31 | GO:0009888 | 0.291 |
| cell projection assembly | 9 | GO:0030031 | 0.291 |
| STAT protein import into nucleus | 5 | GO:0007262 | 0.293 |
| cerebellar Purkinje cell differentiation | 9 | GO:0021702 | 0.293 |
| telencephalon cell migration | 7 | GO:0022029 | 0.293 |
| adult walking behavior | 32 | GO:0007628 | 0.295 |
| negative regulation of interleukin-4 production | 5 | GO:0032713 | 0.295 |
| positive regulation of defense response to virus by host | 11 | GO:0002230 | 0.296 |
| pinocytosis | 5 | GO:0006907 | 0.296 |
| JAK-STAT cascade | 33 | GO:0007259 | 0.296 |
| parturition | 11 | GO:0007567 | 0.296 |
| initiation of viral infection | 12 | GO:0019059 | 0.296 |
| response to pyrethroid | 5 | GO:0046684 | 0.296 |
| glutathione metabolic process | 31 | GO:0006749 | 0.297 |
| synaptic vesicle exocytosis | 20 | GO:0016079 | 0.297 |
| low-density lipoprotein particle remodeling | 10 | GO:0034374 | 0.297 |
| enteric nervous system development | 14 | GO:0048484 | 0.297 |
| long term synaptic depression | 8 | GO:0060292 | 0.297 |
| positive regulation of uterine smooth muscle contraction | 7 | GO:0070474 | 0.297 |
| endoplasmic reticulum organization | 12 | GO:0007029 | 0.298 |
| peptidyl-tyrosine dephosphorylation | 59 | GO:0035335 | 0.298 |
| bile acid biosynthetic process | 21 | GO:0006699 | 0.299 |
| cellular membrane organization | 97 | GO:0016044 | 0.299 |
| regulation of lipid metabolic process | 19 | GO:0019216 | 0.299 |
| plasminogen activation | 5 | GO:0031639 | 0.299 |
| fructose 1,6-bisphosphate metabolic process | 6 | GO:0030388 | 0.3 |
| response to estradiol stimulus | 130 | GO:0032355 | 0.3 |
| positive regulation of phosphorylation | 28 | GO:0042327 | 0.3 |
| chiasma assembly | 5 | GO:0051026 | 0.3 |
| positive regulation of I-kappaB kinase/NF-kappaB cascade | 134 | GO:0043123 | 0.301 |
| negative regulation of cell death | 23 | GO:0060548 | 0.302 |
| photoreceptor cell maintenance | 23 | GO:0045494 | 0.304 |
| regulation of timing of cell differentiation | 5 | GO:0048505 | 0.304 |
| negative regulation of cell cycle arrest | 7 | GO:0071157 | 0.306 |
| glutamine metabolic process | 15 | GO:0006541 | 0.307 |
| viral genome replication | 13 | GO:0019079 | 0.307 |
| myelination in peripheral nervous system | 7 | GO:0022011 | 0.307 |
| regulation of interferon-gamma-mediated signaling pathway | 16 | GO:0060334 | 0.307 |
| calcium ion transmembrane transport | 80 | GO:0070588 | 0.307 |
| triglyceride catabolic process | 19 | GO:0019433 | 0.308 |
| prostate gland development | 9 | GO:0030850 | 0.308 |
| positive regulation of estrogen receptor signaling pathway | 5 | GO:0033148 | 0.308 |
| regulation of cell proliferation | 139 | GO:0042127 | 0.308 |
| lipoprotein biosynthetic process | 6 | GO:0042158 | 0.309 |
| hydrogen peroxide metabolic process | 6 | GO:0042743 | 0.309 |
| negative regulation of synaptic transmission, glutamatergic | 8 | GO:0051967 | 0.309 |
| regulation of locomotion | 5 | GO:0040012 | 0.31 |
| negative regulation of calcium ion transport via voltage-gated calcium channel activity | 10 | GO:0051927 | 0.31 |
| tachykinin receptor signaling pathway | 8 | GO:0007217 | 0.313 |
| associative learning | 22 | GO:0008306 | 0.313 |
| macroautophagy | 6 | GO:0016236 | 0.313 |
| protein localization to organelle | 11 | GO:0033365 | 0.313 |
| regulation of protein stability | 20 | GO:0031647 | 0.314 |
| response to pain | 18 | GO:0048265 | 0.314 |
| digestive tract morphogenesis | 15 | GO:0048546 | 0.314 |
| glycoprotein metabolic process | 7 | GO:0009100 | 0.315 |
| negative regulation of interleukin-8 production | 5 | GO:0032717 | 0.315 |
| regulation of long-term neuronal synaptic plasticity | 30 | GO:0048169 | 0.315 |
| lung saccule development | 5 | GO:0060430 | 0.316 |
| calcium ion-dependent exocytosis | 19 | GO:0017156 | 0.317 |
| induction of apoptosis | 208 | GO:0006917 | 0.318 |
| outer ear morphogenesis | 6 | GO:0042473 | 0.318 |
| cellular response to fatty acid | 9 | GO:0071398 | 0.318 |
| proline transport | 7 | GO:0015824 | 0.319 |
| positive regulation of fatty acid biosynthetic process | 10 | GO:0045723 | 0.319 |
| detection of temperature stimulus involved in sensory perception of pain | 5 | GO:0050965 | 0.319 |
| negative regulation of type 2 immune response | 5 | GO:0002829 | 0.32 |
| ATP biosynthetic process | 57 | GO:0006754 | 0.32 |
| tetracycline transport | 6 | GO:0015904 | 0.321 |
| cell migration involved in gastrulation | 11 | GO:0042074 | 0.321 |
| oocyte maturation | 16 | GO:0001556 | 0.322 |
| complement activation, lectin pathway | 7 | GO:0001867 | 0.322 |
| monoamine transport | 9 | GO:0015844 | 0.323 |
| histone phosphorylation | 9 | GO:0016572 | 0.323 |
| axon extension involved in axon guidance | 9 | GO:0048846 | 0.323 |
| positive regulation of NK T cell activation | 5 | GO:0051135 | 0.323 |
| spinal cord motor neuron differentiation | 8 | GO:0021522 | 0.324 |
| signal transduction in response to DNA damage | 8 | GO:0042770 | 0.324 |
| positive regulation of calcium ion transport via voltage-gated calcium channel activity | 9 | GO:0051929 | 0.324 |
| embryonic skeletal joint morphogenesis | 9 | GO:0060272 | 0.324 |
| cAMP biosynthetic process | 16 | GO:0006171 | 0.325 |
| JUN phosphorylation | 5 | GO:0007258 | 0.325 |
| synaptic vesicle targeting | 5 | GO:0016080 | 0.325 |
| positive regulation of prostaglandin biosynthetic process | 5 | GO:0031394 | 0.326 |
| piRNA metabolic process | 8 | GO:0034587 | 0.326 |
| ncRNA metabolic process | 20 | GO:0034660 | 0.327 |
| cartilage development involved in endochondral bone morphogenesis | 8 | GO:0060351 | 0.327 |
| glycosphingolipid biosynthetic process | 10 | GO:0006688 | 0.328 |
| gland morphogenesis | 5 | GO:0022612 | 0.328 |
| regulation of neurotransmitter levels | 6 | GO:0001505 | 0.329 |
| mitochondrial electron transport, NADH to ubiquinone | 37 | GO:0006120 | 0.329 |
| double-strand break repair via nonhomologous end joining | 10 | GO:0006303 | 0.329 |
| female gonad development | 23 | GO:0008585 | 0.329 |
| mitochondrial ATP synthesis coupled proton transport | 13 | GO:0042776 | 0.329 |
| regulation of cardiac muscle contraction | 5 | GO:0055117 | 0.329 |
| tRNA aminoacylation for protein translation | 43 | GO:0006418 | 0.33 |
| positive regulation of tyrosine phosphorylation of Stat5 protein | 14 | GO:0042523 | 0.33 |
| thrombin receptor signaling pathway | 8 | GO:0070493 | 0.33 |
| conditioned taste aversion | 6 | GO:0001661 | 0.331 |
| peptidyl-arginine methylation, to asymmetrical-dimethyl arginine | 5 | GO:0019919 | 0.331 |
| negative regulation of calcium ion-dependent exocytosis | 5 | GO:0045955 | 0.331 |
| diacylglycerol biosynthetic process | 5 | GO:0006651 | 0.332 |
| GDP-mannose biosynthetic process | 5 | GO:0009298 | 0.332 |
| positive regulation of memory T cell differentiation | 5 | GO:0043382 | 0.332 |
| prepulse inhibition | 12 | GO:0060134 | 0.332 |
| branching involved in prostate gland morphogenesis | 7 | GO:0060442 | 0.332 |
| regulation of canonical Wnt receptor signaling pathway | 7 | GO:0060828 | 0.332 |
| response to estrogen stimulus | 91 | GO:0043627 | 0.334 |
| protein N-linked glycosylation via asparagine | 87 | GO:0018279 | 0.335 |
| epithelial cell proliferation involved in salivary gland morphogenesis | 7 | GO:0060664 | 0.335 |
| steroid metabolic process | 89 | GO:0008202 | 0.336 |
| glycoprotein biosynthetic process | 7 | GO:0009101 | 0.336 |
| DNA ligation involved in DNA repair | 6 | GO:0051103 | 0.337 |
| C-terminal protein lipidation | 25 | GO:0006501 | 0.338 |
| glycerol ether metabolic process | 14 | GO:0006662 | 0.338 |
| activation of adenylate cyclase activity | 33 | GO:0007190 | 0.338 |
| cellular response to cholesterol | 8 | GO:0071397 | 0.338 |
| DNA catabolic process | 5 | GO:0006308 | 0.339 |
| response to zinc ion | 34 | GO:0010043 | 0.339 |
| bone mineralization | 26 | GO:0030282 | 0.339 |
| positive regulation of tyrosine phosphorylation of Stat3 protein | 16 | GO:0042517 | 0.339 |
| positive regulation of cell cycle | 29 | GO:0045787 | 0.339 |
| protein maturation by peptide bond cleavage | 21 | GO:0051605 | 0.34 |
| pituitary gland development | 30 | GO:0021983 | 0.341 |
| lipoprotein transport | 13 | GO:0042953 | 0.341 |
| negative regulation of T cell receptor signaling pathway | 10 | GO:0050860 | 0.341 |
| glycosphingolipid metabolic process | 7 | GO:0006687 | 0.342 |
| response to fructose stimulus | 8 | GO:0009750 | 0.342 |
| response to vitamin A | 24 | GO:0033189 | 0.342 |
| natural killer cell mediated cytotoxicity | 6 | GO:0042267 | 0.342 |
| S-adenosylhomocysteine metabolic process | 12 | GO:0046498 | 0.342 |
| sleep | 12 | GO:0030431 | 0.343 |
| oxidation-reduction process | 582 | GO:0055114 | 0.344 |
| circadian rhythm | 59 | GO:0007623 | 0.345 |
| peptide hormone processing | 18 | GO:0016486 | 0.345 |
| positive regulation of dopamine secretion | 8 | GO:0033603 | 0.345 |
| negative regulation of oligodendrocyte differentiation | 10 | GO:0048715 | 0.345 |
| regulation of mitosis | 14 | GO:0007088 | 0.346 |
| ether lipid biosynthetic process | 5 | GO:0008611 | 0.346 |
| negative regulation of cholesterol storage | 6 | GO:0010887 | 0.346 |
| sodium ion export | 32 | GO:0071436 | 0.346 |
| centrosome cycle | 5 | GO:0007098 | 0.347 |
| aromatic compound catabolic process | 5 | GO:0019439 | 0.347 |
| protein homotrimerization | 16 | GO:0070207 | 0.347 |
| regulation of smooth muscle contraction | 14 | GO:0006940 | 0.348 |
| regulation of G2/M transition of mitotic cell cycle | 7 | GO:0010389 | 0.348 |
| positive regulation of gene-specific transcription | 68 | GO:0043193 | 0.348 |
| organ formation | 7 | GO:0048645 | 0.348 |
| positive regulation of oligodendrocyte differentiation | 10 | GO:0048714 | 0.348 |
| glutamate metabolic process | 11 | GO:0006536 | 0.349 |
| negative regulation of MAP kinase activity | 28 | GO:0043407 | 0.349 |
| apoptosis in response to endoplasmic reticulum stress | 9 | GO:0070059 | 0.349 |
| negative regulation of release of cytochrome c from mitochondria | 5 | GO:0090201 | 0.349 |
| DNA damage induced protein phosphorylation | 6 | GO:0006975 | 0.35 |
| protein destabilization | 15 | GO:0031648 | 0.35 |
| embryonic cleavage | 6 | GO:0040016 | 0.35 |
| potassium ion transport | 154 | GO:0006813 | 0.352 |
| negative regulation of T cell activation | 11 | GO:0050868 | 0.352 |
| positive regulation of cell division | 41 | GO:0051781 | 0.352 |
| mitochondrial membrane organization | 6 | GO:0007006 | 0.353 |
| negative regulation of transporter activity | 5 | GO:0032410 | 0.353 |
| organ development | 18 | GO:0048513 | 0.353 |
| negative regulation of fibrinolysis | 10 | GO:0051918 | 0.353 |
| generation of precursor metabolites and energy | 59 | GO:0006091 | 0.354 |
| lysine catabolic process | 8 | GO:0006554 | 0.354 |
| bile acid metabolic process | 33 | GO:0008206 | 0.354 |
| response to water deprivation | 5 | GO:0009414 | 0.354 |
| regulation of action potential in neuron | 19 | GO:0019228 | 0.354 |
| positive regulation of renal sodium excretion | 10 | GO:0035815 | 0.354 |
| axon cargo transport | 7 | GO:0008088 | 0.355 |
| regulation of protein secretion | 10 | GO:0050708 | 0.355 |
| glycine metabolic process | 6 | GO:0006544 | 0.356 |
| response to xenobiotic stimulus | 7 | GO:0009410 | 0.356 |
| regulation of double-strand break repair via homologous recombination | 6 | GO:0010569 | 0.356 |
| positive regulation of triglyceride biosynthetic process | 6 | GO:0010867 | 0.356 |
| regulation of protein heterodimerization activity | 6 | GO:0043497 | 0.356 |
| cellular protein catabolic process | 6 | GO:0044257 | 0.357 |
| replicative senescence | 8 | GO:0090399 | 0.357 |
| response to gravity | 12 | GO:0009629 | 0.359 |
| cAMP-mediated signaling | 18 | GO:0019933 | 0.359 |
| DNA damage response, signal transduction by p53 class mediator | 9 | GO:0030330 | 0.359 |
| monocyte differentiation | 7 | GO:0030224 | 0.361 |
| positive regulation of potassium ion transport | 8 | GO:0043268 | 0.361 |
| negative regulation of JUN kinase activity | 11 | GO:0043508 | 0.361 |
| cellular response to antibiotic | 11 | GO:0071236 | 0.361 |
| sterol transport | 6 | GO:0015918 | 0.362 |
| signal peptide processing | 8 | GO:0006465 | 0.363 |
| fatty acid catabolic process | 7 | GO:0009062 | 0.363 |
| negative regulation of interleukin-12 production | 9 | GO:0032695 | 0.363 |
| negative regulation of Notch signaling pathway | 9 | GO:0045746 | 0.363 |
| regulation of branching involved in salivary gland morphogenesis by mesenchymal-epithelial signaling | 5 | GO:0060665 | 0.363 |
| positive regulation of vasodilation | 22 | GO:0045909 | 0.364 |
| response to mineralocorticoid stimulus | 6 | GO:0051385 | 0.364 |
| purine nucleotide catabolic process | 11 | GO:0006195 | 0.365 |
| mRNA catabolic process | 15 | GO:0006402 | 0.365 |
| meiotic prophase I | 11 | GO:0007128 | 0.365 |
| heme metabolic process | 6 | GO:0042168 | 0.365 |
| glutamate secretion | 19 | GO:0014047 | 0.366 |
| response to UV-B | 9 | GO:0010224 | 0.367 |
| ciliary or flagellar motility | 15 | GO:0001539 | 0.368 |
| arginine catabolic process | 8 | GO:0006527 | 0.368 |
| quaternary ammonium group transport | 6 | GO:0015697 | 0.368 |
| response to steroid hormone stimulus | 44 | GO:0048545 | 0.37 |
| glycolipid transport | 6 | GO:0046836 | 0.371 |
| paraxial mesoderm formation | 5 | GO:0048341 | 0.371 |
| neurotransmitter secretion | 65 | GO:0007269 | 0.372 |
| drug metabolic process | 18 | GO:0017144 | 0.372 |
| ceramide biosynthetic process | 9 | GO:0046513 | 0.372 |
| cartilage condensation | 20 | GO:0001502 | 0.373 |
| protein export from nucleus | 19 | GO:0006611 | 0.373 |
| negative regulation of activin receptor signaling pathway | 5 | GO:0032926 | 0.373 |
| regulation of synapse structure and activity | 7 | GO:0050803 | 0.373 |
| lens fiber cell development | 9 | GO:0070307 | 0.373 |
| mitochondrion organization | 34 | GO:0007005 | 0.376 |
| keratinocyte proliferation | 13 | GO:0043616 | 0.376 |
| nucleotide phosphorylation | 12 | GO:0046939 | 0.376 |
| ammonium transport | 6 | GO:0015696 | 0.377 |
| phenol-containing compound metabolic process | 6 | GO:0018958 | 0.377 |
| detection of calcium ion | 9 | GO:0005513 | 0.378 |
| regulation of synaptic transmission, glutamatergic | 10 | GO:0051966 | 0.378 |
| gamma-aminobutyric acid signaling pathway | 25 | GO:0007214 | 0.379 |
| respiratory gaseous exchange | 34 | GO:0007585 | 0.379 |
| chromosome condensation | 18 | GO:0030261 | 0.381 |
| cell adhesion mediated by integrin | 5 | GO:0033627 | 0.381 |
| positive regulation of mitosis | 26 | GO:0045840 | 0.381 |
| growth plate cartilage development | 7 | GO:0003417 | 0.382 |
| telomere maintenance via recombination | 22 | GO:0000722 | 0.383 |
| inhibition of adenylate cyclase activity by metabotropic glutamate receptor signaling pathway | 7 | GO:0007196 | 0.383 |
| SCF-dependent proteasomal ubiquitin-dependent protein catabolic process | 11 | GO:0031146 | 0.383 |
| DNA methylation involved in gamete generation | 13 | GO:0043046 | 0.383 |
| triglyceride homeostasis | 16 | GO:0070328 | 0.383 |
| response to inorganic substance | 32 | GO:0010035 | 0.384 |
| reciprocal meiotic recombination | 27 | GO:0007131 | 0.385 |
| carboxylic acid metabolic process | 11 | GO:0019752 | 0.386 |
| negative regulation of mitosis | 7 | GO:0045839 | 0.386 |
| interspecies interaction between organisms | 310 | GO:0044419 | 0.387 |
| positive regulation of astrocyte differentiation | 7 | GO:0048711 | 0.387 |
| positive regulation of cell-cell adhesion | 14 | GO:0022409 | 0.388 |
| interleukin-6-mediated signaling pathway | 5 | GO:0070102 | 0.388 |
| mitotic chromosome condensation | 13 | GO:0007076 | 0.389 |
| phospholipid efflux | 11 | GO:0033700 | 0.389 |
| regulation of fibroblast growth factor receptor signaling pathway | 5 | GO:0040036 | 0.389 |
| glycine biosynthetic process | 5 | GO:0006545 | 0.39 |
| tubulin complex assembly | 6 | GO:0007021 | 0.39 |
| skeletal muscle tissue development | 60 | GO:0007519 | 0.39 |
| positive regulation of phosphatidylinositol 3-kinase activity | 12 | GO:0043552 | 0.39 |
| positive regulation of filopodium assembly | 10 | GO:0051491 | 0.39 |
| cellular glucose homeostasis | 8 | GO:0001678 | 0.392 |
| positive regulation of triglyceride catabolic process | 6 | GO:0010898 | 0.392 |
| positive regulation of natural killer cell mediated cytotoxicity | 14 | GO:0045954 | 0.392 |
| leukocyte homeostasis | 5 | GO:0001776 | 0.393 |
| mitosis | 224 | GO:0007067 | 0.393 |
| T cell lineage commitment | 5 | GO:0002360 | 0.394 |
| mitochondrial electron transport, ubiquinol to cytochrome c | 5 | GO:0006122 | 0.394 |
| positive regulation of epithelial cell differentiation | 8 | GO:0030858 | 0.394 |
| acute inflammatory response to antigenic stimulus | 5 | GO:0002438 | 0.395 |
| apical protein localization | 11 | GO:0045176 | 0.395 |
| locomotion | 7 | GO:0040011 | 0.396 |
| regulation of odontogenesis of dentine-containing tooth | 7 | GO:0042487 | 0.396 |
| L-serine metabolic process | 6 | GO:0006563 | 0.397 |
| membrane to membrane docking | 5 | GO:0022614 | 0.397 |
| positive regulation of protein export from nucleus | 7 | GO:0046827 | 0.398 |
| cholesterol metabolic process | 67 | GO:0008203 | 0.399 |
| activation of caspase activity by cytochrome c | 11 | GO:0008635 | 0.399 |
| positive regulation of viral genome replication | 10 | GO:0045070 | 0.399 |
| cellular response to peptide hormone stimulus | 17 | GO:0071375 | 0.399 |
| response to insecticide | 12 | GO:0017085 | 0.4 |
| negative regulation of appetite | 6 | GO:0032099 | 0.4 |
| positive regulation of Rac GTPase activity | 15 | GO:0032855 | 0.4 |
| negative regulation of gene-specific transcription | 40 | GO:0032582 | 0.401 |
| regulation of androgen receptor signaling pathway | 8 | GO:0060765 | 0.401 |
| Schwann cell differentiation | 6 | GO:0014037 | 0.402 |
| cellular response to reactive oxygen species | 9 | GO:0034614 | 0.402 |
| calcium ion homeostasis | 13 | GO:0055074 | 0.402 |
| myotube differentiation | 10 | GO:0014902 | 0.403 |
| triglyceride biosynthetic process | 32 | GO:0019432 | 0.403 |
| cellular response to hypoxia | 37 | GO:0071456 | 0.403 |
| positive regulation of synaptic transmission, GABAergic | 8 | GO:0032230 | 0.404 |
| positive regulation of mammary gland epithelial cell proliferation | 5 | GO:0033601 | 0.404 |
| behavioral response to nicotine | 8 | GO:0035095 | 0.404 |
| regulation of cell-matrix adhesion | 8 | GO:0001952 | 0.405 |
| negative regulation of smooth muscle cell differentiation | 5 | GO:0051151 | 0.405 |
| lipoprotein metabolic process | 37 | GO:0042157 | 0.406 |
| anti-apoptosis | 209 | GO:0006916 | 0.407 |
| organ growth | 14 | GO:0035265 | 0.407 |
| positive regulation of epidermal growth factor receptor activity | 7 | GO:0045741 | 0.407 |
| pancreas development | 32 | GO:0031016 | 0.408 |
| response to corticosteroid stimulus | 11 | GO:0031960 | 0.409 |
| isocitrate metabolic process | 5 | GO:0006102 | 0.41 |
| membrane protein intracellular domain proteolysis | 11 | GO:0031293 | 0.41 |
| negative regulation of telomere maintenance | 5 | GO:0032205 | 0.41 |
| monocarboxylic acid transport | 8 | GO:0015718 | 0.412 |
| cholesterol efflux | 21 | GO:0033344 | 0.412 |
| lipid biosynthetic process | 92 | GO:0008610 | 0.413 |
| negative regulation of kinase activity | 6 | GO:0033673 | 0.413 |
| neural crest cell fate commitment | 5 | GO:0014034 | 0.414 |
| myeloid cell differentiation | 20 | GO:0030099 | 0.414 |
| negative regulation of smooth muscle cell proliferation | 26 | GO:0048662 | 0.414 |
| protein thiol-disulfide exchange | 6 | GO:0006467 | 0.415 |
| V(D)J recombination | 9 | GO:0033151 | 0.415 |
| regulation of mRNA stability | 12 | GO:0043488 | 0.415 |
| inner ear receptor cell differentiation | 9 | GO:0060113 | 0.416 |
| mitochondrial transport | 15 | GO:0006839 | 0.417 |
| positive regulation of protein ubiquitination | 35 | GO:0031398 | 0.417 |
| intermediate filament-based process | 7 | GO:0045103 | 0.417 |
| male genitalia morphogenesis | 5 | GO:0048808 | 0.417 |
| G-protein signaling, coupled to cGMP nucleotide second messenger | 13 | GO:0007199 | 0.418 |
| single fertilization | 34 | GO:0007338 | 0.418 |
| neural crest cell development | 10 | GO:0014032 | 0.418 |
| blood vessel endothelial cell migration | 8 | GO:0043534 | 0.418 |
| retinol metabolic process | 15 | GO:0042572 | 0.419 |
| placenta development | 27 | GO:0001890 | 0.42 |
| positive regulation of Rab GTPase activity | 5 | GO:0032851 | 0.42 |
| endocardial cushion development | 5 | GO:0003197 | 0.421 |
| smooth muscle tissue development | 14 | GO:0048745 | 0.421 |
| embryonic process involved in female pregnancy | 8 | GO:0060136 | 0.421 |
| mammary gland alveolus development | 17 | GO:0060749 | 0.421 |
| inactivation of MAPK activity | 24 | GO:0000188 | 0.422 |
| blood coagulation, intrinsic pathway | 18 | GO:0007597 | 0.422 |
| B cell apoptosis | 6 | GO:0001783 | 0.423 |
| dentate gyrus development | 11 | GO:0021542 | 0.423 |
| positive regulation of BMP signaling pathway | 15 | GO:0030513 | 0.423 |
| melanin biosynthetic process | 11 | GO:0042438 | 0.423 |
| positive regulation of DNA damage response, signal transduction by p53 class mediator | 5 | GO:0043517 | 0.423 |
| oxidative phosphorylation | 8 | GO:0006119 | 0.424 |
| protein localization | 69 | GO:0008104 | 0.424 |
| positive regulation of fibroblast migration | 5 | GO:0010763 | 0.424 |
| positive regulation of oxidoreductase activity | 7 | GO:0051353 | 0.424 |
| histone mRNA metabolic process | 12 | GO:0008334 | 0.425 |
| positive regulation of TOR signaling cascade | 10 | GO:0032008 | 0.425 |
| negative regulation of angiogenesis | 47 | GO:0016525 | 0.426 |
| negative regulation of blood coagulation | 10 | GO:0030195 | 0.426 |
| adherens junction assembly | 5 | GO:0034333 | 0.426 |
| alternative nuclear mRNA splicing, via spliceosome | 6 | GO:0000380 | 0.427 |
| translation | 299 | GO:0006412 | 0.428 |
| nucleotide biosynthetic process | 17 | GO:0009165 | 0.428 |
| negative regulation of receptor-mediated endocytosis | 6 | GO:0048261 | 0.428 |
| synaptic transmission, GABAergic | 6 | GO:0051932 | 0.428 |
| negative regulation of survival gene product expression | 9 | GO:0008634 | 0.429 |
| activation of phospholipase C activity by dopamine receptor signaling pathway | 8 | GO:0060158 | 0.43 |
| cellular process | 32 | GO:0009987 | 0.431 |
| positive regulation of glucose metabolic process | 6 | GO:0010907 | 0.431 |
| regulation of BMP signaling pathway | 6 | GO:0030510 | 0.431 |
| positive regulation of translational initiation | 7 | GO:0045948 | 0.431 |
| chromatin organization | 21 | GO:0006325 | 0.432 |
| serotonin receptor signaling pathway | 7 | GO:0007210 | 0.432 |
| granulocyte differentiation | 5 | GO:0030851 | 0.432 |
| ventricular cardiac muscle cell development | 7 | GO:0055015 | 0.432 |
| cochlea development | 8 | GO:0090102 | 0.432 |
| induction of apoptosis by oxidative stress | 8 | GO:0008631 | 0.435 |
| negative regulation of anti-apoptosis | 6 | GO:0019987 | 0.435 |
| adrenal gland development | 26 | GO:0030325 | 0.435 |
| ribosomal small subunit biogenesis | 13 | GO:0042274 | 0.435 |
| eye photoreceptor cell development | 19 | GO:0042462 | 0.435 |
| positive regulation of DNA metabolic process | 7 | GO:0051054 | 0.435 |
| negative regulation of heart contraction | 9 | GO:0045822 | 0.436 |
| nucleoside transport | 6 | GO:0015858 | 0.437 |
| central nervous system projection neuron axonogenesis | 8 | GO:0021952 | 0.437 |
| dopamine biosynthetic process | 8 | GO:0042416 | 0.437 |
| odontogenesis | 32 | GO:0042476 | 0.437 |
| regulation of cell-cell adhesion | 7 | GO:0022407 | 0.438 |
| negative regulation of smooth muscle cell apoptosis | 5 | GO:0034392 | 0.438 |
| white fat cell differentiation | 11 | GO:0050872 | 0.438 |
| diaphragm development | 5 | GO:0060539 | 0.438 |
| cellular response to dsRNA | 6 | GO:0071359 | 0.438 |
| DNA-dependent DNA replication initiation | 19 | GO:0006270 | 0.439 |
| phosphate metabolic process | 22 | GO:0006796 | 0.439 |
| positive regulation of penile erection | 7 | GO:0060406 | 0.439 |
| negative regulation of epithelial cell proliferation involved in prostate gland development | 7 | GO:0060770 | 0.439 |
| positive regulation of cartilage development | 7 | GO:0061036 | 0.439 |
| mitotic sister chromatid segregation | 14 | GO:0000070 | 0.44 |
| brain segmentation | 5 | GO:0035284 | 0.44 |
| memory | 48 | GO:0007613 | 0.441 |
| response to metal ion | 23 | GO:0010038 | 0.441 |
| fucose metabolic process | 7 | GO:0006004 | 0.442 |
| short-term memory | 5 | GO:0007614 | 0.442 |
| apoptotic mitochondrial changes | 17 | GO:0008637 | 0.442 |
| collagen fibril organization | 32 | GO:0030199 | 0.442 |
| regulation of cyclin-dependent protein kinase activity | 55 | GO:0000079 | 0.443 |
| NAD biosynthetic process | 9 | GO:0009435 | 0.444 |
| response to lead ion | 23 | GO:0010288 | 0.444 |
| negative regulation of cAMP biosynthetic process | 11 | GO:0030818 | 0.445 |
| retinoic acid metabolic process | 12 | GO:0042573 | 0.445 |
| growth hormone secretion | 8 | GO:0030252 | 0.446 |
| regulation of circadian sleep/wake cycle, sleep | 5 | GO:0045187 | 0.446 |
| inner cell mass cell proliferation | 11 | GO:0001833 | 0.447 |
| GPI anchor biosynthetic process | 32 | GO:0006506 | 0.447 |
| receptor-mediated endocytosis | 54 | GO:0006898 | 0.447 |
| poly(A)+ mRNA export from nucleus | 6 | GO:0016973 | 0.447 |
| optic nerve morphogenesis | 5 | GO:0021631 | 0.447 |
| chromosome organization | 20 | GO:0051276 | 0.447 |
| DNA synthesis involved in DNA repair | 9 | GO:0000731 | 0.448 |
| transcription elongation from RNA polymerase I promoter | 19 | GO:0006362 | 0.448 |
| activation-induced cell death of T cells | 7 | GO:0006924 | 0.448 |
| regulation of protein kinase A signaling cascade | 6 | GO:0010738 | 0.448 |
| hormone metabolic process | 15 | GO:0042445 | 0.449 |
| cellular response to manganese ion | 5 | GO:0071287 | 0.449 |
| regulation of DNA repair | 5 | GO:0006282 | 0.45 |
| histidine metabolic process | 5 | GO:0006547 | 0.45 |
| vesicle targeting | 11 | GO:0006903 | 0.45 |
| xenobiotic catabolic process | 5 | GO:0042178 | 0.45 |
| response to fatty acid | 23 | GO:0070542 | 0.45 |
| nuclear export | 6 | GO:0051168 | 0.451 |
| telomere maintenance | 49 | GO:0000723 | 0.452 |
| positive regulation of epithelial cell proliferation | 53 | GO:0050679 | 0.452 |
| negative regulation of protein secretion | 10 | GO:0050709 | 0.452 |
| cellular response to cell-matrix adhesion | 5 | GO:0071460 | 0.453 |
| negative regulation of gene expression | 50 | GO:0010629 | 0.454 |
| regulation of metabolic process | 6 | GO:0019222 | 0.454 |
| regulation of steroid biosynthetic process | 8 | GO:0050810 | 0.454 |
| cell redox homeostasis | 65 | GO:0045454 | 0.455 |
| glucose catabolic process | 5 | GO:0006007 | 0.456 |
| SRP-dependent cotranslational protein targeting to membrane | 6 | GO:0006614 | 0.456 |
| flavonoid metabolic process | 5 | GO:0009812 | 0.456 |
| positive regulation of catalytic activity | 82 | GO:0043085 | 0.456 |
| positive regulation of osteoblast differentiation | 37 | GO:0045669 | 0.456 |
| positive regulation of heart contraction | 5 | GO:0045823 | 0.456 |
| oligodendrocyte differentiation | 18 | GO:0048709 | 0.456 |
| DNA topological change | 9 | GO:0006265 | 0.457 |
| induction of apoptosis by intracellular signals | 45 | GO:0008629 | 0.457 |
| positive regulation of keratinocyte migration | 5 | GO:0051549 | 0.457 |
| folic acid-containing compound biosynthetic process | 7 | GO:0009396 | 0.458 |
| phosphatidylcholine metabolic process | 10 | GO:0046470 | 0.458 |
| positive regulation of T cell differentiation in thymus | 8 | GO:0033089 | 0.459 |
| rhythmic process | 29 | GO:0048511 | 0.46 |
| regulation of gene expression | 74 | GO:0010468 | 0.461 |
| neuronal ion channel clustering | 5 | GO:0045161 | 0.461 |
| mitotic cell cycle | 292 | GO:0000278 | 0.463 |
| synapsis | 7 | GO:0007129 | 0.463 |
| body fluid secretion | 12 | GO:0007589 | 0.463 |
| sensory perception of pain | 56 | GO:0019233 | 0.463 |
| cell fate commitment | 43 | GO:0045165 | 0.463 |
| protein homotetramerization | 46 | GO:0051289 | 0.463 |
| NADP metabolic process | 8 | GO:0006739 | 0.464 |
| ketone body biosynthetic process | 5 | GO:0046951 | 0.464 |
| N-glycan processing | 10 | GO:0006491 | 0.465 |
| tube morphogenesis | 6 | GO:0035239 | 0.465 |
| regulation of cytokine biosynthetic process | 9 | GO:0042035 | 0.466 |
| ear development | 8 | GO:0043583 | 0.466 |
| positive regulation of B cell activation | 6 | GO:0050871 | 0.466 |
| negative regulation of lipoprotein lipase activity | 5 | GO:0051005 | 0.466 |
| response to cobalt ion | 9 | GO:0032025 | 0.467 |
| protein insertion into membrane | 6 | GO:0051205 | 0.467 |
| embryonic digestive tract morphogenesis | 16 | GO:0048557 | 0.468 |
| DNA recombination | 67 | GO:0006310 | 0.469 |
| lysosomal lumen acidification | 5 | GO:0007042 | 0.469 |
| positive regulation of nitric-oxide synthase activity | 10 | GO:0051000 | 0.469 |
| muscle cell development | 5 | GO:0055001 | 0.469 |
| homeostasis of number of cells | 10 | GO:0048872 | 0.47 |
| hepatocyte differentiation | 9 | GO:0070365 | 0.47 |
| cellular response to dexamethasone stimulus | 18 | GO:0071549 | 0.47 |
| vacuolar acidification | 6 | GO:0007035 | 0.471 |
| negative regulation of DNA endoreduplication | 5 | GO:0032876 | 0.471 |
| cardiac muscle contraction | 26 | GO:0060048 | 0.471 |
| behavior | 39 | GO:0007610 | 0.473 |
| neurotransmitter biosynthetic process | 14 | GO:0042136 | 0.473 |
| oogenesis | 21 | GO:0048477 | 0.473 |
| fructose 2,6-bisphosphate metabolic process | 5 | GO:0006003 | 0.474 |
| embryonic body morphogenesis | 6 | GO:0010172 | 0.474 |
| neuron remodeling | 7 | GO:0016322 | 0.474 |
| positive regulation of synaptic transmission, cholinergic | 6 | GO:0032224 | 0.474 |
| regulation of chondrocyte differentiation | 5 | GO:0032330 | 0.474 |
| glial cell apoptosis | 7 | GO:0034349 | 0.474 |
| norepinephrine metabolic process | 6 | GO:0042415 | 0.474 |
| preassembly of GPI anchor in ER membrane | 16 | GO:0016254 | 0.475 |
| proteoglycan biosynthetic process | 11 | GO:0030166 | 0.475 |
| alcohol metabolic process | 6 | GO:0006066 | 0.477 |
| positive regulation of natural killer cell mediated cytotoxicity directed against tumor cell target | 6 | GO:0002860 | 0.478 |
| interleukin-1-mediated signaling pathway | 12 | GO:0070498 | 0.478 |
| axon regeneration | 14 | GO:0031103 | 0.479 |
| membranous septum morphogenesis | 5 | GO:0003149 | 0.48 |
| response to mechanical stimulus | 77 | GO:0009612 | 0.481 |
| axis specification | 14 | GO:0009798 | 0.482 |
| negative regulation of secretion | 5 | GO:0051048 | 0.483 |
| negative regulation of cytokine production | 5 | GO:0001818 | 0.484 |
| ATP catabolic process | 124 | GO:0006200 | 0.485 |
| iron-sulfur cluster assembly | 10 | GO:0016226 | 0.485 |
| positive regulation of fibroblast proliferation | 40 | GO:0048146 | 0.485 |
| kidney morphogenesis | 5 | GO:0060993 | 0.485 |
| protein metabolic process | 15 | GO:0019538 | 0.486 |
| sexual reproduction | 13 | GO:0019953 | 0.487 |
| intracellular cholesterol transport | 5 | GO:0032367 | 0.487 |
| negative regulation of adenylate cyclase activity | 21 | GO:0007194 | 0.488 |
| drug export | 7 | GO:0046618 | 0.488 |
| exocrine pancreas development | 8 | GO:0031017 | 0.489 |
| regulation of respiratory gaseous exchange by neurological system process | 12 | GO:0002087 | 0.49 |
| positive regulation of fibroblast growth factor receptor signaling pathway | 6 | GO:0045743 | 0.49 |
| mitotic prometaphase | 84 | GO:0000236 | 0.491 |
| G2 phase of mitotic cell cycle | 10 | GO:0000085 | 0.493 |
| regulation of protein metabolic process | 7 | GO:0051246 | 0.494 |
| apoptotic nuclear change | 5 | GO:0030262 | 0.495 |
| adipose tissue development | 16 | GO:0060612 | 0.495 |
| cAMP catabolic process | 12 | GO:0006198 | 0.496 |
| positive regulation of glutamate secretion | 8 | GO:0014049 | 0.497 |
| potassium ion transmembrane transport | 61 | GO:0071805 | 0.499 |
| gastrulation | 26 | GO:0007369 | 0.503 |
| cellular ion homeostasis | 6 | GO:0006873 | 0.504 |
| adenohypophysis development | 9 | GO:0021984 | 0.505 |
| high-density lipoprotein particle clearance | 6 | GO:0034384 | 0.507 |
| M phase of mitotic cell cycle | 95 | GO:0000087 | 0.508 |
| negative regulation of blood vessel endothelial cell migration | 10 | GO:0043537 | 0.513 |
|  |  |  |  |
| **Molecular Function** |  |  |  |
| neuropeptide hormone activity | 27 | GO:0005184 | <0.002 |
| hyaluronic acid binding | 21 | GO:0005540 | <0.002 |
| oxidoreductase activity, acting on paired donors, with incorporation or reduction of molecular oxygen, reduced flavin or flavoprotein as one donor, and incorporation of one atom of oxygen | 10 | GO:0016712 | <0.002 |
| aromatase activity | 21 | GO:0070330 | 0.003 |
| monooxygenase activity | 73 | GO:0004497 | 0.005 |
| pancreatic ribonuclease activity | 8 | GO:0004522 | 0.006 |
| organic anion transmembrane transporter activity | 15 | GO:0008514 | 0.006 |
| sodium:phosphate symporter activity | 8 | GO:0005436 | 0.007 |
| water channel activity | 10 | GO:0015250 | 0.007 |
| oxygen binding | 39 | GO:0019825 | 0.008 |
| protein self-association | 33 | GO:0043621 | 0.008 |
| peptidoglycan receptor activity | 5 | GO:0016019 | 0.009 |
| peptide hormone receptor binding | 10 | GO:0051428 | 0.009 |
| fibronectin binding | 24 | GO:0001968 | 0.01 |
| sodium-dependent phosphate transmembrane transporter activity | 7 | GO:0015321 | 0.011 |
| MAP kinase tyrosine/serine/threonine phosphatase activity | 13 | GO:0017017 | 0.011 |
| hormone activity | 88 | GO:0005179 | 0.012 |
| iron ion binding | 112 | GO:0005506 | 0.012 |
| hydrolase activity, acting on carbon-nitrogen (but not peptide) bonds, in linear amidines | 5 | GO:0016813 | 0.012 |
| nitric-oxide synthase binding | 9 | GO:0050998 | 0.013 |
| transporter activity | 265 | GO:0005215 | 0.014 |
| protein homodimerization activity | 510 | GO:0042803 | 0.015 |
| troponin T binding | 5 | GO:0031014 | 0.016 |
| endopeptidase activator activity | 7 | GO:0061133 | 0.017 |
| purinergic nucleotide receptor activity | 7 | GO:0001614 | 0.018 |
| extracellular ATP-gated cation channel activity | 7 | GO:0004931 | 0.018 |
| IgG binding | 9 | GO:0019864 | 0.02 |
| heme binding | 123 | GO:0020037 | 0.02 |
| electron carrier activity | 169 | GO:0009055 | 0.021 |
| zinc ion transmembrane transporter activity | 17 | GO:0005385 | 0.022 |
| inorganic anion exchanger activity | 16 | GO:0005452 | 0.022 |
| peptidase inhibitor activity | 95 | GO:0030414 | 0.022 |
| dopamine receptor binding | 5 | GO:0050780 | 0.022 |
| endopeptidase inhibitor activity | 36 | GO:0004866 | 0.023 |
| steroid hydroxylase activity | 9 | GO:0008395 | 0.024 |
| galactoside binding | 6 | GO:0016936 | 0.024 |
| lipoxygenase activity | 6 | GO:0016165 | 0.027 |
| peptidoglycan binding | 8 | GO:0042834 | 0.028 |
| endodeoxyribonuclease activity | 9 | GO:0004520 | 0.029 |
| ligand-gated ion channel activity | 14 | GO:0015276 | 0.029 |
| somatostatin receptor activity | 5 | GO:0004994 | 0.03 |
| superoxide-generating NADPH oxidase activity | 10 | GO:0016175 | 0.03 |
| MHC class II receptor activity | 8 | GO:0032395 | 0.03 |
| lipopolysaccharide binding | 13 | GO:0001530 | 0.031 |
| lipopolysaccharide receptor activity | 5 | GO:0001875 | 0.032 |
| phosphorylase activity | 5 | GO:0004645 | 0.033 |
| anion:anion antiporter activity | 16 | GO:0015301 | 0.033 |
| opioid receptor activity | 9 | GO:0004985 | 0.035 |
| pattern recognition receptor activity | 5 | GO:0008329 | 0.035 |
| serine-type endopeptidase inhibitor activity | 81 | GO:0004867 | 0.036 |
| single-stranded RNA binding | 23 | GO:0003727 | 0.037 |
| phospholipase A2 activity | 19 | GO:0004623 | 0.037 |
| phosphatidylinositol-4,5-bisphosphate 3-kinase activity | 5 | GO:0046934 | 0.037 |
| chemokine activity | 43 | GO:0008009 | 0.038 |
| muscarinic acetylcholine receptor activity | 5 | GO:0004981 | 0.039 |
| N-formyl peptide receptor activity | 5 | GO:0004982 | 0.039 |
| receptor signaling protein activity | 41 | GO:0005057 | 0.04 |
| oxidoreductase activity, acting on paired donors, with incorporation or reduction of molecular oxygen | 54 | GO:0016705 | 0.04 |
| phosphatidylinositol phospholipase C activity | 28 | GO:0004435 | 0.041 |
| peptidase activity | 470 | GO:0008233 | 0.041 |
| symporter activity | 107 | GO:0015293 | 0.041 |
| G-protein-coupled receptor binding | 51 | GO:0001664 | 0.042 |
| coreceptor activity | 24 | GO:0015026 | 0.042 |
| cytokine activity | 169 | GO:0005125 | 0.045 |
| sodium-independent organic anion transmembrane transporter activity | 7 | GO:0015347 | 0.046 |
| C-X-C chemokine receptor activity | 6 | GO:0016494 | 0.046 |
| phosphotyrosine binding | 15 | GO:0001784 | 0.047 |
| RNA polymerase II core promoter proximal region sequence-specific DNA binding transcription factor activity involved in positive regulation of transcription | 16 | GO:0001077 | 0.048 |
| tumor necrosis factor receptor binding | 27 | GO:0005164 | 0.049 |
| non-membrane spanning protein tyrosine kinase activity | 38 | GO:0004715 | 0.05 |
| cysteine-type endopeptidase inhibitor activity | 21 | GO:0004869 | 0.051 |
| protein kinase C binding | 42 | GO:0005080 | 0.051 |
| amino acid binding | 29 | GO:0016597 | 0.051 |
| sodium ion binding | 8 | GO:0031402 | 0.051 |
| laminin binding | 17 | GO:0043236 | 0.052 |
| N-acetylgalactosamine 4-O-sulfotransferase activity | 5 | GO:0001537 | 0.054 |
| monovalent inorganic cation transmembrane transporter activity | 8 | GO:0015077 | 0.054 |
| activating transcription factor binding | 10 | GO:0033613 | 0.055 |
| cadmium ion binding | 5 | GO:0046870 | 0.055 |
| gamma-glutamyltransferase activity | 6 | GO:0003840 | 0.057 |
| protein-glutamine gamma-glutamyltransferase activity | 9 | GO:0003810 | 0.058 |
| chemokine receptor activity | 23 | GO:0004950 | 0.059 |
| serine-type peptidase activity | 121 | GO:0008236 | 0.06 |
| hydrolase activity, acting on carbon-nitrogen (but not peptide) bonds, in cyclic amidines | 10 | GO:0016814 | 0.06 |
| ribonuclease H activity | 5 | GO:0004523 | 0.061 |
| intracellular cyclic nucleotide activated cation channel activity | 6 | GO:0005221 | 0.062 |
| transmembrane receptor activity | 167 | GO:0004888 | 0.064 |
| nicotinic acetylcholine-activated cation-selective channel activity | 16 | GO:0004889 | 0.064 |
| cysteine-type endopeptidase activity | 66 | GO:0004197 | 0.065 |
| C-C chemokine binding | 5 | GO:0019957 | 0.065 |
| peptide binding | 66 | GO:0042277 | 0.065 |
| amino acid transmembrane transporter activity | 36 | GO:0015171 | 0.066 |
| structural constituent of epidermis | 9 | GO:0030280 | 0.067 |
| serine-type endopeptidase activity | 135 | GO:0004252 | 0.068 |
| nucleoside kinase activity | 7 | GO:0019206 | 0.068 |
| NAD(P)H oxidase activity | 6 | GO:0016174 | 0.07 |
| structural constituent of cytoskeleton | 91 | GO:0005200 | 0.072 |
| threonine-type endopeptidase activity | 20 | GO:0004298 | 0.073 |
| cytokine receptor activity | 33 | GO:0004896 | 0.073 |
| antigen binding | 27 | GO:0003823 | 0.075 |
| nucleoside binding | 7 | GO:0001882 | 0.077 |
| endopeptidase activity | 59 | GO:0004175 | 0.077 |
| interleukin-1 receptor activity | 8 | GO:0004908 | 0.081 |
| melanocortin receptor activity | 5 | GO:0004977 | 0.081 |
| serine-type carboxypeptidase activity | 5 | GO:0004185 | 0.082 |
| lipase activity | 9 | GO:0016298 | 0.082 |
| sodium:dicarboxylate symporter activity | 9 | GO:0017153 | 0.082 |
| IgE binding | 5 | GO:0019863 | 0.082 |
| lysozyme activity | 8 | GO:0003796 | 0.084 |
| voltage-gated calcium channel activity | 29 | GO:0005245 | 0.085 |
| ionotropic glutamate receptor activity | 18 | GO:0004970 | 0.086 |
| transmembrane receptor protein tyrosine kinase adaptor activity | 9 | GO:0005068 | 0.086 |
| extracellular ligand-gated ion channel activity | 44 | GO:0005230 | 0.086 |
| extracellular-glutamate-gated ion channel activity | 19 | GO:0005234 | 0.086 |
| G-protein coupled amine receptor activity | 6 | GO:0008227 | 0.087 |
| structure-specific DNA binding | 6 | GO:0043566 | 0.087 |
| specific RNA polymerase II transcription factor activity | 81 | GO:0003704 | 0.088 |
| calcium channel activity | 77 | GO:0005262 | 0.089 |
| porin activity | 14 | GO:0015288 | 0.094 |
| chemokine receptor binding | 5 | GO:0042379 | 0.094 |
| purinergic nucleotide receptor activity, G-protein coupled | 23 | GO:0045028 | 0.094 |
| calcium-dependent phospholipase A2 activity | 6 | GO:0047498 | 0.095 |
| aldehyde dehydrogenase [NAD(P)+] activity | 7 | GO:0004030 | 0.096 |
| gap junction channel activity | 13 | GO:0005243 | 0.097 |
| G-protein coupled photoreceptor activity | 7 | GO:0008020 | 0.097 |
| sugar binding | 168 | GO:0005529 | 0.098 |
| phosphotransferase activity, alcohol group as acceptor | 47 | GO:0016773 | 0.099 |
| CCR5 chemokine receptor binding | 5 | GO:0031730 | 0.099 |
| caspase activator activity | 20 | GO:0008656 | 0.1 |
| high voltage-gated calcium channel activity | 7 | GO:0008331 | 0.101 |
| ion channel activity | 313 | GO:0005216 | 0.102 |
| adenosine deaminase activity | 9 | GO:0004000 | 0.103 |
| L-amino acid transmembrane transporter activity | 6 | GO:0015179 | 0.105 |
| chitinase activity | 7 | GO:0004568 | 0.106 |
| phospholipase inhibitor activity | 10 | GO:0004859 | 0.106 |
| scavenger receptor activity | 45 | GO:0005044 | 0.107 |
| cytokine receptor binding | 14 | GO:0005126 | 0.108 |
| vitamin D binding | 8 | GO:0005499 | 0.108 |
| T cell receptor binding | 5 | GO:0042608 | 0.108 |
| protein dimerization activity | 105 | GO:0046983 | 0.108 |
| poly(U) RNA binding | 6 | GO:0008266 | 0.109 |
| chloride channel activity | 66 | GO:0005254 | 0.11 |
| sodium:potassium-exchanging ATPase activity | 11 | GO:0005391 | 0.11 |
| fatty acid binding | 23 | GO:0005504 | 0.116 |
| protein transmembrane transporter activity | 5 | GO:0008320 | 0.116 |
| receptor binding | 298 | GO:0005102 | 0.12 |
| beta-amyloid binding | 26 | GO:0001540 | 0.122 |
| voltage-gated anion channel activity | 6 | GO:0008308 | 0.123 |
| water transmembrane transporter activity | 6 | GO:0005372 | 0.124 |
| FMN binding | 14 | GO:0010181 | 0.125 |
| MHC class I receptor activity | 12 | GO:0032393 | 0.125 |
| RNA polymerase binding | 5 | GO:0070063 | 0.126 |
| polypeptide N-acetylgalactosaminyltransferase activity | 18 | GO:0004653 | 0.128 |
| neuropeptide receptor activity | 13 | GO:0008188 | 0.128 |
| cAMP-dependent protein kinase activity | 7 | GO:0004691 | 0.129 |
| G-protein beta/gamma-subunit complex binding | 23 | GO:0031683 | 0.129 |
| protease binding | 50 | GO:0002020 | 0.13 |
| oxygen transporter activity | 10 | GO:0005344 | 0.13 |
| calcium sensitive guanylate cyclase activator activity | 5 | GO:0008048 | 0.13 |
| cobalt ion binding | 5 | GO:0050897 | 0.13 |
| glycine binding | 13 | GO:0016594 | 0.133 |
| glycine transmembrane transporter activity | 5 | GO:0015187 | 0.135 |
| hexokinase activity | 6 | GO:0004396 | 0.136 |
| ion transmembrane transporter activity | 16 | GO:0015075 | 0.136 |
| extracellular-glycine-gated chloride channel activity | 5 | GO:0016934 | 0.136 |
| structural constituent of eye lens | 20 | GO:0005212 | 0.14 |
| S-adenosylmethionine-dependent methyltransferase activity | 17 | GO:0008757 | 0.14 |
| MHC class I protein binding | 12 | GO:0042288 | 0.14 |
| 2-acylglycerol O-acyltransferase activity | 5 | GO:0003846 | 0.141 |
| L-lactate dehydrogenase activity | 5 | GO:0004459 | 0.141 |
| deaminase activity | 5 | GO:0019239 | 0.141 |
| photoreceptor activity | 9 | GO:0009881 | 0.142 |
| small conjugating protein ligase activity | 7 | GO:0019787 | 0.142 |
| antiporter activity | 37 | GO:0015297 | 0.143 |
| phosphatidylcholine-sterol O-acyltransferase activator activity | 5 | GO:0060228 | 0.143 |
| cytokine binding | 17 | GO:0019955 | 0.145 |
| galactose binding | 6 | GO:0005534 | 0.146 |
| choline transmembrane transporter activity | 8 | GO:0015220 | 0.146 |
| death receptor binding | 14 | GO:0005123 | 0.147 |
| sequence-specific DNA binding | 506 | GO:0043565 | 0.147 |
| peptide antigen binding | 12 | GO:0042605 | 0.148 |
| caspase inhibitor activity | 20 | GO:0043027 | 0.148 |
| cation binding | 40 | GO:0043169 | 0.148 |
| sphingomyelin phosphodiesterase activity | 6 | GO:0004767 | 0.149 |
| hydrogen-exporting ATPase activity, phosphorylative mechanism | 14 | GO:0008553 | 0.15 |
| neutral amino acid transmembrane transporter activity | 12 | GO:0015175 | 0.15 |
| calcium activated cation channel activity | 5 | GO:0005227 | 0.152 |
| cation channel activity | 29 | GO:0005261 | 0.153 |
| omega peptidase activity | 7 | GO:0008242 | 0.153 |
| histone-arginine N-methyltransferase activity | 7 | GO:0008469 | 0.153 |
| ribonuclease activity | 18 | GO:0004540 | 0.154 |
| voltage-gated ion channel activity | 148 | GO:0005244 | 0.154 |
| potassium ion binding | 9 | GO:0030955 | 0.161 |
| tumor necrosis factor receptor activity | 12 | GO:0005031 | 0.162 |
| AT DNA binding | 5 | GO:0003680 | 0.164 |
| purine nucleotide binding | 8 | GO:0017076 | 0.164 |
| sphingosine-1-phosphate phosphatase activity | 5 | GO:0042392 | 0.164 |
| 3-chloroallyl aldehyde dehydrogenase activity | 8 | GO:0004028 | 0.167 |
| ankyrin binding | 9 | GO:0030506 | 0.169 |
| C-C chemokine receptor activity | 14 | GO:0016493 | 0.173 |
| chemoattractant activity | 16 | GO:0042056 | 0.173 |
| neuropeptide binding | 8 | GO:0042923 | 0.177 |
| glutathione peroxidase activity | 15 | GO:0004602 | 0.178 |
| glucose binding | 12 | GO:0005536 | 0.178 |
| SH2 domain binding | 33 | GO:0042169 | 0.178 |
| lysosphingolipid and lysophosphatidic acid receptor activity | 11 | GO:0001619 | 0.179 |
| natural killer cell lectin-like receptor binding | 5 | GO:0046703 | 0.179 |
| Gram-positive bacterial cell surface binding | 5 | GO:0051637 | 0.179 |
| peroxiredoxin activity | 7 | GO:0051920 | 0.179 |
| lipase inhibitor activity | 5 | GO:0055102 | 0.179 |
| interleukin-6 receptor binding | 5 | GO:0005138 | 0.181 |
| nuclear localization sequence binding | 9 | GO:0008139 | 0.181 |
| beta-N-acetylhexosaminidase activity | 5 | GO:0004563 | 0.182 |
| heparin binding | 124 | GO:0008201 | 0.183 |
| metal ion transmembrane transporter activity | 16 | GO:0046873 | 0.183 |
| iron ion transmembrane transporter activity | 5 | GO:0005381 | 0.184 |
| folic acid binding | 14 | GO:0005542 | 0.184 |
| hydrogen ion transmembrane transporter activity | 26 | GO:0015078 | 0.186 |
| calcium- and calmodulin-responsive adenylate cyclase activity | 6 | GO:0008294 | 0.188 |
| carbonate dehydratase activity | 15 | GO:0004089 | 0.189 |
| L-serine transmembrane transporter activity | 7 | GO:0015194 | 0.19 |
| transcription regulator activity | 376 | GO:0030528 | 0.19 |
| L-glutamate transmembrane transporter activity | 11 | GO:0005313 | 0.191 |
| cGMP binding | 13 | GO:0030553 | 0.191 |
| acetylcholine binding | 14 | GO:0042166 | 0.192 |
| mRNA 5'-UTR binding | 6 | GO:0048027 | 0.192 |
| hydrolase activity, acting on acid anhydrides, catalyzing transmembrane movement of substances | 47 | GO:0016820 | 0.194 |
| serotonin receptor activity | 14 | GO:0004993 | 0.195 |
| phosphoglucomutase activity | 7 | GO:0004614 | 0.196 |
| transmembrane receptor protein tyrosine phosphatase activity | 17 | GO:0005001 | 0.196 |
| [heparan sulfate]-glucosamine 3-sulfotransferase 1 activity | 7 | GO:0008467 | 0.196 |
| spectrin binding | 8 | GO:0030507 | 0.198 |
| ATP-dependent protein binding | 7 | GO:0043008 | 0.198 |
| aminopeptidase activity | 29 | GO:0004177 | 0.199 |
| cation:chloride symporter activity | 8 | GO:0015377 | 0.2 |
| steroid binding | 32 | GO:0005496 | 0.201 |
| CARD domain binding | 8 | GO:0050700 | 0.202 |
| growth factor activity | 157 | GO:0008083 | 0.203 |
| intramolecular transferase activity, phosphotransferases | 7 | GO:0016868 | 0.203 |
| NF-kappaB-inducing kinase activity | 6 | GO:0004704 | 0.204 |
| kinase binding | 42 | GO:0019900 | 0.206 |
| peptide YY receptor activity | 5 | GO:0001601 | 0.207 |
| potassium channel activity | 79 | GO:0005267 | 0.207 |
| calcium, potassium:sodium antiporter activity | 5 | GO:0008273 | 0.207 |
| NF-kappaB binding | 24 | GO:0051059 | 0.208 |
| nucleotide-sugar transmembrane transporter activity | 6 | GO:0005338 | 0.21 |
| N-methyl-D-aspartate selective glutamate receptor activity | 6 | GO:0004972 | 0.211 |
| kainate selective glutamate receptor activity | 7 | GO:0015277 | 0.211 |
| cyclin-dependent protein kinase regulator activity | 9 | GO:0016538 | 0.213 |
| protein tyrosine phosphatase activity | 97 | GO:0004725 | 0.215 |
| phosphoglycolate phosphatase activity | 5 | GO:0008967 | 0.215 |
| type 2 fibroblast growth factor receptor binding | 5 | GO:0005111 | 0.216 |
| double-stranded RNA binding | 34 | GO:0003725 | 0.217 |
| disulfide oxidoreductase activity | 6 | GO:0015036 | 0.217 |
| amine transmembrane transporter activity | 5 | GO:0005275 | 0.218 |
| long-chain fatty acid-CoA ligase activity | 13 | GO:0004467 | 0.219 |
| type 1 fibroblast growth factor receptor binding | 5 | GO:0005105 | 0.221 |
| transmembrane transporter activity | 45 | GO:0022857 | 0.221 |
| D-glucose transmembrane transporter activity | 8 | GO:0055056 | 0.226 |
| interleukin-1 receptor binding | 8 | GO:0005149 | 0.227 |
| phosphodiesterase I activity | 5 | GO:0004528 | 0.228 |
| drug binding | 81 | GO:0008144 | 0.228 |
| ATPase activity, coupled to transmembrane movement of ions, phosphorylative mechanism | 35 | GO:0015662 | 0.228 |
| voltage-gated sodium channel activity | 16 | GO:0005248 | 0.231 |
| non-membrane spanning protein tyrosine phosphatase activity | 8 | GO:0004726 | 0.232 |
| inward rectifier potassium channel activity | 21 | GO:0005242 | 0.232 |
| phosphatidylcholine binding | 8 | GO:0031210 | 0.234 |
| transition metal ion binding | 6 | GO:0046914 | 0.234 |
| phospholipase activity | 10 | GO:0004620 | 0.235 |
| uridine kinase activity | 5 | GO:0004849 | 0.236 |
| protein disulfide isomerase activity | 9 | GO:0003756 | 0.237 |
| peroxidase activity | 30 | GO:0004601 | 0.237 |
| glutamate receptor activity | 14 | GO:0008066 | 0.238 |
| isomerase activity | 114 | GO:0016853 | 0.239 |
| androgen receptor binding | 36 | GO:0050681 | 0.24 |
| lipid transporter activity | 19 | GO:0005319 | 0.241 |
| tetracycline:hydrogen antiporter activity | 5 | GO:0015520 | 0.241 |
| hydrogen ion transporting ATP synthase activity, rotational mechanism | 17 | GO:0046933 | 0.241 |
| alditol:NADP+ 1-oxidoreductase activity | 5 | GO:0004032 | 0.242 |
| chromatin DNA binding | 15 | GO:0031490 | 0.244 |
| glucocorticoid receptor binding | 12 | GO:0035259 | 0.244 |
| WW domain binding | 19 | GO:0050699 | 0.244 |
| monocarboxylic acid transmembrane transporter activity | 9 | GO:0008028 | 0.245 |
| channel activity | 13 | GO:0015267 | 0.246 |
| oxidoreductase activity | 521 | GO:0016491 | 0.247 |
| chloride channel regulator activity | 5 | GO:0017081 | 0.248 |
| GABA-A receptor activity | 19 | GO:0004890 | 0.249 |
| neurotransmitter:sodium symporter activity | 19 | GO:0005328 | 0.25 |
| identical protein binding | 399 | GO:0042802 | 0.251 |
| calcium-dependent phospholipid binding | 25 | GO:0005544 | 0.252 |
| acetylcholine receptor activity | 14 | GO:0015464 | 0.252 |
| DNA-(apurinic or apyrimidinic site) lyase activity | 11 | GO:0003906 | 0.253 |
| sodium channel activity | 27 | GO:0005272 | 0.253 |
| estradiol 17-beta-dehydrogenase activity | 11 | GO:0004303 | 0.254 |
| alcohol dehydrogenase (NAD) activity | 7 | GO:0004022 | 0.255 |
| UDP-galactose:beta-N-acetylglucosamine beta-1,3-galactosyltransferase activity | 8 | GO:0008499 | 0.256 |
| siRNA binding | 6 | GO:0035197 | 0.258 |
| general transcriptional repressor activity | 9 | GO:0016565 | 0.259 |
| MDM2 binding | 5 | GO:0070215 | 0.259 |
| thrombin receptor activity | 5 | GO:0015057 | 0.263 |
| apoptotic protease activator activity | 5 | GO:0016505 | 0.263 |
| extracellular matrix structural constituent conferring tensile strength | 6 | GO:0030020 | 0.263 |
| profilin binding | 9 | GO:0005522 | 0.265 |
| actin monomer binding | 9 | GO:0003785 | 0.266 |
| RNA cap binding | 7 | GO:0000339 | 0.269 |
| cholesterol binding | 26 | GO:0015485 | 0.269 |
| hemoglobin binding | 5 | GO:0030492 | 0.269 |
| translation regulator activity | 9 | GO:0045182 | 0.269 |
| intracellular calcium activated chloride channel activity | 5 | GO:0005229 | 0.27 |
| magnesium ion binding | 183 | GO:0000287 | 0.272 |
| lysophospholipase activity | 13 | GO:0004622 | 0.273 |
| copper ion transmembrane transporter activity | 6 | GO:0005375 | 0.273 |
| oxidoreductase activity, acting on the CH-OH group of donors, NAD or NADP as acceptor | 26 | GO:0016616 | 0.273 |
| transferase activity, transferring hexosyl groups | 36 | GO:0016758 | 0.274 |
| phospholipase D activity | 7 | GO:0004630 | 0.275 |
| hydrolase activity, acting on glycosyl bonds | 72 | GO:0016798 | 0.275 |
| peptide hormone binding | 31 | GO:0017046 | 0.275 |
| catalytic activity | 709 | GO:0003824 | 0.277 |
| testosterone 17-beta-dehydrogenase (NAD+) activity | 5 | GO:0050327 | 0.28 |
| bacterial cell surface binding | 11 | GO:0051635 | 0.28 |
| proton-transporting ATPase activity, rotational mechanism | 19 | GO:0046961 | 0.283 |
| proteasome binding | 6 | GO:0070628 | 0.285 |
| nucleotide diphosphatase activity | 6 | GO:0004551 | 0.286 |
| metalloexopeptidase activity | 13 | GO:0008235 | 0.287 |
| mRNA 3'-UTR binding | 26 | GO:0003730 | 0.289 |
| ion channel inhibitor activity | 7 | GO:0008200 | 0.289 |
| Ran GTPase binding | 15 | GO:0008536 | 0.289 |
| dipeptidyl-peptidase activity | 11 | GO:0008239 | 0.292 |
| methyl-CpG binding | 8 | GO:0008327 | 0.292 |
| citrate transmembrane transporter activity | 5 | GO:0015137 | 0.293 |
| interleukin-1 binding | 5 | GO:0019966 | 0.294 |
| dopamine binding | 10 | GO:0035240 | 0.296 |
| selenium binding | 13 | GO:0008430 | 0.298 |
| glutathione transferase activity | 17 | GO:0004364 | 0.3 |
| calcium-dependent protein serine/threonine phosphatase activity | 5 | GO:0004723 | 0.3 |
| glucose transmembrane transporter activity | 12 | GO:0005355 | 0.303 |
| microtubule motor activity | 75 | GO:0003777 | 0.304 |
| prostaglandin receptor activity | 5 | GO:0004955 | 0.304 |
| GPI anchor binding | 6 | GO:0034235 | 0.304 |
| transferase activity, transferring pentosyl groups | 9 | GO:0016763 | 0.307 |
| choline binding | 6 | GO:0033265 | 0.307 |
| protein-arginine omega-N asymmetric methyltransferase activity | 5 | GO:0035242 | 0.307 |
| eukaryotic cell surface binding | 22 | GO:0043499 | 0.308 |
| taste receptor activity | 12 | GO:0008527 | 0.309 |
| low-density lipoprotein receptor activity | 12 | GO:0005041 | 0.311 |
| complement binding | 7 | GO:0001848 | 0.312 |
| ribonucleoprotein binding | 16 | GO:0043021 | 0.313 |
| phosphatidylinositol 3-kinase regulator activity | 6 | GO:0035014 | 0.314 |
| acetate-CoA ligase activity | 5 | GO:0003987 | 0.315 |
| RNA polymerase II core promoter proximal region sequence-specific DNA binding | 7 | GO:0000978 | 0.319 |
| insulin-like growth factor receptor binding | 14 | GO:0005159 | 0.319 |
| ferric iron binding | 10 | GO:0008199 | 0.319 |
| beta-tubulin binding | 22 | GO:0048487 | 0.319 |
| R-SMAD binding | 12 | GO:0070412 | 0.32 |
| vasopressin receptor activity | 5 | GO:0005000 | 0.321 |
| retinol dehydrogenase activity | 12 | GO:0004745 | 0.322 |
| deoxyribonuclease activity | 8 | GO:0004536 | 0.323 |
| quaternary ammonium group transmembrane transporter activity | 6 | GO:0015651 | 0.323 |
| palmitoyl-CoA hydrolase activity | 8 | GO:0016290 | 0.326 |
| sialyltransferase activity | 19 | GO:0008373 | 0.327 |
| prostaglandin E receptor activity | 5 | GO:0004957 | 0.328 |
| glucuronosyltransferase activity | 12 | GO:0015020 | 0.33 |
| ATPase activity | 147 | GO:0016887 | 0.332 |
| protein tyrosine kinase activator activity | 8 | GO:0030296 | 0.332 |
| heparan sulfate proteoglycan binding | 13 | GO:0043395 | 0.332 |
| phosphatidylinositol-3,4-bisphosphate binding | 12 | GO:0043325 | 0.334 |
| Gram-negative bacterial cell surface binding | 5 | GO:0051636 | 0.334 |
| antioxidant activity | 16 | GO:0016209 | 0.335 |
| apolipoprotein binding | 11 | GO:0034185 | 0.336 |
| BMP receptor binding | 7 | GO:0070700 | 0.338 |
| pseudouridine synthase activity | 15 | GO:0009982 | 0.339 |
| organic cation transmembrane transporter activity | 8 | GO:0015101 | 0.342 |
| NADH dehydrogenase (ubiquinone) activity | 38 | GO:0008137 | 0.348 |
| glutamate receptor binding | 9 | GO:0035254 | 0.348 |
| aldo-keto reductase (NADP) activity | 8 | GO:0004033 | 0.349 |
| anion transmembrane transporter activity | 11 | GO:0008509 | 0.351 |
| thyroid hormone transmembrane transporter activity | 5 | GO:0015349 | 0.352 |
| ammonia transmembrane transporter activity | 6 | GO:0051739 | 0.353 |
| ATP-dependent DNA helicase activity | 28 | GO:0004003 | 0.355 |
| NAPE-specific phospholipase D activity | 5 | GO:0070290 | 0.355 |
| acid phosphatase activity | 10 | GO:0003993 | 0.357 |
| tubulin-tyrosine ligase activity | 13 | GO:0004835 | 0.357 |
| cell surface binding | 15 | GO:0043498 | 0.358 |
| transaminase activity | 25 | GO:0008483 | 0.36 |
| high-affinity glutamate transmembrane transporter activity | 5 | GO:0005314 | 0.361 |
| kinase inhibitor activity | 5 | GO:0019210 | 0.362 |
| metallopeptidase activity | 160 | GO:0008237 | 0.365 |
| centromeric DNA binding | 6 | GO:0019237 | 0.365 |
| protein complex binding | 211 | GO:0032403 | 0.365 |
| dopamine receptor activity | 5 | GO:0004952 | 0.368 |
| C2H2 zinc finger domain binding | 11 | GO:0070742 | 0.37 |
| aminoacylase activity | 5 | GO:0004046 | 0.371 |
| lyase activity | 126 | GO:0016829 | 0.371 |
| NADH dehydrogenase activity | 13 | GO:0003954 | 0.373 |
| mannose binding | 16 | GO:0005537 | 0.374 |
| 5'-nucleotidase activity | 9 | GO:0008253 | 0.376 |
| titin binding | 7 | GO:0031432 | 0.376 |
| calcium-dependent protein binding | 40 | GO:0048306 | 0.379 |
| growth hormone-releasing hormone receptor activity | 5 | GO:0016520 | 0.38 |
| hydrolase activity, hydrolyzing O-glycosyl compounds | 32 | GO:0004553 | 0.381 |
| translation activator activity | 7 | GO:0008494 | 0.381 |
| DNA-dependent ATPase activity | 34 | GO:0008094 | 0.382 |
| adrenergic receptor activity | 9 | GO:0004935 | 0.383 |
| ATPase binding | 25 | GO:0051117 | 0.384 |
| sequence-specific enhancer binding RNA polymerase II transcription factor activity | 88 | GO:0003705 | 0.386 |
| secondary active monocarboxylate transmembrane transporter activity | 5 | GO:0015355 | 0.386 |
| type 1 angiotensin receptor binding | 5 | GO:0031702 | 0.388 |
| peptidase activator activity | 10 | GO:0016504 | 0.389 |
| pyridoxal phosphate binding | 54 | GO:0030170 | 0.389 |
| protein tyrosine/threonine phosphatase activity | 5 | GO:0008330 | 0.39 |
| nucleoside transmembrane transporter activity | 6 | GO:0005337 | 0.391 |
| sequence-specific DNA binding RNA polymerase II transcription factor activity | 10 | GO:0000981 | 0.394 |
| Ras GTPase activator activity | 16 | GO:0005099 | 0.394 |
| AU-rich element binding | 12 | GO:0017091 | 0.394 |
| epidermal growth factor receptor binding | 19 | GO:0005154 | 0.395 |
| phosphatidylinositol-3-phosphate binding | 12 | GO:0032266 | 0.395 |
| monosaccharide binding | 14 | GO:0048029 | 0.395 |
| phospholipid-translocating ATPase activity | 15 | GO:0004012 | 0.397 |
| protein kinase B binding | 7 | GO:0043422 | 0.397 |
| virion binding | 6 | GO:0046790 | 0.397 |
| ribose phosphate diphosphokinase activity | 5 | GO:0004749 | 0.401 |
| NAD(P)+-protein-arginine ADP-ribosyltransferase activity | 5 | GO:0003956 | 0.406 |
| interleukin-12 receptor binding | 6 | GO:0005143 | 0.407 |
| neurotransmitter binding | 8 | GO:0042165 | 0.408 |
| guanyl nucleotide binding | 18 | GO:0019001 | 0.409 |
| toxin transporter activity | 6 | GO:0019534 | 0.409 |
| high-density lipoprotein particle binding | 9 | GO:0008035 | 0.41 |
| GDP binding | 36 | GO:0019003 | 0.41 |
| gamma-tubulin binding | 15 | GO:0043015 | 0.41 |
| prenyltransferase activity | 8 | GO:0004659 | 0.412 |
| glutathione binding | 13 | GO:0043295 | 0.412 |
| cofactor binding | 20 | GO:0048037 | 0.412 |
| Rac guanyl-nucleotide exchange factor activity | 8 | GO:0030676 | 0.416 |
| damaged DNA binding | 52 | GO:0003684 | 0.418 |
| flavin-containing monooxygenase activity | 5 | GO:0004499 | 0.418 |
| protein disulfide oxidoreductase activity | 26 | GO:0015035 | 0.418 |
| 1-phosphatidylinositol-3-kinase activity | 12 | GO:0016303 | 0.418 |
| neuropeptide Y receptor activity | 10 | GO:0004983 | 0.419 |
| phosphatidylinositol binding | 64 | GO:0035091 | 0.419 |
| histamine receptor activity | 6 | GO:0004969 | 0.423 |
| ferrous iron binding | 15 | GO:0008198 | 0.423 |
| phenylalanine-tRNA ligase activity | 5 | GO:0004826 | 0.427 |
| oxidoreductase activity, acting on paired donors, with oxidation of a pair of donors resulting in the reduction of molecular oxygen to two molecules of water | 5 | GO:0016717 | 0.427 |
| GPI-anchor transamidase activity | 5 | GO:0003923 | 0.428 |
| protein binding, bridging | 62 | GO:0030674 | 0.432 |
| 1-acylglycerophosphocholine O-acyltransferase activity | 5 | GO:0047184 | 0.433 |
| cytochrome-c oxidase activity | 26 | GO:0004129 | 0.435 |
| androgen binding | 5 | GO:0005497 | 0.435 |
| enzyme activator activity | 45 | GO:0008047 | 0.435 |
| voltage-gated chloride channel activity | 15 | GO:0005247 | 0.437 |
| drug transmembrane transporter activity | 14 | GO:0015238 | 0.438 |
| cyclic-nucleotide phosphodiesterase activity | 7 | GO:0004112 | 0.439 |
| calmodulin-dependent protein kinase activity | 17 | GO:0004683 | 0.443 |
| snoRNP binding | 5 | GO:0030519 | 0.443 |
| iron-sulfur cluster binding | 50 | GO:0051536 | 0.443 |
| heme transporter activity | 6 | GO:0015232 | 0.446 |
| acyl-CoA thioesterase activity | 7 | GO:0016291 | 0.447 |
| specific transcriptional repressor activity | 100 | GO:0016566 | 0.447 |
| translation release factor activity | 6 | GO:0003747 | 0.449 |
| bile acid transmembrane transporter activity | 7 | GO:0015125 | 0.451 |
| hedgehog receptor activity | 8 | GO:0008158 | 0.455 |
| transcription regulatory region DNA binding | 31 | GO:0044212 | 0.458 |
| phospholipid transporter activity | 6 | GO:0005548 | 0.459 |
| alpha-mannosidase activity | 9 | GO:0004559 | 0.46 |
| butyrate-CoA ligase activity | 6 | GO:0047760 | 0.461 |
| mannosidase activity | 6 | GO:0015923 | 0.462 |
| ubiquitin conjugating enzyme binding | 9 | GO:0031624 | 0.462 |
| mismatched DNA binding | 11 | GO:0030983 | 0.464 |
| retinoid binding | 9 | GO:0005501 | 0.466 |
| G-protein alpha-subunit binding | 14 | GO:0001965 | 0.467 |
| manganese ion binding | 38 | GO:0030145 | 0.467 |
| structural molecule activity | 237 | GO:0005198 | 0.469 |
| acetylgalactosaminyltransferase activity | 7 | GO:0008376 | 0.47 |
| oxidoreductase activity, acting on the aldehyde or oxo group of donors, NAD or NADP as acceptor | 8 | GO:0016620 | 0.475 |
| epinephrine binding | 6 | GO:0051379 | 0.483 |
| double-stranded DNA binding | 142 | GO:0003690 | 0.484 |
| retinoic acid receptor binding | 9 | GO:0042974 | 0.484 |
| transcription activator activity | 367 | GO:0016563 | 0.486 |
| growth hormone receptor binding | 6 | GO:0005131 | 0.487 |
| retinoic acid binding | 11 | GO:0001972 | 0.488 |
| carboxylic acid binding | 9 | GO:0031406 | 0.488 |
| MutSalpha complex binding | 5 | GO:0032407 | 0.491 |
| nerve growth factor binding | 5 | GO:0048406 | 0.493 |
| 3'-phosphoadenosine 5'-phosphosulfate binding | 8 | GO:0050656 | 0.494 |
| GTPase inhibitor activity | 13 | GO:0005095 | 0.495 |
| nucleoside-diphosphatase activity | 10 | GO:0017110 | 0.495 |
| inhibitory extracellular ligand-gated ion channel activity | 5 | GO:0005237 | 0.496 |
| retinal binding | 9 | GO:0016918 | 0.503 |
| copper ion binding | 53 | GO:0005507 | 0.506 |
| triglyceride lipase activity | 16 | GO:0004806 | 0.519 |
|  |  |  |  |
| **Cellular Component** |  |  |  |
| cytoplasmic part | 20 | GO:0044444 | 0.008 |
| gap junction | 26 | GO:0005921 | 0.013 |
| connexon complex | 18 | GO:0005922 | 0.018 |
| vesicle | 31 | GO:0031982 | 0.018 |
| dendritic spine membrane | 5 | GO:0032591 | 0.018 |
| intrinsic to internal side of plasma membrane | 11 | GO:0031235 | 0.019 |
| alpha-amino-3-hydroxy-5-methyl-4-isoxazolepropionic acid selective glutamate receptor complex | 13 | GO:0032281 | 0.019 |
| rough endoplasmic reticulum | 36 | GO:0005791 | 0.02 |
| MHC class II protein complex | 12 | GO:0042613 | 0.02 |
| intracellular part | 21 | GO:0044424 | 0.021 |
| intrinsic to membrane | 26 | GO:0031224 | 0.022 |
| phagocytic cup | 11 | GO:0001891 | 0.023 |
| extracellular space | 786 | GO:0005615 | 0.023 |
| NADPH oxidase complex | 10 | GO:0043020 | 0.023 |
| polysome | 20 | GO:0005844 | 0.025 |
| outer dense fiber | 6 | GO:0001520 | 0.029 |
| receptor complex | 20 | GO:0043235 | 0.03 |
| apical plasma membrane | 226 | GO:0016324 | 0.038 |
| nucleosome | 36 | GO:0000786 | 0.04 |
| anchored to plasma membrane | 26 | GO:0046658 | 0.042 |
| sarcomere | 57 | GO:0030017 | 0.043 |
| asymmetric synapse | 12 | GO:0032279 | 0.043 |
| lipopolysaccharide receptor complex | 6 | GO:0046696 | 0.044 |
| transport vesicle membrane | 19 | GO:0030658 | 0.051 |
| myosin filament | 17 | GO:0032982 | 0.051 |
| T cell receptor complex | 12 | GO:0042101 | 0.054 |
| axon terminus | 39 | GO:0043679 | 0.054 |
| voltage-gated calcium channel complex | 24 | GO:0005891 | 0.055 |
| flagellum | 37 | GO:0019861 | 0.056 |
| external side of plasma membrane | 176 | GO:0009897 | 0.057 |
| multivesicular body | 20 | GO:0005771 | 0.059 |
| vacuolar proton-transporting V-type ATPase complex | 11 | GO:0016471 | 0.059 |
| alpha-beta T cell receptor complex | 5 | GO:0042105 | 0.06 |
| nicotinic acetylcholine-gated receptor-channel complex | 17 | GO:0005892 | 0.061 |
| extrinsic to internal side of plasma membrane | 27 | GO:0031234 | 0.064 |
| integral to mitochondrial membrane | 5 | GO:0032592 | 0.064 |
| IkappaB kinase complex | 10 | GO:0008385 | 0.065 |
| photoreceptor outer segment | 34 | GO:0001750 | 0.066 |
| cell surface | 341 | GO:0009986 | 0.068 |
| membrane fraction | 654 | GO:0005624 | 0.072 |
| endocytic vesicle membrane | 29 | GO:0030666 | 0.072 |
| extracellular vesicular exosome | 6 | GO:0070062 | 0.073 |
| pre-autophagosomal structure membrane | 6 | GO:0034045 | 0.075 |
| brush border membrane | 42 | GO:0031526 | 0.077 |
| eukaryotic translation elongation factor 1 complex | 5 | GO:0005853 | 0.079 |
| stereocilium bundle | 7 | GO:0032421 | 0.079 |
| proteasome core complex | 19 | GO:0005839 | 0.086 |
| membrane attack complex | 7 | GO:0005579 | 0.087 |
| B cell receptor complex | 5 | GO:0019815 | 0.088 |
| mitochondrial outer membrane translocase complex | 6 | GO:0005742 | 0.089 |
| MHC class I protein complex | 18 | GO:0042612 | 0.089 |
| terminal button | 49 | GO:0043195 | 0.089 |
| pore complex | 17 | GO:0046930 | 0.089 |
| stored secretory granule | 88 | GO:0030141 | 0.09 |
| CD95 death-inducing signaling complex | 5 | GO:0031265 | 0.09 |
| dendrite membrane | 11 | GO:0032590 | 0.091 |
| brush border | 32 | GO:0005903 | 0.092 |
| proteasome core complex, alpha-subunit complex | 8 | GO:0019773 | 0.093 |
| Arp2/3 protein complex | 8 | GO:0005885 | 0.096 |
| integrin complex | 28 | GO:0008305 | 0.096 |
| nuclear envelope lumen | 9 | GO:0005641 | 0.097 |
| keratin filament | 55 | GO:0045095 | 0.097 |
| platelet alpha granule membrane | 7 | GO:0031092 | 0.098 |
| condensed nuclear chromosome, centromeric region | 10 | GO:0000780 | 0.1 |
| anchored to membrane | 124 | GO:0031225 | 0.1 |
| voltage-gated sodium channel complex | 12 | GO:0001518 | 0.102 |
| late endosome membrane | 60 | GO:0031902 | 0.103 |
| proton-transporting V-type ATPase, V0 domain | 6 | GO:0033179 | 0.104 |
| M band | 8 | GO:0031430 | 0.108 |
| immunological synapse | 15 | GO:0001772 | 0.109 |
| basolateral plasma membrane | 149 | GO:0016323 | 0.111 |
| U12-type spliceosomal complex | 23 | GO:0005689 | 0.112 |
| lysosome | 232 | GO:0005764 | 0.114 |
| vacuole | 14 | GO:0005773 | 0.114 |
| low-density lipoprotein particle | 11 | GO:0034362 | 0.116 |
| proton-transporting two-sector ATPase complex, proton-transporting domain | 10 | GO:0033177 | 0.119 |
| myofibril | 37 | GO:0030016 | 0.121 |
| intermediate filament | 95 | GO:0005882 | 0.123 |
| proteasome complex | 64 | GO:0000502 | 0.127 |
| neuronal cell body | 296 | GO:0043025 | 0.127 |
| contractile fiber | 18 | GO:0043292 | 0.128 |
| lysosomal membrane | 112 | GO:0005765 | 0.129 |
| perikaryon | 58 | GO:0043204 | 0.132 |
| lysosomal lumen | 9 | GO:0043202 | 0.133 |
| nuclear periphery | 6 | GO:0034399 | 0.134 |
| filamentous actin | 22 | GO:0031941 | 0.137 |
| troponin complex | 8 | GO:0005861 | 0.141 |
| symbiont-containing vacuole membrane | 6 | GO:0020005 | 0.144 |
| sodium:potassium-exchanging ATPase complex | 8 | GO:0005890 | 0.145 |
| A band | 9 | GO:0031672 | 0.145 |
| phosphatidylinositol 3-kinase complex | 14 | GO:0005942 | 0.146 |
| node of Ranvier | 7 | GO:0033268 | 0.146 |
| clathrin-coated endocytic vesicle membrane | 5 | GO:0030669 | 0.149 |
| F-actin capping protein complex | 7 | GO:0008290 | 0.151 |
| proton-transporting V-type ATPase, V1 domain | 8 | GO:0033180 | 0.151 |
| septin complex | 12 | GO:0031105 | 0.152 |
| proton-transporting two-sector ATPase complex | 8 | GO:0016469 | 0.153 |
| guanyl-nucleotide exchange factor complex | 5 | GO:0032045 | 0.153 |
| chloride channel complex | 52 | GO:0034707 | 0.153 |
| spectrin-associated cytoskeleton | 5 | GO:0014731 | 0.154 |
| vesicle membrane | 18 | GO:0012506 | 0.156 |
| integral to endoplasmic reticulum membrane | 56 | GO:0030176 | 0.161 |
| internal side of plasma membrane | 40 | GO:0009898 | 0.168 |
| endoplasmic reticulum membrane | 575 | GO:0005789 | 0.169 |
| desmosome | 21 | GO:0030057 | 0.17 |
| multivesicular body membrane | 5 | GO:0032585 | 0.173 |
| hemoglobin complex | 9 | GO:0005833 | 0.174 |
| N-methyl-D-aspartate selective glutamate receptor complex | 8 | GO:0017146 | 0.174 |
| postsynaptic membrane | 171 | GO:0045211 | 0.174 |
| outer membrane-bounded periplasmic space | 10 | GO:0030288 | 0.175 |
| peroxisomal matrix | 30 | GO:0005782 | 0.177 |
| actomyosin contractile ring | 6 | GO:0005826 | 0.179 |
| neuron projection terminus | 9 | GO:0044306 | 0.179 |
| cyclin-dependent protein kinase activating kinase holoenzyme complex | 6 | GO:0019907 | 0.18 |
| endosome membrane | 137 | GO:0010008 | 0.184 |
| presynaptic membrane | 55 | GO:0042734 | 0.184 |
| autophagic vacuole membrane | 14 | GO:0000421 | 0.185 |
| extrinsic to plasma membrane | 20 | GO:0019897 | 0.189 |
| cornified envelope | 18 | GO:0001533 | 0.191 |
| proton-transporting two-sector ATPase complex, catalytic domain | 8 | GO:0033178 | 0.191 |
| very-low-density lipoprotein particle | 17 | GO:0034361 | 0.191 |
| axonemal dynein complex | 13 | GO:0005858 | 0.194 |
| membrane raft | 155 | GO:0045121 | 0.197 |
| microsome | 297 | GO:0005792 | 0.199 |
| Cajal body | 45 | GO:0015030 | 0.204 |
| organelle membrane | 7 | GO:0031090 | 0.207 |
| high-density lipoprotein particle | 21 | GO:0034364 | 0.208 |
| proton-transporting ATP synthase complex, catalytic core F(1) | 6 | GO:0045261 | 0.208 |
| SUN-KASH complex | 6 | GO:0034993 | 0.211 |
| calcium- and calmodulin-dependent protein kinase complex | 8 | GO:0005954 | 0.213 |
| ionotropic glutamate receptor complex | 10 | GO:0008328 | 0.218 |
| T-tubule | 27 | GO:0030315 | 0.222 |
| chylomicron | 10 | GO:0042627 | 0.224 |
| Golgi cisterna | 5 | GO:0031985 | 0.227 |
| central element | 5 | GO:0000801 | 0.23 |
| photoreceptor connecting cilium | 18 | GO:0032391 | 0.232 |
| proton-transporting ATP synthase complex, coupling factor F(o) | 10 | GO:0045263 | 0.232 |
| cell junction | 481 | GO:0030054 | 0.236 |
| transcription factor complex | 249 | GO:0005667 | 0.244 |
| mitochondrial crista | 10 | GO:0030061 | 0.244 |
| alpha DNA polymerase:primase complex | 6 | GO:0005658 | 0.252 |
| varicosity | 5 | GO:0043196 | 0.252 |
| neurofilament | 9 | GO:0005883 | 0.254 |
| COPII vesicle coat | 8 | GO:0030127 | 0.264 |
| nuclear chromosome | 29 | GO:0000228 | 0.266 |
| endosome | 333 | GO:0005768 | 0.266 |
| dendritic shaft | 42 | GO:0043198 | 0.267 |
| nuclear outer membrane | 23 | GO:0005640 | 0.271 |
| cytosolic ribosome | 7 | GO:0022626 | 0.272 |
| cell body fiber | 7 | GO:0070852 | 0.272 |
| Set1C/COMPASS complex | 9 | GO:0048188 | 0.276 |
| photoreceptor inner segment | 20 | GO:0001917 | 0.277 |
| phagocytic vesicle membrane | 11 | GO:0030670 | 0.282 |
| flagellar axoneme | 7 | GO:0035086 | 0.284 |
| nuclear part | 7 | GO:0044428 | 0.284 |
| dendrite | 248 | GO:0030425 | 0.286 |
| synapse | 315 | GO:0045202 | 0.291 |
| replication fork | 13 | GO:0005657 | 0.293 |
| muscle myosin complex | 16 | GO:0005859 | 0.298 |
| cell leading edge | 30 | GO:0031252 | 0.299 |
| mitochondrial respiratory chain complex I | 39 | GO:0005747 | 0.303 |
| COPI vesicle coat | 12 | GO:0030126 | 0.307 |
| centromeric heterochromatin | 7 | GO:0005721 | 0.311 |
| male germ cell nucleus | 19 | GO:0001673 | 0.318 |
| Mre11 complex | 5 | GO:0030870 | 0.321 |
| microvillus membrane | 17 | GO:0031528 | 0.321 |
| podosome | 11 | GO:0002102 | 0.325 |
| cis-Golgi network | 25 | GO:0005801 | 0.326 |
| COPI-coated vesicle | 9 | GO:0030137 | 0.326 |
| spindle microtubule | 35 | GO:0005876 | 0.329 |
| apical cortex | 8 | GO:0045179 | 0.329 |
| paraspeckles | 7 | GO:0042382 | 0.331 |
| peripheral to membrane of membrane fraction | 8 | GO:0000300 | 0.332 |
| DNA-directed RNA polymerase I complex | 5 | GO:0005736 | 0.333 |
| mitochondrial respiratory chain complex III | 6 | GO:0005750 | 0.338 |
| clathrin sculpted gamma-aminobutyric acid transport vesicle membrane | 8 | GO:0061202 | 0.339 |
| synaptic vesicle | 96 | GO:0008021 | 0.34 |
| DNA-directed RNA polymerase II, core complex | 12 | GO:0005665 | 0.343 |
| perinuclear region of cytoplasm | 433 | GO:0048471 | 0.344 |
| soluble fraction | 486 | GO:0005625 | 0.345 |
| platelet dense granule membrane | 5 | GO:0031088 | 0.347 |
| platelet alpha granule | 12 | GO:0031091 | 0.347 |
| synaptobrevin 2-SNAP-25-syntaxin-1a complex | 5 | GO:0070044 | 0.347 |
| uropod | 7 | GO:0001931 | 0.349 |
| mitochondrial proton-transporting ATP synthase complex | 16 | GO:0005753 | 0.349 |
| ESC/E(Z) complex | 9 | GO:0035098 | 0.349 |
| Golgi cis cisterna | 5 | GO:0000137 | 0.352 |
| nuclear euchromatin | 8 | GO:0005719 | 0.36 |
| motile cilium | 11 | GO:0031514 | 0.36 |
| trans-Golgi network transport vesicle | 10 | GO:0030140 | 0.364 |
| cell fraction | 7 | GO:0000267 | 0.365 |
| U7 snRNP | 7 | GO:0005683 | 0.366 |
| signalosome | 10 | GO:0008180 | 0.368 |
| cytoplasmic vesicle membrane | 90 | GO:0030659 | 0.369 |
| cortical cytoskeleton | 28 | GO:0030863 | 0.37 |
| catenin complex | 8 | GO:0016342 | 0.376 |
| peroxisomal membrane | 42 | GO:0005778 | 0.379 |
| DNA-dependent protein kinase-DNA ligase 4 complex | 6 | GO:0005958 | 0.379 |
| Golgi-associated vesicle | 13 | GO:0005798 | 0.381 |
| mitochondrial outer membrane | 107 | GO:0005741 | 0.382 |
| myelin sheath | 22 | GO:0043209 | 0.383 |
| MMXD complex | 5 | GO:0071817 | 0.383 |
| GPI-anchor transamidase complex | 5 | GO:0042765 | 0.386 |
| cytoplasmic vesicle | 352 | GO:0031410 | 0.387 |
| neuromuscular junction | 40 | GO:0031594 | 0.39 |
| P granule | 8 | GO:0043186 | 0.392 |
| fibrinogen complex | 8 | GO:0005577 | 0.394 |
| mitochondrial small ribosomal subunit | 22 | GO:0005763 | 0.394 |
| mitochondrial matrix | 202 | GO:0005759 | 0.397 |
| signal recognition particle, endoplasmic reticulum targeting | 6 | GO:0005786 | 0.398 |
| dynein complex | 32 | GO:0030286 | 0.406 |
| cell body | 19 | GO:0044297 | 0.407 |
| NuA4 histone acetyltransferase complex | 15 | GO:0035267 | 0.416 |
| WINAC complex | 6 | GO:0071778 | 0.417 |
| filopodium membrane | 8 | GO:0031527 | 0.421 |
| interstitial matrix | 14 | GO:0005614 | 0.425 |
| BLOC-1 complex | 7 | GO:0031083 | 0.426 |
| condensin complex | 5 | GO:0000796 | 0.428 |
| melanosome | 88 | GO:0042470 | 0.429 |
| chaperonin-containing T-complex | 7 | GO:0005832 | 0.434 |
| transport vesicle | 48 | GO:0030133 | 0.434 |
| intraflagellar transport particle B | 7 | GO:0030992 | 0.434 |
| MCM complex | 6 | GO:0042555 | 0.435 |
| MutLalpha complex | 5 | GO:0032389 | 0.436 |
| secretory granule membrane | 16 | GO:0030667 | 0.438 |
| chromosome | 247 | GO:0005694 | 0.441 |
| lateral element | 8 | GO:0000800 | 0.443 |
| pyruvate dehydrogenase complex | 7 | GO:0045254 | 0.443 |
| condensed chromosome | 27 | GO:0000793 | 0.446 |
| late endosome | 65 | GO:0005770 | 0.447 |
| acrosomal vesicle | 52 | GO:0001669 | 0.449 |
| extrinsic to external side of plasma membrane | 6 | GO:0031232 | 0.449 |
| spindle pole | 60 | GO:0000922 | 0.451 |
| mitochondrial ribosome | 21 | GO:0005761 | 0.451 |
| voltage-gated potassium channel complex | 90 | GO:0008076 | 0.452 |
| condensed chromosome kinetochore | 60 | GO:0000777 | 0.458 |
| sarcoplasmic reticulum lumen | 5 | GO:0033018 | 0.458 |
| SNARE complex | 24 | GO:0031201 | 0.46 |
| microtubule-based flagellum | 26 | GO:0009434 | 0.461 |
| axolemma | 13 | GO:0030673 | 0.461 |
| spindle midzone | 9 | GO:0051233 | 0.461 |
| proteasome regulatory particle | 8 | GO:0005838 | 0.465 |
| acrosomal membrane | 11 | GO:0002080 | 0.466 |
| pre-snoRNP complex | 6 | GO:0070761 | 0.468 |
| mitochondrial proton-transporting ATP synthase complex, coupling factor F(o) | 8 | GO:0000276 | 0.473 |
| ER to Golgi transport vesicle membrane | 15 | GO:0012507 | 0.474 |
| small nucleolar ribonucleoprotein complex | 10 | GO:0005732 | 0.477 |
| specific granule | 11 | GO:0042581 | 0.477 |
| fascia adherens | 12 | GO:0005916 | 0.478 |
| mRNA cleavage and polyadenylation specificity factor complex | 9 | GO:0005847 | 0.479 |
| clathrin-coated endocytic vesicle | 5 | GO:0045334 | 0.479 |
| dense core granule | 5 | GO:0031045 | 0.481 |
| STAGA complex | 13 | GO:0030914 | 0.483 |
| cell | 5 | GO:0005623 | 0.489 |
| mitochondrial intermembrane space | 37 | GO:0005758 | 0.489 |
| oligosaccharyltransferase complex | 10 | GO:0008250 | 0.493 |
| large ribosomal subunit | 18 | GO:0015934 | 0.493 |
| sarcoplasmic reticulum membrane | 20 | GO:0033017 | 0.493 |
| MLL5-L complex | 9 | GO:0070688 | 0.493 |
| axoneme | 7 | GO:0005930 | 0.494 |
| mitochondrial membrane | 71 | GO:0031966 | 0.495 |
| melanosome membrane | 8 | GO:0033162 | 0.506 |
