# Supplementary material for: Choriodecidual Infection Downregulates Angiogenesis and Morphogenesis Pathways in Fetal Lungs from Macaca Nemestrina
Source: PLoS One. 2012 Oct 9;7(10):e46863. doi: 10.1371/journal.pone.0046863 (PMC3467273; doi:10.1371/journal.pone.0046863)
Supplement: Table S3 — Gene sets downregulated after GBS exposure relative to the control group. (DOCX) [file pone.0046863.s004.docx]

Table S3. Gene sets downregulated after GBS exposure relative to the control group

| Gene set description | Gene set length | Gene Ontology ID | p-value |
| --- | --- | --- | --- |
|  |  |  |  |
| **Biological Process** |  |  |  |
| positive regulation of receptor recycling | 5 | GO:0001921 | 0.008 |
| blood vessel maturation | 5 | GO:0001955 | 0.008 |
| chromatin assembly or disassembly | 38 | GO:0006333 | 0.008 |
| carnitine shuttle | 8 | GO:0006853 | 0.008 |
| small GTPase mediated signal transduction | 301 | GO:0007264 | 0.008 |
| carnitine metabolic process | 7 | GO:0009437 | 0.008 |
| peptidyl-histidine phosphorylation | 5 | GO:0018106 | 0.008 |
| microtubule organizing center organization | 5 | GO:0031023 | 0.008 |
| substrate adhesion-dependent cell spreading | 19 | GO:0034446 | 0.008 |
| protein ubiquitination involved in ubiquitin-dependent protein catabolic process | 45 | GO:0042787 | 0.008 |
| stress fiber assembly | 8 | GO:0043149 | 0.008 |
| cardiac muscle tissue development | 12 | GO:0048738 | 0.008 |
| genitalia development | 5 | GO:0048806 | 0.008 |
| regulation of epithelial cell proliferation | 13 | GO:0050678 | 0.008 |
| limb bud formation | 7 | GO:0060174 | 0.008 |
| neuron projection development | 77 | GO:0031175 | 0.011 |
| regulation of Cdc42 GTPase activity | 7 | GO:0043088 | 0.011 |
| positive regulation of myoblast differentiation | 10 | GO:0045663 | 0.011 |
| regulation of fatty acid oxidation | 19 | GO:0046320 | 0.011 |
| somatic stem cell division | 9 | GO:0048103 | 0.011 |
| luteinization | 9 | GO:0001553 | 0.012 |
| cell fate determination | 19 | GO:0001709 | 0.012 |
| regulation of epithelial cell differentiation | 11 | GO:0030856 | 0.012 |
| embryonic hindlimb morphogenesis | 29 | GO:0035116 | 0.012 |
| morphogenesis of an epithelial sheet | 7 | GO:0002011 | 0.013 |
| polysaccharide metabolic process | 6 | GO:0005976 | 0.013 |
| cell-matrix adhesion | 79 | GO:0007160 | 0.013 |
| cerebral cortex neuron differentiation | 9 | GO:0021895 | 0.013 |
| sprouting angiogenesis | 10 | GO:0002040 | 0.014 |
| regulation of transcription from RNA polymerase III promoter | 8 | GO:0006359 | 0.014 |
| positive regulation of GTPase activity | 116 | GO:0043547 | 0.014 |
| negative regulation of focal adhesion assembly | 5 | GO:0051895 | 0.014 |
| epithelial cell maturation | 6 | GO:0002070 | 0.015 |
| negative regulation of epithelial cell migration | 5 | GO:0010633 | 0.015 |
| hemopoiesis | 70 | GO:0030097 | 0.016 |
| phagocytosis | 35 | GO:0006909 | 0.017 |
| actin filament-based movement | 20 | GO:0030048 | 0.017 |
| endothelial cell migration | 17 | GO:0043542 | 0.017 |
| positive regulation of anti-apoptosis | 47 | GO:0045768 | 0.017 |
| regulation of nitric-oxide synthase activity | 12 | GO:0050999 | 0.017 |
| vasculogenesis | 58 | GO:0001570 | 0.018 |
| regulation of mitotic cell cycle | 23 | GO:0007346 | 0.018 |
| neuroblast proliferation | 16 | GO:0007405 | 0.018 |
| regulation of microtubule-based process | 11 | GO:0032886 | 0.018 |
| negative regulation of Ras protein signal transduction | 17 | GO:0046580 | 0.018 |
| embryonic organ development | 15 | GO:0048568 | 0.018 |
| neuron projection morphogenesis | 36 | GO:0048812 | 0.018 |
| progesterone receptor signaling pathway | 5 | GO:0050847 | 0.018 |
| regulation of endocytosis | 18 | GO:0030100 | 0.019 |
| valine metabolic process | 6 | GO:0006573 | 0.02 |
| negative regulation of endothelial cell migration | 9 | GO:0010596 | 0.02 |
| mammary gland epithelial cell proliferation | 7 | GO:0033598 | 0.02 |
| coenzyme A biosynthetic process | 7 | GO:0015937 | 0.021 |
| mammary gland development | 34 | GO:0030879 | 0.021 |
| inositol phosphate dephosphorylation | 6 | GO:0046855 | 0.021 |
| actin filament polymerization | 15 | GO:0030041 | 0.022 |
| fatty acid beta-oxidation using acyl-CoA dehydrogenase | 6 | GO:0033539 | 0.022 |
| retrograde axon cargo transport | 6 | GO:0008090 | 0.023 |
| insulin-like growth factor receptor signaling pathway | 16 | GO:0048009 | 0.023 |
| Notch receptor processing | 16 | GO:0007220 | 0.024 |
| regulation of vascular endothelial growth factor receptor signaling pathway | 5 | GO:0030947 | 0.024 |
| response to osmotic stress | 20 | GO:0006970 | 0.025 |
| peptidyl-threonine phosphorylation | 24 | GO:0018107 | 0.025 |
| lamellipodium assembly | 20 | GO:0030032 | 0.025 |
| negative regulation of epithelial cell differentiation | 5 | GO:0030857 | 0.025 |
| muscle cell homeostasis | 16 | GO:0046716 | 0.025 |
| microtubule polymerization | 5 | GO:0046785 | 0.025 |
| relaxation of vascular smooth muscle | 5 | GO:0060087 | 0.025 |
| face development | 6 | GO:0060324 | 0.025 |
| natural killer cell differentiation | 9 | GO:0001779 | 0.026 |
| RNA processing | 99 | GO:0006396 | 0.026 |
| cytoskeleton organization | 89 | GO:0007010 | 0.026 |
| positive regulation of Rho protein signal transduction | 6 | GO:0035025 | 0.026 |
| positive regulation of DNA repair | 22 | GO:0045739 | 0.026 |
| regulation of organ growth | 8 | GO:0046620 | 0.026 |
| nuclear-transcribed mRNA catabolic process, deadenylation-dependent decay | 50 | GO:0000288 | 0.027 |
| hair follicle development | 37 | GO:0001942 | 0.027 |
| transcription from RNA polymerase III promoter | 33 | GO:0006383 | 0.027 |
| endosome organization | 18 | GO:0007032 | 0.027 |
| negative regulation of protein complex assembly | 9 | GO:0031333 | 0.027 |
| positive regulation of protein import into nucleus | 5 | GO:0042307 | 0.027 |
| ruffle organization | 14 | GO:0031529 | 0.028 |
| positive regulation of peptidyl-serine phosphorylation | 35 | GO:0033138 | 0.028 |
| histone H4 acetylation | 21 | GO:0043967 | 0.028 |
| positive regulation of positive chemotaxis | 11 | GO:0050927 | 0.028 |
| peptide cross-linking via chondroitin 4-sulfate glycosaminoglycan | 6 | GO:0019800 | 0.029 |
| regulation of multicellular organism growth | 31 | GO:0040014 | 0.029 |
| central nervous system neuron axonogenesis | 6 | GO:0021955 | 0.03 |
| negative regulation of S phase of mitotic cell cycle | 10 | GO:0045749 | 0.03 |
| regulation of peptidyl-tyrosine phosphorylation | 11 | GO:0050730 | 0.03 |
| vagina development | 10 | GO:0060068 | 0.03 |
| histone ubiquitination | 8 | GO:0016574 | 0.031 |
| embryonic arm morphogenesis | 5 | GO:0035117 | 0.031 |
| heart contraction | 10 | GO:0060047 | 0.031 |
| positive regulation of cell migration | 102 | GO:0030335 | 0.032 |
| synaptic vesicle endocytosis | 10 | GO:0048488 | 0.032 |
| histone mRNA catabolic process | 14 | GO:0071044 | 0.032 |
| osteoblast development | 15 | GO:0002076 | 0.033 |
| endocytosis | 138 | GO:0006897 | 0.033 |
| peptidyl-serine phosphorylation | 55 | GO:0018105 | 0.033 |
| positive regulation of JUN kinase activity | 24 | GO:0043507 | 0.033 |
| embryonic morphogenesis | 13 | GO:0048598 | 0.033 |
| regulation of small GTPase mediated signal transduction | 162 | GO:0051056 | 0.033 |
| cyclic nucleotide biosynthetic process | 16 | GO:0009190 | 0.034 |
| endothelial cell differentiation | 9 | GO:0045446 | 0.034 |
| regulation of cholesterol metabolic process | 5 | GO:0090181 | 0.034 |
| microtubule anchoring | 7 | GO:0034453 | 0.035 |
| regulation of protein kinase activity | 16 | GO:0045859 | 0.035 |
| negative regulation of translational initiation | 10 | GO:0045947 | 0.035 |
| nuclear-transcribed mRNA catabolic process | 5 | GO:0000956 | 0.036 |
| JNK cascade | 60 | GO:0007254 | 0.036 |
| positive regulation of endocytosis | 14 | GO:0045807 | 0.036 |
| stress-activated MAPK cascade | 51 | GO:0051403 | 0.036 |
| positive regulation of protein complex assembly | 9 | GO:0031334 | 0.037 |
| activation of MAPKK activity | 43 | GO:0000186 | 0.038 |
| protein polyubiquitination | 57 | GO:0000209 | 0.038 |
| angiogenesis | 176 | GO:0001525 | 0.038 |
| vesicle docking involved in exocytosis | 20 | GO:0006904 | 0.038 |
| negative regulation of microtubule depolymerization | 17 | GO:0007026 | 0.038 |
| regulation of cell migration | 45 | GO:0030334 | 0.039 |
| negative regulation of translation involved in gene silencing by miRNA | 5 | GO:0035278 | 0.039 |
| negative regulation of neurogenesis | 16 | GO:0050768 | 0.039 |
| ovarian follicle development | 51 | GO:0001541 | 0.04 |
| activation of JUN kinase activity | 39 | GO:0007257 | 0.04 |
| interkinetic nuclear migration | 7 | GO:0022027 | 0.04 |
| negative regulation of cell migration | 62 | GO:0030336 | 0.04 |
| actin cytoskeleton reorganization | 27 | GO:0031532 | 0.04 |
| establishment or maintenance of epithelial cell apical/basal polarity | 7 | GO:0045197 | 0.04 |
| nitric oxide metabolic process | 12 | GO:0046209 | 0.04 |
| protein autophosphorylation | 108 | GO:0046777 | 0.04 |
| negative regulation of cell adhesion | 32 | GO:0007162 | 0.041 |
| activation of protein kinase A activity | 16 | GO:0034199 | 0.041 |
| positive regulation of blood vessel endothelial cell migration | 12 | GO:0043536 | 0.041 |
| protein modification process | 147 | GO:0006464 | 0.042 |
| epidermal growth factor receptor signaling pathway | 76 | GO:0007173 | 0.042 |
| protein targeting | 40 | GO:0006605 | 0.043 |
| regulation of catalytic activity | 118 | GO:0050790 | 0.043 |
| limb development | 18 | GO:0060173 | 0.043 |
| selenocysteine incorporation | 7 | GO:0001514 | 0.044 |
| establishment or maintenance of cell polarity | 24 | GO:0007163 | 0.044 |
| intracellular protein kinase cascade | 110 | GO:0007243 | 0.044 |
| primary microRNA processing | 5 | GO:0031053 | 0.044 |
| exonucleolytic nuclear-transcribed mRNA catabolic process involved in deadenylation-dependent decay | 24 | GO:0043928 | 0.044 |
| myeloid cell homeostasis | 7 | GO:0002262 | 0.045 |
| regulation of transcription from RNA polymerase II promoter by nuclear hormone receptor | 58 | GO:0034339 | 0.045 |
| Rac protein signal transduction | 13 | GO:0016601 | 0.046 |
| establishment of cell polarity | 16 | GO:0030010 | 0.046 |
| skeletal muscle thin filament assembly | 6 | GO:0030240 | 0.046 |
| endoderm development | 39 | GO:0007492 | 0.047 |
| rRNA catabolic process | 5 | GO:0016075 | 0.047 |
| cerebral cortex development | 44 | GO:0021987 | 0.047 |
| hemopoietic stem cell proliferation | 8 | GO:0071425 | 0.047 |
| negative regulation of canonical Wnt receptor signaling pathway | 58 | GO:0090090 | 0.047 |
| heart morphogenesis | 41 | GO:0003007 | 0.048 |
| nucleosome disassembly | 9 | GO:0006337 | 0.048 |
| cellular lipid metabolic process | 127 | GO:0044255 | 0.048 |
| nerve growth factor receptor signaling pathway | 216 | GO:0048011 | 0.048 |
| embryonic camera-type eye morphogenesis | 9 | GO:0048596 | 0.048 |
| positive regulation of viral transcription | 44 | GO:0050434 | 0.048 |
| termination of RNA polymerase II transcription | 42 | GO:0006369 | 0.049 |
| microtubule nucleation | 13 | GO:0007020 | 0.049 |
| transmembrane receptor protein tyrosine kinase signaling pathway | 89 | GO:0007169 | 0.05 |
| histone acetylation | 23 | GO:0016573 | 0.05 |
| positive regulation of protein binding | 22 | GO:0032092 | 0.05 |
| mitotic metaphase | 6 | GO:0000089 | 0.051 |
| ovulation from ovarian follicle | 11 | GO:0001542 | 0.051 |
| positive regulation of sodium ion transport | 9 | GO:0010765 | 0.051 |
| positive regulation of Rho GTPase activity | 19 | GO:0032321 | 0.051 |
| positive regulation of catenin import into nucleus | 6 | GO:0035413 | 0.051 |
| germ cell development | 37 | GO:0007281 | 0.052 |
| coenzyme biosynthetic process | 6 | GO:0009108 | 0.052 |
| regulation of acetyl-CoA biosynthetic process from pyruvate | 12 | GO:0010510 | 0.052 |
| artery smooth muscle contraction | 6 | GO:0014824 | 0.052 |
| regulation of fatty acid metabolic process | 8 | GO:0019217 | 0.052 |
| gene silencing by RNA | 30 | GO:0031047 | 0.052 |
| positive regulation of cell-matrix adhesion | 14 | GO:0001954 | 0.053 |
| negative regulation of fat cell differentiation | 23 | GO:0045599 | 0.053 |
| elastic fiber assembly | 7 | GO:0048251 | 0.053 |
| regulation of dendrite morphogenesis | 14 | GO:0048814 | 0.053 |
| nucleotide-excision repair | 66 | GO:0006289 | 0.054 |
| histone H3 acetylation | 36 | GO:0043966 | 0.054 |
| positive regulation of smooth muscle cell migration | 20 | GO:0014911 | 0.055 |
| protein palmitoylation | 8 | GO:0018345 | 0.055 |
| regulation of Rac GTPase activity | 6 | GO:0032314 | 0.055 |
| cellular response to retinoic acid | 33 | GO:0071300 | 0.055 |
| RNA methylation | 5 | GO:0001510 | 0.056 |
| sex differentiation | 26 | GO:0007548 | 0.056 |
| erythrocyte homeostasis | 8 | GO:0034101 | 0.056 |
| protein phosphorylation | 559 | GO:0006468 | 0.057 |
| negative regulation of protein ubiquitination | 15 | GO:0031397 | 0.057 |
| dopaminergic neuron differentiation | 6 | GO:0071542 | 0.057 |
| cochlea morphogenesis | 13 | GO:0090103 | 0.057 |
| organ morphogenesis | 150 | GO:0009887 | 0.058 |
| stem cell development | 5 | GO:0048864 | 0.058 |
| blood vessel development | 53 | GO:0001568 | 0.059 |
| cGMP biosynthetic process | 20 | GO:0006182 | 0.059 |
| positive regulation of protein sumoylation | 8 | GO:0033235 | 0.059 |
| negative regulation of protein kinase B signaling cascade | 14 | GO:0051898 | 0.059 |
| regulation of insulin secretion involved in cellular response to glucose stimulus | 7 | GO:0061178 | 0.059 |
| positive regulation of MAPKKK cascade by fibroblast growth factor receptor signaling pathway | 6 | GO:0090080 | 0.059 |
| negative regulation of protein binding | 19 | GO:0032091 | 0.06 |
| embryonic hemopoiesis | 9 | GO:0035162 | 0.06 |
| negative regulation of MAPKKK cascade | 15 | GO:0043409 | 0.06 |
| positive regulation by host of viral transcription | 6 | GO:0043923 | 0.06 |
| negative regulation of osteoblast differentiation | 26 | GO:0045668 | 0.06 |
| vascular endothelial growth factor receptor signaling pathway | 20 | GO:0048010 | 0.06 |
| myelination | 46 | GO:0042552 | 0.061 |
| chromatin silencing at rDNA | 7 | GO:0000183 | 0.062 |
| lymph vessel development | 10 | GO:0001945 | 0.062 |
| lysosomal transport | 7 | GO:0007041 | 0.062 |
| positive regulation of vascular endothelial growth factor receptor signaling pathway | 12 | GO:0030949 | 0.062 |
| regulation of developmental pigmentation | 7 | GO:0048070 | 0.062 |
| definitive hemopoiesis | 8 | GO:0060216 | 0.062 |
| protein complex assembly | 117 | GO:0006461 | 0.063 |
| negative regulation of axon extension involved in axon guidance | 8 | GO:0048843 | 0.063 |
| convergent extension involved in organogenesis | 5 | GO:0060029 | 0.063 |
| transcription elongation from RNA polymerase II promoter | 61 | GO:0006368 | 0.064 |
| fatty acid elongation, saturated fatty acid | 5 | GO:0019367 | 0.064 |
| maturation of 5.8S rRNA | 5 | GO:0000460 | 0.065 |
| transcription initiation, DNA-dependent | 20 | GO:0006352 | 0.065 |
| protein lipoylation | 5 | GO:0009249 | 0.065 |
| cell migration | 113 | GO:0016477 | 0.065 |
| regulation of actin cytoskeleton organization | 19 | GO:0032956 | 0.065 |
| positive regulation of protein catabolic process | 28 | GO:0045732 | 0.065 |
| somitogenesis | 44 | GO:0001756 | 0.066 |
| positive regulation of MAP kinase activity | 31 | GO:0043406 | 0.066 |
| regulation of embryonic development | 9 | GO:0045995 | 0.066 |
| negative regulation of epithelial cell proliferation | 42 | GO:0050680 | 0.066 |
| focal adhesion assembly | 16 | GO:0048041 | 0.067 |
| reduction of cytosolic calcium ion concentration | 7 | GO:0051481 | 0.067 |
| regulation of transcription from RNA polymerase II promoter | 272 | GO:0006357 | 0.068 |
| cell-cell junction assembly | 13 | GO:0007043 | 0.068 |
| phosphorylation | 629 | GO:0016310 | 0.068 |
| blood vessel morphogenesis | 19 | GO:0048514 | 0.068 |
| platelet activation | 230 | GO:0030168 | 0.069 |
| positive regulation of Ras protein signal transduction | 15 | GO:0046579 | 0.069 |
| ventricular cardiac muscle cell differentiation | 5 | GO:0055012 | 0.069 |
| protein K11-linked ubiquitination | 24 | GO:0070979 | 0.069 |
| myeloid progenitor cell differentiation | 5 | GO:0002318 | 0.07 |
| glomerular basement membrane development | 5 | GO:0032836 | 0.07 |
| positive regulation of lipid biosynthetic process | 5 | GO:0046889 | 0.07 |
| MAPKKK cascade | 58 | GO:0000165 | 0.071 |
| transcription elongation, DNA-dependent | 5 | GO:0006354 | 0.071 |
| transforming growth factor beta receptor signaling pathway | 77 | GO:0007179 | 0.071 |
| mRNA 3'-end processing | 32 | GO:0031124 | 0.071 |
| positive regulation of transcription, DNA-dependent | 372 | GO:0045893 | 0.071 |
| regulation of systemic arterial blood pressure by endothelin | 5 | GO:0003100 | 0.073 |
| fatty acid beta-oxidation | 32 | GO:0006635 | 0.073 |
| positive regulation of epithelial cell migration | 14 | GO:0010634 | 0.073 |
| cellular respiration | 17 | GO:0045333 | 0.073 |
| induction of positive chemotaxis | 16 | GO:0050930 | 0.073 |
| cell migration involved in sprouting angiogenesis | 5 | GO:0002042 | 0.074 |
| cortical actin cytoskeleton organization | 14 | GO:0030866 | 0.074 |
| retrograde transport, endosome to Golgi | 20 | GO:0042147 | 0.074 |
| regulation of fatty acid biosynthetic process | 6 | GO:0042304 | 0.074 |
| protein stabilization | 51 | GO:0050821 | 0.075 |
| tissue homeostasis | 15 | GO:0001894 | 0.076 |
| ubiquitin-dependent protein catabolic process | 149 | GO:0006511 | 0.076 |
| insulin receptor signaling pathway | 148 | GO:0008286 | 0.076 |
| vesicle-mediated transport | 212 | GO:0016192 | 0.076 |
| negative regulation of cell growth | 111 | GO:0030308 | 0.076 |
| negative regulation of translational initiation in response to stress | 5 | GO:0032057 | 0.076 |
| barbed-end actin filament capping | 8 | GO:0051016 | 0.076 |
| regulation of glucose transport | 29 | GO:0010827 | 0.077 |
| dendrite development | 34 | GO:0016358 | 0.077 |
| protein catabolic process | 47 | GO:0030163 | 0.078 |
| cell-substrate adhesion | 7 | GO:0031589 | 0.078 |
| negative regulation of vascular permeability | 7 | GO:0043116 | 0.078 |
| negative regulation of sequence-specific DNA binding transcription factor activity | 46 | GO:0043433 | 0.078 |
| negative regulation of cell differentiation | 32 | GO:0045596 | 0.078 |
| axon extension | 9 | GO:0048675 | 0.078 |
| mammary gland duct morphogenesis | 8 | GO:0060603 | 0.078 |
| histone mRNA 3'-end processing | 6 | GO:0006398 | 0.079 |
| homophilic cell adhesion | 113 | GO:0007156 | 0.079 |
| signal complex assembly | 8 | GO:0007172 | 0.079 |
| response to muscle activity | 9 | GO:0014850 | 0.079 |
| intracellular signal transduction | 319 | GO:0035556 | 0.079 |
| protein oligomerization | 39 | GO:0051259 | 0.079 |
| positive regulation of lamellipodium assembly | 6 | GO:0010592 | 0.08 |
| pre-microRNA processing | 6 | GO:0031054 | 0.081 |
| receptor catabolic process | 5 | GO:0032801 | 0.081 |
| vasoconstriction | 13 | GO:0042310 | 0.081 |
| positive regulation of signal transduction | 15 | GO:0009967 | 0.082 |
| negative regulation of Wnt receptor signaling pathway | 43 | GO:0030178 | 0.082 |
| embryonic placenta development | 24 | GO:0001892 | 0.083 |
| mRNA export from nucleus | 51 | GO:0006406 | 0.083 |
| axonogenesis | 98 | GO:0007409 | 0.083 |
| negative regulation of transcription, DNA-dependent | 298 | GO:0045892 | 0.083 |
| uterus development | 7 | GO:0060065 | 0.083 |
| neuromuscular junction development | 28 | GO:0007528 | 0.085 |
| regulation of cell shape | 65 | GO:0008360 | 0.085 |
| protein transport | 520 | GO:0015031 | 0.085 |
| positive regulation of ATPase activity | 18 | GO:0032781 | 0.085 |
| intracellular protein transport | 205 | GO:0006886 | 0.086 |
| histone deacetylation | 20 | GO:0016575 | 0.086 |
| toll-like receptor 1 signaling pathway | 68 | GO:0034130 | 0.086 |
| toll-like receptor 2 signaling pathway | 68 | GO:0034134 | 0.086 |
| positive regulation of muscle cell differentiation | 26 | GO:0051149 | 0.087 |
| positive regulation of transcription initiation from RNA polymerase II promoter | 5 | GO:0060261 | 0.087 |
| regulation of epidermal growth factor receptor activity | 6 | GO:0007176 | 0.088 |
| Schwann cell development | 5 | GO:0014044 | 0.088 |
| axon guidance | 312 | GO:0007411 | 0.089 |
| histone H4-K5 acetylation | 5 | GO:0043981 | 0.089 |
| histone H4-K8 acetylation | 5 | GO:0043982 | 0.089 |
| positive regulation of S phase of mitotic cell cycle | 6 | GO:0045750 | 0.089 |
| base-excision repair, AP site formation | 8 | GO:0006285 | 0.09 |
| cell cycle arrest | 127 | GO:0007050 | 0.09 |
| G2/M transition DNA damage checkpoint | 16 | GO:0031572 | 0.09 |
| synapse organization | 20 | GO:0050808 | 0.09 |
| negative regulation of protein serine/threonine kinase activity | 8 | GO:0071901 | 0.09 |
| spinal cord dorsal/ventral patterning | 5 | GO:0021513 | 0.091 |
| protein kinase B signaling cascade | 17 | GO:0043491 | 0.091 |
| negative regulation of organ growth | 7 | GO:0046621 | 0.091 |
| positive regulation of canonical Wnt receptor signaling pathway | 41 | GO:0090263 | 0.091 |
| negative regulation of translation | 36 | GO:0017148 | 0.092 |
| cellular response to hormone stimulus | 39 | GO:0032870 | 0.092 |
| histone H4-K12 acetylation | 7 | GO:0043983 | 0.092 |
| cardiac muscle cell proliferation | 8 | GO:0060038 | 0.092 |
| Wnt receptor signaling pathway, planar cell polarity pathway | 11 | GO:0060071 | 0.092 |
| Golgi organization | 26 | GO:0007030 | 0.093 |
| cyclic nucleotide metabolic process | 5 | GO:0009187 | 0.093 |
| negative regulation of cell-substrate adhesion | 9 | GO:0010812 | 0.093 |
| neural crest cell migration | 27 | GO:0001755 | 0.094 |
| glycogen metabolic process | 33 | GO:0005977 | 0.094 |
| mitotic sister chromatid cohesion | 5 | GO:0007064 | 0.095 |
| protein ubiquitination | 197 | GO:0016567 | 0.095 |
| hippo signaling cascade | 12 | GO:0035329 | 0.095 |
| positive regulation of smoothened signaling pathway | 13 | GO:0045880 | 0.095 |
| growth hormone receptor signaling pathway | 8 | GO:0060396 | 0.095 |
| nucleotide-excision repair, DNA damage removal | 21 | GO:0000718 | 0.097 |
| Rho protein signal transduction | 52 | GO:0007266 | 0.097 |
| positive regulation of phosphatidylinositol 3-kinase cascade | 25 | GO:0014068 | 0.098 |
| transcription elongation from RNA polymerase III promoter | 12 | GO:0006385 | 0.1 |
| termination of RNA polymerase III transcription | 12 | GO:0006386 | 0.1 |
| phosphatidylinositol 3-kinase cascade | 11 | GO:0014065 | 0.1 |
| depyrimidination | 5 | GO:0045008 | 0.1 |
| protein O-linked glycosylation | 18 | GO:0006493 | 0.101 |
| response to DNA damage stimulus | 279 | GO:0006974 | 0.101 |
| SMAD protein complex assembly | 8 | GO:0007183 | 0.101 |
| RNA modification | 15 | GO:0009451 | 0.101 |
| secretion by cell | 9 | GO:0032940 | 0.101 |
| branching morphogenesis of a tube | 30 | GO:0048754 | 0.101 |
| negative regulation of stress fiber assembly | 7 | GO:0051497 | 0.101 |
| positive regulation of establishment of protein localization in plasma membrane | 11 | GO:0090004 | 0.101 |
| chromatin modification | 237 | GO:0016568 | 0.102 |
| actin cytoskeleton organization | 140 | GO:0030036 | 0.103 |
| positive regulation of fatty acid beta-oxidation | 6 | GO:0032000 | 0.103 |
| female gamete generation | 17 | GO:0007292 | 0.104 |
| regulation of Rho protein signal transduction | 70 | GO:0035023 | 0.104 |
| regulation of phosphorylation | 29 | GO:0042325 | 0.104 |
| Notch signaling pathway | 59 | GO:0007219 | 0.105 |
| oligodendrocyte development | 23 | GO:0014003 | 0.105 |
| regulation of transforming growth factor beta receptor signaling pathway | 17 | GO:0017015 | 0.106 |
| negative regulation of cell size | 7 | GO:0045792 | 0.106 |
| palate development | 46 | GO:0060021 | 0.106 |
| lateral mesoderm development | 5 | GO:0048368 | 0.107 |
| neural tube formation | 13 | GO:0001841 | 0.108 |
| protein targeting to peroxisome | 9 | GO:0006625 | 0.108 |
| fatty acid metabolic process | 90 | GO:0006631 | 0.108 |
| SMAD protein import into nucleus | 8 | GO:0007184 | 0.108 |
| positive regulation of transforming growth factor beta receptor signaling pathway | 18 | GO:0030511 | 0.108 |
| striated muscle cell differentiation | 14 | GO:0051146 | 0.108 |
| protein K63-linked ubiquitination | 22 | GO:0070534 | 0.108 |
| maintenance of DNA repeat elements | 5 | GO:0043570 | 0.109 |
| MyD88-dependent toll-like receptor signaling pathway | 72 | GO:0002755 | 0.11 |
| energy reserve metabolic process | 97 | GO:0006112 | 0.11 |
| protein sulfation | 6 | GO:0006477 | 0.11 |
| toll-like receptor 3 signaling pathway | 63 | GO:0034138 | 0.11 |
| phosphatidylinositol phosphorylation | 22 | GO:0046854 | 0.11 |
| positive regulation of focal adhesion assembly | 7 | GO:0051894 | 0.11 |
| hippocampus development | 44 | GO:0021766 | 0.111 |
| ovulation cycle | 13 | GO:0042698 | 0.111 |
| cellular response to glucagon stimulus | 35 | GO:0071377 | 0.111 |
| Wnt receptor signaling pathway | 147 | GO:0016055 | 0.112 |
| regulation of transcription elongation, DNA-dependent | 12 | GO:0032784 | 0.112 |
| glucose transport | 63 | GO:0015758 | 0.113 |
| pyramidal neuron development | 6 | GO:0021860 | 0.113 |
| cell-cell junction organization | 58 | GO:0045216 | 0.113 |
| negative regulation of TOR signaling cascade | 5 | GO:0032007 | 0.114 |
| negative chemotaxis | 12 | GO:0050919 | 0.114 |
| auditory receptor cell differentiation | 16 | GO:0042491 | 0.115 |
| histone H4-K16 acetylation | 9 | GO:0043984 | 0.115 |
| negative regulation of myoblast differentiation | 8 | GO:0045662 | 0.115 |
| cardiac myofibril assembly | 11 | GO:0055003 | 0.115 |
| epithelial cell-cell adhesion | 6 | GO:0090136 | 0.115 |
| nuclear-transcribed mRNA catabolic process, nonsense-mediated decay | 27 | GO:0000184 | 0.116 |
| embryonic pattern specification | 21 | GO:0009880 | 0.116 |
| lung development | 108 | GO:0030324 | 0.116 |
| mRNA transport | 69 | GO:0051028 | 0.116 |
| assembly of spliceosomal tri-snRNP | 6 | GO:0000244 | 0.117 |
| protein deacetylation | 13 | GO:0006476 | 0.117 |
| nuclear migration | 7 | GO:0007097 | 0.117 |
| negative regulation of endocytosis | 7 | GO:0045806 | 0.117 |
| phosphatidylinositol metabolic process | 23 | GO:0046488 | 0.117 |
| positive regulation of transcription regulator activity | 9 | GO:0090047 | 0.117 |
| spliceosome assembly | 15 | GO:0000245 | 0.118 |
| cellular membrane fusion | 29 | GO:0006944 | 0.118 |
| synaptic vesicle maturation | 7 | GO:0016188 | 0.118 |
| cerebral cortex cell migration | 8 | GO:0021795 | 0.118 |
| pancreatic juice secretion | 6 | GO:0030157 | 0.118 |
| positive regulation of collagen biosynthetic process | 14 | GO:0032967 | 0.118 |
| embryonic foregut morphogenesis | 10 | GO:0048617 | 0.118 |
| RNA splicing, via transesterification reactions | 23 | GO:0000375 | 0.119 |
| activation of phospholipase C activity | 56 | GO:0007202 | 0.119 |
| negative regulation of DNA replication | 21 | GO:0008156 | 0.119 |
| regulation of hydrogen peroxide metabolic process | 6 | GO:0010310 | 0.119 |
| histone H2B ubiquitination | 7 | GO:0033523 | 0.119 |
| phospholipid biosynthetic process | 51 | GO:0008654 | 0.12 |
| face morphogenesis | 17 | GO:0060325 | 0.12 |
| mesoderm formation | 27 | GO:0001707 | 0.121 |
| transcription initiation from RNA polymerase II promoter | 65 | GO:0006367 | 0.121 |
| segment specification | 5 | GO:0007379 | 0.121 |
| calcium-dependent cell-cell adhesion | 26 | GO:0016339 | 0.121 |
| TOR signaling cascade | 8 | GO:0031929 | 0.121 |
| molybdopterin cofactor biosynthetic process | 6 | GO:0032324 | 0.121 |
| negative regulation of fibroblast growth factor receptor signaling pathway | 10 | GO:0040037 | 0.121 |
| paraxial mesoderm development | 10 | GO:0048339 | 0.121 |
| lymphoid progenitor cell differentiation | 5 | GO:0002320 | 0.122 |
| regulation of glycogen biosynthetic process | 8 | GO:0005979 | 0.123 |
| intra-Golgi vesicle-mediated transport | 17 | GO:0006891 | 0.123 |
| fatty acid beta-oxidation using acyl-CoA oxidase | 11 | GO:0033540 | 0.123 |
| negative regulation of programmed cell death | 11 | GO:0043069 | 0.123 |
| regulation of binding | 5 | GO:0051098 | 0.123 |
| negative regulation of nerve growth factor receptor signaling pathway | 5 | GO:0051387 | 0.123 |
| tRNA modification | 10 | GO:0006400 | 0.124 |
| peptidyl-diphthamide biosynthetic process from peptidyl-histidine | 5 | GO:0017183 | 0.124 |
| thalamus development | 10 | GO:0021794 | 0.124 |
| nose development | 5 | GO:0043584 | 0.124 |
| positive regulation of long-term neuronal synaptic plasticity | 6 | GO:0048170 | 0.125 |
| positive regulation of mast cell chemotaxis | 5 | GO:0060754 | 0.125 |
| peroxisome organization | 17 | GO:0007031 | 0.126 |
| mitotic metaphase/anaphase transition | 13 | GO:0007091 | 0.126 |
| protein deubiquitination | 30 | GO:0016579 | 0.126 |
| long-chain fatty acid metabolic process | 14 | GO:0001676 | 0.127 |
| non-canonical Wnt receptor signaling pathway | 9 | GO:0035567 | 0.127 |
| respiratory system process | 7 | GO:0003016 | 0.128 |
| mitotic cell cycle spindle assembly checkpoint | 30 | GO:0007094 | 0.128 |
| cerebellar Purkinje cell layer development | 9 | GO:0021680 | 0.129 |
| regulation of microtubule polymerization or depolymerization | 5 | GO:0031110 | 0.129 |
| mitotic anaphase | 9 | GO:0000090 | 0.13 |
| adenosine receptor signaling pathway | 8 | GO:0001973 | 0.13 |
| positive regulation of pseudopodium assembly | 9 | GO:0031274 | 0.13 |
| intronless viral mRNA export from host nucleus | 6 | GO:0046784 | 0.13 |
| endochondral ossification | 19 | GO:0001958 | 0.131 |
| muscle organ development | 104 | GO:0007517 | 0.131 |
| ubiquitin-dependent SMAD protein catabolic process | 6 | GO:0030579 | 0.131 |
| protein localization at cell surface | 18 | GO:0034394 | 0.131 |
| positive regulation of Notch signaling pathway | 11 | GO:0045747 | 0.131 |
| negative regulation of heart rate | 6 | GO:0010459 | 0.132 |
| phosphatidylinositol dephosphorylation | 7 | GO:0046856 | 0.132 |
| peptidyl-lysine monomethylation | 6 | GO:0018026 | 0.133 |
| male sex determination | 10 | GO:0030238 | 0.133 |
| cytoskeletal anchoring at nuclear membrane | 5 | GO:0090286 | 0.133 |
| establishment of protein localization | 14 | GO:0045184 | 0.134 |
| tissue remodeling | 9 | GO:0048771 | 0.134 |
| retina homeostasis | 8 | GO:0001895 | 0.135 |
| positive regulation of cGMP biosynthetic process | 9 | GO:0030828 | 0.135 |
| muscle cell differentiation | 35 | GO:0042692 | 0.135 |
| cellular metabolic process | 20 | GO:0044237 | 0.135 |
| auditory receptor cell stereocilium organization | 7 | GO:0060088 | 0.135 |
| mechanoreceptor differentiation | 7 | GO:0042490 | 0.136 |
| nuclear mRNA splicing, via spliceosome | 161 | GO:0000398 | 0.138 |
| in utero embryonic development | 213 | GO:0001701 | 0.138 |
| muscle filament sliding | 38 | GO:0030049 | 0.138 |
| progesterone metabolic process | 5 | GO:0042448 | 0.138 |
| apoptotic cell clearance | 13 | GO:0043277 | 0.138 |
| regulation of protein binding | 11 | GO:0043393 | 0.138 |
| microtubule cytoskeleton organization | 58 | GO:0000226 | 0.139 |
| negative regulation of smoothened signaling pathway | 16 | GO:0045879 | 0.139 |
| protein heterooligomerization | 82 | GO:0051291 | 0.139 |
| branching involved in mammary gland duct morphogenesis | 11 | GO:0060444 | 0.139 |
| MyD88-independent toll-like receptor signaling pathway | 64 | GO:0002756 | 0.14 |
| negative regulation of Rho protein signal transduction | 9 | GO:0035024 | 0.14 |
| regulation of anti-apoptosis | 8 | GO:0045767 | 0.14 |
| neural tube closure | 55 | GO:0001843 | 0.141 |
| cerebral cortex radially oriented cell migration | 5 | GO:0021799 | 0.141 |
| canonical Wnt receptor signaling pathway | 60 | GO:0060070 | 0.142 |
| nuclear-transcribed mRNA poly(A) tail shortening | 24 | GO:0000289 | 0.143 |
| G-protein coupled receptor internalization | 8 | GO:0002031 | 0.143 |
| citrate metabolic process | 6 | GO:0006101 | 0.143 |
| misfolded or incompletely synthesized protein catabolic process | 5 | GO:0006515 | 0.143 |
| learning | 37 | GO:0007612 | 0.143 |
| spinal cord development | 20 | GO:0021510 | 0.143 |
| negative regulation of histone acetylation | 7 | GO:0035067 | 0.143 |
| protein autoubiquitination | 26 | GO:0051865 | 0.143 |
| proteasomal ubiquitin-dependent protein catabolic process | 49 | GO:0043161 | 0.144 |
| positive regulation of cell adhesion | 37 | GO:0045785 | 0.144 |
| positive regulation of ubiquitin-protein ligase activity | 8 | GO:0051443 | 0.144 |
| mRNA processing | 262 | GO:0006397 | 0.145 |
| positive regulation of cell-substrate adhesion | 31 | GO:0010811 | 0.145 |
| wound healing, spreading of epidermal cells | 8 | GO:0035313 | 0.145 |
| dorsal/ventral axis specification | 15 | GO:0009950 | 0.146 |
| negative regulation of chondrocyte differentiation | 11 | GO:0032331 | 0.146 |
| 5S class rRNA transcription from RNA polymerase III type 1 promoter | 6 | GO:0042791 | 0.146 |
| tRNA transcription from RNA polymerase III promoter | 6 | GO:0042797 | 0.146 |
| embryonic skeletal system development | 34 | GO:0048706 | 0.146 |
| positive regulation of axonogenesis | 22 | GO:0050772 | 0.146 |
| response to UV | 38 | GO:0009411 | 0.147 |
| cytoplasmic microtubule organization | 8 | GO:0031122 | 0.147 |
| regulation of smooth muscle cell differentiation | 7 | GO:0051150 | 0.147 |
| establishment of mitotic spindle orientation | 9 | GO:0000132 | 0.149 |
| regulation of exocytosis | 20 | GO:0017157 | 0.149 |
| regulation of mitotic metaphase/anaphase transition | 11 | GO:0030071 | 0.149 |
| actomyosin structure organization | 8 | GO:0031032 | 0.149 |
| response to fluid shear stress | 7 | GO:0034405 | 0.149 |
| forelimb morphogenesis | 11 | GO:0035136 | 0.15 |
| positive regulation of protein kinase B signaling cascade | 40 | GO:0051897 | 0.15 |
| pyrimidine nucleotide metabolic process | 5 | GO:0006220 | 0.151 |
| organelle organization | 14 | GO:0006996 | 0.151 |
| DNA damage checkpoint | 23 | GO:0000077 | 0.152 |
| regulation of Notch signaling pathway | 5 | GO:0008593 | 0.152 |
| sarcomere organization | 15 | GO:0045214 | 0.152 |
| brain morphogenesis | 11 | GO:0048854 | 0.152 |
| mitochondrion morphogenesis | 6 | GO:0070584 | 0.152 |
| dendrite morphogenesis | 17 | GO:0048813 | 0.153 |
| transcription from RNA polymerase II promoter | 289 | GO:0006366 | 0.154 |
| transcription from mitochondrial promoter | 6 | GO:0006390 | 0.154 |
| mitochondrion transport along microtubule | 8 | GO:0047497 | 0.154 |
| lung-associated mesenchyme development | 9 | GO:0060484 | 0.154 |
| peptidyl-lysine methylation | 11 | GO:0018022 | 0.155 |
| negative regulation of epidermal growth factor receptor signaling pathway | 34 | GO:0042059 | 0.155 |
| positive regulation of apoptosis | 207 | GO:0043065 | 0.155 |
| regulation of body fluid levels | 6 | GO:0050878 | 0.155 |
| protein N-linked glycosylation | 17 | GO:0006487 | 0.156 |
| response to aluminum ion | 7 | GO:0010044 | 0.156 |
| extracellular matrix organization | 90 | GO:0030198 | 0.156 |
| cellular response to stimulus | 5 | GO:0051716 | 0.156 |
| DNA methylation | 21 | GO:0006306 | 0.157 |
| transcription termination, DNA-dependent | 5 | GO:0006353 | 0.157 |
| positive regulation of microtubule polymerization | 11 | GO:0031116 | 0.157 |
| glycine catabolic process | 6 | GO:0006546 | 0.158 |
| acetyl-CoA metabolic process | 7 | GO:0006084 | 0.159 |
| telomere maintenance via telomerase | 14 | GO:0007004 | 0.159 |
| negative regulation of insulin secretion | 27 | GO:0046676 | 0.159 |
| Golgi localization | 5 | GO:0051645 | 0.159 |
| Mullerian duct regression | 5 | GO:0001880 | 0.16 |
| positive regulation of epithelial to mesenchymal transition | 17 | GO:0010718 | 0.16 |
| early endosome to late endosome transport | 13 | GO:0045022 | 0.16 |
| retinal rod cell development | 7 | GO:0046548 | 0.16 |
| cellular component organization | 14 | GO:0016043 | 0.161 |
| morphogenesis of embryonic epithelium | 13 | GO:0016331 | 0.161 |
| muscle fiber development | 6 | GO:0048747 | 0.161 |
| G2/M transition of mitotic cell cycle | 105 | GO:0000086 | 0.163 |
| double-strand break repair | 56 | GO:0006302 | 0.163 |
| embryonic limb morphogenesis | 60 | GO:0030326 | 0.163 |
| protein tetramerization | 19 | GO:0051262 | 0.163 |
| hormone-mediated signaling pathway | 21 | GO:0009755 | 0.164 |
| somatic hypermutation of immunoglobulin genes | 10 | GO:0016446 | 0.164 |
| protein homooligomerization | 99 | GO:0051260 | 0.164 |
| negative regulation of transcription from RNA polymerase II promoter | 300 | GO:0000122 | 0.165 |
| lymphangiogenesis | 8 | GO:0001946 | 0.165 |
| muscle contraction | 108 | GO:0006936 | 0.165 |
| protein processing | 25 | GO:0016485 | 0.165 |
| negative regulation of striated muscle tissue development | 9 | GO:0045843 | 0.165 |
| platelet-derived growth factor receptor signaling pathway | 26 | GO:0048008 | 0.165 |
| cell chemotaxis | 15 | GO:0060326 | 0.166 |
| skeletal muscle contraction | 12 | GO:0003009 | 0.167 |
| DNA repair | 287 | GO:0006281 | 0.168 |
| centriole replication | 6 | GO:0007099 | 0.168 |
| somatic recombination of immunoglobulin gene segments | 6 | GO:0016447 | 0.168 |
| negative regulation of DNA recombination | 5 | GO:0045910 | 0.168 |
| centrosome localization | 6 | GO:0051642 | 0.168 |
| positive regulation of bone mineralization | 24 | GO:0030501 | 0.169 |
| adherens junction organization | 34 | GO:0034332 | 0.169 |
| embryonic digit morphogenesis | 43 | GO:0042733 | 0.169 |
| positive regulation of gluconeogenesis | 6 | GO:0045722 | 0.169 |
| mammary gland branching involved in thelarche | 5 | GO:0060744 | 0.169 |
| fatty acid transport | 17 | GO:0015908 | 0.17 |
| L-fucose catabolic process | 9 | GO:0042355 | 0.17 |
| cell death | 145 | GO:0008219 | 0.171 |
| choline metabolic process | 5 | GO:0019695 | 0.171 |
| estrogen receptor signaling pathway | 14 | GO:0030520 | 0.171 |
| centrosome organization | 15 | GO:0051297 | 0.171 |
| Wnt receptor signaling pathway involved in somitogenesis | 6 | GO:0090244 | 0.171 |
| pyrimidine base catabolic process | 7 | GO:0006208 | 0.173 |
| regulation of Wnt receptor signaling pathway | 15 | GO:0030111 | 0.173 |
| salivary gland morphogenesis | 10 | GO:0007435 | 0.174 |
| regulation of G-protein coupled receptor protein signaling pathway | 38 | GO:0008277 | 0.174 |
| protein retention in ER lumen | 9 | GO:0006621 | 0.175 |
| positive regulation of axon extension | 17 | GO:0045773 | 0.175 |
| toll-like receptor 4 signaling pathway | 75 | GO:0034142 | 0.176 |
| glycogen catabolic process | 17 | GO:0005980 | 0.177 |
| protein import into mitochondrial inner membrane | 6 | GO:0045039 | 0.177 |
| regulation of glucose import | 5 | GO:0046324 | 0.177 |
| exocytosis | 73 | GO:0006887 | 0.178 |
| negative regulation of cardiac muscle cell proliferation | 5 | GO:0060044 | 0.178 |
| patterning of blood vessels | 33 | GO:0001569 | 0.179 |
| fertilization | 31 | GO:0009566 | 0.18 |
| positive regulation of neuron projection development | 32 | GO:0010976 | 0.182 |
| Golgi to endosome transport | 9 | GO:0006895 | 0.183 |
| gamete generation | 18 | GO:0007276 | 0.183 |
| pronephros development | 5 | GO:0048793 | 0.183 |
| hexose transport | 41 | GO:0008645 | 0.184 |
| exit from mitosis | 5 | GO:0010458 | 0.184 |
| positive regulation of proteolysis | 13 | GO:0045862 | 0.184 |
| negative regulation of vasoconstriction | 5 | GO:0045906 | 0.184 |
| regulation of cytoskeleton organization | 8 | GO:0051493 | 0.184 |
| autophagic vacuole assembly | 19 | GO:0000045 | 0.185 |
| cobalt ion transport | 6 | GO:0006824 | 0.185 |
| cell junction assembly | 88 | GO:0034329 | 0.185 |
| negative regulation of protein catabolic process | 19 | GO:0042177 | 0.185 |
| long-chain fatty acid biosynthetic process | 5 | GO:0042759 | 0.185 |
| CRD-mediated mRNA stabilization | 5 | GO:0070934 | 0.185 |
| positive regulation of protein homodimerization activity | 5 | GO:0090073 | 0.185 |
| mRNA polyadenylation | 19 | GO:0006378 | 0.186 |
| cholesterol catabolic process | 8 | GO:0006707 | 0.186 |
| Wnt receptor signaling pathway, calcium modulating pathway | 23 | GO:0007223 | 0.186 |
| Toll signaling pathway | 75 | GO:0008063 | 0.186 |
| cell proliferation | 362 | GO:0008283 | 0.186 |
| positive regulation of mitotic cell cycle | 24 | GO:0045931 | 0.186 |
| ureteric bud development | 44 | GO:0001657 | 0.187 |
| blastocyst formation | 5 | GO:0001825 | 0.187 |
| myofibril assembly | 6 | GO:0030239 | 0.187 |
| skin morphogenesis | 7 | GO:0043589 | 0.187 |
| positive regulation of histone H3-K4 methylation | 5 | GO:0051571 | 0.187 |
| pericardium development | 8 | GO:0060039 | 0.187 |
| induction of apoptosis by extracellular signals | 107 | GO:0008624 | 0.189 |
| histone H3-K4 methylation | 15 | GO:0051568 | 0.189 |
| negative regulation of growth | 7 | GO:0045926 | 0.19 |
| response to cocaine | 31 | GO:0042220 | 0.191 |
| regulation of insulin secretion | 75 | GO:0050796 | 0.191 |
| regulation of Rab GTPase activity | 36 | GO:0032313 | 0.192 |
| regulation of neuron differentiation | 25 | GO:0045664 | 0.192 |
| response to copper ion | 25 | GO:0046688 | 0.192 |
| calcium ion import | 6 | GO:0070509 | 0.192 |
| actin filament organization | 44 | GO:0007015 | 0.193 |
| axon midline choice point recognition | 5 | GO:0016199 | 0.193 |
| collagen biosynthetic process | 6 | GO:0032964 | 0.193 |
| positive regulation of helicase activity | 5 | GO:0051096 | 0.193 |
| two-component signal transduction system (phosphorelay) | 7 | GO:0000160 | 0.194 |
| anterior/posterior axis specification, embryo | 10 | GO:0008595 | 0.194 |
| prostate gland epithelium morphogenesis | 11 | GO:0060740 | 0.194 |
| kidney development | 94 | GO:0001822 | 0.195 |
| taurine metabolic process | 6 | GO:0019530 | 0.195 |
| mammary gland epithelium development | 7 | GO:0061180 | 0.195 |
| transcription from RNA polymerase I promoter | 25 | GO:0006360 | 0.196 |
| cell adhesion | 558 | GO:0007155 | 0.196 |
| glycosaminoglycan biosynthetic process | 14 | GO:0006024 | 0.197 |
| regulation of translation | 89 | GO:0006417 | 0.197 |
| Mo-molybdopterin cofactor biosynthetic process | 7 | GO:0006777 | 0.197 |
| histone monoubiquitination | 9 | GO:0010390 | 0.198 |
| positive regulation of protein autophosphorylation | 11 | GO:0031954 | 0.198 |
| melanosome localization | 6 | GO:0032400 | 0.198 |
| meiotic mismatch repair | 6 | GO:0000710 | 0.199 |
| germ cell migration | 10 | GO:0008354 | 0.199 |
| actin filament capping | 20 | GO:0051693 | 0.199 |
| melanosome organization | 13 | GO:0032438 | 0.2 |
| negative regulation of glucose import | 9 | GO:0046325 | 0.2 |
| enzyme linked receptor protein signaling pathway | 9 | GO:0007167 | 0.201 |
| fibroblast migration | 6 | GO:0010761 | 0.201 |
| induction of apoptosis in response to chemical stimulus | 5 | GO:0031558 | 0.201 |
| regulation of protein localization | 33 | GO:0032880 | 0.202 |
| histone lysine methylation | 17 | GO:0034968 | 0.202 |
| heart looping | 36 | GO:0001947 | 0.203 |
| mitochondrial DNA replication | 8 | GO:0006264 | 0.203 |
| response to laminar fluid shear stress | 9 | GO:0034616 | 0.203 |
| microtubule bundle formation | 13 | GO:0001578 | 0.204 |
| outflow tract morphogenesis | 15 | GO:0003151 | 0.204 |
| membrane protein ectodomain proteolysis | 15 | GO:0006509 | 0.204 |
| striatum development | 11 | GO:0021756 | 0.204 |
| positive regulation of caspase activity | 20 | GO:0043280 | 0.204 |
| histone H3-K9 methylation | 7 | GO:0051567 | 0.204 |
| heart development | 186 | GO:0007507 | 0.205 |
| positive regulation of receptor biosynthetic process | 5 | GO:0010870 | 0.205 |
| snRNA processing | 12 | GO:0016180 | 0.205 |
| negative regulation of cell-cell adhesion | 13 | GO:0022408 | 0.205 |
| cell differentiation | 591 | GO:0030154 | 0.206 |
| embryonic forelimb morphogenesis | 21 | GO:0035115 | 0.207 |
| response to corticosterone stimulus | 31 | GO:0051412 | 0.207 |
| regulation of cell adhesion | 34 | GO:0030155 | 0.208 |
| vesicle organization | 9 | GO:0016050 | 0.209 |
| ephrin receptor signaling pathway | 12 | GO:0048013 | 0.209 |
| toll-like receptor signaling pathway | 76 | GO:0002224 | 0.21 |
| lymphocyte differentiation | 6 | GO:0030098 | 0.21 |
| entry of virus into host cell | 8 | GO:0046718 | 0.21 |
| negative regulation of protein kinase activity | 51 | GO:0006469 | 0.211 |
| negative regulation of NF-kappaB import into nucleus | 5 | GO:0042347 | 0.211 |
| sphingomyelin biosynthetic process | 5 | GO:0006686 | 0.212 |
| odontogenesis of dentine-containing tooth | 52 | GO:0042475 | 0.212 |
| vesicle transport along microtubule | 9 | GO:0047496 | 0.212 |
| 3'-phosphoadenosine 5'-phosphosulfate metabolic process | 13 | GO:0050427 | 0.212 |
| metanephros development | 38 | GO:0001656 | 0.213 |
| respiratory tube development | 5 | GO:0030323 | 0.213 |
| inner ear development | 32 | GO:0048839 | 0.213 |
| cardiac muscle cell differentiation | 15 | GO:0055007 | 0.213 |
| hair follicle placode formation | 5 | GO:0060789 | 0.213 |
| acetyl-CoA biosynthetic process from pyruvate | 6 | GO:0006086 | 0.214 |
| receptor internalization | 17 | GO:0031623 | 0.214 |
| steroid hormone mediated signaling pathway | 51 | GO:0043401 | 0.214 |
| bone remodeling | 12 | GO:0046849 | 0.214 |
| morphogenesis of an epithelium | 23 | GO:0002009 | 0.215 |
| ADP biosynthetic process | 6 | GO:0006172 | 0.215 |
| endosome to lysosome transport | 24 | GO:0008333 | 0.215 |
| heme transport | 7 | GO:0015886 | 0.216 |
| peptide biosynthetic process | 7 | GO:0043043 | 0.216 |
| mammary duct terminal end bud growth | 5 | GO:0060763 | 0.216 |
| chaperone cofactor-dependent protein refolding | 5 | GO:0070389 | 0.216 |
| phosphatidic acid biosynthetic process | 6 | GO:0006654 | 0.217 |
| developmental pigmentation | 15 | GO:0048066 | 0.217 |
| epithelium development | 5 | GO:0060429 | 0.217 |
| branching involved in salivary gland morphogenesis | 13 | GO:0060445 | 0.217 |
| cytoskeletal anchoring at plasma membrane | 10 | GO:0007016 | 0.218 |
| regulated secretory pathway | 7 | GO:0045055 | 0.218 |
| negative regulation of hydrolase activity | 6 | GO:0051346 | 0.218 |
| transcription initiation from RNA polymerase I promoter | 25 | GO:0006361 | 0.219 |
| RNA splicing | 256 | GO:0008380 | 0.219 |
| receptor clustering | 17 | GO:0043113 | 0.219 |
| surfactant homeostasis | 7 | GO:0043129 | 0.219 |
| vitamin A metabolic process | 7 | GO:0006776 | 0.22 |
| pyrimidine nucleotide biosynthetic process | 8 | GO:0006221 | 0.221 |
| somite rostral/caudal axis specification | 6 | GO:0032525 | 0.221 |
| histone methylation | 19 | GO:0016571 | 0.222 |
| mammary gland involution | 9 | GO:0060056 | 0.222 |
| embryo development | 134 | GO:0009790 | 0.223 |
| rRNA methylation | 5 | GO:0031167 | 0.223 |
| positive regulation of multicellular organism growth | 28 | GO:0040018 | 0.223 |
| smooth muscle cell differentiation | 16 | GO:0051145 | 0.223 |
| methylation-dependent chromatin silencing | 5 | GO:0006346 | 0.224 |
| protein folding | 177 | GO:0006457 | 0.225 |
| positive regulation of cellular metabolic process | 8 | GO:0031325 | 0.226 |
| positive regulation of histone acetylation | 13 | GO:0035066 | 0.226 |
| lipid phosphorylation | 6 | GO:0046834 | 0.226 |
| peptidyl-tyrosine phosphorylation | 43 | GO:0018108 | 0.227 |
| regulation of blood coagulation | 11 | GO:0030193 | 0.227 |
| positive regulation of cyclin-dependent protein kinase activity involved in G1/S | 5 | GO:0031659 | 0.227 |
| protein peptidyl-prolyl isomerization | 19 | GO:0000413 | 0.229 |
| cell-cell adhesion | 116 | GO:0016337 | 0.229 |
| mRNA modification | 8 | GO:0016556 | 0.229 |
| negative regulation of transforming growth factor beta receptor signaling pathway | 34 | GO:0030512 | 0.229 |
| regulation of protein ubiquitination | 9 | GO:0031396 | 0.23 |
| positive regulation of glucose import | 31 | GO:0046326 | 0.23 |
| primitive streak formation | 5 | GO:0090009 | 0.23 |
| N-acetylglucosamine metabolic process | 15 | GO:0006044 | 0.232 |
| vitamin metabolic process | 54 | GO:0006766 | 0.232 |
| cell projection organization | 47 | GO:0030030 | 0.234 |
| cell morphogenesis involved in differentiation | 8 | GO:0000904 | 0.235 |
| dichotomous subdivision of terminal units involved in salivary gland branching | 5 | GO:0060666 | 0.235 |
| AMP biosynthetic process | 6 | GO:0006167 | 0.236 |
| Golgi to plasma membrane transport | 6 | GO:0006893 | 0.236 |
| anterior/posterior axis specification | 14 | GO:0009948 | 0.236 |
| negative regulation of cyclin-dependent protein kinase activity | 15 | GO:0045736 | 0.236 |
| drug transport | 10 | GO:0015893 | 0.237 |
| lung lobe morphogenesis | 6 | GO:0060463 | 0.237 |
| response to testosterone stimulus | 36 | GO:0033574 | 0.238 |
| sphingolipid metabolic process | 41 | GO:0006665 | 0.239 |
| embryonic organ morphogenesis | 7 | GO:0048562 | 0.239 |
| enucleate erythrocyte differentiation | 6 | GO:0043353 | 0.24 |
| regulation of synaptic plasticity | 33 | GO:0048167 | 0.24 |
| traversing start control point of mitotic cell cycle | 7 | GO:0007089 | 0.241 |
| negative regulation by host of viral transcription | 6 | GO:0043922 | 0.242 |
| pyruvate metabolic process | 21 | GO:0006090 | 0.243 |
| vesicle fusion | 15 | GO:0006906 | 0.243 |
| neuroprotection | 32 | GO:0043526 | 0.243 |
| negative regulation of neuron differentiation | 52 | GO:0045665 | 0.243 |
| activation of MAPK activity | 91 | GO:0000187 | 0.244 |
| cellular protein localization | 25 | GO:0034613 | 0.244 |
| embryonic eye morphogenesis | 7 | GO:0048048 | 0.244 |
| organ induction | 15 | GO:0001759 | 0.245 |
| water-soluble vitamin metabolic process | 46 | GO:0006767 | 0.245 |
| diterpenoid metabolic process | 5 | GO:0016101 | 0.245 |
| negative regulation of mitotic cell cycle | 16 | GO:0045930 | 0.245 |
| cGMP catabolic process | 7 | GO:0046069 | 0.245 |
| cellular response to transforming growth factor beta stimulus | 20 | GO:0071560 | 0.245 |
| proteasome assembly | 5 | GO:0043248 | 0.246 |
| neuromuscular process controlling balance | 35 | GO:0050885 | 0.246 |
| phosphatidylserine metabolic process | 6 | GO:0006658 | 0.247 |
| male genitalia development | 21 | GO:0030539 | 0.247 |
| positive regulation of glycogen biosynthetic process | 13 | GO:0045725 | 0.247 |
| Sertoli cell proliferation | 6 | GO:0060011 | 0.247 |
| regulation of gene expression by genetic imprinting | 14 | GO:0006349 | 0.248 |
| synapse assembly | 38 | GO:0007416 | 0.248 |
| myoblast fusion | 16 | GO:0007520 | 0.248 |
| negative regulation of neuron projection development | 23 | GO:0010977 | 0.248 |
| lateral ventricle development | 7 | GO:0021670 | 0.248 |
| positive regulation of Wnt receptor signaling pathway | 18 | GO:0030177 | 0.248 |
| regulation of fat cell differentiation | 8 | GO:0045598 | 0.248 |
| cellular response to epidermal growth factor stimulus | 6 | GO:0071364 | 0.248 |
| phosphatidylinositol biosynthetic process | 13 | GO:0006661 | 0.249 |
| mitotic metaphase plate congression | 10 | GO:0007080 | 0.249 |
| negative regulation of peptidyl-serine phosphorylation | 10 | GO:0033137 | 0.249 |
| negative regulation of norepinephrine secretion | 7 | GO:0010700 | 0.25 |
| cognition | 16 | GO:0050890 | 0.25 |
| response to transforming growth factor beta stimulus | 5 | GO:0071559 | 0.25 |
| gastrulation with mouth forming second | 21 | GO:0001702 | 0.251 |
| chromatin remodeling | 54 | GO:0006338 | 0.251 |
| carbohydrate transport | 37 | GO:0008643 | 0.251 |
| histone deubiquitination | 12 | GO:0016578 | 0.251 |
| dorsal/ventral neural tube patterning | 15 | GO:0021904 | 0.251 |
| cellular response to growth factor stimulus | 55 | GO:0071363 | 0.251 |
| CTP biosynthetic process | 12 | GO:0006241 | 0.252 |
| positive regulation of stress fiber assembly | 17 | GO:0051496 | 0.252 |
| ATP synthesis coupled proton transport | 16 | GO:0015986 | 0.253 |
| cardiac epithelial to mesenchymal transition | 7 | GO:0060317 | 0.254 |
| activation of transmembrane receptor protein tyrosine kinase activity | 7 | GO:0007171 | 0.255 |
| intra-S DNA damage checkpoint | 5 | GO:0031573 | 0.255 |
| epidermis morphogenesis | 9 | GO:0048730 | 0.255 |
| response to redox state | 7 | GO:0051775 | 0.255 |
| epithelial to mesenchymal transition | 25 | GO:0001837 | 0.256 |
| cardiac cell differentiation | 5 | GO:0035051 | 0.256 |
| aerobic respiration | 14 | GO:0009060 | 0.257 |
| gene expression | 398 | GO:0010467 | 0.257 |
| adult behavior | 14 | GO:0030534 | 0.257 |
| response to folic acid | 12 | GO:0051593 | 0.257 |
| protein import into nucleus | 39 | GO:0006606 | 0.258 |
| positive regulation of mesenchymal cell proliferation | 34 | GO:0002053 | 0.259 |
| positive regulation of gene expression | 103 | GO:0010628 | 0.259 |
| melanosome transport | 11 | GO:0032402 | 0.259 |
| sulfur compound metabolic process | 16 | GO:0006790 | 0.26 |
| negative regulation of phosphatidylinositol 3-kinase cascade | 6 | GO:0014067 | 0.26 |
| fat cell differentiation | 49 | GO:0045444 | 0.26 |
| nervous system development | 424 | GO:0007399 | 0.261 |
| regulation of viral genome replication | 5 | GO:0045069 | 0.261 |
| tongue development | 9 | GO:0043586 | 0.262 |
| negative regulation of JNK cascade | 17 | GO:0046329 | 0.263 |
| embryonic viscerocranium morphogenesis | 8 | GO:0048703 | 0.263 |
| determination of left/right symmetry | 43 | GO:0007368 | 0.264 |
| cellular response to UV | 19 | GO:0034644 | 0.264 |
| anatomical structure regression | 5 | GO:0060033 | 0.264 |
| epithelial tube branching involved in lung morphogenesis | 16 | GO:0060441 | 0.265 |
| chromatin silencing | 15 | GO:0006342 | 0.266 |
| regulation of muscle contraction | 26 | GO:0006937 | 0.266 |
| cell cycle | 522 | GO:0007049 | 0.267 |
| elevation of cytosolic calcium ion concentration involved in G-protein signaling coupled to IP3 second messenger | 16 | GO:0051482 | 0.267 |
| protein K48-linked deubiquitination | 8 | GO:0071108 | 0.267 |
| very long-chain fatty acid metabolic process | 12 | GO:0000038 | 0.268 |
| endocrine pancreas development | 113 | GO:0031018 | 0.268 |
| positive regulation of smooth muscle cell proliferation | 53 | GO:0048661 | 0.268 |
| acrosome assembly | 7 | GO:0001675 | 0.269 |
| blastocyst development | 21 | GO:0001824 | 0.269 |
| positive regulation of endothelial cell migration | 18 | GO:0010595 | 0.269 |
| RNA 3'-end processing | 7 | GO:0031123 | 0.269 |
| regulation of dendrite development | 12 | GO:0050773 | 0.269 |
| sulfate transport | 16 | GO:0008272 | 0.27 |
| notochord development | 9 | GO:0030903 | 0.27 |
| positive regulation of fat cell differentiation | 18 | GO:0045600 | 0.27 |
| venous blood vessel morphogenesis | 5 | GO:0048845 | 0.27 |
| inositol metabolic process | 8 | GO:0006020 | 0.271 |
| positive chemotaxis | 21 | GO:0050918 | 0.271 |
| hemoglobin metabolic process | 5 | GO:0020027 | 0.272 |
| regulation of intestinal cholesterol absorption | 5 | GO:0030300 | 0.272 |
| reproductive structure development | 10 | GO:0048608 | 0.272 |
| chorio-allantoic fusion | 6 | GO:0060710 | 0.272 |
| receptor recycling | 6 | GO:0001881 | 0.273 |
| regulation of translational initiation | 29 | GO:0006446 | 0.273 |
| response to ionizing radiation | 47 | GO:0010212 | 0.273 |
| mitochondrial respiratory chain complex I assembly | 12 | GO:0032981 | 0.273 |
| COPII vesicle coating | 9 | GO:0048208 | 0.273 |
| regulation of defense response to virus | 5 | GO:0050688 | 0.273 |
| rRNA processing | 96 | GO:0006364 | 0.274 |
| microtubule-based movement | 88 | GO:0007018 | 0.274 |
| negative regulation of sodium ion transport | 5 | GO:0010766 | 0.274 |
| DNA integration | 9 | GO:0015074 | 0.274 |
| viral reproduction | 317 | GO:0016032 | 0.274 |
| telencephalon development | 24 | GO:0021537 | 0.274 |
| anatomical structure formation involved in morphogenesis | 19 | GO:0048646 | 0.274 |
| trophectodermal cell differentiation | 13 | GO:0001829 | 0.275 |
| regulation of ARF GTPase activity | 24 | GO:0032312 | 0.275 |
| regulation of cell differentiation | 32 | GO:0045595 | 0.276 |
| protein maturation | 10 | GO:0051604 | 0.276 |
| liver development | 96 | GO:0001889 | 0.277 |
| peptidyl-proline modification | 14 | GO:0018208 | 0.277 |
| positive regulation of striated muscle cell differentiation | 6 | GO:0051155 | 0.277 |
| DNA strand elongation involved in DNA replication | 30 | GO:0006271 | 0.278 |
| cytoplasmic sequestering of transcription factor | 9 | GO:0042994 | 0.278 |
| negative regulation of erythrocyte differentiation | 6 | GO:0045647 | 0.278 |
| alpha-beta T cell differentiation | 7 | GO:0046632 | 0.279 |
| DNA duplex unwinding | 7 | GO:0032508 | 0.28 |
| response to growth factor stimulus | 20 | GO:0070848 | 0.28 |
| stem cell maintenance | 20 | GO:0019827 | 0.281 |
| histone H3-K9 demethylation | 8 | GO:0033169 | 0.281 |
| negative regulation of cell cycle | 45 | GO:0045786 | 0.281 |
| digestive tract development | 24 | GO:0048565 | 0.281 |
| DNA damage response, signal transduction resulting in induction of apoptosis | 20 | GO:0008630 | 0.282 |
| rRNA transcription | 15 | GO:0009303 | 0.282 |
| anterior/posterior pattern formation | 111 | GO:0009952 | 0.282 |
| protein ADP-ribosylation | 23 | GO:0006471 | 0.283 |
| ER to Golgi vesicle-mediated transport | 46 | GO:0006888 | 0.283 |
| osteoblast differentiation | 47 | GO:0001649 | 0.284 |
| positive regulation of histone deacetylation | 8 | GO:0031065 | 0.285 |
| negative regulation of epinephrine secretion | 7 | GO:0032811 | 0.285 |
| somatic stem cell maintenance | 30 | GO:0035019 | 0.285 |
| reverse cholesterol transport | 17 | GO:0043691 | 0.285 |
| tight junction assembly | 27 | GO:0070830 | 0.287 |
| negative regulation of multicellular organism growth | 11 | GO:0040015 | 0.288 |
| calcium ion transport into cytosol | 11 | GO:0060402 | 0.288 |
| spermatid nucleus differentiation | 6 | GO:0007289 | 0.289 |
| positive regulation of transcription factor import into nucleus | 6 | GO:0042993 | 0.289 |
| brain development | 221 | GO:0007420 | 0.29 |
| ribosomal large subunit biogenesis | 10 | GO:0042273 | 0.29 |
| pigmentation | 37 | GO:0043473 | 0.291 |
| response to calcium ion | 65 | GO:0051592 | 0.291 |
| sperm ejaculation | 7 | GO:0042713 | 0.292 |
| development of primary female sexual characteristics | 5 | GO:0046545 | 0.292 |
| CDP-choline pathway | 8 | GO:0006657 | 0.293 |
| positive regulation of dendrite morphogenesis | 15 | GO:0050775 | 0.293 |
| negative regulation of acute inflammatory response | 6 | GO:0002674 | 0.294 |
| negative regulation of phosphorylation | 28 | GO:0042326 | 0.294 |
| succinate metabolic process | 9 | GO:0006105 | 0.295 |
| positive regulation of JAK-STAT cascade | 14 | GO:0046427 | 0.295 |
| cilium morphogenesis | 19 | GO:0060271 | 0.295 |
| positive regulation of protein phosphorylation | 88 | GO:0001934 | 0.296 |
| glial cell differentiation | 21 | GO:0010001 | 0.296 |
| BMP signaling pathway | 43 | GO:0030509 | 0.296 |
| glucose import | 5 | GO:0046323 | 0.296 |
| regulation of myelination | 5 | GO:0031641 | 0.297 |
| positive regulation of transcription from RNA polymerase II promoter | 445 | GO:0045944 | 0.297 |
| regulation of vasodilation | 10 | GO:0042312 | 0.298 |
| glucocorticoid receptor signaling pathway | 9 | GO:0042921 | 0.298 |
| negative regulation of protein phosphorylation | 28 | GO:0001933 | 0.299 |
| intramembranous ossification | 6 | GO:0001957 | 0.299 |
| tetrahydrobiopterin biosynthetic process | 6 | GO:0006729 | 0.299 |
| activation of adenylate cyclase activity by dopamine receptor signaling pathway | 10 | GO:0007191 | 0.299 |
| CenH3-containing nucleosome assembly at centromere | 19 | GO:0034080 | 0.299 |
| methylation | 127 | GO:0032259 | 0.3 |
| inner ear morphogenesis | 61 | GO:0042472 | 0.301 |
| positive regulation of cyclin-dependent protein kinase activity | 8 | GO:0045737 | 0.301 |
| cell division | 284 | GO:0051301 | 0.301 |
| chromatin-mediated maintenance of transcription | 6 | GO:0048096 | 0.302 |
| post-Golgi vesicle-mediated transport | 41 | GO:0006892 | 0.303 |
| UV protection | 11 | GO:0009650 | 0.303 |
| tRNA aminoacylation | 10 | GO:0043039 | 0.303 |
| negative regulation of MHC class II biosynthetic process | 5 | GO:0045347 | 0.303 |
| positive regulation of DNA replication | 39 | GO:0045740 | 0.303 |
| Golgi vesicle transport | 8 | GO:0048193 | 0.303 |
| negative regulation of cytokine-mediated signaling pathway | 6 | GO:0001960 | 0.304 |
| positive regulation of SMAD protein import into nucleus | 7 | GO:0060391 | 0.304 |
| SMAD protein signal transduction | 17 | GO:0060395 | 0.304 |
| salivary gland cavitation | 5 | GO:0060662 | 0.304 |
| 2-oxoglutarate metabolic process | 20 | GO:0006103 | 0.305 |
| mRNA cleavage | 11 | GO:0006379 | 0.305 |
| wound healing | 95 | GO:0042060 | 0.305 |
| embryonic digestive tract development | 16 | GO:0048566 | 0.305 |
| labyrinthine layer development | 8 | GO:0060711 | 0.305 |
| superior temporal gyrus development | 5 | GO:0071109 | 0.305 |
| termination of RNA polymerase I transcription | 21 | GO:0006363 | 0.306 |
| activation of protein kinase B activity | 11 | GO:0032148 | 0.306 |
| limb morphogenesis | 22 | GO:0035108 | 0.306 |
| planar cell polarity pathway involved in neural tube closure | 13 | GO:0090179 | 0.306 |
| NLS-bearing substrate import into nucleus | 13 | GO:0006607 | 0.307 |
| heparan sulfate proteoglycan biosynthetic process | 8 | GO:0015012 | 0.307 |
| multicellular organism growth | 54 | GO:0035264 | 0.307 |
| protein K6-linked ubiquitination | 7 | GO:0085020 | 0.308 |
| multicellular organismal response to stress | 11 | GO:0033555 | 0.309 |
| positive regulation of DNA binding | 19 | GO:0043388 | 0.309 |
| lipid transport | 73 | GO:0006869 | 0.31 |
| negative regulation of platelet activation | 7 | GO:0010544 | 0.31 |
| lactation | 43 | GO:0007595 | 0.311 |
| proteoglycan metabolic process | 8 | GO:0006029 | 0.312 |
| tyrosine catabolic process | 5 | GO:0006572 | 0.312 |
| positive regulation of neuron differentiation | 53 | GO:0045666 | 0.312 |
| positive regulation of transferase activity | 6 | GO:0051347 | 0.312 |
| endocytic recycling | 10 | GO:0032456 | 0.313 |
| negative regulation of telomerase activity | 6 | GO:0051974 | 0.313 |
| electron transport chain | 90 | GO:0022900 | 0.314 |
| positive regulation of translation | 36 | GO:0045727 | 0.314 |
| blood vessel remodeling | 33 | GO:0001974 | 0.315 |
| nucleocytoplasmic transport | 32 | GO:0006913 | 0.315 |
| mRNA metabolic process | 120 | GO:0016071 | 0.317 |
| negative regulation of proteasomal ubiquitin-dependent protein catabolic process | 6 | GO:0032435 | 0.317 |
| oocyte development | 9 | GO:0048599 | 0.317 |
| prostate gland growth | 8 | GO:0060736 | 0.317 |
| retinal ganglion cell axon guidance | 17 | GO:0031290 | 0.318 |
| skin development | 42 | GO:0043588 | 0.318 |
| transmembrane receptor protein serine/threonine kinase signaling pathway | 13 | GO:0007178 | 0.319 |
| blood coagulation, extrinsic pathway | 6 | GO:0007598 | 0.319 |
| axon extension involved in development | 5 | GO:0048676 | 0.319 |
| generation of neurons | 9 | GO:0048699 | 0.319 |
| axon ensheathment | 6 | GO:0008366 | 0.32 |
| regulation of transcription from RNA polymerase II promoter in response to oxidative stress | 6 | GO:0043619 | 0.32 |
| negative regulation of insulin receptor signaling pathway | 19 | GO:0046627 | 0.32 |
| sulfation | 7 | GO:0051923 | 0.32 |
| negative regulation of cell-matrix adhesion | 9 | GO:0001953 | 0.321 |
| dorsal/ventral pattern formation | 47 | GO:0009953 | 0.321 |
| gene silencing | 8 | GO:0016458 | 0.321 |
| negative regulation of nitric oxide biosynthetic process | 8 | GO:0045019 | 0.321 |
| base-excision repair, gap-filling | 7 | GO:0006287 | 0.322 |
| lung morphogenesis | 11 | GO:0060425 | 0.322 |
| vasculature development | 28 | GO:0001944 | 0.323 |
| DNA replication | 147 | GO:0006260 | 0.323 |
| UTP biosynthetic process | 11 | GO:0006228 | 0.324 |
| sphingolipid biosynthetic process | 8 | GO:0030148 | 0.324 |
| male meiosis I | 13 | GO:0007141 | 0.325 |
| negative regulation of gene-specific transcription from RNA polymerase II promoter | 166 | GO:0010553 | 0.325 |
| respiratory electron transport chain | 87 | GO:0022904 | 0.325 |
| endoplasmic reticulum calcium ion homeostasis | 5 | GO:0032469 | 0.325 |
| retrograde vesicle-mediated transport, Golgi to ER | 23 | GO:0006890 | 0.326 |
| sensory cilium assembly | 8 | GO:0035058 | 0.326 |
| spindle assembly | 21 | GO:0051225 | 0.326 |
| adrenergic receptor signaling pathway | 8 | GO:0071875 | 0.326 |
| negative regulation of neuron apoptosis | 83 | GO:0043524 | 0.327 |
| regulation of glycogen catabolic process | 6 | GO:0005981 | 0.328 |
| neuron maturation | 10 | GO:0042551 | 0.328 |
| positive regulation of keratinocyte differentiation | 5 | GO:0045618 | 0.328 |
| response to arsenic-containing substance | 18 | GO:0046685 | 0.328 |
| protein polymerization | 27 | GO:0051258 | 0.328 |
| chondroitin sulfate biosynthetic process | 8 | GO:0030206 | 0.329 |
| actin filament bundle assembly | 22 | GO:0051017 | 0.329 |
| neuron development | 36 | GO:0048666 | 0.33 |
| negative regulation of cellular component movement | 7 | GO:0051271 | 0.33 |
| regulation of DNA replication | 11 | GO:0006275 | 0.331 |
| male meiosis | 13 | GO:0007140 | 0.331 |
| thyroid gland development | 19 | GO:0030878 | 0.331 |
| cytoplasmic mRNA processing body assembly | 5 | GO:0033962 | 0.331 |
| positive regulation of sequence-specific DNA binding transcription factor activity | 53 | GO:0051091 | 0.332 |
| endothelial cell morphogenesis | 8 | GO:0001886 | 0.333 |
| GTP biosynthetic process | 12 | GO:0006183 | 0.334 |
| amine metabolic process | 7 | GO:0009308 | 0.334 |
| protein targeting to lysosome | 8 | GO:0006622 | 0.335 |
| negative regulation of epithelial to mesenchymal transition | 9 | GO:0010719 | 0.335 |
| regulation of RNA splicing | 14 | GO:0043484 | 0.335 |
| regulation of smoothened signaling pathway | 13 | GO:0008589 | 0.336 |
| dopamine transport | 6 | GO:0015872 | 0.336 |
| cardiac muscle fiber development | 7 | GO:0048739 | 0.336 |
| cell morphogenesis | 52 | GO:0000902 | 0.337 |
| morphogenesis of a branching structure | 8 | GO:0001763 | 0.337 |
| negative regulation of endothelial cell proliferation | 19 | GO:0001937 | 0.337 |
| central nervous system development | 131 | GO:0007417 | 0.337 |
| response to pH | 17 | GO:0009268 | 0.337 |
| regulation of receptor activity | 13 | GO:0010469 | 0.337 |
| negative regulation of ossification | 12 | GO:0030279 | 0.337 |
| negative regulation of synaptic transmission, GABAergic | 8 | GO:0032229 | 0.337 |
| lipid homeostasis | 16 | GO:0055088 | 0.337 |
| plasma membrane repair | 6 | GO:0001778 | 0.338 |
| spongiotrophoblast layer development | 5 | GO:0060712 | 0.339 |
| meiosis | 73 | GO:0007126 | 0.34 |
| negative regulation of bone mineralization | 10 | GO:0030502 | 0.34 |
| positive regulation of interleukin-1 beta production | 6 | GO:0032731 | 0.34 |
| retinoic acid receptor signaling pathway | 16 | GO:0048384 | 0.34 |
| visual learning | 35 | GO:0008542 | 0.341 |
| fibril organization | 7 | GO:0043206 | 0.341 |
| phosphatidylinositol-mediated signaling | 71 | GO:0048015 | 0.341 |
| nucleoside diphosphate phosphorylation | 8 | GO:0006165 | 0.342 |
| DNA metabolic process | 36 | GO:0006259 | 0.342 |
| olfactory bulb development | 16 | GO:0021772 | 0.342 |
| smoothened signaling pathway | 36 | GO:0007224 | 0.343 |
| response to iron ion | 19 | GO:0010039 | 0.343 |
| negative regulation of axonogenesis | 18 | GO:0050771 | 0.344 |
| urogenital system development | 12 | GO:0001655 | 0.345 |
| phosphatidylethanolamine biosynthetic process | 6 | GO:0006646 | 0.345 |
| positive regulation of transcription from RNA polymerase II promoter during mitosis | 5 | GO:0046022 | 0.345 |
| regulation of branching involved in mammary gland duct morphogenesis | 5 | GO:0060762 | 0.345 |
| DNA fragmentation involved in apoptotic nuclear change | 25 | GO:0006309 | 0.346 |
| smooth muscle contraction | 18 | GO:0006939 | 0.346 |
| synaptonemal complex assembly | 13 | GO:0007130 | 0.346 |
| cerebellar cortex morphogenesis | 5 | GO:0021696 | 0.346 |
| positive regulation of lipoprotein lipase activity | 9 | GO:0051006 | 0.346 |
| ectoderm development | 18 | GO:0007398 | 0.347 |
| regulation of gene expression, epigenetic | 5 | GO:0040029 | 0.347 |
| negative regulation of coagulation | 6 | GO:0050819 | 0.347 |
| DNA dealkylation involved in DNA repair | 6 | GO:0006307 | 0.348 |
| regulation of exit from mitosis | 8 | GO:0007096 | 0.348 |
| integrin-mediated signaling pathway | 82 | GO:0007229 | 0.348 |
| long-term memory | 22 | GO:0007616 | 0.348 |
| mesenchymal to epithelial transition | 6 | GO:0060231 | 0.349 |
| estrogen metabolic process | 11 | GO:0008210 | 0.35 |
| negative regulation of BMP signaling pathway | 28 | GO:0030514 | 0.35 |
| reactive oxygen species metabolic process | 22 | GO:0072593 | 0.351 |
| DNA demethylation | 5 | GO:0080111 | 0.351 |
| positive regulation of cardiac muscle cell proliferation | 11 | GO:0060045 | 0.352 |
| positive regulation of membrane protein ectodomain proteolysis | 11 | GO:0051044 | 0.353 |
| regulation of sodium ion transport | 11 | GO:0002028 | 0.354 |
| neurofilament cytoskeleton organization | 9 | GO:0060052 | 0.354 |
| mRNA splice site selection | 10 | GO:0006376 | 0.355 |
| chromosome segregation | 60 | GO:0007059 | 0.355 |
| regulation of gene-specific transcription from RNA polymerase II promoter | 31 | GO:0010551 | 0.355 |
| developmental growth | 26 | GO:0048589 | 0.355 |
| positive regulation of cell migration involved in sprouting angiogenesis | 5 | GO:0090050 | 0.355 |
| phosphatidylcholine biosynthetic process | 15 | GO:0006656 | 0.356 |
| response to growth hormone stimulus | 12 | GO:0060416 | 0.356 |
| tricarboxylic acid cycle | 23 | GO:0006099 | 0.357 |
| metabotropic glutamate receptor signaling pathway | 6 | GO:0007216 | 0.357 |
| dorsal spinal cord development | 5 | GO:0021516 | 0.357 |
| regulation of vascular permeability | 7 | GO:0043114 | 0.357 |
| trophoblast giant cell differentiation | 9 | GO:0060707 | 0.357 |
| transmembrane receptor protein tyrosine phosphatase signaling pathway | 8 | GO:0007185 | 0.359 |
| immunoglobulin V(D)J recombination | 5 | GO:0033152 | 0.36 |
| positive regulation of MAPKKK cascade | 60 | GO:0043410 | 0.36 |
| regulation of neurogenesis | 6 | GO:0050767 | 0.36 |
| tRNA processing | 73 | GO:0008033 | 0.361 |
| regulation of vasoconstriction | 19 | GO:0019229 | 0.361 |
| filopodium assembly | 24 | GO:0046847 | 0.361 |
| myoblast proliferation | 5 | GO:0051450 | 0.361 |
| cytokinesis | 54 | GO:0000910 | 0.362 |
| maintenance of protein location in nucleus | 11 | GO:0051457 | 0.363 |
| determination of adult lifespan | 13 | GO:0008340 | 0.364 |
| protein localization to nucleus | 16 | GO:0034504 | 0.364 |
| regulation of osteoblast differentiation | 9 | GO:0045667 | 0.364 |
| intracellular transport | 22 | GO:0046907 | 0.364 |
| branching involved in ureteric bud morphogenesis | 43 | GO:0001658 | 0.366 |
| neuron migration | 80 | GO:0001764 | 0.366 |
| protein dephosphorylation | 129 | GO:0006470 | 0.366 |
| negative regulation of cell proliferation | 350 | GO:0008285 | 0.366 |
| potassium ion import | 5 | GO:0010107 | 0.366 |
| negative regulation of B cell differentiation | 6 | GO:0045578 | 0.366 |
| transcription-coupled nucleotide-excision repair | 43 | GO:0006283 | 0.367 |
| regulation of signal transduction | 25 | GO:0009966 | 0.367 |
| biomineral tissue development | 18 | GO:0031214 | 0.367 |
| cellular response to amino acid stimulus | 33 | GO:0071230 | 0.367 |
| positive regulation of urine volume | 7 | GO:0035810 | 0.368 |
| cell aging | 32 | GO:0007569 | 0.369 |
| post-embryonic development | 81 | GO:0009791 | 0.369 |
| removal of superoxide radicals | 11 | GO:0019430 | 0.369 |
| microtubule-based process | 35 | GO:0007017 | 0.37 |
| cellular response to starvation | 24 | GO:0009267 | 0.37 |
| positive regulation of kidney development | 7 | GO:0090184 | 0.37 |
| nucleobase, nucleoside and nucleotide interconversion | 18 | GO:0015949 | 0.371 |
| histone H3-K36 demethylation | 5 | GO:0070544 | 0.371 |
| virus-host interaction | 10 | GO:0019048 | 0.372 |
| ATP-dependent chromatin remodeling | 5 | GO:0043044 | 0.372 |
| response to antibiotic | 39 | GO:0046677 | 0.372 |
| mammary gland epithelial cell differentiation | 9 | GO:0060644 | 0.372 |
| embryonic axis specification | 6 | GO:0000578 | 0.373 |
| fructose metabolic process | 9 | GO:0006000 | 0.373 |
| regulation of cell cycle | 91 | GO:0051726 | 0.373 |
| T cell receptor V(D)J recombination | 5 | GO:0033153 | 0.374 |
| regulation of Ras protein signal transduction | 6 | GO:0046578 | 0.374 |
| COPI coating of Golgi vesicle | 10 | GO:0048205 | 0.374 |
| anatomical structure development | 12 | GO:0048856 | 0.374 |
| neuron apoptosis | 25 | GO:0051402 | 0.374 |
| drug transmembrane transport | 14 | GO:0006855 | 0.375 |
| regulation of MAPKKK cascade | 12 | GO:0043408 | 0.375 |
| midgut development | 15 | GO:0007494 | 0.376 |
| fatty acid oxidation | 16 | GO:0019395 | 0.376 |
| activation of JNKK activity | 7 | GO:0007256 | 0.377 |
| hexose transmembrane transport | 7 | GO:0035428 | 0.377 |
| cellular response to vitamin D | 9 | GO:0071305 | 0.377 |
| mismatch repair | 18 | GO:0006298 | 0.378 |
| mitochondrial fission | 6 | GO:0000266 | 0.379 |
| nuclear envelope organization | 6 | GO:0006998 | 0.379 |
| central nervous system myelination | 9 | GO:0022010 | 0.379 |
| steroid hormone receptor signaling pathway | 17 | GO:0030518 | 0.379 |
| cellular amino acid biosynthetic process | 36 | GO:0008652 | 0.38 |
| positive regulation of gamma-aminobutyric acid secretion | 6 | GO:0014054 | 0.38 |
| DNA-dependent DNA replication | 20 | GO:0006261 | 0.381 |
| glucocorticoid metabolic process | 7 | GO:0008211 | 0.381 |
| proximal/distal pattern formation | 23 | GO:0009954 | 0.381 |
| synaptic vesicle docking involved in exocytosis | 9 | GO:0016081 | 0.381 |
| cardiac muscle tissue morphogenesis | 7 | GO:0055008 | 0.381 |
| peptidyl-glutamic acid carboxylation | 11 | GO:0017187 | 0.382 |
| positive regulation of ossification | 17 | GO:0045778 | 0.383 |
| spermatogenesis | 348 | GO:0007283 | 0.384 |
| response to alkaloid | 9 | GO:0043279 | 0.384 |
| regulation of sequence-specific DNA binding transcription factor activity | 55 | GO:0051090 | 0.384 |
| activation of protein kinase activity | 20 | GO:0032147 | 0.385 |
| hypothalamus development | 11 | GO:0021854 | 0.387 |
| forebrain development | 76 | GO:0030900 | 0.387 |
| DNA damage response, detection of DNA damage | 7 | GO:0042769 | 0.387 |
| negative regulation of apoptosis | 271 | GO:0043066 | 0.387 |
| cellular response to lithium ion | 17 | GO:0071285 | 0.387 |
| calcium-independent cell-cell adhesion | 24 | GO:0016338 | 0.388 |
| cellular response to alkaloid | 6 | GO:0071312 | 0.388 |
| ethanol oxidation | 11 | GO:0006069 | 0.389 |
| pentose-phosphate shunt | 11 | GO:0006098 | 0.39 |
| cellular amino acid metabolic process | 52 | GO:0006520 | 0.39 |
| synaptic vesicle transport | 9 | GO:0048489 | 0.39 |
| cell motility | 9 | GO:0048870 | 0.39 |
| positive regulation of cellular component movement | 13 | GO:0051272 | 0.39 |
| pathway-restricted SMAD protein phosphorylation | 10 | GO:0060389 | 0.391 |
| DNA replication checkpoint | 8 | GO:0000076 | 0.392 |
| ribosomal protein import into nucleus | 6 | GO:0006610 | 0.392 |
| nucleus organization | 8 | GO:0006997 | 0.392 |
| regulation of glucose metabolic process | 5 | GO:0010906 | 0.393 |
| regulation of phosphoprotein phosphatase activity | 10 | GO:0043666 | 0.393 |
| cell development | 23 | GO:0048468 | 0.393 |
| translational initiation | 60 | GO:0006413 | 0.395 |
| positive regulation of peptidyl-threonine phosphorylation | 11 | GO:0010800 | 0.395 |
| cellular response to indole-3-methanol | 5 | GO:0071681 | 0.395 |
| regulation of striated muscle tissue development | 6 | GO:0016202 | 0.396 |
| regulation of bone mineralization | 11 | GO:0030500 | 0.396 |
| myoblast differentiation | 13 | GO:0045445 | 0.396 |
| detection of mechanical stimulus involved in sensory perception of pain | 8 | GO:0050966 | 0.396 |
| double-strand break repair via homologous recombination | 34 | GO:0000724 | 0.397 |
| anterograde axon cargo transport | 6 | GO:0008089 | 0.397 |
| negative regulation of signal transduction | 53 | GO:0009968 | 0.397 |
| respiratory system development | 6 | GO:0060541 | 0.397 |
| positive regulation of protein import into nucleus, translocation | 8 | GO:0033160 | 0.398 |
| camera-type eye morphogenesis | 18 | GO:0048593 | 0.398 |
| cellular response to gamma radiation | 8 | GO:0071480 | 0.398 |
| inhibition of adenylate cyclase activity by dopamine receptor signaling pathway | 5 | GO:0007195 | 0.399 |
| cytidine deamination | 7 | GO:0009972 | 0.4 |
| thymus development | 28 | GO:0048538 | 0.401 |
| base-excision repair | 33 | GO:0006284 | 0.402 |
| response to radiation | 36 | GO:0009314 | 0.402 |
| epithelial cell proliferation | 12 | GO:0050673 | 0.402 |
| cell growth | 54 | GO:0016049 | 0.403 |
| negative regulation of catenin import into nucleus | 8 | GO:0035414 | 0.403 |
| ventricular septum development | 8 | GO:0003281 | 0.404 |
| learning or memory | 48 | GO:0007611 | 0.405 |
| regulation of JNK cascade | 11 | GO:0046328 | 0.405 |
| lung alveolus development | 34 | GO:0048286 | 0.405 |
| positive regulation of cardiac muscle hypertrophy | 5 | GO:0010613 | 0.406 |
| L-phenylalanine catabolic process | 9 | GO:0006559 | 0.407 |
| positive regulation of gene-specific transcription from RNA polymerase II promoter | 203 | GO:0010552 | 0.407 |
| cellular protein complex assembly | 13 | GO:0043623 | 0.408 |
| polyamine metabolic process | 12 | GO:0006595 | 0.409 |
| branched chain family amino acid catabolic process | 18 | GO:0009083 | 0.409 |
| hair follicle morphogenesis | 25 | GO:0031069 | 0.41 |
| negative regulation of insulin-like growth factor receptor signaling pathway | 6 | GO:0043569 | 0.41 |
| mRNA capping | 29 | GO:0006370 | 0.411 |
| protein import into peroxisome matrix | 9 | GO:0016558 | 0.411 |
| Leydig cell differentiation | 15 | GO:0033327 | 0.411 |
| response to interleukin-6 | 6 | GO:0070741 | 0.411 |
| spermatid development | 57 | GO:0007286 | 0.412 |
| protein secretion | 18 | GO:0009306 | 0.412 |
| neural tube development | 31 | GO:0021915 | 0.412 |
| hemidesmosome assembly | 12 | GO:0031581 | 0.412 |
| susceptibility to natural killer cell mediated cytotoxicity | 5 | GO:0042271 | 0.412 |
| mesenchymal cell differentiation | 8 | GO:0048762 | 0.412 |
| hard palate development | 5 | GO:0060022 | 0.412 |
| ERK1 and ERK2 cascade | 14 | GO:0070371 | 0.412 |
| glycoprotein catabolic process | 8 | GO:0006516 | 0.413 |
| hindbrain development | 21 | GO:0030902 | 0.413 |
| GTP catabolic process | 185 | GO:0006184 | 0.414 |
| ceramide metabolic process | 16 | GO:0006672 | 0.414 |
| blastocyst growth | 5 | GO:0001832 | 0.415 |
| positive regulation of pathway-restricted SMAD protein phosphorylation | 16 | GO:0010862 | 0.415 |
| cilium assembly | 37 | GO:0042384 | 0.415 |
| skeletal system development | 149 | GO:0001501 | 0.417 |
| regulation of angiogenesis | 24 | GO:0045765 | 0.417 |
| tetrahydrofolate metabolic process | 8 | GO:0046653 | 0.417 |
| retina development in camera-type eye | 60 | GO:0060041 | 0.418 |
| platelet dense granule organization | 8 | GO:0060155 | 0.418 |
| cellular amino acid catabolic process | 5 | GO:0009063 | 0.419 |
| peripheral nervous system axon regeneration | 12 | GO:0014012 | 0.419 |
| maternal process involved in parturition | 6 | GO:0060137 | 0.419 |
| cell-substrate junction assembly | 6 | GO:0007044 | 0.421 |
| female meiosis | 7 | GO:0007143 | 0.421 |
| very-low-density lipoprotein particle remodeling | 7 | GO:0034372 | 0.421 |
| triglyceride mobilization | 6 | GO:0006642 | 0.422 |
| heme biosynthetic process | 18 | GO:0006783 | 0.422 |
| response to amino acid stimulus | 48 | GO:0043200 | 0.422 |
| carnitine biosynthetic process | 5 | GO:0045329 | 0.422 |
| regulation of ryanodine-sensitive calcium-release channel activity | 6 | GO:0060314 | 0.422 |
| sensory perception of sound | 110 | GO:0007605 | 0.423 |
| melanocyte differentiation | 21 | GO:0030318 | 0.423 |
| resolution of meiotic recombination intermediates | 5 | GO:0000712 | 0.424 |
| female pregnancy | 91 | GO:0007565 | 0.424 |
| neurotransmitter transport | 47 | GO:0006836 | 0.425 |
| cellular metal ion homeostasis | 5 | GO:0006875 | 0.425 |
| negative regulation of cartilage development | 7 | GO:0061037 | 0.425 |
| ossification | 87 | GO:0001503 | 0.426 |
| positive regulation of cholesterol biosynthetic process | 7 | GO:0045542 | 0.426 |
| synaptic transmission, dopaminergic | 12 | GO:0001963 | 0.427 |
| histidine catabolic process | 6 | GO:0006548 | 0.427 |
| androgen receptor signaling pathway | 41 | GO:0030521 | 0.427 |
| positive regulation of synaptogenesis | 13 | GO:0051965 | 0.427 |
| labyrinthine layer blood vessel development | 15 | GO:0060716 | 0.428 |
| mitotic cell cycle checkpoint | 13 | GO:0007093 | 0.429 |
| positive regulation of cell growth | 61 | GO:0030307 | 0.429 |
| protein trimerization | 7 | GO:0070206 | 0.429 |
| regulation of the force of heart contraction | 18 | GO:0002026 | 0.43 |
| peptidyl-arginine N-methylation | 6 | GO:0035246 | 0.43 |
| spermatid differentiation | 5 | GO:0048515 | 0.43 |
| regulation of synaptic transmission | 25 | GO:0050804 | 0.43 |
| protein localization to chromosome, telomeric region | 6 | GO:0070198 | 0.43 |
| lung vasculature development | 7 | GO:0060426 | 0.431 |
| bone trabecula formation | 6 | GO:0060346 | 0.433 |
| pachytene | 7 | GO:0000239 | 0.434 |
| regulation of growth | 73 | GO:0040008 | 0.434 |
| positive regulation of hormone secretion | 12 | GO:0046887 | 0.434 |
| protein heterotetramerization | 8 | GO:0051290 | 0.435 |
| cholesterol homeostasis | 50 | GO:0042632 | 0.436 |
| cerebellum morphogenesis | 6 | GO:0021587 | 0.437 |
| positive regulation of blood coagulation | 10 | GO:0030194 | 0.437 |
| negative regulation of catalytic activity | 79 | GO:0043086 | 0.437 |
| histone H2A acetylation | 12 | GO:0043968 | 0.437 |
| artery morphogenesis | 17 | GO:0048844 | 0.437 |
| establishment of planar polarity | 9 | GO:0001736 | 0.438 |
| embryonic heart tube development | 17 | GO:0035050 | 0.438 |
| regulation of protein homodimerization activity | 8 | GO:0043496 | 0.438 |
| regulation of protein phosphorylation | 29 | GO:0001932 | 0.439 |
| tongue morphogenesis | 6 | GO:0043587 | 0.439 |
| porphyrin biosynthetic process | 13 | GO:0006779 | 0.44 |
| erythrocyte differentiation | 32 | GO:0030218 | 0.44 |
| long-chain fatty acid transport | 11 | GO:0015909 | 0.441 |
| spleen development | 22 | GO:0048536 | 0.441 |
| protein heterotrimerization | 7 | GO:0070208 | 0.441 |
| killing of cells of other organism | 7 | GO:0031640 | 0.442 |
| positive regulation of protein serine/threonine kinase activity | 14 | GO:0071902 | 0.442 |
| bile acid and bile salt transport | 17 | GO:0015721 | 0.443 |
| bleb assembly | 5 | GO:0032060 | 0.443 |
| collagen metabolic process | 7 | GO:0032963 | 0.443 |
| response to retinoic acid | 62 | GO:0032526 | 0.444 |
| antigen receptor-mediated signaling pathway | 5 | GO:0050851 | 0.444 |
| pharyngeal system development | 5 | GO:0060037 | 0.444 |
| lysosome organization | 29 | GO:0007040 | 0.445 |
| RNA metabolic process | 154 | GO:0016070 | 0.445 |
| bone morphogenesis | 13 | GO:0060349 | 0.445 |
| response to acid | 21 | GO:0001101 | 0.446 |
| nitric oxide mediated signal transduction | 13 | GO:0007263 | 0.446 |
| magnesium ion transport | 10 | GO:0015693 | 0.447 |
| glycosaminoglycan metabolic process | 7 | GO:0030203 | 0.447 |
| neuron death | 6 | GO:0070997 | 0.448 |
| protein autoprocessing | 11 | GO:0016540 | 0.449 |
| positive regulation of reactive oxygen species metabolic process | 20 | GO:2000379 | 0.449 |
| protein targeting to mitochondrion | 29 | GO:0006626 | 0.45 |
| circadian regulation of gene expression | 8 | GO:0032922 | 0.45 |
| T cell differentiation in thymus | 29 | GO:0033077 | 0.45 |
| secretion | 8 | GO:0046903 | 0.45 |
| carbohydrate metabolic process | 301 | GO:0005975 | 0.451 |
| lipid glycosylation | 6 | GO:0030259 | 0.451 |
| negative regulation of synaptic transmission | 11 | GO:0050805 | 0.451 |
| cotranslational protein targeting to membrane | 9 | GO:0006613 | 0.452 |
| negative regulation of axon extension | 10 | GO:0030517 | 0.452 |
| positive regulation of epithelial cell proliferation involved in lung morphogenesis | 6 | GO:0060501 | 0.452 |
| DNA ligation | 8 | GO:0006266 | 0.453 |
| attachment of GPI anchor to protein | 7 | GO:0016255 | 0.453 |
| very long-chain fatty acid biosynthetic process | 8 | GO:0042761 | 0.453 |
| biosynthetic process | 49 | GO:0009058 | 0.454 |
| positive regulation of nitric-oxide synthase 2 biosynthetic process | 5 | GO:0051773 | 0.454 |
| cellular response to estradiol stimulus | 11 | GO:0071392 | 0.454 |
| DNA replication, synthesis of RNA primer | 5 | GO:0006269 | 0.455 |
| protein maturation by protein folding | 5 | GO:0022417 | 0.455 |
| neuron differentiation | 114 | GO:0030182 | 0.455 |
| activin receptor signaling pathway | 5 | GO:0032924 | 0.455 |
| positive regulation of protein dephosphorylation | 5 | GO:0035307 | 0.455 |
| response to methylmercury | 13 | GO:0051597 | 0.455 |
| UDP-N-acetylglucosamine metabolic process | 6 | GO:0006047 | 0.456 |
| regulation of actin polymerization or depolymerization | 10 | GO:0008064 | 0.456 |
| hindlimb morphogenesis | 12 | GO:0035137 | 0.456 |
| camera-type eye development | 48 | GO:0043010 | 0.456 |
| pattern specification process | 88 | GO:0007389 | 0.457 |
| fibroblast growth factor receptor signaling pathway | 54 | GO:0008543 | 0.457 |
| positive regulation of mitochondrial depolarization | 6 | GO:0051901 | 0.457 |
| copper ion transport | 14 | GO:0006825 | 0.458 |
| retinal metabolic process | 8 | GO:0042574 | 0.458 |
| regulation of GTPase activity | 14 | GO:0043087 | 0.459 |
| skeletal system morphogenesis | 39 | GO:0048705 | 0.459 |
| fear response | 7 | GO:0042596 | 0.46 |
| nucleosome positioning | 6 | GO:0016584 | 0.461 |
| positive regulation of neuron apoptosis | 40 | GO:0043525 | 0.461 |
| positive regulation of cell size | 6 | GO:0045793 | 0.461 |
| digestive system development | 6 | GO:0055123 | 0.461 |
| galactose catabolic process | 7 | GO:0019388 | 0.462 |
| positive regulation of protein secretion | 18 | GO:0050714 | 0.462 |
| cysteine metabolic process | 5 | GO:0006534 | 0.463 |
| dephosphorylation | 134 | GO:0016311 | 0.463 |
| peptidyl-lysine dimethylation | 5 | GO:0018027 | 0.463 |
| lens morphogenesis in camera-type eye | 14 | GO:0002089 | 0.464 |
| catecholamine metabolic process | 9 | GO:0006584 | 0.464 |
| negative regulation of proteolysis | 21 | GO:0045861 | 0.464 |
| thyroid hormone transport | 7 | GO:0070327 | 0.464 |
| cell fate specification | 18 | GO:0001708 | 0.465 |
| microtubule depolymerization | 6 | GO:0007019 | 0.465 |
| negative regulation of protein dephosphorylation | 5 | GO:0035308 | 0.465 |
| regulation of osteoclast differentiation | 9 | GO:0045670 | 0.465 |
| protein K48-linked ubiquitination | 32 | GO:0070936 | 0.466 |
| B cell lineage commitment | 5 | GO:0002326 | 0.467 |
| nitrogen compound metabolic process | 19 | GO:0006807 | 0.467 |
| stem cell differentiation | 12 | GO:0048863 | 0.467 |
| phospholipid homeostasis | 8 | GO:0055091 | 0.467 |
| positive regulation of cell cycle arrest | 7 | GO:0071158 | 0.467 |
| regulation of cell growth | 75 | GO:0001558 | 0.468 |
| negative regulation of NF-kappaB transcription factor activity | 39 | GO:0032088 | 0.468 |
| regulation of gene-specific transcription | 9 | GO:0032583 | 0.468 |
| cellular response to insulin stimulus | 75 | GO:0032869 | 0.468 |
| growth | 36 | GO:0040007 | 0.468 |
| heterophilic cell-cell adhesion | 26 | GO:0007157 | 0.47 |
| positive regulation of chondrocyte differentiation | 11 | GO:0032332 | 0.471 |
| 9-cis-retinoic acid biosynthetic process | 6 | GO:0042904 | 0.471 |
| negative regulation of androgen receptor signaling pathway | 8 | GO:0060766 | 0.471 |
| DNA packaging | 8 | GO:0006323 | 0.472 |
| response to unfolded protein | 56 | GO:0006986 | 0.472 |
| neuron cell-cell adhesion | 11 | GO:0007158 | 0.472 |
| mating behavior | 9 | GO:0007617 | 0.472 |
| negative regulation of mammary gland epithelial cell proliferation | 5 | GO:0033600 | 0.472 |
| amyloid precursor protein catabolic process | 8 | GO:0042987 | 0.472 |
| platelet degranulation | 77 | GO:0002576 | 0.473 |
| homeostatic process | 11 | GO:0042592 | 0.473 |
| positive regulation of cholesterol storage | 6 | GO:0010886 | 0.474 |
| positive regulation of glycolysis | 9 | GO:0045821 | 0.474 |
| G1 phase of mitotic cell cycle | 29 | GO:0000080 | 0.475 |
| regulation of alternative nuclear mRNA splicing, via spliceosome | 9 | GO:0000381 | 0.475 |
| fructose 6-phosphate metabolic process | 8 | GO:0006002 | 0.476 |
| positive regulation of systemic arterial blood pressure | 6 | GO:0003084 | 0.477 |
| activation of Rac GTPase activity | 7 | GO:0032863 | 0.477 |
| bone development | 19 | GO:0060348 | 0.477 |
| positive regulation of thymocyte apoptosis | 6 | GO:0070245 | 0.477 |
| glomerulus development | 5 | GO:0032835 | 0.478 |
| regulation of ARF protein signal transduction | 16 | GO:0032012 | 0.479 |
| regulation of protein transport | 8 | GO:0051223 | 0.479 |
| regulation of branching involved in prostate gland morphogenesis | 7 | GO:0060687 | 0.479 |
| fatty acid alpha-oxidation | 7 | GO:0001561 | 0.48 |
| optic nerve development | 5 | GO:0021554 | 0.48 |
| high-density lipoprotein particle remodeling | 14 | GO:0034375 | 0.48 |
| positive regulation of exocytosis | 8 | GO:0045921 | 0.48 |
| pseudouridine synthesis | 14 | GO:0001522 | 0.481 |
| 'de novo' posttranslational protein folding | 34 | GO:0051084 | 0.481 |
| positive regulation of neuroblast proliferation | 13 | GO:0002052 | 0.482 |
| peptide metabolic process | 8 | GO:0006518 | 0.482 |
| mannose metabolic process | 11 | GO:0006013 | 0.483 |
| lens fiber cell differentiation | 10 | GO:0070306 | 0.483 |
| cell volume homeostasis | 12 | GO:0006884 | 0.485 |
| Ras protein signal transduction | 72 | GO:0007265 | 0.485 |
| positive regulation of peptidase activity | 11 | GO:0010952 | 0.485 |
| positive regulation of epidermal growth factor receptor signaling pathway | 10 | GO:0045742 | 0.485 |
| regulation of smooth muscle cell proliferation | 5 | GO:0048660 | 0.485 |
| response to stress | 203 | GO:0006950 | 0.486 |
| locomotory behavior | 79 | GO:0007626 | 0.487 |
| pantothenate metabolic process | 11 | GO:0015939 | 0.487 |
| cellular response to fibroblast growth factor stimulus | 6 | GO:0044344 | 0.487 |
| negative regulation of JAK-STAT cascade | 8 | GO:0046426 | 0.487 |
| gonad development | 26 | GO:0008406 | 0.488 |
| muscle cell fate commitment | 5 | GO:0042693 | 0.488 |
| leukocyte activation | 5 | GO:0045321 | 0.488 |
| atrial cardiac muscle tissue morphogenesis | 6 | GO:0055009 | 0.488 |
| glycogen biosynthetic process | 18 | GO:0005978 | 0.489 |
| fatty acid biosynthetic process | 60 | GO:0006633 | 0.489 |
| suckling behavior | 11 | GO:0001967 | 0.49 |
| mitotic cell cycle G1/S transition checkpoint | 11 | GO:0031575 | 0.49 |
| regulation of MAP kinase activity | 6 | GO:0043405 | 0.49 |
| positive regulation of cell death | 18 | GO:0010942 | 0.491 |
| protein glycosylation | 67 | GO:0006486 | 0.493 |
| lipid metabolic process | 315 | GO:0006629 | 0.493 |
| lipopolysaccharide biosynthetic process | 8 | GO:0009103 | 0.493 |
| protein neddylation | 7 | GO:0045116 | 0.493 |
| dolichol-linked oligosaccharide biosynthetic process | 31 | GO:0006488 | 0.494 |
| regulation of neuron projection development | 9 | GO:0010975 | 0.494 |
| neuron recognition | 6 | GO:0008038 | 0.495 |
| positive regulation of integrin activation | 5 | GO:0033625 | 0.495 |
| methionine metabolic process | 5 | GO:0006555 | 0.496 |
| mitotic spindle organization | 18 | GO:0007052 | 0.496 |
| epidermal cell differentiation | 7 | GO:0009913 | 0.496 |
| protein sumoylation | 12 | GO:0016925 | 0.496 |
| DNA damage response, signal transduction by p53 class mediator resulting in induction of apoptosis | 20 | GO:0042771 | 0.496 |
| folic acid transport | 9 | GO:0015884 | 0.497 |
| sperm capacitation | 9 | GO:0048240 | 0.497 |
| high-density lipoprotein particle assembly | 5 | GO:0034380 | 0.498 |
| cellular component disassembly involved in apoptosis | 48 | GO:0006921 | 0.499 |
| neurotransmitter uptake | 7 | GO:0001504 | 0.5 |
| sister chromatid cohesion | 9 | GO:0007062 | 0.5 |
| cholesterol transport | 25 | GO:0030301 | 0.5 |
| cartilage development | 63 | GO:0051216 | 0.5 |
| tissue morphogenesis | 6 | GO:0048729 | 0.502 |
| cardiac left ventricle morphogenesis | 5 | GO:0003214 | 0.503 |
| cellular aldehyde metabolic process | 12 | GO:0006081 | 0.504 |
| negative regulation of retinoic acid receptor signaling pathway | 7 | GO:0048387 | 0.504 |
| homotypic cell-cell adhesion | 5 | GO:0034109 | 0.506 |
| post-embryonic camera-type eye development | 5 | GO:0031077 | 0.507 |
| positive regulation of fatty acid oxidation | 5 | GO:0046321 | 0.51 |
| regulation of circadian rhythm | 9 | GO:0042752 | 0.511 |
| neurotransmitter catabolic process | 10 | GO:0042135 | 0.514 |
| negative regulation of neuroblast proliferation | 7 | GO:0007406 | 0.515 |
| cellular response to X-ray | 5 | GO:0071481 | 0.515 |
| negative regulation of epidermal growth factor receptor activity | 5 | GO:0007175 | 0.519 |
| embryonic skeletal system morphogenesis | 50 | GO:0048704 | 0.52 |
| ventricular cardiac muscle tissue morphogenesis | 26 | GO:0055010 | 0.52 |
|  |  |  |  |
| **Molecular function** |  |  |  |
| proline-rich region binding | 16 | GO:0070064 | 0.008 |
| positive transcription elongation factor activity | 7 | GO:0008159 | 0.01 |
| acid-amino acid ligase activity | 79 | GO:0016881 | 0.01 |
| histone acetyltransferase binding | 15 | GO:0035035 | 0.011 |
| growth factor binding | 39 | GO:0019838 | 0.012 |
| SMAD binding | 37 | GO:0046332 | 0.012 |
| phosphoprotein binding | 37 | GO:0051219 | 0.012 |
| transmembrane receptor protein tyrosine kinase activity | 54 | GO:0004714 | 0.013 |
| transforming growth factor beta receptor activity | 18 | GO:0005024 | 0.013 |
| two-component sensor activity | 14 | GO:0000155 | 0.014 |
| semaphorin receptor binding | 6 | GO:0030215 | 0.015 |
| transforming growth factor beta binding | 12 | GO:0050431 | 0.018 |
| receptor signaling protein serine/threonine kinase activity | 11 | GO:0004702 | 0.02 |
| MAP kinase kinase kinase activity | 21 | GO:0004709 | 0.02 |
| phospholipase binding | 14 | GO:0043274 | 0.02 |
| protein kinase regulator activity | 6 | GO:0019887 | 0.022 |
| histone acetyltransferase activity | 39 | GO:0004402 | 0.024 |
| protein histidine kinase activity | 6 | GO:0004673 | 0.027 |
| cadherin binding | 22 | GO:0045296 | 0.028 |
| acyl-CoA dehydrogenase activity | 14 | GO:0003995 | 0.03 |
| nucleocytoplasmic transporter activity | 12 | GO:0005487 | 0.03 |
| myosin heavy chain binding | 5 | GO:0032036 | 0.033 |
| laminin-1 binding | 8 | GO:0043237 | 0.033 |
| ion channel binding | 6 | GO:0044325 | 0.033 |
| ribosomal protein S6 kinase activity | 5 | GO:0004711 | 0.035 |
| protein tyrosine kinase activity | 125 | GO:0004713 | 0.035 |
| acyltransferase activity | 144 | GO:0008415 | 0.035 |
| ligand-regulated transcription factor activity | 11 | GO:0003706 | 0.036 |
| nuclease activity | 97 | GO:0004518 | 0.036 |
| protein phosphatase inhibitor activity | 25 | GO:0004864 | 0.037 |
| transferase activity, transferring phosphorus-containing groups | 494 | GO:0016772 | 0.037 |
| peptide receptor activity, G-protein coupled | 9 | GO:0008528 | 0.039 |
| DNA N-glycosylase activity | 9 | GO:0019104 | 0.04 |
| protein kinase activity | 500 | GO:0004672 | 0.042 |
| enoyl-CoA hydratase activity | 6 | GO:0004300 | 0.043 |
| cAMP-dependent protein kinase inhibitor activity | 5 | GO:0004862 | 0.043 |
| solute:hydrogen antiporter activity | 10 | GO:0015299 | 0.043 |
| xenobiotic-transporting ATPase activity | 5 | GO:0008559 | 0.044 |
| palmitoyltransferase activity | 10 | GO:0016409 | 0.045 |
| hormone binding | 22 | GO:0042562 | 0.045 |
| phosphorus-oxygen lyase activity | 17 | GO:0016849 | 0.046 |
| guanyl-nucleotide exchange factor activity | 142 | GO:0005085 | 0.048 |
| protein phosphatase 1 binding | 8 | GO:0008157 | 0.048 |
| enzyme regulator activity | 17 | GO:0030234 | 0.048 |
| receptor signaling protein tyrosine kinase activity | 13 | GO:0004716 | 0.049 |
| vascular endothelial growth factor receptor activity | 10 | GO:0005021 | 0.049 |
| armadillo repeat domain binding | 10 | GO:0070016 | 0.049 |
| actin binding | 307 | GO:0003779 | 0.05 |
| platelet-derived growth factor receptor binding | 16 | GO:0005161 | 0.05 |
| ErbB-3 class receptor binding | 5 | GO:0043125 | 0.05 |
| ubiquitin binding | 29 | GO:0043130 | 0.05 |
| SH3 domain binding | 123 | GO:0017124 | 0.051 |
| 1-acylglycerol-3-phosphate O-acyltransferase activity | 9 | GO:0003841 | 0.052 |
| structural constituent of muscle | 44 | GO:0008307 | 0.054 |
| phosphatidylinositol 3-kinase binding | 23 | GO:0043548 | 0.055 |
| ubiquitin-protein ligase activity | 239 | GO:0004842 | 0.056 |
| guanylate cyclase activity | 14 | GO:0004383 | 0.057 |
| TBP-class protein binding | 13 | GO:0017025 | 0.058 |
| phosphatidylinositol-3-phosphatase activity | 5 | GO:0004438 | 0.059 |
| peptide-methionine-(S)-S-oxide reductase activity | 6 | GO:0008113 | 0.059 |
| Rab GTPase binding | 39 | GO:0017137 | 0.06 |
| beta-2 adrenergic receptor binding | 5 | GO:0031698 | 0.06 |
| NADPH binding | 8 | GO:0070402 | 0.06 |
| GTPase activator activity | 204 | GO:0005096 | 0.061 |
| kinase activity | 660 | GO:0016301 | 0.061 |
| inositol or phosphatidylinositol phosphatase activity | 22 | GO:0004437 | 0.062 |
| fatty-acyl-CoA binding | 18 | GO:0000062 | 0.063 |
| oxidoreductase activity, acting on the CH-CH group of donors | 21 | GO:0016627 | 0.063 |
| ADP-ribosylation factor binding | 6 | GO:0030306 | 0.065 |
| ribosome binding | 25 | GO:0043022 | 0.066 |
| protein serine/threonine kinase activity | 390 | GO:0004674 | 0.067 |
| myosin binding | 21 | GO:0017022 | 0.067 |
| cytochrome-b5 reductase activity | 8 | GO:0004128 | 0.068 |
| RNA polymerase II transcription elongation factor activity | 9 | GO:0016944 | 0.068 |
| protein-lysine N-methyltransferase activity | 6 | GO:0016279 | 0.069 |
| phosphatase binding | 12 | GO:0019902 | 0.069 |
| ADP binding | 30 | GO:0043531 | 0.069 |
| inositol-polyphosphate 5-phosphatase activity | 10 | GO:0004445 | 0.07 |
| MAP kinase kinase activity | 15 | GO:0004708 | 0.07 |
| transforming growth factor beta receptor, pathway-specific cytoplasmic mediator activity | 5 | GO:0030618 | 0.07 |
| NAD-dependent histone deacetylase activity (H3-K9 specific) | 14 | GO:0046969 | 0.07 |
| tubulin binding | 29 | GO:0015631 | 0.071 |
| peptidyl-prolyl cis-trans isomerase activity | 35 | GO:0003755 | 0.072 |
| microtubule-severing ATPase activity | 5 | GO:0008568 | 0.072 |
| receptor tyrosine kinase binding | 21 | GO:0030971 | 0.072 |
| dynactin binding | 5 | GO:0034452 | 0.072 |
| RNA helicase activity | 13 | GO:0003724 | 0.073 |
| small GTPase binding | 11 | GO:0031267 | 0.073 |
| 3-hydroxyacyl-CoA dehydrogenase activity | 8 | GO:0003857 | 0.075 |
| fibroblast growth factor binding | 13 | GO:0017134 | 0.075 |
| ubiquitin-ubiquitin ligase activity | 9 | GO:0034450 | 0.075 |
| four-way junction DNA binding | 6 | GO:0000400 | 0.076 |
| Rho guanyl-nucleotide exchange factor activity | 72 | GO:0005089 | 0.076 |
| histone deacetylase activity | 14 | GO:0004407 | 0.077 |
| exonuclease activity | 51 | GO:0004527 | 0.077 |
| Rho GTPase activator activity | 22 | GO:0005100 | 0.077 |
| bubble DNA binding | 6 | GO:0000405 | 0.079 |
| nucleosome binding | 5 | GO:0031491 | 0.079 |
| histone acetyl-lysine binding | 8 | GO:0070577 | 0.079 |
| thyroid hormone receptor activity | 8 | GO:0004887 | 0.08 |
| dipeptidase activity | 12 | GO:0016805 | 0.083 |
| ubiquitin thiolesterase activity | 60 | GO:0004221 | 0.084 |
| store-operated calcium channel activity | 7 | GO:0015279 | 0.084 |
| small GTPase regulator activity | 31 | GO:0005083 | 0.085 |
| histone deacetylase activity (H3-K14 specific) | 11 | GO:0031078 | 0.085 |
| histone deacetylase activity (H3-K9 specific) | 11 | GO:0032129 | 0.085 |
| nuclear hormone receptor binding | 9 | GO:0035257 | 0.086 |
| vascular endothelial growth factor receptor binding | 7 | GO:0005172 | 0.087 |
| transcription elongation regulator activity | 13 | GO:0003711 | 0.089 |
| phosphorylase kinase activity | 5 | GO:0004689 | 0.09 |
| integrin binding | 90 | GO:0005178 | 0.092 |
| ligase activity | 347 | GO:0016874 | 0.092 |
| microtubule plus-end binding | 12 | GO:0051010 | 0.092 |
| RAGE receptor binding | 7 | GO:0050786 | 0.093 |
| transmembrane receptor protein serine/threonine kinase activity | 14 | GO:0004675 | 0.094 |
| MAP kinase activity | 15 | GO:0004707 | 0.094 |
| sterol binding | 6 | GO:0032934 | 0.094 |
| mitogen-activated protein kinase binding | 10 | GO:0051019 | 0.094 |
| 3'-5'-exoribonuclease activity | 12 | GO:0000175 | 0.095 |
| ubiquitin protein ligase binding | 89 | GO:0031625 | 0.095 |
| 14-3-3 protein binding | 5 | GO:0071889 | 0.095 |
| microtubule binding | 87 | GO:0008017 | 0.096 |
| protein kinase A catalytic subunit binding | 8 | GO:0034236 | 0.096 |
| ATP-dependent peptidase activity | 8 | GO:0004176 | 0.097 |
| frizzled-2 binding | 6 | GO:0005110 | 0.097 |
| GTP-dependent protein binding | 12 | GO:0030742 | 0.098 |
| heat shock protein binding | 68 | GO:0031072 | 0.098 |
| NAD-dependent histone deacetylase activity (H3-K14 specific) | 12 | GO:0032041 | 0.1 |
| histone deacetylase activity (H3-K16 specific) | 12 | GO:0034739 | 0.1 |
| NAD-dependent histone deacetylase activity (H4-K16 specific) | 12 | GO:0046970 | 0.1 |
| Notch binding | 9 | GO:0005112 | 0.101 |
| single-stranded DNA specific endodeoxyribonuclease activity | 6 | GO:0000014 | 0.102 |
| cyclin-dependent protein kinase activity | 28 | GO:0004693 | 0.102 |
| JUN kinase kinase kinase activity | 5 | GO:0004706 | 0.103 |
| sodium:hydrogen antiporter activity | 8 | GO:0015385 | 0.103 |
| 1-phosphatidylinositol-4-phosphate 5-kinase activity | 8 | GO:0016308 | 0.105 |
| Rac GTPase binding | 17 | GO:0048365 | 0.108 |
| clathrin binding | 11 | GO:0030276 | 0.109 |
| protein complex scaffold | 21 | GO:0032947 | 0.112 |
| NAD binding | 45 | GO:0051287 | 0.112 |
| sodium channel regulator activity | 7 | GO:0017080 | 0.114 |
| Rac GTPase activator activity | 15 | GO:0030675 | 0.114 |
| galactosyltransferase activity | 24 | GO:0008378 | 0.115 |
| Ran guanyl-nucleotide exchange factor activity | 6 | GO:0005087 | 0.116 |
| protein kinase activator activity | 12 | GO:0030295 | 0.116 |
| vitamin D receptor binding | 18 | GO:0042809 | 0.116 |
| chromo shadow domain binding | 6 | GO:0070087 | 0.116 |
| inositol trisphosphate 3-kinase activity | 7 | GO:0008440 | 0.117 |
| GTPase binding | 23 | GO:0051020 | 0.117 |
| aminomethyltransferase activity | 5 | GO:0004047 | 0.118 |
| insulin receptor binding | 32 | GO:0005158 | 0.118 |
| protein phosphatase type 2A regulator activity | 19 | GO:0008601 | 0.118 |
| polyubiquitin binding | 13 | GO:0031593 | 0.118 |
| 3'-5' exonuclease activity | 15 | GO:0008408 | 0.12 |
| sulfotransferase activity | 44 | GO:0008146 | 0.121 |
| vinculin binding | 10 | GO:0017166 | 0.123 |
| chemorepellent activity | 5 | GO:0045499 | 0.123 |
| RS domain binding | 7 | GO:0050733 | 0.124 |
| beta-catenin binding | 57 | GO:0008013 | 0.126 |
| transcription factor binding | 250 | GO:0008134 | 0.126 |
| metalloendopeptidase inhibitor activity | 10 | GO:0008191 | 0.126 |
| helicase activity | 132 | GO:0004386 | 0.127 |
| insulin binding | 6 | GO:0043559 | 0.127 |
| transcription repressor activity | 258 | GO:0016564 | 0.129 |
| polynucleotide adenylyltransferase activity | 6 | GO:0004652 | 0.13 |
| protein phosphatase binding | 45 | GO:0019903 | 0.13 |
| translation initiation factor binding | 7 | GO:0031369 | 0.13 |
| type I transforming growth factor beta receptor binding | 7 | GO:0034713 | 0.13 |
| LIM domain binding | 8 | GO:0030274 | 0.131 |
| ligand-dependent nuclear receptor transcription coactivator activity | 36 | GO:0030374 | 0.132 |
| primary amine oxidase activity | 6 | GO:0008131 | 0.133 |
| retinol binding | 12 | GO:0019841 | 0.133 |
| mitogen-activated protein kinase kinase kinase binding | 19 | GO:0031435 | 0.133 |
| phosphatidylinositol phosphate kinase activity | 9 | GO:0016307 | 0.134 |
| exoribonuclease activity | 11 | GO:0004532 | 0.135 |
| transferase activity, transferring glycosyl groups | 194 | GO:0016757 | 0.135 |
| histone methyltransferase activity (H3-K4 specific) | 12 | GO:0042800 | 0.136 |
| RNA polymerase II transcription factor activity | 143 | GO:0003702 | 0.138 |
| protein methyltransferase activity | 15 | GO:0008276 | 0.138 |
| hydrolase activity, acting on acid anhydrides | 20 | GO:0016817 | 0.138 |
| transcription corepressor activity | 160 | GO:0003714 | 0.139 |
| mannosyltransferase activity | 8 | GO:0000030 | 0.143 |
| insulin receptor substrate binding | 13 | GO:0043560 | 0.143 |
| ATP-dependent helicase activity | 80 | GO:0008026 | 0.144 |
| cysteine-type peptidase activity | 112 | GO:0008234 | 0.144 |
| sulfate transmembrane transporter activity | 6 | GO:0015116 | 0.145 |
| translation repressor activity | 7 | GO:0030371 | 0.145 |
| methyltransferase activity | 148 | GO:0008168 | 0.146 |
| SUMO ligase activity | 9 | GO:0019789 | 0.146 |
| cobalamin binding | 8 | GO:0031419 | 0.146 |
| transforming growth factor beta receptor binding | 15 | GO:0005160 | 0.147 |
| calcium ion transmembrane transporter activity | 6 | GO:0015085 | 0.147 |
| I-SMAD binding | 11 | GO:0070411 | 0.147 |
| repressing transcription factor binding | 18 | GO:0070491 | 0.147 |
| glycosaminoglycan binding | 23 | GO:0005539 | 0.149 |
| protein transporter activity | 73 | GO:0008565 | 0.149 |
| acetyltransferase activity | 9 | GO:0016407 | 0.15 |
| activin binding | 13 | GO:0048185 | 0.15 |
| 5'-3' exonuclease activity | 8 | GO:0008409 | 0.151 |
| hyalurononglucosaminidase activity | 6 | GO:0004415 | 0.152 |
| transcription coactivator activity | 216 | GO:0003713 | 0.153 |
| frizzled binding | 22 | GO:0005109 | 0.153 |
| lamin binding | 9 | GO:0005521 | 0.153 |
| translation repressor activity, nucleic acid binding | 7 | GO:0000900 | 0.154 |
| diacylglycerol kinase activity | 13 | GO:0004143 | 0.155 |
| collagen binding | 49 | GO:0005518 | 0.157 |
| protein C-terminus binding | 187 | GO:0008022 | 0.158 |
| protein deacetylase activity | 8 | GO:0033558 | 0.158 |
| outward rectifier potassium channel activity | 6 | GO:0015271 | 0.161 |
| retinoic acid receptor activity | 5 | GO:0003708 | 0.162 |
| ephrin receptor activity | 14 | GO:0005003 | 0.162 |
| sulfuric ester hydrolase activity | 13 | GO:0008484 | 0.162 |
| actin filament binding | 72 | GO:0051015 | 0.162 |
| ligand-dependent nuclear receptor activity | 47 | GO:0004879 | 0.163 |
| RPTP-like protein binding | 6 | GO:0042153 | 0.163 |
| Rho GTPase binding | 30 | GO:0017048 | 0.165 |
| gamma-catenin binding | 12 | GO:0045295 | 0.165 |
| quinone binding | 11 | GO:0048038 | 0.166 |
| actin-dependent ATPase activity | 11 | GO:0030898 | 0.167 |
| general RNA polymerase II transcription factor activity | 24 | GO:0016251 | 0.168 |
| mitogen-activated protein kinase kinase binding | 12 | GO:0031434 | 0.168 |
| leucine zipper domain binding | 5 | GO:0043522 | 0.168 |
| insulin-like growth factor I binding | 7 | GO:0031994 | 0.169 |
| NAD+ binding | 12 | GO:0070403 | 0.169 |
| alpha-actinin binding | 5 | GO:0051393 | 0.171 |
| benzodiazepine receptor activity | 7 | GO:0008503 | 0.172 |
| hydrolase activity, hydrolyzing N-glycosyl compounds | 7 | GO:0016799 | 0.175 |
| Ras GTPase binding | 15 | GO:0017016 | 0.178 |
| phospholipase C activity | 22 | GO:0004629 | 0.179 |
| enzyme binding | 199 | GO:0019899 | 0.181 |
| type II transforming growth factor beta receptor binding | 7 | GO:0005114 | 0.182 |
| calcium-transporting ATPase activity | 9 | GO:0005388 | 0.182 |
| dynein binding | 8 | GO:0045502 | 0.182 |
| tau protein binding | 8 | GO:0048156 | 0.182 |
| RNA polymerase II carboxy-terminal domain kinase activity | 15 | GO:0008353 | 0.184 |
| G-protein coupled receptor kinase activity | 7 | GO:0004703 | 0.186 |
| phosphatidylinositol N-acetylglucosaminyltransferase activity | 5 | GO:0017176 | 0.187 |
| coenzyme binding | 26 | GO:0050662 | 0.188 |
| glycolipid binding | 7 | GO:0051861 | 0.188 |
| protein tyrosine/serine/threonine phosphatase activity | 39 | GO:0008138 | 0.189 |
| GTP-Rho binding | 9 | GO:0017049 | 0.189 |
| snoRNA binding | 9 | GO:0030515 | 0.189 |
| RNA polymerase III transcription factor activity | 11 | GO:0003709 | 0.191 |
| ubiquitin-specific protease activity | 38 | GO:0004843 | 0.191 |
| tropomyosin binding | 14 | GO:0005523 | 0.191 |
| ligase activity, forming aminoacyl-tRNA and related compounds | 8 | GO:0016876 | 0.191 |
| syntaxin-1 binding | 16 | GO:0017075 | 0.191 |
| histone deacetylase binding | 52 | GO:0042826 | 0.191 |
| fibroblast growth factor receptor binding | 17 | GO:0005104 | 0.194 |
| unmethylated CpG binding | 7 | GO:0045322 | 0.194 |
| tRNA binding | 25 | GO:0000049 | 0.196 |
| SNARE binding | 19 | GO:0000149 | 0.198 |
| histone-lysine N-methyltransferase activity | 28 | GO:0018024 | 0.198 |
| S100 alpha binding | 5 | GO:0048155 | 0.2 |
| transcription initiation factor activity | 12 | GO:0016986 | 0.202 |
| 1-phosphatidylinositol binding | 22 | GO:0005545 | 0.203 |
| axon guidance receptor activity | 7 | GO:0008046 | 0.203 |
| ATPase activator activity | 9 | GO:0001671 | 0.204 |
| phosphoprotein phosphatase activity | 136 | GO:0004721 | 0.204 |
| biotin carboxylase activity | 6 | GO:0004075 | 0.205 |
| snRNA binding | 6 | GO:0017069 | 0.207 |
| 3',5'-cyclic-AMP phosphodiesterase activity | 16 | GO:0004115 | 0.211 |
| Rab guanyl-nucleotide exchange factor activity | 5 | GO:0017112 | 0.211 |
| alpha-catenin binding | 7 | GO:0045294 | 0.211 |
| N-acetyltransferase activity | 27 | GO:0008080 | 0.213 |
| 1-aminocyclopropane-1-carboxylate synthase activity | 5 | GO:0016847 | 0.213 |
| co-SMAD binding | 9 | GO:0070410 | 0.213 |
| p53 binding | 38 | GO:0002039 | 0.217 |
| DNA helicase activity | 18 | GO:0003678 | 0.217 |
| endonuclease activity | 71 | GO:0004519 | 0.219 |
| oxidoreductase activity, acting on NADH or NADPH | 12 | GO:0016651 | 0.219 |
| glycoprotein binding | 47 | GO:0001948 | 0.22 |
| fucosyltransferase activity | 11 | GO:0008417 | 0.22 |
| carboxylesterase activity | 35 | GO:0004091 | 0.221 |
| L-ascorbic acid binding | 21 | GO:0031418 | 0.221 |
| fructose-2,6-bisphosphate 2-phosphatase activity | 8 | GO:0004331 | 0.222 |
| 3',5'-cyclic-nucleotide phosphodiesterase activity | 23 | GO:0004114 | 0.223 |
| 7S RNA binding | 6 | GO:0008312 | 0.223 |
| lipoprotein particle binding | 6 | GO:0071813 | 0.224 |
| protein serine/threonine kinase activator activity | 7 | GO:0043539 | 0.226 |
| platelet-derived growth factor binding | 12 | GO:0048407 | 0.226 |
| translation initiation factor activity | 58 | GO:0003743 | 0.227 |
| steroid hormone receptor activity | 49 | GO:0003707 | 0.231 |
| RNA binding | 656 | GO:0003723 | 0.232 |
| secondary active sulfate transmembrane transporter activity | 9 | GO:0008271 | 0.233 |
| cAMP binding | 18 | GO:0030552 | 0.233 |
| guanylate kinase activity | 14 | GO:0004385 | 0.235 |
| acetylglucosaminyltransferase activity | 14 | GO:0008375 | 0.235 |
| extracellular matrix structural constituent | 68 | GO:0005201 | 0.238 |
| protein N-terminus binding | 90 | GO:0047485 | 0.239 |
| ubiquinol-cytochrome-c reductase activity | 7 | GO:0008121 | 0.24 |
| fatty acid elongase activity | 6 | GO:0009922 | 0.24 |
| phosphatidylserine binding | 11 | GO:0001786 | 0.241 |
| protein kinase C activity | 15 | GO:0004697 | 0.241 |
| arylsulfatase activity | 11 | GO:0004065 | 0.242 |
| LRR domain binding | 6 | GO:0030275 | 0.244 |
| thyroid hormone receptor binding | 29 | GO:0046966 | 0.246 |
| ATPase activity, coupled | 11 | GO:0042623 | 0.247 |
| opioid peptide activity | 5 | GO:0001515 | 0.248 |
| biotin binding | 6 | GO:0009374 | 0.248 |
| chaperone binding | 43 | GO:0051087 | 0.249 |
| lipid binding | 177 | GO:0008289 | 0.251 |
| bile acid:sodium symporter activity | 5 | GO:0008508 | 0.252 |
| cation transmembrane transporter activity | 20 | GO:0008324 | 0.253 |
| rRNA methyltransferase activity | 5 | GO:0008649 | 0.255 |
| nucleotidyltransferase activity | 76 | GO:0016779 | 0.257 |
| Wnt-protein binding | 25 | GO:0017147 | 0.257 |
| fibroblast growth factor receptor activity | 5 | GO:0005007 | 0.258 |
| JUN kinase binding | 9 | GO:0008432 | 0.258 |
| thiamine pyrophosphate binding | 6 | GO:0030976 | 0.258 |
| phosphatidylinositol-3,4,5-trisphosphate binding | 18 | GO:0005547 | 0.259 |
| intermediate filament binding | 6 | GO:0019215 | 0.259 |
| toxin binding | 9 | GO:0015643 | 0.26 |
| hydrolase activity, acting on carbon-nitrogen (but not peptide) bonds, in linear amides | 16 | GO:0016811 | 0.26 |
| kinesin binding | 19 | GO:0019894 | 0.26 |
| Rab GTPase activator activity | 50 | GO:0005097 | 0.262 |
| DNA-directed DNA polymerase activity | 29 | GO:0003887 | 0.265 |
| protein serine/threonine phosphatase activity | 41 | GO:0004722 | 0.267 |
| kinase activator activity | 6 | GO:0019209 | 0.267 |
| ATPase activity, coupled to transmembrane movement of substances | 35 | GO:0042626 | 0.267 |
| hydrolase activity, acting on acid anhydrides, in phosphorus-containing anhydrides | 19 | GO:0016818 | 0.268 |
| chromatin binding | 225 | GO:0003682 | 0.269 |
| aldehyde dehydrogenase (NAD) activity | 9 | GO:0004029 | 0.27 |
| aspartic-type endopeptidase activity | 21 | GO:0004190 | 0.27 |
| ARF GTPase activator activity | 25 | GO:0008060 | 0.27 |
| nucleobase, nucleoside, nucleotide kinase activity | 9 | GO:0019205 | 0.271 |
| phosphoric ester hydrolase activity | 8 | GO:0042578 | 0.271 |
| methylated histone residue binding | 28 | GO:0035064 | 0.272 |
| bHLH transcription factor binding | 11 | GO:0043425 | 0.272 |
| fatty acid transporter activity | 5 | GO:0015245 | 0.276 |
| hydrolase activity, acting on carbon-nitrogen (but not peptide) bonds, in cyclic amides | 8 | GO:0016812 | 0.276 |
| Ras guanyl-nucleotide exchange factor activity | 11 | GO:0005088 | 0.277 |
| translation elongation factor activity | 30 | GO:0003746 | 0.279 |
| metallocarboxypeptidase activity | 25 | GO:0004181 | 0.279 |
| phosphatidate phosphatase activity | 11 | GO:0008195 | 0.281 |
| low-density lipoprotein particle binding | 15 | GO:0030169 | 0.283 |
| protein phosphatase 2A binding | 16 | GO:0051721 | 0.285 |
| cytoskeletal protein binding | 50 | GO:0008092 | 0.287 |
| ephrin receptor binding | 13 | GO:0046875 | 0.29 |
| low-density lipoprotein particle receptor binding | 15 | GO:0050750 | 0.29 |
| rRNA binding | 25 | GO:0019843 | 0.291 |
| thioesterase binding | 5 | GO:0031996 | 0.291 |
| oxidoreductase activity, acting on the CH-NH2 group of donors, oxygen as acceptor | 7 | GO:0016641 | 0.292 |
| protein kinase A binding | 13 | GO:0051018 | 0.292 |
| syntaxin binding | 26 | GO:0019905 | 0.293 |
| phosphotransferase activity, for other substituted phosphate groups | 10 | GO:0016780 | 0.296 |
| AMP-activated protein kinase activity | 7 | GO:0004679 | 0.297 |
| ARF guanyl-nucleotide exchange factor activity | 18 | GO:0005086 | 0.297 |
| insulin-like growth factor II binding | 5 | GO:0031995 | 0.3 |
| RNA methyltransferase activity | 6 | GO:0008173 | 0.301 |
| troponin I binding | 5 | GO:0031013 | 0.301 |
| FK506 binding | 16 | GO:0005528 | 0.302 |
| insulin-like growth factor binding | 23 | GO:0005520 | 0.303 |
| ribonuclease P activity | 9 | GO:0004526 | 0.304 |
| retinoid X receptor binding | 14 | GO:0046965 | 0.304 |
| N-acetylglucosamine 6-O-sulfotransferase activity | 5 | GO:0001517 | 0.307 |
| acetylcholine receptor binding | 6 | GO:0033130 | 0.309 |
| inositol hexakisphosphate 5-kinase activity | 5 | GO:0000832 | 0.313 |
| calcium-dependent cysteine-type endopeptidase activity | 16 | GO:0004198 | 0.313 |
| sugar:hydrogen symporter activity | 10 | GO:0005351 | 0.313 |
| flavin adenine dinucleotide binding | 76 | GO:0050660 | 0.313 |
| DNA-directed RNA polymerase activity | 35 | GO:0003899 | 0.314 |
| extracellular matrix binding | 20 | GO:0050840 | 0.314 |
| NAD+ ADP-ribosyltransferase activity | 26 | GO:0003950 | 0.316 |
| protein domain specific binding | 212 | GO:0019904 | 0.316 |
| 1-alkyl-2-acetylglycerophosphocholine esterase activity | 5 | GO:0003847 | 0.318 |
| chloride transmembrane transporter activity | 5 | GO:0015108 | 0.319 |
| MutLalpha complex binding | 6 | GO:0032405 | 0.319 |
| calcium-activated potassium channel activity | 14 | GO:0015269 | 0.321 |
| cytoskeletal adaptor activity | 20 | GO:0008093 | 0.325 |
| RNA polymerase II transcription factor binding | 7 | GO:0001085 | 0.327 |
| phosphatidylinositol-4,5-bisphosphate 5-phosphatase activity | 7 | GO:0004439 | 0.328 |
| O-acyltransferase activity | 5 | GO:0008374 | 0.328 |
| semaphorin receptor activity | 7 | GO:0017154 | 0.329 |
| oxidoreductase activity, acting on paired donors, with incorporation or reduction of molecular oxygen, 2-oxoglutarate as one donor, and incorporation of one atom each of oxygen into both donors | 7 | GO:0016706 | 0.331 |
| mRNA binding | 58 | GO:0003729 | 0.332 |
| adenosine receptor activity, G-protein coupled | 6 | GO:0001609 | 0.333 |
| endoribonuclease activity | 6 | GO:0004521 | 0.335 |
| telomeric DNA binding | 11 | GO:0042162 | 0.336 |
| protein kinase inhibitor activity | 24 | GO:0004860 | 0.338 |
| microfilament motor activity | 18 | GO:0000146 | 0.339 |
| acylglycerol lipase activity | 9 | GO:0047372 | 0.34 |
| alpha-N-acetylneuraminate alpha-2,8-sialyltransferase activity | 5 | GO:0003828 | 0.342 |
| calcium channel regulator activity | 21 | GO:0005246 | 0.344 |
| pyrophosphatase activity | 5 | GO:0016462 | 0.344 |
| transferase activity, transferring nitrogenous groups | 16 | GO:0016769 | 0.344 |
| NADP binding | 38 | GO:0050661 | 0.344 |
| phosphate binding | 14 | GO:0042301 | 0.345 |
| nucleoside diphosphate kinase activity | 11 | GO:0004550 | 0.346 |
| glutamate binding | 11 | GO:0016595 | 0.348 |
| potassium channel inhibitor activity | 6 | GO:0019870 | 0.348 |
| estrogen receptor binding | 26 | GO:0030331 | 0.351 |
| E-box binding | 15 | GO:0070888 | 0.352 |
| phosphatase inhibitor activity | 5 | GO:0019212 | 0.355 |
| phosphatidylinositol-4,5-bisphosphate binding | 22 | GO:0005546 | 0.356 |
| hydrolase activity, acting on carbon-nitrogen (but not peptide) bonds | 17 | GO:0016810 | 0.356 |
| phosphatidylinositol-3,5-bisphosphate binding | 11 | GO:0080025 | 0.356 |
| AMP binding | 13 | GO:0016208 | 0.357 |
| fructose-6-phosphate binding | 5 | GO:0070095 | 0.357 |
| carboxypeptidase activity | 39 | GO:0004180 | 0.359 |
| Wnt receptor activity | 18 | GO:0042813 | 0.359 |
| glycerophosphodiester phosphodiesterase activity | 7 | GO:0008889 | 0.36 |
| MAP-kinase scaffold activity | 5 | GO:0005078 | 0.364 |
| histone demethylase activity (H3-K9 specific) | 6 | GO:0032454 | 0.365 |
| delayed rectifier potassium channel activity | 12 | GO:0005251 | 0.367 |
| histone binding | 57 | GO:0042393 | 0.367 |
| neurotransmitter transporter activity | 6 | GO:0005326 | 0.368 |
| histone methyltransferase activity (H3-K9 specific) | 5 | GO:0046974 | 0.368 |
| transmembrane-ephrin receptor activity | 8 | GO:0005005 | 0.369 |
| double-stranded telomeric DNA binding | 5 | GO:0003691 | 0.371 |
| nucleotide kinase activity | 5 | GO:0019201 | 0.371 |
| substrate-specific transmembrane transporter activity | 13 | GO:0022891 | 0.371 |
| SH3/SH2 adaptor activity | 50 | GO:0005070 | 0.372 |
| cAMP-dependent protein kinase regulator activity | 17 | GO:0008603 | 0.375 |
| small protein activating enzyme activity | 5 | GO:0008641 | 0.376 |
| enzyme inhibitor activity | 37 | GO:0004857 | 0.378 |
| calcium:sodium antiporter activity | 5 | GO:0005432 | 0.378 |
| oxidoreductase activity, acting on the aldehyde or oxo group of donors, disulfide as acceptor | 5 | GO:0016624 | 0.379 |
| aminoacyl-tRNA ligase activity | 46 | GO:0004812 | 0.381 |
| nucleoside-triphosphatase activity | 118 | GO:0017111 | 0.383 |
| protein kinase binding | 209 | GO:0019901 | 0.384 |
| calcium-release channel activity | 5 | GO:0015278 | 0.385 |
| nitric-oxide synthase regulator activity | 6 | GO:0030235 | 0.388 |
| GTP binding | 355 | GO:0005525 | 0.389 |
| phosphoric diester hydrolase activity | 51 | GO:0008081 | 0.39 |
| glycosphingolipid binding | 7 | GO:0043208 | 0.39 |
| motor activity | 111 | GO:0003774 | 0.391 |
| polysaccharide binding | 12 | GO:0030247 | 0.392 |
| neurexin binding | 10 | GO:0042043 | 0.392 |
| misfolded protein binding | 11 | GO:0051787 | 0.392 |
| SNAP receptor activity | 20 | GO:0005484 | 0.395 |
| folic acid transporter activity | 8 | GO:0008517 | 0.395 |
| Hsp90 protein binding | 14 | GO:0051879 | 0.397 |
| structural constituent of ribosome | 149 | GO:0003735 | 0.399 |
| receptor signaling complex scaffold activity | 17 | GO:0030159 | 0.399 |
| oxidoreductase activity, acting on single donors with incorporation of molecular oxygen, incorporation of two atoms of oxygen | 76 | GO:0016702 | 0.4 |
| structural constituent of myelin sheath | 5 | GO:0019911 | 0.402 |
| carboxy-lyase activity | 22 | GO:0016831 | 0.406 |
| thioredoxin-disulfide reductase activity | 5 | GO:0004791 | 0.407 |
| translation factor activity, nucleic acid binding | 23 | GO:0008135 | 0.407 |
| cell adhesion molecule binding | 32 | GO:0050839 | 0.408 |
| calmodulin binding | 156 | GO:0005516 | 0.409 |
| potassium channel regulator activity | 28 | GO:0015459 | 0.409 |
| DNA topoisomerase activity | 6 | GO:0003916 | 0.412 |
| peroxisome proliferator activated receptor binding | 9 | GO:0042975 | 0.414 |
| cholesterol transporter activity | 14 | GO:0017127 | 0.416 |
| phosphatidylinositol transporter activity | 6 | GO:0008526 | 0.417 |
| protein phosphatase 2B binding | 8 | GO:0030346 | 0.418 |
| very long-chain fatty acid-CoA ligase activity | 5 | GO:0031957 | 0.418 |
| carbohydrate binding | 33 | GO:0030246 | 0.42 |
| voltage-gated potassium channel activity | 80 | GO:0005249 | 0.425 |
| PDZ domain binding | 97 | GO:0030165 | 0.426 |
| 2 iron, 2 sulfur cluster binding | 17 | GO:0051537 | 0.426 |
| unfolded protein binding | 118 | GO:0051082 | 0.427 |
| promoter binding | 189 | GO:0010843 | 0.428 |
| adenylate cyclase binding | 6 | GO:0008179 | 0.429 |
| ionotropic glutamate receptor binding | 19 | GO:0035255 | 0.429 |
| cyclin-dependent protein kinase inhibitor activity | 11 | GO:0004861 | 0.43 |
| 3-beta-hydroxy-delta5-steroid dehydrogenase activity | 5 | GO:0003854 | 0.431 |
| metalloendopeptidase activity | 107 | GO:0004222 | 0.431 |
| phospholipid binding | 70 | GO:0005543 | 0.432 |
| mannosyl-oligosaccharide 1,2-alpha-mannosidase activity | 8 | GO:0004571 | 0.435 |
| single-stranded DNA binding | 62 | GO:0003697 | 0.439 |
| ATP-dependent RNA helicase activity | 18 | GO:0004004 | 0.439 |
| 3',5'-cyclic-GMP phosphodiesterase activity | 15 | GO:0047555 | 0.439 |
| transferase activity, transferring acyl groups other than amino-acyl groups | 19 | GO:0016747 | 0.44 |
| inositol or phosphatidylinositol kinase activity | 14 | GO:0004428 | 0.441 |
| DNA bending activity | 49 | GO:0008301 | 0.441 |
| histone methyltransferase activity | 7 | GO:0042054 | 0.441 |
| structural constituent of nuclear pore | 6 | GO:0017056 | 0.442 |
| diacylglycerol binding | 10 | GO:0019992 | 0.442 |
| GTPase activity | 217 | GO:0003924 | 0.443 |
| BH domain binding | 9 | GO:0051400 | 0.443 |
| protein kinase A regulatory subunit binding | 9 | GO:0034237 | 0.445 |
| cytidine deaminase activity | 7 | GO:0004126 | 0.446 |
| alpha-tubulin binding | 16 | GO:0043014 | 0.447 |
| receptor antagonist activity | 6 | GO:0048019 | 0.447 |
| protein heterodimerization activity | 312 | GO:0046982 | 0.45 |
| ligand-gated sodium channel activity | 9 | GO:0015280 | 0.452 |
| adenylate cyclase activity | 13 | GO:0004016 | 0.453 |
| cyclin binding | 15 | GO:0030332 | 0.455 |
| ryanodine-sensitive calcium-release channel activity | 5 | GO:0005219 | 0.457 |
| Hsp70 protein binding | 15 | GO:0030544 | 0.457 |
| phosphatidylethanolamine binding | 5 | GO:0008429 | 0.46 |
| 2-oxoglutarate-dependent dioxygenase activity | 5 | GO:0010302 | 0.461 |
| alpha(1,3)-fucosyltransferase activity | 7 | GO:0046920 | 0.467 |
| hydrolase activity, acting on ester bonds | 33 | GO:0016788 | 0.468 |
| alpha-2A adrenergic receptor binding | 5 | GO:0031694 | 0.468 |
| 4 iron, 4 sulfur cluster binding | 25 | GO:0051539 | 0.469 |
| histone demethylase activity (H3-K36 specific) | 7 | GO:0051864 | 0.47 |
| adenylate kinase activity | 7 | GO:0004017 | 0.473 |
| endoribonuclease activity, producing 5'-phosphomonoesters | 5 | GO:0016891 | 0.476 |
| serotonin binding | 10 | GO:0051378 | 0.477 |
| magnesium ion transmembrane transporter activity | 6 | GO:0015095 | 0.478 |
| P-P-bond-hydrolysis-driven protein transmembrane transporter activity | 10 | GO:0015450 | 0.48 |
| dolichyl-diphosphooligosaccharide-protein glycotransferase activity | 10 | GO:0004579 | 0.485 |
| angiotensin type II receptor activity | 5 | GO:0004945 | 0.486 |
| protein-hormone receptor activity | 8 | GO:0016500 | 0.487 |
| endodeoxyribonuclease activity, producing 5'-phosphomonoesters | 8 | GO:0016888 | 0.491 |
| eukaryotic initiation factor 4E binding | 6 | GO:0008190 | 0.498 |
| phosphatase activity | 103 | GO:0016791 | 0.498 |
| cyclosporin A binding | 7 | GO:0016018 | 0.5 |
| GABA receptor binding | 9 | GO:0050811 | 0.502 |
| calcium ion binding | 625 | GO:0005509 | 0.504 |
| poly(A) RNA binding | 12 | GO:0008143 | 0.505 |
| monoamine transmembrane transporter activity | 7 | GO:0008504 | 0.505 |
| transcription cofactor activity | 48 | GO:0003712 | 0.508 |
| ligand-dependent nuclear receptor binding | 16 | GO:0016922 | 0.508 |
| thiolester hydrolase activity | 9 | GO:0016790 | 0.514 |
|  |  |  |  |
| **Cellular component** |  |  |  |
| clathrin adaptor complex | 23 | GO:0030131 | 0.009 |
| axon part | 15 | GO:0033267 | 0.01 |
| tight junction | 83 | GO:0005923 | 0.011 |
| lamellipodium | 99 | GO:0030027 | 0.011 |
| CUL4 RING ubiquitin ligase complex | 11 | GO:0080008 | 0.013 |
| actin filament | 39 | GO:0005884 | 0.016 |
| Axin-APC-beta-catenin-GSK3B complex | 7 | GO:0034747 | 0.017 |
| Schmidt-Lanterman incisure | 8 | GO:0043220 | 0.017 |
| AP-type membrane coat adaptor complex | 6 | GO:0030119 | 0.018 |
| endomembrane system | 91 | GO:0012505 | 0.019 |
| insulin-responsive compartment | 5 | GO:0032593 | 0.021 |
| holo TFIIH complex | 12 | GO:0005675 | 0.022 |
| nonmotile primary cilium | 9 | GO:0031513 | 0.022 |
| striated muscle thin filament | 6 | GO:0005865 | 0.023 |
| basal lamina | 18 | GO:0005605 | 0.027 |
| transcription factor TFIID complex | 20 | GO:0005669 | 0.029 |
| PcG protein complex | 10 | GO:0031519 | 0.029 |
| growth cone | 91 | GO:0030426 | 0.032 |
| insoluble fraction | 64 | GO:0005626 | 0.034 |
| microtubule associated complex | 34 | GO:0005875 | 0.034 |
| AP-2 adaptor complex | 10 | GO:0030122 | 0.034 |
| focal adhesion | 112 | GO:0005925 | 0.035 |
| cell cortex | 112 | GO:0005938 | 0.035 |
| gamma-tubulin ring complex | 5 | GO:0008274 | 0.039 |
| motile primary cilium | 7 | GO:0031512 | 0.041 |
| trans-Golgi network membrane | 6 | GO:0032588 | 0.041 |
| DNA-directed RNA polymerase III complex | 8 | GO:0005666 | 0.042 |
| unconventional myosin complex | 7 | GO:0016461 | 0.042 |
| ruffle | 76 | GO:0001726 | 0.048 |
| Cul4A-RING ubiquitin ligase complex | 7 | GO:0031464 | 0.048 |
| recycling endosome | 36 | GO:0055037 | 0.048 |
| RNA-induced silencing complex | 8 | GO:0016442 | 0.05 |
| cAMP-dependent protein kinase complex | 10 | GO:0005952 | 0.051 |
| sex chromatin | 6 | GO:0001739 | 0.053 |
| BRCA1-A complex | 6 | GO:0070531 | 0.056 |
| zonula adherens | 8 | GO:0005915 | 0.058 |
| HOPS complex | 11 | GO:0030897 | 0.062 |
| exosome (RNase complex) | 15 | GO:0000178 | 0.063 |
| anaphase-promoting complex | 25 | GO:0005680 | 0.064 |
| cullin-RING ubiquitin ligase complex | 10 | GO:0031461 | 0.066 |
| chromatin assembly complex | 6 | GO:0005678 | 0.067 |
| smooth endoplasmic reticulum | 11 | GO:0005790 | 0.071 |
| stress fiber | 46 | GO:0001725 | 0.072 |
| ubiquitin ligase complex | 63 | GO:0000151 | 0.073 |
| integral to nuclear inner membrane | 5 | GO:0005639 | 0.075 |
| platelet dense tubular network membrane | 8 | GO:0031095 | 0.076 |
| VCB complex | 7 | GO:0030891 | 0.077 |
| cell-cell adherens junction | 39 | GO:0005913 | 0.081 |
| apicolateral plasma membrane | 7 | GO:0016327 | 0.082 |
| U5 snRNP | 7 | GO:0005682 | 0.083 |
| Ada2/Gcn5/Ada3 transcription activator complex | 14 | GO:0005671 | 0.084 |
| cell-cell junction | 92 | GO:0005911 | 0.084 |
| small-subunit processome | 8 | GO:0032040 | 0.084 |
| early endosome | 117 | GO:0005769 | 0.085 |
| transcriptional repressor complex | 38 | GO:0017053 | 0.088 |
| Nup107-160 complex | 10 | GO:0031080 | 0.088 |
| chromatin remodeling complex | 17 | GO:0016585 | 0.089 |
| U4/U6 x U5 tri-snRNP complex | 8 | GO:0046540 | 0.089 |
| histone acetyltransferase complex | 10 | GO:0000123 | 0.09 |
| THO complex | 5 | GO:0000347 | 0.091 |
| THO complex part of transcription export complex | 5 | GO:0000445 | 0.091 |
| Golgi stack | 30 | GO:0005795 | 0.091 |
| phosphorylase kinase complex | 5 | GO:0005964 | 0.091 |
| early endosome membrane | 50 | GO:0031901 | 0.091 |
| MOZ/MORF histone acetyltransferase complex | 5 | GO:0070776 | 0.092 |
| laminin-1 complex | 7 | GO:0005606 | 0.093 |
| actin cytoskeleton | 178 | GO:0015629 | 0.094 |
| mitochondrial nucleoid | 34 | GO:0042645 | 0.094 |
| catalytic step 2 spliceosome | 78 | GO:0071013 | 0.096 |
| intermediate filament cytoskeleton | 37 | GO:0045111 | 0.097 |
| lateral loop | 6 | GO:0043219 | 0.1 |
| cytoplasmic mRNA processing body | 35 | GO:0000932 | 0.103 |
| ruffle membrane | 42 | GO:0032587 | 0.105 |
| nuclear speck | 125 | GO:0016607 | 0.109 |
| collagen type IV | 6 | GO:0005587 | 0.11 |
| postsynaptic density | 120 | GO:0014069 | 0.111 |
| transcription factor TFTC complex | 14 | GO:0033276 | 0.112 |
| Cul3-RING ubiquitin ligase complex | 10 | GO:0031463 | 0.113 |
| protein-DNA complex | 10 | GO:0032993 | 0.114 |
| nuclear body | 30 | GO:0016604 | 0.115 |
| costamere | 15 | GO:0043034 | 0.115 |
| intercalated disc | 13 | GO:0014704 | 0.12 |
| basal part of cell | 8 | GO:0045178 | 0.123 |
| basement membrane | 84 | GO:0005604 | 0.124 |
| transcription export complex | 6 | GO:0000346 | 0.125 |
| vesicular fraction | 9 | GO:0042598 | 0.125 |
| mRNA cap binding complex | 11 | GO:0005845 | 0.126 |
| DNA-directed RNA polymerase II, holoenzyme | 8 | GO:0016591 | 0.126 |
| axonal growth cone | 6 | GO:0044295 | 0.126 |
| myosin II complex | 6 | GO:0016460 | 0.129 |
| nuclear pore | 65 | GO:0005643 | 0.131 |
| adherens junction | 43 | GO:0005912 | 0.132 |
| transcription elongation factor complex | 14 | GO:0008023 | 0.137 |
| delta DNA polymerase complex | 5 | GO:0043625 | 0.139 |
| PML body | 66 | GO:0016605 | 0.141 |
| microfibril | 7 | GO:0001527 | 0.143 |
| rough endoplasmic reticulum membrane | 7 | GO:0030867 | 0.143 |
| intercellular canaliculus | 9 | GO:0046581 | 0.144 |
| beta-catenin destruction complex | 6 | GO:0030877 | 0.147 |
| cytoplasmic dynein complex | 13 | GO:0005868 | 0.148 |
| Golgi transport complex | 10 | GO:0017119 | 0.148 |
| protein phosphatase type 2A complex | 19 | GO:0000159 | 0.149 |
| cytoskeleton | 858 | GO:0005856 | 0.15 |
| endocytic vesicle | 33 | GO:0030139 | 0.15 |
| microtubule organizing center | 100 | GO:0005815 | 0.156 |
| kinetochore microtubule | 5 | GO:0005828 | 0.156 |
| polysomal ribosome | 6 | GO:0042788 | 0.156 |
| Golgi membrane | 413 | GO:0000139 | 0.159 |
| FHF complex | 5 | GO:0070695 | 0.162 |
| integrator complex | 12 | GO:0032039 | 0.163 |
| neuronal cell body membrane | 11 | GO:0032809 | 0.164 |
| sarcolemma | 79 | GO:0042383 | 0.164 |
| protein serine/threonine phosphatase complex | 12 | GO:0008287 | 0.166 |
| pericentriolar material | 15 | GO:0000242 | 0.167 |
| nuclear matrix | 73 | GO:0016363 | 0.169 |
| mediator complex | 31 | GO:0016592 | 0.172 |
| nuclear chromosome, telomeric region | 15 | GO:0000784 | 0.173 |
| DNA replication factor C complex | 5 | GO:0005663 | 0.173 |
| histone deacetylase complex | 30 | GO:0000118 | 0.175 |
| spectrin | 9 | GO:0008091 | 0.175 |
| trans-Golgi network | 90 | GO:0005802 | 0.177 |
| nuclear inclusion body | 5 | GO:0042405 | 0.178 |
| coated pit | 50 | GO:0005905 | 0.181 |
| CD40 receptor complex | 11 | GO:0035631 | 0.181 |
| chromatin silencing complex | 7 | GO:0005677 | 0.187 |
| chromatin | 103 | GO:0000785 | 0.188 |
| cortical actin cytoskeleton | 20 | GO:0030864 | 0.189 |
| stress granule | 15 | GO:0010494 | 0.191 |
| CRD-mediated mRNA stability complex | 5 | GO:0070937 | 0.191 |
| spliceosomal complex | 120 | GO:0005681 | 0.192 |
| centrosome | 196 | GO:0005813 | 0.192 |
| hemidesmosome | 9 | GO:0030056 | 0.194 |
| integral to mitochondrial outer membrane | 9 | GO:0031307 | 0.195 |
| heterotrimeric G-protein complex | 37 | GO:0005834 | 0.199 |
| dystrophin-associated glycoprotein complex | 17 | GO:0016010 | 0.199 |
| nucleoplasm | 800 | GO:0005654 | 0.2 |
| transcription factor TFIIA complex | 5 | GO:0005672 | 0.203 |
| actomyosin | 5 | GO:0042641 | 0.205 |
| Golgi apparatus part | 6 | GO:0044431 | 0.205 |
| inclusion body | 8 | GO:0016234 | 0.207 |
| recycling endosome membrane | 25 | GO:0055038 | 0.211 |
| clathrin coat of trans-Golgi network vesicle | 8 | GO:0030130 | 0.213 |
| clathrin coat of coated pit | 6 | GO:0030132 | 0.213 |
| mitochondrial intermembrane space protein transporter complex | 6 | GO:0042719 | 0.214 |
| spindle | 107 | GO:0005819 | 0.215 |
| midbody | 64 | GO:0030496 | 0.215 |
| filopodium | 42 | GO:0030175 | 0.217 |
| exocyst | 8 | GO:0000145 | 0.22 |
| nBAF complex | 12 | GO:0071565 | 0.22 |
| synaptosome | 144 | GO:0019717 | 0.226 |
| kinetochore | 86 | GO:0000776 | 0.227 |
| nucleolar ribonuclease P complex | 7 | GO:0005655 | 0.227 |
| eukaryotic translation initiation factor 2B complex | 6 | GO:0005851 | 0.227 |
| nuclear lamina | 8 | GO:0005652 | 0.229 |
| cyclin-dependent protein kinase holoenzyme complex | 10 | GO:0000307 | 0.23 |
| DNA replication factor A complex | 7 | GO:0005662 | 0.23 |
| cytoplasmic membrane-bounded vesicle | 110 | GO:0016023 | 0.23 |
| prefoldin complex | 9 | GO:0016272 | 0.23 |
| npBAF complex | 11 | GO:0071564 | 0.23 |
| nuclear heterochromatin | 17 | GO:0005720 | 0.234 |
| integral to peroxisomal membrane | 14 | GO:0005779 | 0.234 |
| ER-Golgi intermediate compartment | 41 | GO:0005793 | 0.234 |
| pseudopodium | 9 | GO:0031143 | 0.234 |
| integral to Golgi membrane | 45 | GO:0030173 | 0.235 |
| membrane coat | 31 | GO:0030117 | 0.242 |
| dendritic spine | 87 | GO:0043197 | 0.244 |
| mitochondrial proton-transporting ATP synthase complex, catalytic core F(1) | 5 | GO:0000275 | 0.245 |
| smooth endoplasmic reticulum membrane | 6 | GO:0030868 | 0.247 |
| calcium channel complex | 8 | GO:0034704 | 0.247 |
| nuclear membrane | 114 | GO:0031965 | 0.248 |
| extracellular matrix | 194 | GO:0031012 | 0.253 |
| histone methyltransferase complex | 18 | GO:0035097 | 0.253 |
| collagen | 80 | GO:0005581 | 0.254 |
| mitochondrial inner membrane presequence translocase complex | 11 | GO:0005744 | 0.254 |
| respiratory chain | 49 | GO:0070469 | 0.255 |
| mitochondrial large ribosomal subunit | 23 | GO:0005762 | 0.257 |
| meiotic cohesin complex | 6 | GO:0030893 | 0.258 |
| SAGA-type complex | 5 | GO:0070461 | 0.258 |
| kinesin complex | 21 | GO:0005871 | 0.259 |
| mitochondrial envelope | 25 | GO:0005740 | 0.26 |
| dendrite cytoplasm | 15 | GO:0032839 | 0.263 |
| SWI/SNF complex | 15 | GO:0016514 | 0.27 |
| NuRD complex | 15 | GO:0016581 | 0.27 |
| exocytic vesicle | 7 | GO:0070382 | 0.27 |
| Golgi lumen | 26 | GO:0005796 | 0.271 |
| Golgi-associated vesicle membrane | 6 | GO:0030660 | 0.271 |
| histone pre-mRNA 3'end processing complex | 6 | GO:0071204 | 0.272 |
| MLL1 complex | 27 | GO:0071339 | 0.273 |
| synaptonemal complex | 23 | GO:0000795 | 0.276 |
| microvillus | 39 | GO:0005902 | 0.281 |
| transcription factor TFIIIC complex | 7 | GO:0000127 | 0.282 |
| centriole | 43 | GO:0005814 | 0.282 |
| NURF complex | 6 | GO:0016589 | 0.283 |
| PTW/PP1 phosphatase complex | 6 | GO:0072357 | 0.284 |
| anchored to external side of plasma membrane | 6 | GO:0031362 | 0.285 |
| male pronucleus | 5 | GO:0001940 | 0.286 |
| protein phosphatase type 1 complex | 6 | GO:0000164 | 0.287 |
| nuclear chromatin | 47 | GO:0000790 | 0.287 |
| PCAF complex | 6 | GO:0000125 | 0.288 |
| glycosylphosphatidylinositol-N-acetylglucosaminyltransferase (GPI-GnT) complex | 7 | GO:0000506 | 0.288 |
| integral to mitochondrial inner membrane | 6 | GO:0031305 | 0.288 |
| clathrin-coated vesicle | 41 | GO:0030136 | 0.291 |
| ATP-binding cassette (ABC) transporter complex | 10 | GO:0043190 | 0.293 |
| synaptic vesicle membrane | 50 | GO:0030672 | 0.294 |
| sarcoglycan complex | 6 | GO:0016012 | 0.296 |
| female pronucleus | 7 | GO:0001939 | 0.298 |
| intracellular membrane-bounded organelle | 219 | GO:0043231 | 0.298 |
| cytoplasmic microtubule | 31 | GO:0005881 | 0.3 |
| phagocytic vesicle | 7 | GO:0045335 | 0.3 |
| pi-body | 5 | GO:0071546 | 0.302 |
| AMP-activated protein kinase complex | 7 | GO:0031588 | 0.305 |
| BBSome | 8 | GO:0034464 | 0.305 |
| proteinaceous extracellular matrix | 284 | GO:0005578 | 0.307 |
| neuron projection membrane | 7 | GO:0032589 | 0.308 |
| autophagic vacuole | 16 | GO:0005776 | 0.309 |
| chromocenter | 8 | GO:0010369 | 0.311 |
| Z disc | 61 | GO:0030018 | 0.311 |
| microtubule cytoskeleton | 87 | GO:0015630 | 0.312 |
| AP-1 adaptor complex | 7 | GO:0030121 | 0.317 |
| chromosome, telomeric region | 29 | GO:0000781 | 0.322 |
| chromatoid body | 9 | GO:0033391 | 0.323 |
| excitatory synapse | 11 | GO:0060076 | 0.323 |
| axon | 196 | GO:0030424 | 0.325 |
| cleavage furrow | 29 | GO:0032154 | 0.332 |
| integral to membrane of membrane fraction | 26 | GO:0000299 | 0.333 |
| microtubule basal body | 49 | GO:0005932 | 0.333 |
| paranode region of axon | 6 | GO:0033270 | 0.333 |
| cell projection | 307 | GO:0042995 | 0.333 |
| ER-Golgi intermediate compartment membrane | 17 | GO:0033116 | 0.336 |
| vacuolar membrane | 10 | GO:0005774 | 0.339 |
| caveola | 59 | GO:0005901 | 0.339 |
| myosin complex | 55 | GO:0016459 | 0.339 |
| plasma membrane enriched fraction | 7 | GO:0001950 | 0.34 |
| eukaryotic translation initiation factor 4F complex | 8 | GO:0016281 | 0.342 |
| nuclear envelope | 113 | GO:0005635 | 0.344 |
| eukaryotic translation initiation factor 3 complex | 13 | GO:0005852 | 0.344 |
| clathrin coated vesicle membrane | 13 | GO:0030665 | 0.344 |
| neuron projection | 137 | GO:0043005 | 0.344 |
| flotillin complex | 7 | GO:0016600 | 0.353 |
| stereocilium | 19 | GO:0032420 | 0.353 |
| Golgi cisterna membrane | 65 | GO:0032580 | 0.354 |
| heterochromatin | 22 | GO:0000792 | 0.355 |
| rough microsome | 6 | GO:0019718 | 0.355 |
| nuclear inner membrane | 28 | GO:0005637 | 0.356 |
| pronucleus | 13 | GO:0045120 | 0.357 |
| small ribosomal subunit | 24 | GO:0015935 | 0.358 |
| clathrin vesicle coat | 5 | GO:0030125 | 0.361 |
| lamin filament | 5 | GO:0005638 | 0.364 |
| TRAPP complex | 5 | GO:0030008 | 0.364 |
| azurophil granule | 6 | GO:0042582 | 0.365 |
| ribonucleoprotein complex | 270 | GO:0030529 | 0.367 |
| nuclear telomere cap complex | 9 | GO:0000783 | 0.37 |
| zymogen granule membrane | 11 | GO:0042589 | 0.373 |
| Ino80 complex | 14 | GO:0031011 | 0.375 |
| Sin3 complex | 9 | GO:0016580 | 0.376 |
| centriolar satellite | 7 | GO:0034451 | 0.376 |
| small nuclear ribonucleoprotein complex | 17 | GO:0030532 | 0.377 |
| dynactin complex | 7 | GO:0005869 | 0.385 |
| gamma-tubulin complex | 5 | GO:0000930 | 0.388 |
| sarcoplasmic reticulum | 39 | GO:0016529 | 0.388 |
| intrinsic to endoplasmic reticulum membrane | 19 | GO:0031227 | 0.389 |
| synaptic cleft | 5 | GO:0043083 | 0.39 |
| lamellipodium membrane | 11 | GO:0031258 | 0.392 |
| cytosolic large ribosomal subunit | 37 | GO:0022625 | 0.397 |
| presynaptic active zone | 12 | GO:0048786 | 0.397 |
| nonhomologous end joining complex | 6 | GO:0070419 | 0.399 |
| peroxisome | 101 | GO:0005777 | 0.403 |
| compact myelin | 5 | GO:0043218 | 0.405 |
| SCF ubiquitin ligase complex | 21 | GO:0019005 | 0.406 |
| exon-exon junction complex | 11 | GO:0035145 | 0.407 |
| platelet alpha granule lumen | 46 | GO:0031093 | 0.413 |
| I band | 16 | GO:0031674 | 0.414 |
| intracellular organelle | 11 | GO:0043229 | 0.415 |
| basal plasma membrane | 30 | GO:0009925 | 0.418 |
| apical part of cell | 79 | GO:0045177 | 0.418 |
| cytosolic part | 8 | GO:0044445 | 0.42 |
| protein complex | 318 | GO:0043234 | 0.421 |
| apical junction complex | 9 | GO:0043296 | 0.423 |
| mitochondrial inner membrane | 316 | GO:0005743 | 0.424 |
| WASH complex | 6 | GO:0071203 | 0.426 |
| clathrin coat | 9 | GO:0030118 | 0.429 |
| spindle pole centrosome | 6 | GO:0031616 | 0.433 |
| condensed nuclear chromosome | 35 | GO:0000794 | 0.436 |
| telomerase holoenzyme complex | 6 | GO:0005697 | 0.438 |
| extrinsic to membrane | 35 | GO:0019898 | 0.438 |
| lipid particle | 23 | GO:0005811 | 0.439 |
| tRNA-splicing ligase complex | 5 | GO:0072669 | 0.439 |
| granular component | 5 | GO:0001652 | 0.44 |
| cell projection membrane | 6 | GO:0031253 | 0.444 |
| cilium membrane | 13 | GO:0060170 | 0.444 |
| pre-autophagosomal structure | 5 | GO:0000407 | 0.445 |
| condensed chromosome outer kinetochore | 8 | GO:0000940 | 0.446 |
| chromosome, centromeric region | 118 | GO:0000775 | 0.447 |
| membrane-bounded vesicle | 13 | GO:0031988 | 0.447 |
| lateral plasma membrane | 28 | GO:0016328 | 0.448 |
| heterogeneous nuclear ribonucleoprotein complex | 18 | GO:0030530 | 0.456 |
| spherical high-density lipoprotein particle | 7 | GO:0034366 | 0.456 |
| XY body | 11 | GO:0001741 | 0.46 |
| Golgi medial cisterna | 5 | GO:0005797 | 0.46 |
| cilium axoneme | 45 | GO:0035085 | 0.461 |
| plasma membrane part | 7 | GO:0044459 | 0.462 |
| aminoacyl-tRNA synthetase multienzyme complex | 7 | GO:0017101 | 0.464 |
| cell periphery | 14 | GO:0071944 | 0.464 |
| endoplasmic reticulum lumen | 105 | GO:0005788 | 0.465 |
| junctional sarcoplasmic reticulum membrane | 6 | GO:0014701 | 0.465 |
| cytosolic small ribosomal subunit | 32 | GO:0022627 | 0.466 |
| lamellar body | 10 | GO:0042599 | 0.469 |
| clathrin sculpted monoamine transport vesicle membrane | 5 | GO:0070083 | 0.469 |
| glycogen granule | 7 | GO:0042587 | 0.472 |
| aggresome | 15 | GO:0016235 | 0.473 |
| ribosome | 178 | GO:0005840 | 0.476 |
| mitochondrial respiratory chain | 15 | GO:0005746 | 0.481 |
| ciliary rootlet | 10 | GO:0035253 | 0.486 |
| juxtaparanode region of axon | 8 | GO:0044224 | 0.488 |
| zymogen granule | 5 | GO:0042588 | 0.493 |
| microtubule | 258 | GO:0005874 | 0.498 |
| cilium | 126 | GO:0005929 | 0.505 |
| HAUS complex | 7 | GO:0070652 | 0.507 |
